# Design and generation of mRNAs encoding conserved regions of SARS-CoV-2 ORF1ab for T cell-mediated immune activation

**DOI:** 10.2217/fvl-2023-0066

**Published:** 2023-06-24

**Authors:** Cao Minh Nguyen, Bac An Luong, Thu Thuy Thi Tran, Hoai Nghia Nguyen, Le Son Tran

**Affiliations:** 1 Medical Genetics Institute, Vietnam; 2 University of Medicine & Pharmacy at Ho Chi Minh City, Vietnam

**Keywords:** IFN-γ, *in vitro* transcription (IVT), mRNA vaccine, ORF1ab, peripheral blood mononuclear cells (PBMCs), SARS-CoV-2, T cell epitopes

## Abstract

**Aim:**

To generate mRNAs encoding conserved regions within SARS-CoV-2 ORF1ab which can induce strong T-cell responses to overcome the immune invasion of newly emergent variants.

**Methods:**

We selected two conserved regions with a high density of T-cell epitopes using immunoinformatics for mRNA synthesis. The ability of testing mRNAs to activate T cells for IFN-γ production was examined by an ELISpot assay and flow cytometry.

**Results:**

Two synthesized mRNAs were successfully translated in MDA-MB-231 cells and had comparable potency to the spike mRNA to induce CD4^+^ and CD8^+^ T-cell responses in peripheral blood mononuclear cells in 29 out of 34 participants.

**Conclusion:**

This study provides a proof-of-concept for the use of SARS-CoV-2 conserved regions to develop booster vaccines capable of eliciting T-cell-mediated immunity.

The outbreak of SARS-CoV-2 has had a devastating impact on many aspects of life [1–3]. SARS-CoV-2 is a positive-sense single-stranded RNA virus with an envelope containing lipid bilayers, spike (S), envelope (E), and membrane (M) proteins on its surface [4,5]. The S protein of SARS-CoV-2 plays an important role in host cell binding and membrane fusion between viral envelope and host cell membrane. DNA or mRNA from S gene has been used in many COVID-19 vaccines, notably the BNT162b2 (Pfizer), mRNA-1273 (Moderna), and ChAdOx1 (AstraZeneca) [6–8]. While vaccines targeting the S protein has been shown to reduce COVID-19 morbidity and mortality [8], the intrinsic ability of SARS-CoV-2 to mutate quickly leads to generation of new viral strains with altered S protein that are capable of evading vaccine-induced immunity [9]. In particular, the highly transmissible SARS-CoV-2 Omicron variant has been shown to escape from immunity elicited by vaccines targeting previous variants of S protein, causing large waves of infection worldwide [10–12].

While much attention has been focused on the humoral aspect of vaccine-induced immunity, the T cell-mediated immunity is a key determinant of clinical outcome after SARS-CoV-2 infection as well as the efficacy of vaccines. By eliminating infected cells, virus-specific T cells are essential for reducing viral multiplication, preventing initial infection, and working alongside with the humoral immunity to provide long-term memory responses [13,14]. It has been shown that memory T cells in the lung, which were detected at 10 months after infection [15], still retained their ability to provide clinical protection even in patients who had shown a significant reduction in neutralizing antibody response only four months post infection [16,17]. Therefore, T cell-based vaccines targeting highly conserved regions of the SARS-CoV-2 genome might be useful in tackling the challenge whereby newly emergent SARS-CoV-2 strains evade the vaccine-induced T-cell immunity protection [18].

SARS-CoV-2 genome is a single-stranded positive sense RNA molecule which allows direct viral protein production inside host cells [19,20]. SARS-CoV-2 enters host cell mainly through the interaction between the S protein and the receptor angiotensin-converting enzyme 2 (ACE2) on target cell [21,22]. After viral entry, viral genomic RNA is translated into two polyproteins pp1a and pp1ab of 4405 and 7096 amino acids, respectively [19]. The cleavage of these two polyproteins generates 16 non-structural proteins (Nsp1 to Nsp16). These proteins are collectively called the replication–transcription complex (RTC), which is required for viral replication and transcription [23]. Unlike S protein, the RTC is highly conserved in SARS-CoV-2 genome and among the Coronaviridae family [19,24]. Multiple epitopes in non-structure proteins were identified within these regions and shown to elicit strong T-cell responses in human [25]. T-cell responses against RTC have been shown to provide protection without seroconversion in healthcare workers and disease-free close contacts [26,27]. Yarmarkovich *et al.* [28] identified multiple T cell-specific epitopes in the pp1ab polyprotein, indicating that targeting ORF1ab, which encodes this polyprotein, could be a promising approach for vaccine development. However, it should be noted that the T-cell epitopes predicted in that study have not yet undergone experimental validation [28]. Additionally, the study did not account for the diverse range of HLA (human leucocyte antigens) types present in different ethnic populations, especially those that are highly prevalent in underrepresented communities.

Therefore, this study aims to address these limitations by analyzing T-cell epitopes within the conserved regions of the SARS-CoV-2 ORF1ab specifically using the prevalent HLA class I and II types found in the Vietnamese population, which were not examined in the previous study by Yarmarkovich *et al.* To validate the expression and immunogenicity of the selected regions, we designed mRNAs encoding those specific regions and subsequently transfected them into human peripheral blood mononuclear cells (PBMCs) to assess their ability to activate T-cell responses.

## Materials & methods

### *In silico* prediction of epitopes & selection of target regions in SARS-CoV-2 *ORF1ab* gene for IVT mRNA synthesis

The HLA class I typing data from the Vietnamese population were retrieved from previous studies (Supplementary Table 1) [29,30] to predict epitopes in ORF1ab (YP_009724389.1) using Tepitool [31] with the following settings: NetMHCpan method, 9-mers peptide and IC50 <500 nM. Subsequently, Vaxijen [32] and class I immunogenicity tool [33] were used to predict if the epitopes predicted by Tepitool could be recognized by CD4^+^ T cell and have high immunogenicity. A threshold value of <50 nM, >0.4, and 0 was applied for Tepitool, Vaxijen, and class I immunogenicity tools respectively to filter epitopes with strong binding affinity to HLA-I and high antigenicity.

Similarly, HLA class II binding epitopes were predicted using Tepitool and the Vietnamese HLA class II typing data retrieved from previous publications (Supplementary Table 1) [29,30,34] with the following settings: NetMHCIIpan method and IC50 <1000 nM. Subsequently, a set of predicted epitopes (input data) was fed into Vaxijen to predict the antigenicity. The threshold values for Tepitool and Vaxijen were set as IC50 <50 nM and >0.4 respectively to select epitopes with strong binding affinity to HLA-II types and high antigenicity [32].

To ensure the stability and translation efficacy of IVT mRNA transcripts, we designed plasmid constructs carrying a coding region of 4410 bp, which had a similar length to the coding sequence of S protein-based mRNA vaccine (BNT162b2) that was previously developed by Pfizer-BioNTech [35–37]. A sliding-window based approach (window size = 1470 aa, step size = 1 aa) was used to select two overlapping regions within the ORF1ab that contain the highest density of HLA type I and II restricted epitopes.

### Plasmid design for IVT mRNA synthesis & cloning strategy

The sequences of selected regions were optimized using the Codon optimization tool (Integrated DNA Technologies, IA, USA). If a BspQI cut site appears, the codons will be changed into synonym codons. Next, we replaced the coding sequence of S protein in the Pfizer's construct [35–37] with our target coding sequences. As a result, the final constructs consisted of a 5′ UTR, a signal peptide, a target coding region, a 3′UTR, and a poly(A) tail. The poly(A) tail was 110 amino acids (aa) in length, consisting of 30 adenosine residues, followed by 10 aa linker sequence and another 70 adenosine residues (Supplementary Figure 2).

To synthesize the mRNAs, we used the HiScribe T7 mRNA Kit with CleanCap^®^ Reagent AG (New England Biolab, E2080, MA, USA). To accommodate the AG capping analog, a compatible T7 promoter sequence was added to the 5′ end of the construct, with a minor modification as indicated in the kit, using 5′-TAATACGACTCACTATAA-3′ instead of 5′-TAATACGACTCACTATA-3′. In addition, a BspQI site was added at the end of poly(A) tail for linearization. Two additional control constructs were generated in this study including: one carrying the same mRNA sequences as BNT162b2 (S), referred to as S and the other undergoin transfection process without addition of mRNA, referred to as mock. The constructs were cloned into pEZclone plasmid provided and synthesized by Epoch Life Science (TX, US).

### mRNA synthesis by *in vitro* transcription

mRNA was synthesized using the HiScribe T7 mRNA Kit with CleanCap Reagent AG (New England Biolab, E2080) in a 20 μl reaction, following the recommended protocol with some modifications [38]. Briefly, 1 μl N1-Methylpseudouridine-5′-Triphosphate (100 mM) (TriLink Biotechnologies, N-1081, CA, USA) was used instead of UTP. 1 μl Pyrophosphatase (New England Biolab, M0361) and 0.5 μl RNase Inhibitor (New England Biolab, M0314) were added to the reaction. After 3 h at 37°C, DNase-1 was added to digest the DNA template at 37°C for 15 min, followed by mRNA purification using Monarch RNA Cleanup Kit (New England Biolab, T2040). The length and integrity of purified mRNA were analyzed on 2% agarose gel.

To verify the mRNA sequence, IVT mRNAs were reverse-transcribed into cDNAs using RevertAid First Strand cDNA Synthesis Kit (Thermo Fisher Scientific, K1622, MA, USA) and random hexamer primers. cDNAs were then ligated with sequencing adaptors using NEBNext Ultra II DNA Library Prep Kit (New England Biolab, E7645). The libraries were sequenced on Nextseq2000 (Illumina, CA, USA) with Nextseq2000 P3 kit (Illumina, CA, USA) for one million reads and mapped to the reference plasmid sequence.

### Verification of Cap-1 attachment to mRNA products

To assess if mRNA products were capped, an assay based on Thermostable RNase H (New England Biolab, M0523) was performed as described in previous publications [39,40]. Briefly, a biotinylated chimera RNA-DNA probe containing 10 2′-O-methylated RNA nucleotides, followed by 6 deoxyribonucleotides (dNs) (5′GACCAGAmAmGmAmAmUmAmCmUmAm3′) was synthesized (Integrated DNA Technologies) [39,40]. Probe hydridization and RNase H digestion was performed with 17 μg of mRNA, 0.6 μl of probe (50 μM), 1.5 μl RNase H (5 U/μl), and 1.5 μl 10X RNase H Reaction Buffer and run as following: 95°C for 2 min, 65°C for 2 min, 55°C for 2 min and 40°C for 60 min. Klenow fill-in was incubated at 25°C for 15 min with 5 μl of hybridization product, 0.6 μl dNTP (2.5 mM), 1.5 μl 10X NEBuffer, and 1.5 μl DNA Polymerase I, Large (Klenow) Fragment (5 U/ul). The reaction was stopped by adding 10 mM EDTA and incubating at 75°C for 20 min. 15 μl of Dyna Bead (Thermo Fisher Scientific) was added to the reaction and incubated at room temperature for 20 min. The beads were washed once with 1X high salt buffer (5 mM Tris-HCl, 0.5 mM EDTA, 1 M NaCl), once with 1X Low salt buffer (5 mM Tris-HCl, 0.5 mM EDTA, 60 mM NaCl), and then eluted with 10 μl of DNA/RNAse-free water. The eluates were analyzed on 21% Urea-PAGE gel at 150 V for 3 h. Gel was stained with SYBRGold (Invitrogen, MA, USA) and visualized by the iBright Imaging system (Invitrogen, MA, USA).

### Verification of correct poly(A) tail expression

To assess if the mRNA had the poly(A) tail as designed, the mRNAs were converted into cDNA using d(T) self-designed primer 5′-GCGACCACCGATTTTTTTTTTTT-3′ and the RevertAid First Strand cDNA Synthesis Kit (Thermo Fisher Scientific). cDNA was PCR amplified using the TaKaRa Taq Hot Start Version (TaKaRa Bio, Kyoto, Japan, R007A) and the primers: 1: 5′-AATGATACGGCGACCACCGA-3′ and 2: 5′-AAGCACGCAGCAATGCAG CT-3′. Amplified DNA products were subjected to Sanger sequencing.

### mRNA electroporation

Electroporation was performed using Neon Transfection System (Thermo Fisher Scientific, MPK5000) and Neon Transfection System 10 μl Kit (Thermo Fisher Scientific, MPK1096). Human breast cancer cell MDA-MB-231 (ATCC) was resuspended in Resuspension Buffer R at the density of 2 × 10^7^ cells/ml. Transfection was performed using a 10 μl tip with 1 μg mRNA with the recommended settings (1400 V, 10 ms, and 4 pulses). Transfected cells were incubated in a humidified incubator containing 5% CO_2_ at 37°C overnight, followed by assessing cell viability by using Zombie Red Fixable Viability Kit (Biolegend, cat. 423109, USA, CA) and immunofluorescence staining.

### Immunofluorescence staining for verification of mRNA translation

After overnight incubation, cells were washed with PBS and fixed with 4 % paraformaldehyde (PFA) at room temperature for 15 min. Cells were then washed three-times with PBS and permeabilized with 0.2% Triton X-100 in PBS at room temperature for 10 min. Permeabilized cells were washed three-times with PBS and incubated with blocking buffer (2% BSA in PBS) at room temperature for 1 h. The cells were then incubated with anti-SARS-CoV-2 3C-like Protease Antibody (Cell Signaling Technology, 51661, MA, USA) diluted at 1:100 in blocking buffer overnight at 4°C. After being washed three-times with PBS, cells were incubated with Goat anti-Rabbit IgG (H + L) Highly Cross-Adsorbed Secondary Antibody, Alexa Fluor 488 (Thermo Fisher Scientific, A-11034) diluted at 1:500 in blocking buffer for 2 h at room temperature in the dark. Cells were counterstained with DAPI (Thermo Fisher Scientific, 62248) at 2 μg/ml for 10 min at room temperature. The cells were mounted onto glass slides with Fluoromount-G Mounting Medium (Thermo Fisher Scientific, 00-4958-02). Images were captured using an Oxion Inverso fluorescence microscope (Euromex, IN, USA) and were analyzed using ImageFocUSlpha (version x64) (Euromex).

### Participant recruitment & PBMC isolation

Blood samples were collected from a total of 34 volunteers who had received various SARS-CoV-2 vaccines. These volunteers were all researchers employed at our institute. As part of our institute's protocol, these individuals were required to undergo a rapid antigen test (Humasis Covid-19Ag test, Humasis Co., Ltd, Gyeonggi-do, Republic of Korea) whenever they experienced symptoms to confirm their SARS-CoV-2 infection status. Among the volunteers who tested positive, we divided them into two groups based on the time elapsed since their positive test result: one group included individuals who had been tested positive within one month prior to the screening, and the other group included individuals who had been tested positive more than one month prior to the screening. On the other hand, volunteers who had never tested positive for SARS-CoV-2 before donating their blood were classified as the ‘unknown’ group.

Written informed consent was obtained from all study participants. This study was approved by the Ethic Committee of Medical Genetics Institute (23QĐ-VDTYH). PBMCs were isolated from 10 ml of whole blood by density gradient centrifugation in Ficoll-Paque PLUS (Cytiva, DC, USA), washed two-times with PBS, resuspended in 90 % FBS, 10 % DMSO and frozen in liquid nitrogen for storage. PBMC cells were thawed at 37°C, seeded in 6-well plate at 2 × 10^6^ cells/ml and cultured in RPMI 1640 Medium, GlutaMAX Supplement (Gibco, MA, USA) supplemented with 10 % FBS, 1 % Pen/Strep, and 50 μM 2-Mercaptoethanol) for two days. Half of the PBMC was supplemented with 100 ng/ml Human GM-CSF Recombinant Protein (Thermo Fisher Scientific) and 10 ng/ml Human IL-4 Recombinant Protein (Thermo Fisher Scientific) for dendritic cell activation while the rest supplied with 10 ng/ml Human IL-2 Recombinant Protein (Thermo Fisher Scientific) to maintain T cell. To mature the dendritic cells, 6 h before electrotransfection, both LPS (Sigma-Aldrich, MA, USA) and Human IFN-γ Recombinant Protein (Thermo Fisher Scientific) were added at 10 ng/ml.

### PBMC transfection

Both non-adherent cells (T cells) and semi-adherent cells (mature dendritic cells) were collected by gently pipetting with cold PBS. T cells were resuspended in RPMI 1640 Medium, GlutaMAX Supplement (Gibco) supplemented with 10% FBS, 0% Pen/strep, and 50 μM 2-Mercaptoethanol to reach 5 × 10^5^ cells/ml. Activated dendritic cells (semi-adherent cells) were suspended in Resuspension Buffer T to obtain 2 × 10^7^ cells/ml and subsequently transfected with 0.5 μg mRNA, using Neon Transfection System (Thermo Fisher Scientific, MA, USA) with the following settings: 10 μl tip, 1500 volt, 30 ms and 1 pulse. The Zombie Red™ Fixable Viability Kit (Biolegend, cat. 423109) was used to assess the viability of electroporated PBMCs as per manufacturer's instructions. Transfected dendritic cells (2 × 10^5^ cells) were mixed with T cells at 1:1 ratio and seeded in 96-wells plates before performing ELISpot assay.

### HLA typing by Sanger sequencing

HLA I types of participants were determined as previously described [41]. Genomic DNA was extracted from the whole blood of 14 participants using GeneJET Whole Blood Genomic DNA Purification Mini Kit (Thermo Fisher Scientific, MA, US, K0781). Isolated genomic DNA was amplified with the following primers: 5A2 (5′CCC AGA CGC CGA GGA TGG CCG'3) and 3A2 (5′GCA GGG CGG AAC CTC AGA GTC ACT CTC T'3), using 30 cycles of 98°C for 10s, 70°C for 30s, 72°C for 33s. PCR products were cleaned up using Kappa pure bead (Roche, Basel, Switzerland) with the ratio of 0.6x and then subjected to Sanger sequencing using exon 2 primers (5′GTTTCATTTTCAGTTTAGGCCA'3 and 5′TTACCCGGTTTCATT TTCAG'3) and exon 3 primers (5′TGTTGGTCCCAATTGTCTCCCCTC'3 and 5′TCCTTGTG-GGAGGCCAG'3). The results were analyzed using SOAPTyping tool as described [42].

### ELISpot assay

To evaluate the T-cell response when stimulated with the mRNA, Human IFN-γ ELISpotPRO kit (ALP) (Mabtech, 3420-2AST-2, Nacka Strand, Sweden) was used to measure IFN-γ secretion by PBMCs, following the manufacturer's instruction. Briefly, the ELISpot strips were washed five-times with 200 μl PBS, blocked with 200 μl fresh RPMI (10% FBS) medium for 30 min. Transfected cells were seeded into strips at 2 × 10^5^ cells per well in 200 μl medium. After 24 h, the medium was removed, and the wells were washed five-times with 200 μl PBS, followed by addition of 100 μl of 7-B6-ALP (diluted 200-times in PBS, 0.5% FBS) and 2 h incubation at room temperature. The wells were then washed five-times with PBS. 100 μl of filtered BCIP/NBT-plus choromogen substrate was added to the wells for 5 to 15 min. The color development was stopped by extensively washing with tap water. The dried wells were visualized using Mabtech ASTOR ELISpot Reader (Mabtech) and the spot counts were analyzed by Mabtech Apex software (Mabtech). The reactivity was defined as the increase in the number of spots when PBMCs transfected with target mRNAs were compared with mock.

### Flow cytometry

To identify the T-cell subset that secretes the IFN-γ, flow cytometry intracellular staining (ICS) was performed using BD Cytofix/Cytoperm Plus Kit (BD Bioscience, NJ, USA) and appropriate antibodies. Transfected denritic cells and T cells from the co-culture experiments were seeded in a 96-well plate and incubated in 5% CO_2_ for 48 h and supplemented with 1 μl BD GolgiStop. After 6 h, cells were harvested and washed two-times with PBS before being incubated with Human TruStain FcX (Biolegend) for 15 min at 4°C for Fc receptor blocking. Cells were then washed twice with FACS buffer (PBS, 2% FBS) and incubated in the dark, at 4°C for 30 min with PerCP anti-human CD3 Antibody (Biolegend, 300326), PE/Cyanine7 anti-human CD4 Antibody (Biolegend, 300511), and APC/Cyanine7 anti-human CD8 Antibody (Biolegend, 344713) diluted at 1:100 in FACS buffer. Cells were washed twice with FACS buffer, followed by addition of 250 μl of Fixation/Permeabilization solution, the mixture was then incubated for 20 min at 4°C in the dark. Next, the cells were washed twice with 1 × BD Perm/Wash buffer. The cells were evenly divided into two sets for staining with two panels. For the first set of cells, we added FITC anti-human IL-4 Antibody (Biolegend, 500807) and APC anti-human IFN-γ Antibody (Biolegend, 502512) at a dilution of 1:100 in 1 × BD Perm/Wash buffer. In the second set, we added APC anti-human TNF-α Antibody (Biolegend, 502913) and FITC anti-human IL-2 Antibody (Biolegend, 500304). All cells were then incubated overnight at 4°C in the dark. Afterward, the cells were washed twice with 1 × BD Perm/Wash buffer and acquired on a flow cytometer (BD Accuri C6, BD Bioscience, NJ, USA).

### Statistical analysis

The one-way ANOVA (analysis of variance) was used to compare the means of three groups of PBMC electroporated with S, P1, P2 mRNA and mock.

## Results

### Selection of regions with high T cell epitope density in the SARS-CoV-2 ORinstitu1ab

**Figure 1.**
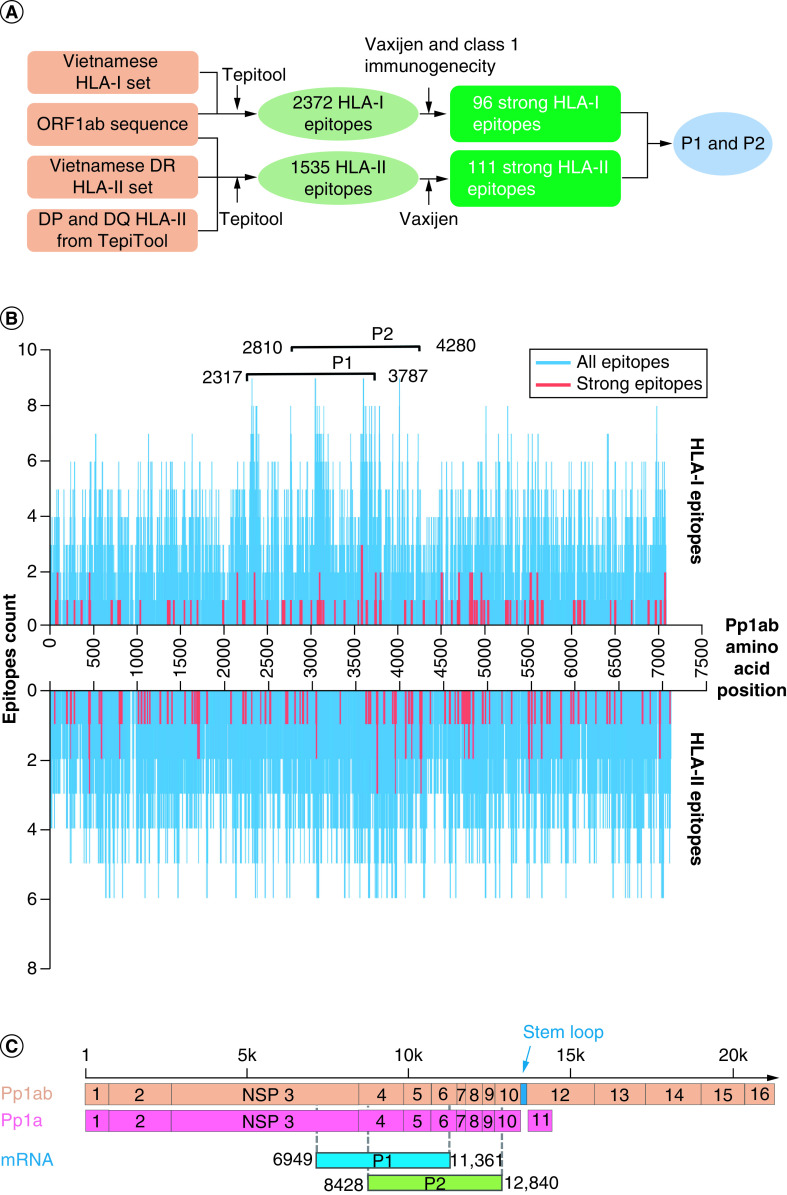
Profiling T-cell epitope landscape of SARS-CoV-2 ORF1ab polyproteins. **(A)** Workflow to predict and analyze T cell epitopes. T cell epitopes were predicted based upon the ORF1ab amino acid sequence and the HLA-I and HLA-II data retrieved from the Vietnamese population using Tepitool with the default settings. Strong epitopes were filtered using Vaxijen and class 1 immunogenicity tool. **(B)** The distributions of the HLA-I restricted epitopes (top) and HLA-II restricted epitopes (bottom) within the ORF1ab. The blue line represents all the epitopes predicted by Tepitool while the red line shows the strong epitopes by Vaxijen and class 1 immunogenicity tool (IC50 <50 nM, Vaxijen >0.4 and class 1 immunogenicity tool >0). Sliding window analysis (step = 1) was used to select P1 and P2 regions containing the highest density of T-cell epitopes. **(C)** Mapping of P1 and P2 regions to viral Pp1ab and Pp1a polyproteins. P1 and P2 cover several non-structure proteins of SARS-CoV-2 (from NSP3 to NSP10).

The open reading frame 1ab (ORF1ab) region was previously shown to be one of the most conserved regions of the SARS-CoV-2 genome [19,24]. Therefore, we employed the workflow presented in Figure 1A to identify T cell epitopes within this region that could be presented by the most common HLA-I and HLA-II types among Vietnamese populations. By using the Tepitool (IC50 threshold value <500 nM), we identified a total of 2372 epitopes predicted to bind to HLA-I alleles (Figure 1A & Supplementary Table 2). Of those binding epitopes, we selected strong binders (IC50 <50 nM) to further evaluate their antigenicity by using Vaxijen (threshold value >0.4) and class I immunogenicity (threshold value >0) tool. As a result, we identified a list of 96 epitopes with strong binding affinity to HLA and high immunogenicity (Figure 1A & Supplementary Table 2). Similarly, using the set of Vietnamese HLA-II alleles, we identified 1535 epitopes predicted to bind to these HLA-II types by using Tepitool (threshold value <1000 nM). By VaxiJen, we also identified a list of 111 epitopes with strong binding affinity to HLA-II and strong immunogenicity (Figure 1A & Supplementary Table 2).

We next examined the distribution of predicted binding epitopes along the amino acid sequence of the pp1ab polypeptide sequence (Figure 1B). By using a sliding window approach, we selected two overlapping regions of 1470 aa, named P1 (aa 2317 to 3787) and P2 (aa 2810 to 4280), spanning the region with the highest density of epitopes predicted to bind to both HLA-I and HLA-II (Figure 1B). The selected P1 region covered 25% (592/2372) and 22% (338/1535) of HLA-I and HLA-II restricted epitopes, respectively. Of those epitopes, 25% (24/96) and 16% (18/111) were predicted to be strong HLA-I and HLA-II binders, respectively, and had high immunogenicity (Table 1). Likewise, the P2 region had comparable epitope coverages with 24% (571/2372) for HLA-I and 23% (350/1535) for HLA-II epitopes (Table 1). Compared with P1, P2 covered a lower proportion of strong HLA-I binding epitopes (23% vs 25%, Table 1) but a higher proportion of strong HLA-II binding epitopes (23% vs 16%, Table 1). Those strong binding epitopes were predicted to preferably bind to HLA-A*02:06 (8.24% and 9.15% for P1 and P2, respectively) and HLA-DRB1*10:01 (15.93% and 17.5% for P1 and P2, respectively, Supplementary Figure 1). We next mapped the P1 and P2 regions to the SARS-CoV-2 genome and found that P1 region overlapped with the entire sequences of Nsp4 and Nsp5 while P2 covered the entire sequences of Nsp5-Nsp9 (Figure 1C).

**Table 1.** Number of epitopes and strong binding epitopes covered by P1 and P2 regions.

	P1		P2		Total epitopes
	Epitopes	Coverage	Epitopes	Coverage	
HLA-I	592	25%	571	24%	2372
Strong HLA-I	24	25%	22	23%	96
HLA-II	338	22%	350	23%	1535
Strong HLA-II	18	16%	26	23%	111

### *In vitro* synthesis of P1 & P2 mRNA

**Figure 2.**
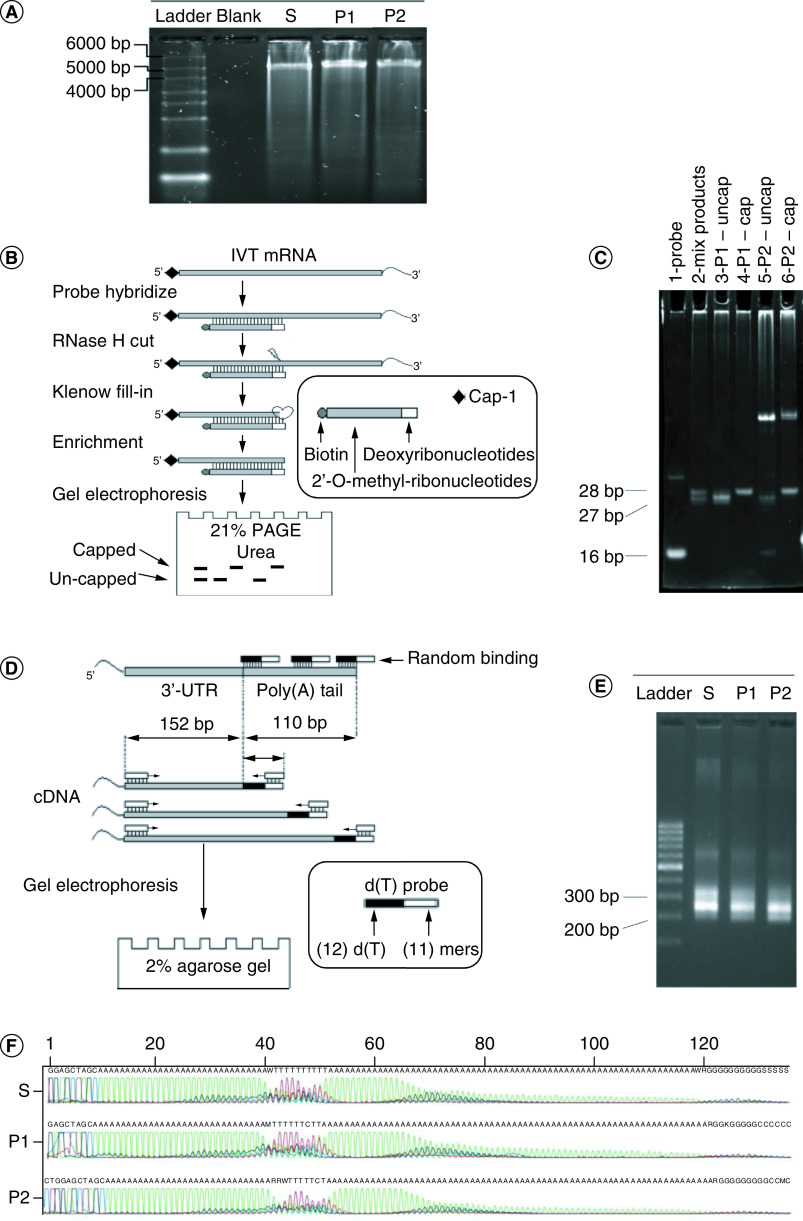
*In vitro* synthesis of mRNA encoding P1 and P2. **(A)** Agarose gel electrophoresis of *in vitro* transcribed mRNA P1 and P2. The full-length mRNA products were detected at less than 5 kb. **(B)** Detection of 5′ capped mRNA by RNase H cleavage assay. A biotin-chimeric probe was designed to bind in the 5′UTR of the mRNA products. RNase H cleavage resulted in a jagged end which was subsequently filled by the Klenow enzyme. The hybrid products were pulled down and then analyzed on 21% Urea PAGE denaturing gel, which can detect one ribonucleotide difference between the fragments with or without cap-1. **(C)** Urea PAGE denaturing gel electrophoresis of the capped or uncapped P1 and P2. P1 and P2 were synthesized with and without the AG cap analog and subjected to the RNase H cleavage assay. The products were run on 21% Urea PAGE denaturing gel (lanes 3–6). The probe (lane 1) and mix products (lane 2) consisting of both capped and uncapped P1 were run as visualization references. **(D)** An assay measuring the length of mRNA poly(A) tail. d(T) primers with an adapter and a primer bound to 5′UTR were designed to convert mRNA to cDNA, followed by amplification of the poly(A) tails. The amplified cDNA products were subsequently analyzed by Sanger sequencing and agarose gel electrophoresis. **(E)** The length of poly(A) tails of S, P1 and P2 mRNA were analyzed by the assay described in **(D)** using 2% agarose gel. **(F)** The sequence of amplified cDNA products from the poly(A) tail analysis assay analyzed by Sanger sequencing.

We next constructed DNA plasmids for IVT mRNAs encoding the P1 and P2 regions based on the design of Pfizer-BioNTech's (BNT162b2) IVT plasmid [35–37] (Supplementary Figure 2). As expected, we detected the full-length IVT mRNA products (4928 bp) of P1, P2, and the control spike protein S1 on agarose gel (Figure 2A).

To quantify the capping efficiency, we used a previously described method which is based on the generation of 5′cleavage ends of mRNA by biotin-tagged probes and RNase H (Figure 2B) [41,42]. The cleavage fragments were analysed on denaturing urea polyacrylamide gel to differentiate the capped from the uncapped products (Figure 2B). We found that P1 mRNA showed a single band of 27 bp corresponding to uncapped product when capping reagents were not added (Lane 3, Figure 2C). In contrast, a single band with 1 bp longer was oberserved upon supplementation with capping reagents (Lane 4, Figure 2C), suggesting that P1 mRNA was successfully capped. Likewise, we obtained similar results for P2 mRNA in lane 4 and 6 (Figure 2C). In these lanes, we observed unexpected products which could be due to the nonspecific binding of the hybrid capture probes to P2 mRNA.

To verify whether P1 and P2 mRNAs contain the full-length poly(A) tail, we carried out reverse transcription using our customized probes composed of 12 dT and 11 mers to convert mRNAs to cDNAs, followed by PCR amplification (Figure 2D). The PCR products were expected to have various lengths, ranging from 152 to 362 bp due to different binding sites of probes to poly(A) tail (Figure 2D). We detected multiple fragments of various sizes, ranging from 250 bp to 350 bp, with the brightest band at 280 bp for S, P1, and P2 products (Figure 2E). To verify the presence of full-length poly-A tail sequence (110-bp long), we performed Sanger sequencing on PCR products. The sequencing results showed a full poly(A) tail of 110 nucleotides for all IVT mRNA products (Figure 2F). Taken together, we demonstrated that we successfully synthesised IVT P1 and P2 mRNAs with 5′ cap and 3′ poly(A) tail.

### Detection of P1 & P2 mRNA translation in cells

**Figure 3.**
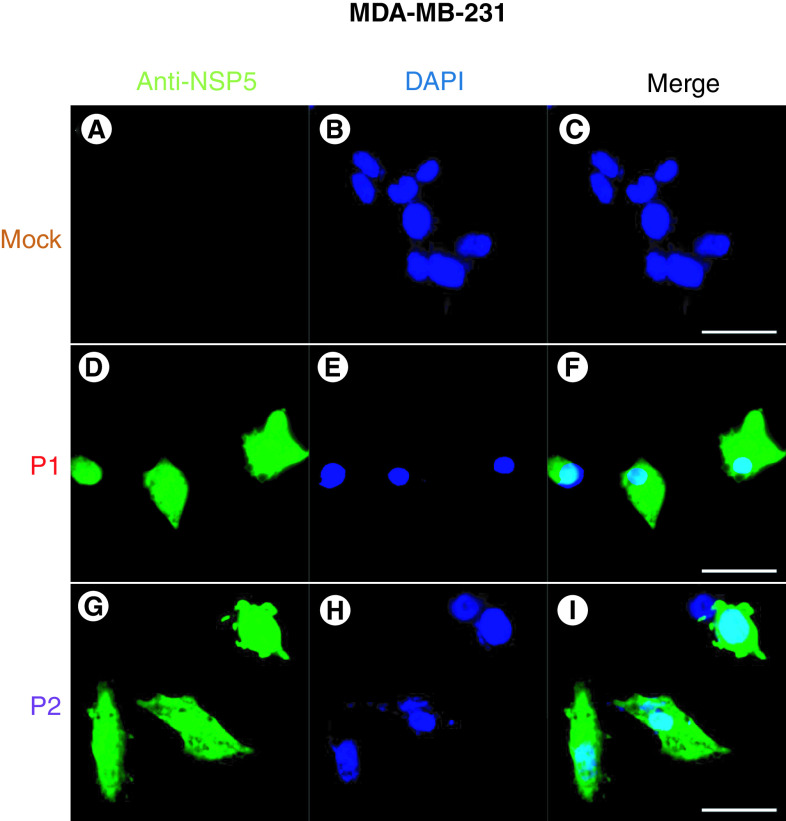
Expression of P1 and P2 mRNAs in human cells. Immunofluorescence images (scale bar = 20 μm) of MDA-MB-231 cells transfected with **(A–C)** no mRNA, **(D–F)** P1 mRNA, or **(G–I)** P2 mRNA. Transfected cells were stained with anti-NSP5 antibody (green) to detect the expression of P1 and P2 mRNAs. Cellular nuclei were stained by DAPI (blue).

To examine the translation of IVT mRNAs encoding P1 and P2 regions in human cells, we transfected MDA-MB-231 cells with IVT mRNA of P1 and P2 by electroporation. With our electroporation procedure, we observed that more than 80% of cells remained viable 24 hours after electroporation, and there were no significant differences in the viability of cells electroporated with different mRNAs (Supplementary Figure 3). Since both P1 and P2 mRNAs encode for Nsp5 (Figure 1C), we stained transfected cells with anti-Nsp5 antibody. There was no signal detected in cells not electroporated (mock condition) (Figure 3A–C) while we detected the expression of P1 and P2 mRNAs both in the nucleus and cytoplasm of MDA-MB-231 cells (Figure 3D–F & G–I) . Thus, these data confirmed the translation of IVT mRNAs encoding P1 and P2 in the human MDA-MB-231 cells. We observed consistent results when transfecting HEK293T cells with P1 and P2 mRNAs (Supplementary Figure 4). These results indicated that P1 and P2 mRNA can be translated in many human cell types.

### P1 & P2 mRNAs induced IFN-γ production in both CD4^+^ & CD8^+^ T cells

To investigate the immunogenicity of P1 and P2 mRNAs in triggering T-cell responses, we enrolled a total of 34 participants, and their demographic information and viral infection status are summarized in Table 2. Among the participants, females constituted a slightly higher proportion (56%) compared with males (44%, Table 2). The median age of the group was 25 years. The majority of participants (94.1%, 32/34, Table 2) had received three doses of the SARS-CoV-2 vaccine, except for two individuals (participant 26 and 29, Supplementary Table 3) who had received only two doses. Due to limited vaccine availability at the time, participants received a combination of different vaccine types, including DNA-based AstraZeneca, mRNA-based Pfizer-BioNTech and Moderna, or whole-cell-based Vero vaccine (Table 2). Supplementary Table 3 provides detailed information regarding the vaccination dates and the time elapsed between vaccination and blood donation for each participant. It is noteworthy that all participants had HLA types that matched the common HLA types observed among the Vietnamese population (Supplementary Table 3). Based on the duration since the last active infection, participants were categorized into three groups (Table 2). Among the 34 recruited participants, 14 had tested positive for SARS-CoV-2 by a rapid antigen test and exhibited symptoms within the past month (less than 1 month), while 12 participants had tested positive for the virus more than one month ago (>1 month). The remaining 8 participants (unknown) had never tested positive or experienced any symptoms before donating their blood.

**Table 2.** The demographic information and infection status of 34 participants.

		<1 month (n = 14)		>1 month (n = 12)		Unknown (n = 8)		All	
Age	Median	27		24		26		25	
	Min	23		22		24		22	
	Max	33		40		40		40	

		n	%	n	%	n	%	n	%
Sex	Female	9	64%	5	42%	5	63%	19	56%
	Male	5	36%	7	58%	3	38%	15	44%
Vaccinations (n)	2	0	0%	1	8%	1	13%	2	6%
	3	14	100%	11	92%	7	88%	32	94%

**Figure 4.**
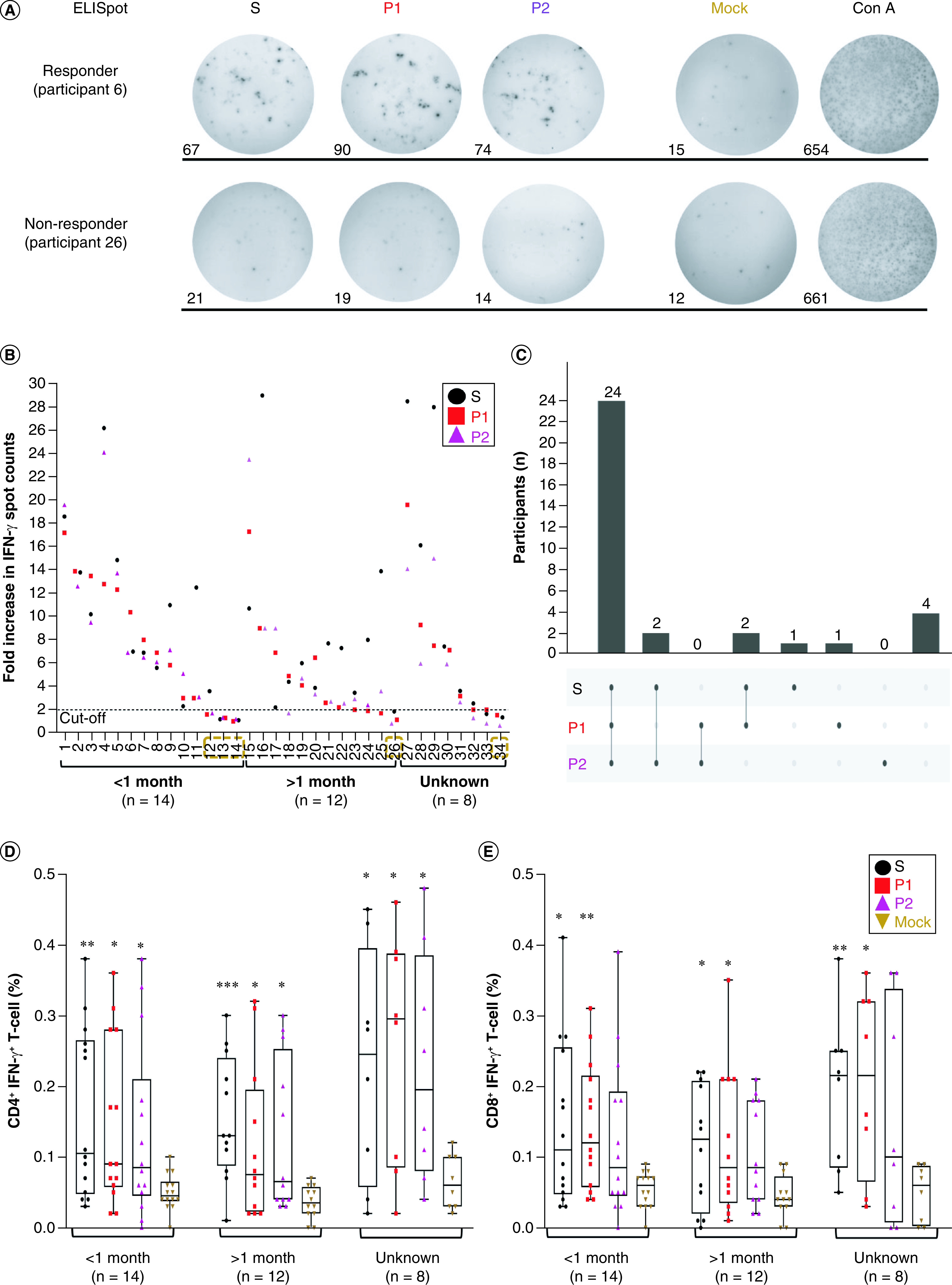
P1 or P2 mRNA induces IFN-γ production from CD4 and CD8 T cells. **(A)** Representative ELISpots of PBMCs transfected with S, P1, P2, no mRNA (mock) or PBMCs treated with ConA (positive control) from participant 6 (responder) and 26 (non-responder). The number of spot counts per well was indicated. **(B)** Fold increase of IFN-γ spot counts of PBMCs after transfection with S, P1, or P2 mRNA relative to the mock treatment from 34 participants. Participants having a fold increase less than 2 (cutoff value) were classified as non-responders. **(C)** UpSet plot showing the number of participants having T-cell responses to S, P1 or P2 mRNA, as well as the overlap between such conditions. Percentages of **(D)** CD4^+^ IFN-γ^+^ T cells and **(E)** CD8^+^ IFN-γ^+^ T cells measured by flowcytometry from 34 participants, grouped into <1 month, >1 month, and unknown, after transfection with S, P1, or P2 mRNA. One-way ANOVA test was performed to compare the frequencies of CD4^+^ IFN-γ^+^ T cells and CD8^+^ IFN-γ^+^ T cells across different groups; *p < 0.05; **p < 0.01; ***p < 0.001.

PBMCs from 34 participants were electroporated with S, P1, P2 mRNAs or no mRNA (mock). One day after electroporation, we observed that the viability of PBMCs was similar across all groups, with more than 80% of cells remaining viable (Supplementary Figure 5A). As a positive control, PBMCs were stimulated with concanavalin A (conA), which resulted in the highest levels of IFN-γ secretion. In contrast, electroporation without mRNA (mock) led to negligible levels of IFN-γ production in PBMCs (Figure 4A). To assess T-cell responses, we compared the fold changes in the number of IFN-γ spots generated by PBMCs electroporated with S, P1, or P2 mRNA relative to those without mRNA. Participants with fold increases greater than 2 were considered responders, while those below this cutoff were classified as non-responders (Figure 4A) [43]. For visual representation, we plotted the fold increases in ELISpot data in descending order within each group. We observed that the majority of participants in each group exhibited detectable IFN-γ responses following electroporation with P1 or P2 mRNA compared with mock stimulation (Figure 4B). Specifically, among participants in the <1 month group, 11 out of 14 (78.6%) were responders, while in the >1 month group, 11 out of 12 (91.7%) participants showed a response. In the unknown group, 7 out of 8 (87.5%) participants were responders (Figure 4B & Supplementary Figure 5B). Furthermore, there were no significant differences in the levels of IFN-γ response to both P1 and P2 mRNAs among the three participant groups (Figure 4B & Supplementary Figure 5B), indicating that P1 and P2 mRNAs could induce T-cell responses independent of participants' past infection status. Among the 29 responders across all groups, 24 participants (70.6%) responded to both P1 and P2 mRNAs, while 5 participants (8.8%) showed responses to either P1 (3 participants) or P2 (2 participants) mRNA alone (Figure 4C). Additionally, we found that the participant coverage for P1 mRNA was comparable to that of S mRNA, which induced IFN-γ secretion in 27 out of 34 participants (79.4%) (Figure 4C). Thus, our data demonstrated that epitopes derived from P1 and P2 mRNAs could be presented and trigger T-cell responses in PBMCs from the majority of participants, regardless of their infection status.

We next performed intracellular staining for IFN-γ by flow cytometry to profile CD4^+^ and CD8^+^ T-cell responses to P1 or P2 mRNA (Supplementary Figure 5C). Consistent with ELISpot data, we detected a significant increase in the frequencies of IFN-γ producing CD4^+^ T cells upon stimulation with either P1 or P2 mRNAs compared with mock stimulation regardless of participants' past infection status (0.09% and 0.09% for P1 mRNA and P2 mRNA, respectively vs 0.05% for mock, p < 0.001, Figure 4D & Supplementary Figure 6A). Regarding CD8^+^ T-cell activation, stimulation with P1 mRNA significantly increased the frequencies of IFN-γ producing cells in all participant groups (0.13% for P1 mRNA vs 0.05% for mock, p < 0.0001, Figure 4E & Supplementary Figure 6B), while P2 mRNA exhibited a lower level of IFN-γ activation and did not achieve statistically significant induction in any of the three participant groups (0.09% for P2 mRNA vs 0.05% for mock, p < 0.01, Figure 4E & Supplementary Figure 6B). In alignment with IFN-γ findings, stimulation with P1 mRNA led to significant increases in CD4^+^ T TNF-α^+^ frequencies (0.07% for P1 mRNA vs 0.05% for mock, p < 0.05, Supplementary Figure 6C) and CD8^+^ TNF-α^+^ T-cell frequencies (0.065% for P2 mRNA vs 0.05% for mock, p < 0.05, Supplementary Figure 6D), whereas no significant induction of these cell subsets was observed following stimulation with P2 mRNA (p > 0.05, Supplementary Figure 6C & D).

In contrast to IFN-γ and TNF-α responses, we were unable to detect any significant changes in IL-2 or IL-4 production in all participants across the three groups after transfection with either S, P1, or P2 mRNA (Supplementary Figure 6E–H). Thus, our data demonstrate that P1 mRNA has the capability to activate both CD4^+^ and CD8^+^ T cells in participants, irrespective of their past infection status, resulting in a preferential production of IFN-γ and TNF-α. These two major cytokines, characteristic of Th1 cells, play a vital role in promoting T cell-mediated immunity, which is crucial for offering comprehensive protection against the virus. In contrast, P2 mRNA primarily activates CD4^+^ T cells but demonstrates a weaker activation of CD8^+^ T cells.

## Discussion

In this study, we described a new strategy to construct a SARS-CoV-2 mRNA vaccine that targets a conserved region in SARS-CoV-2 genome, the ORF1ab. The advantage of this vaccine over the existing SARS-CoV-2 mRNA vaccines is that it could avoid immune escape by not targeting the spike (S) protein which is known to easily acquire multiple mutations [9–12]. The identification of ORF1ab as a potential target for SARS-CoV-2 vaccine has been described in previous studies [28,44–48]. However, these studies mainly focused on *in silico* predictions of potential candidates, lacking experimental validation of their immunogenicity. In our study, we developed a comprehensive workflow that involved both *in silico* selection and experimental validation of target regions for their expression and immunogenicity in PBMCs of volunteers. Through this workflow, we identified two overlapping regions, P1 and P2, which were selected based on their high densities of T-cell epitopes predicted to bind to HLA-I and HLA-II types highly prevalent in the Vietnamese populations (Figure 1). Importantly, we successfully demonstrated that mRNAs encoding these two regions were capable of activating T-cell responses in PBMCs from the majority of participants, irrespective of their previous infection status.

To obtain mRNA with both a 5′ cap and a poly(A) tail, we designed DNA plasmid constructs for *in vitro* transcription (IVT) reactions (Figure 2). We successfully demonstrated that the IVT-synthesized mRNAs could be effectively translated in human cells, as evidenced by the expression of the Nsp5 protein (Figure 3 & Supplementary Figure 4). Subsequently, we assessed the ability of P1 and P2 mRNAs, in comparison with S mRNA, to activate T-cell responses in PBMCs obtained from 34 participants. As expected, stimulation with S mRNA resulted in IFN-γ production, as detected by the ELISpot assay, in the majority of participants (29/34, 85.3%, Figure 4). Similarly, stimulation with P1 or P2 mRNA induced T cell responses in 29 out of 34 participants' PBMCs (85.3%, Figure 4C), indicating that both P1 and P2 mRNAs could effectively activate T cell-mediated responses in a substantial proportion of the participants. Interestingly, we did not observe a significant difference in IFN-γ induction between participants with less than 1 month or more than 1 month post-active infection, suggesting that the response to P1 and P2 mRNAs could arise from either newly primed T cells or memory T cells. Among the participants, there were five individuals who did not exhibit detectable responses to either P1 or P2 mRNA. This lack of response could be attributed to the limitations of our assay, which primarily detects recall responses, or the participants' prior infection status. Out of these five non-responders, three participants (11, 12 and 13) had recently experienced active infection for less than 1 month, and one participant (34) had never tested positive prior to blood donation, indicating a possible absence of a memory response to SARS-CoV-2. The remaining non-responder was participant 26, who had experienced active infection more than 5 months ago, suggesting that the memory response to the virus might have waned over time. Therefore, it is imperative to conduct serological tests for SARS-CoV-2 antibodies in future studies to evaluate the virus-specific immune memory in these participants.

In line with our ELISpot findings, we consistently observed a notable increase in IFN-γ production by both CD4^+^ and CD8^+^ T cells upon stimulation with P1 mRNA, regardless of participants' infection status. In contrast, stimulation with P2 mRNA did not lead to a statistically significant increase in the frequencies of CD8^+^ IFN-γ^+^ T cells. This observation can be attributed to the lower number of HLA-I epitopes mapped to the P2 region compared with the P1 region (592 vs 571 for HLA-I epitopes, Table 1). Furthermore, we consistently detected a similar trend in TNF-α responses, indicating that P1 mRNA could be presented by both HLA-I and HLA-II, thereby activating both CD8^+^ and CD4^+^ T cells to produce Th1-associated cytokines. On the other hand, P2 mRNA appeared to be predominantly presented by HLA-II, primarily activating CD4^+^ T cells.

Our study is subject to several limitations that should be acknowledged. Firstly, the sample size of our study was relatively small. To obtain a more accurate estimation of the coverage of P1 and P2 mRNAs in the Vietnamese population, a larger cohort should be included in future studies. Secondly, due to the limitations of our assay primarily detecting recall responses, we were unable to determine whether the observed T-cell responses were derived from *de novo* priming or recall responses. Given that more than 90% of our population had been infected with SARS-CoV-2 or vaccinated [49–51], distinguishing between these two response types becomes challenging. Access to immunological assays determining baseline responses to viral capsid or spike proteins was not available during the recruitment stage, which limited our ability to differentiate and interpret the origin of the T-cell responses. Therefore, it is crucial to perform antibody tests measuring viral-specific antibodies in the serum to confirm participants' infection status. Thirdly, the T-cell responses measured by ELISpot or flow cytometry were directed toward the whole P1 and P2 polyproteins. To identify the specific epitopes responsible for triggering T-cell responses and IFN-γ production, it is necessary to design a set of peptides corresponding to potential epitopes and assess their capacity to activate T-cell responses. Lastly, the success of a mRNA vaccine should be evaluated based on factors such as uptake, biodistribution, expression levels, and the ability to provide protection against the virus. These aspects were not addressed in our study. Therefore, future investigations should focus on examining whether P1 or P2 mRNA can effectively trigger T-cell responses *in vivo* on animal models and provide neutralization of the virus.

## Conclusion

In conclusion, our study utilized epitope prediction tools and a public HLA dataset from an underrepresented population, specifically Vietnam, to design and synthesize two mRNAs encoding regions with the highest densities of T cell epitopes. We successfully demonstrated that these IVT mRNAs can be effectively translated in human cells and activate robust CD4^+^ and CD8^+^ T-cell responses in PBMCs from the Vietnamese population. These findings establish a proof of concept for the development of mRNA vaccines targeting conserved regions of viral proteins, which can address the challenge of reduced efficacy in existing vaccines caused by the emergence of new SARS-CoV-2 variants. Furthermore, our workflow holds the potential to serve as a rapid strategy for screening potential candidates in the development of mRNA-based vaccines targeting tumor antigens.

**Summary points**

- The emergence of new mutations in the spike (S) protein of SARS-CoV-2 variants has posed challenges to the effectiveness of existing vaccines.
- T cell-mediated immune responses, known for their role in impeding virus replication and clearing infected cells, are crucial for combating viral infections. Therefore, developing vaccines that target conserved viral regions and elicit strong T-cell responses is essential.
- This study aimed to design and generate *in vitro* transcribed (IVT) mRNAs encoding conserved regions within the open reading frame 1ab (ORF1ab) of SARS-CoV-2, capable of inducing robust T-cell responses.
- Immunoinformatics tools were employed to select conserved regions enriched with T-cell epitopes, and plasmid vectors were designed for mRNA synthesis.
- The translation efficiency of the synthesized mRNAs in human cells was confirmed through an immunofluorescence assay. Additionally, the ability of the mRNA constructs to activate T cells from human peripheral blood mononuclear cells (PBMCs) for interferon-gamma (IFN-γ) production was evaluated using an enzyme-linked immunospot (ELISpot) assay and flow cytometry.
- The successful translation of the two synthesized mRNAs was observed in MDA-MB-231 and HEK293T cells, confirming their functional capacity.
- Notably, the P1 mRNA exhibited comparable potency to the spike mRNA in inducing both CD4^+^ and CD8^+^ T-cell responses in PBMCs from 29 out of 34 participants (85.3%). Similarly, the P2 mRNA triggered CD4^+^ T-cell responses in the majority of patients, although it showed a weaker induction of CD8^+^ T cells compared with the P1 mRNA.
- This study provides a proof-of-concept for utilizing conserved regions within the SARS-CoV-2 genome to develop booster vaccines that can effectively elicit T cell-mediated immunity. By targeting these conserved regions, the vaccines may overcome the challenges posed by emerging viral variants and contribute to enhanced immune responses against SARS-CoV-2.
