# Supplementary figures and images for: Design and generation of mRNAs encoding conserved regions of SARS-CoV-2 ORF1ab for T cell-mediated immune activation
Source: Future Virol. 2023 Jun 24;18(8):501–16. doi: 10.2217/fvl-2023-0066 (PMC10308627; doi:10.2217/fvl-2023-0066)

# HLA class I

# HLA class II

**A**

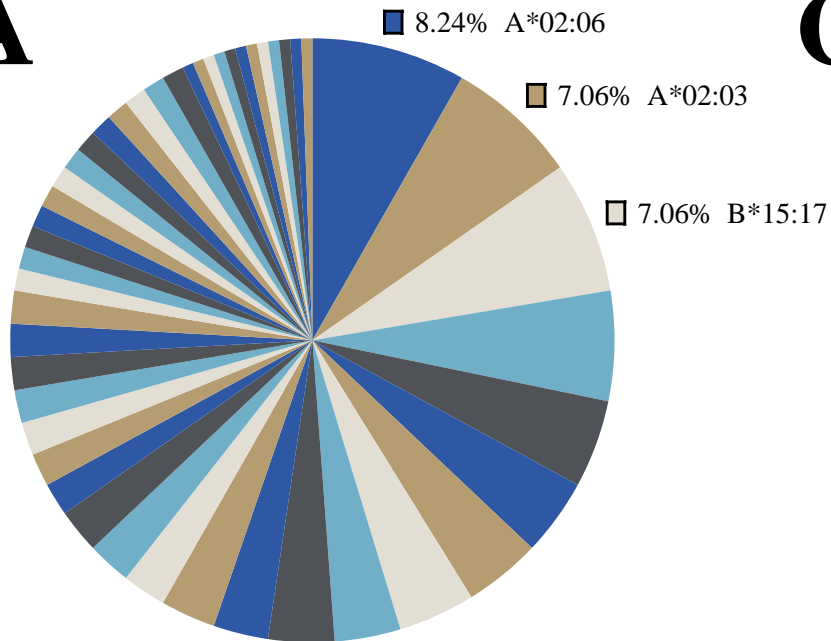

**C**

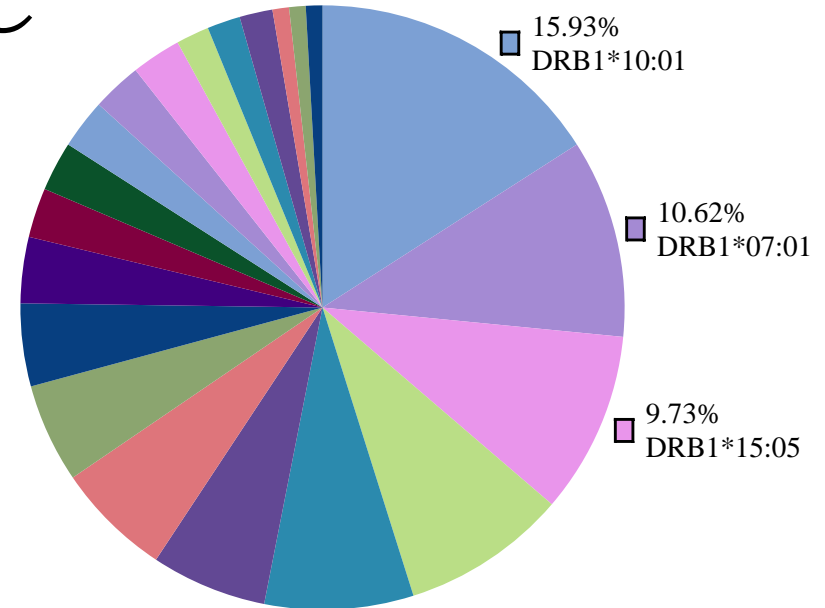

**B**

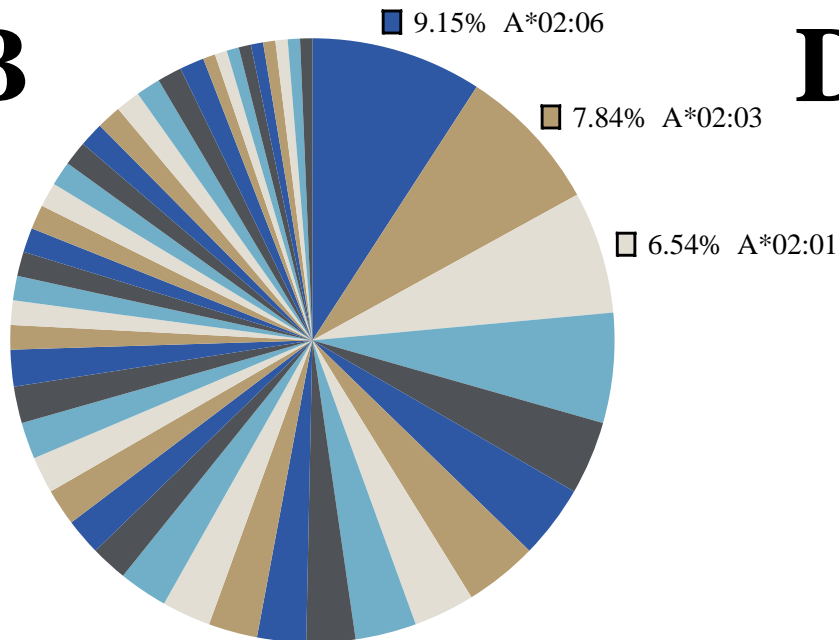

**D**

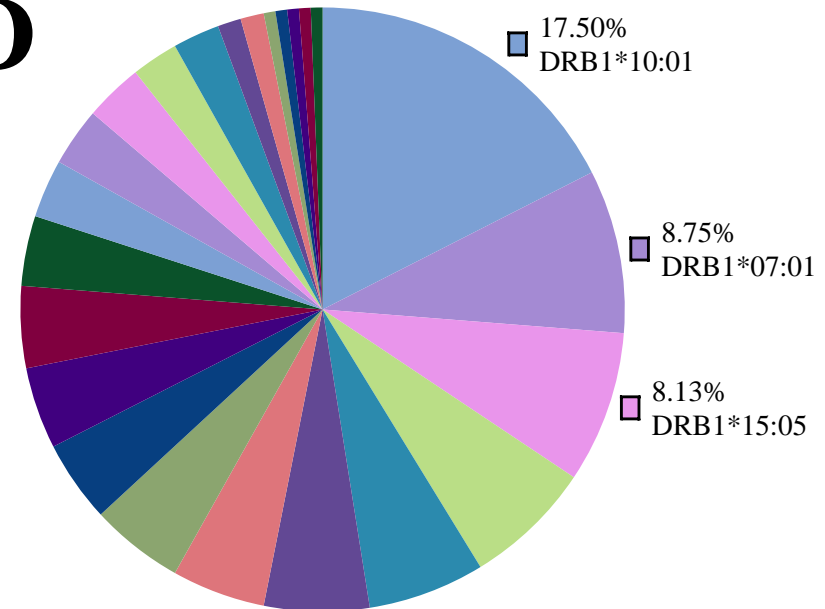

**P1**

**P2**

Supplement: Supplementary file 1 [file fvl-18-501-s1.pdf]

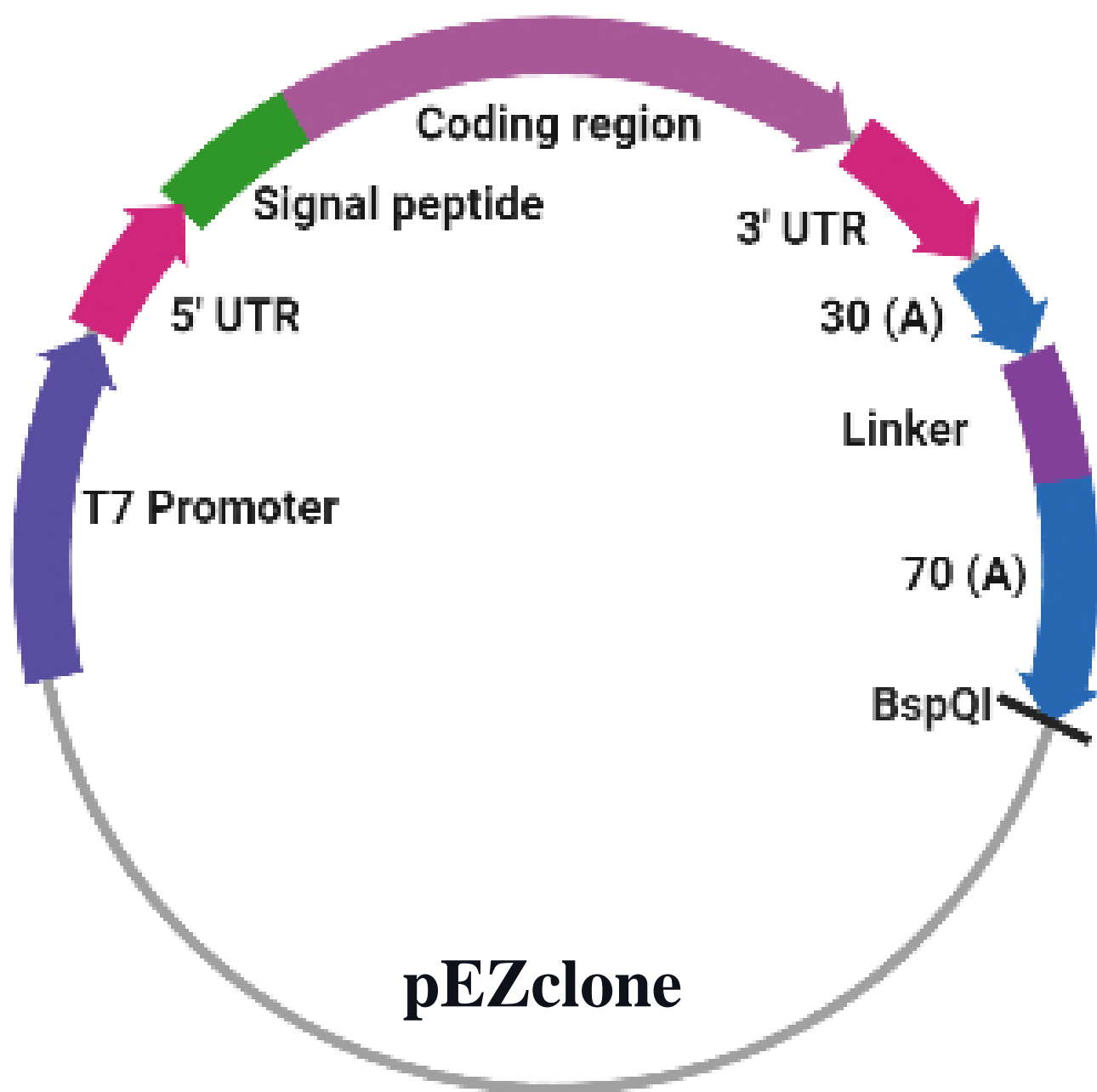

Supplement: Supplementary file 2 [file fvl-18-501-s2.pdf]

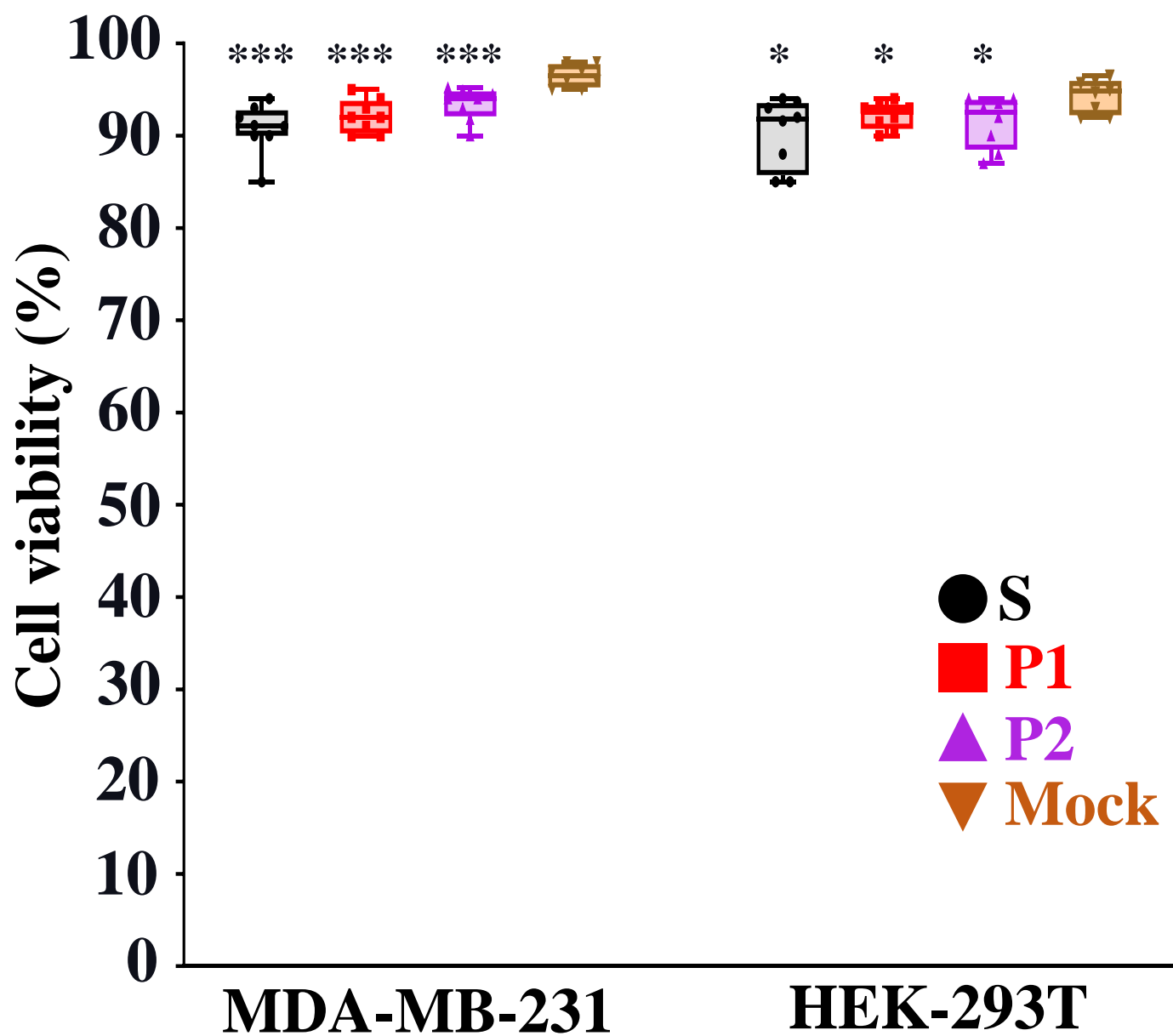

Supplement: Supplementary file 3 [file fvl-18-501-s3.pdf]

HEK-293T

Anti-NSP5

DAPI

Merge

Mock

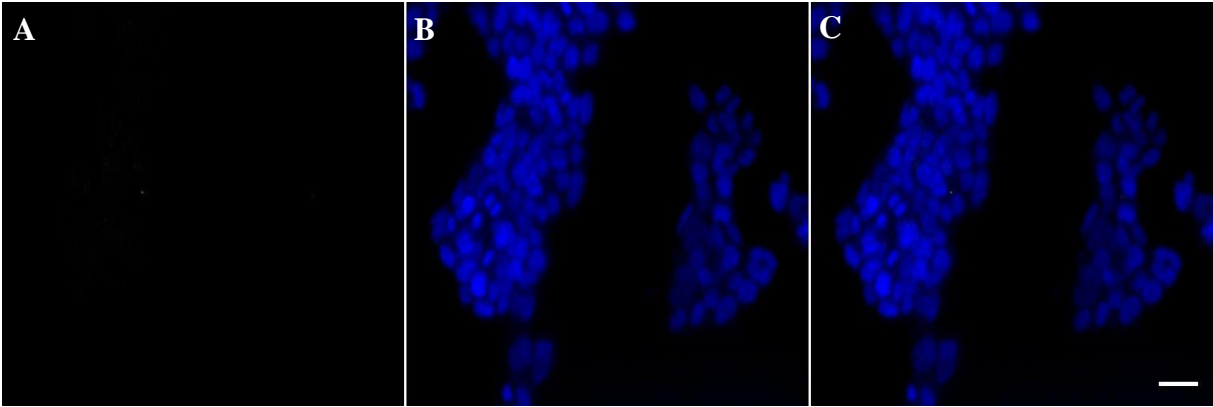

P1

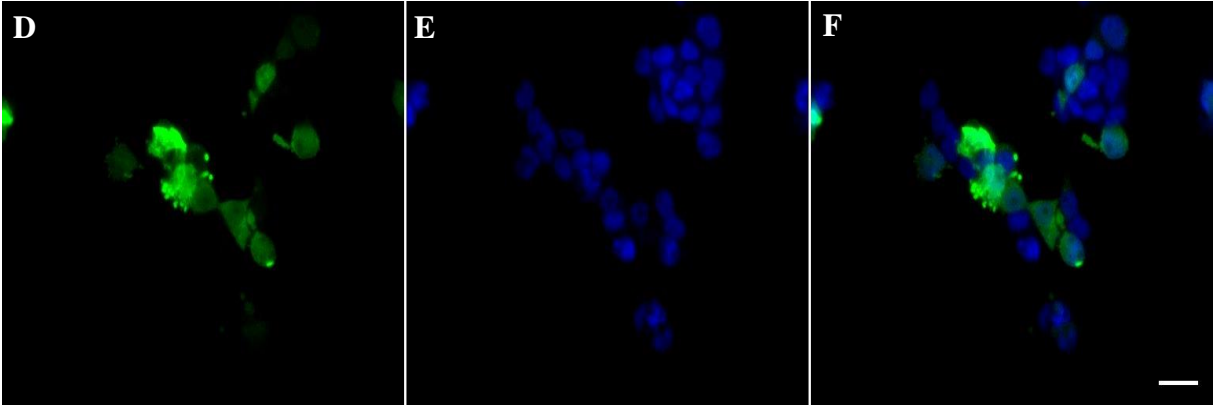

P2

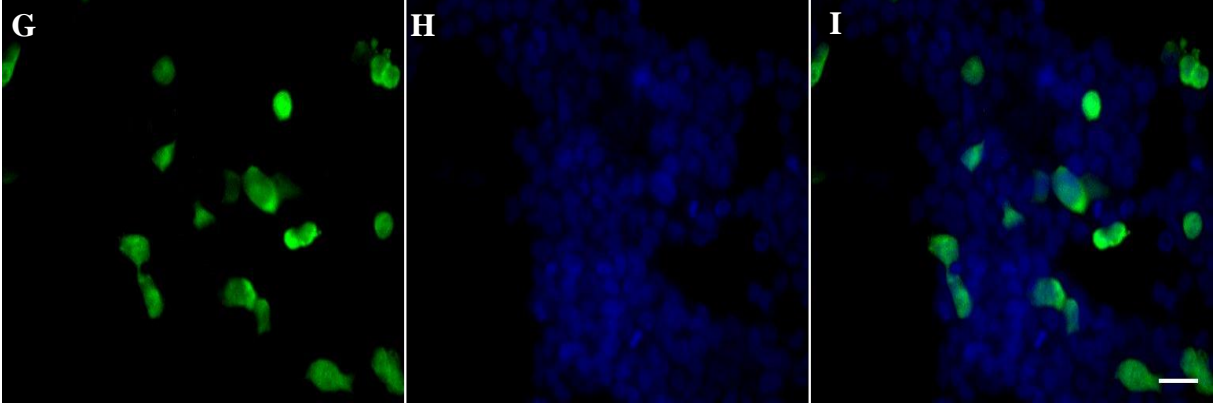

Supplement: Supplementary file 4 [file fvl-18-501-s4.pdf]

**A**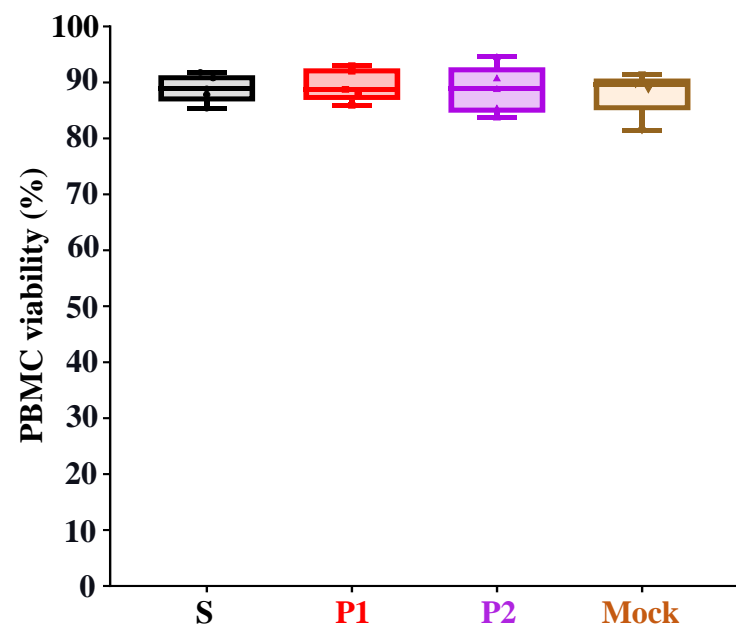**B**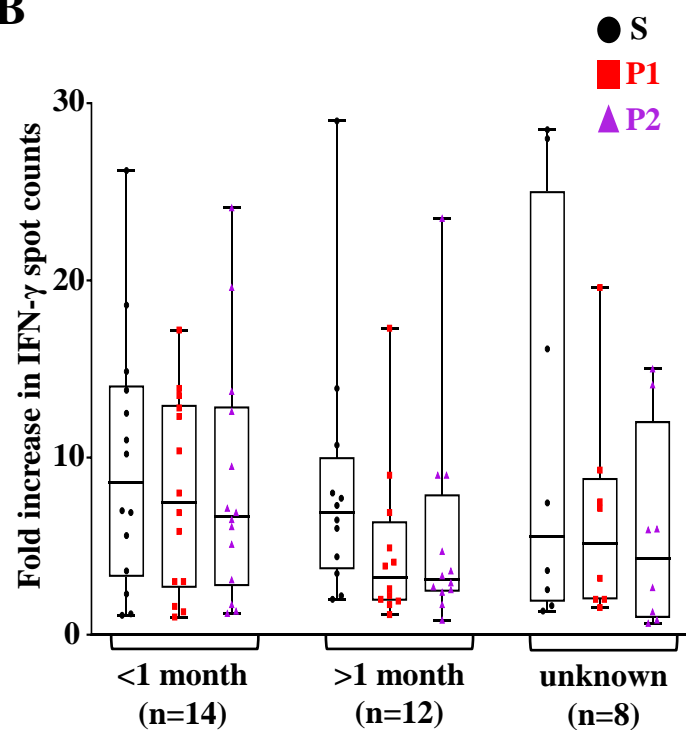**C**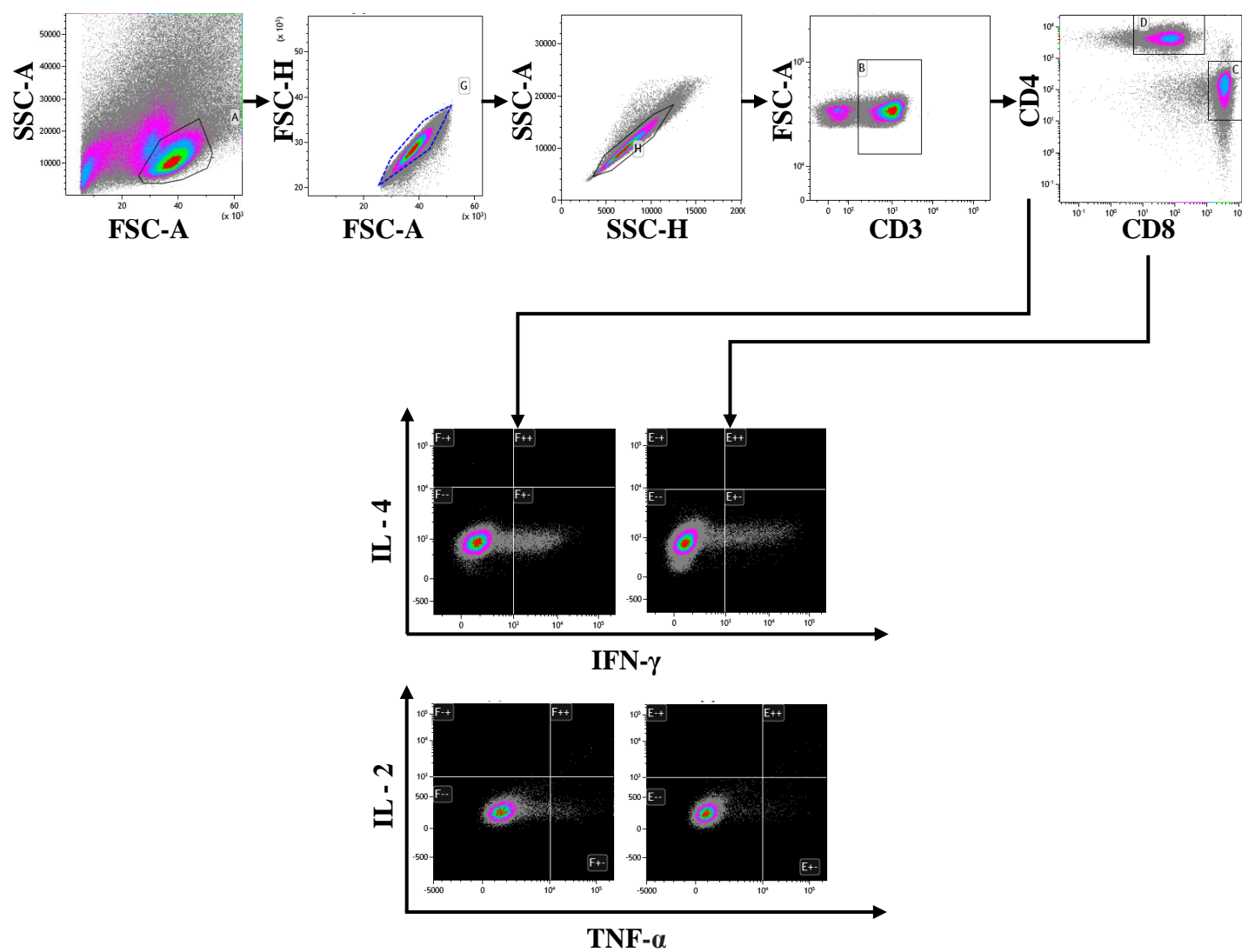

Supplement: Supplementary file 5 [file fvl-18-501-s5.pdf]

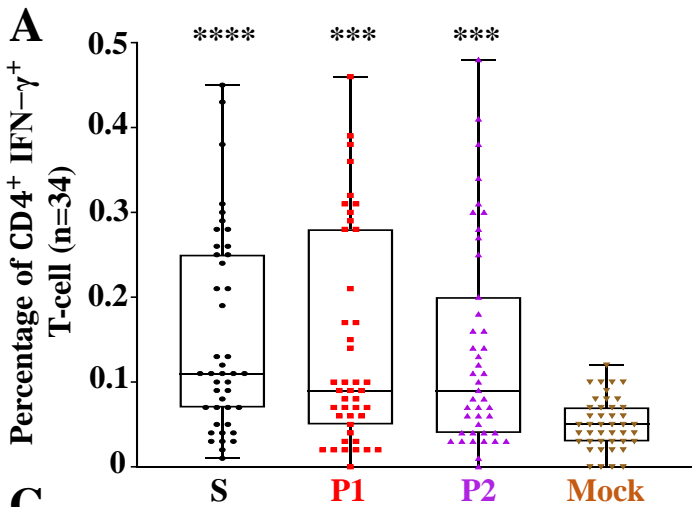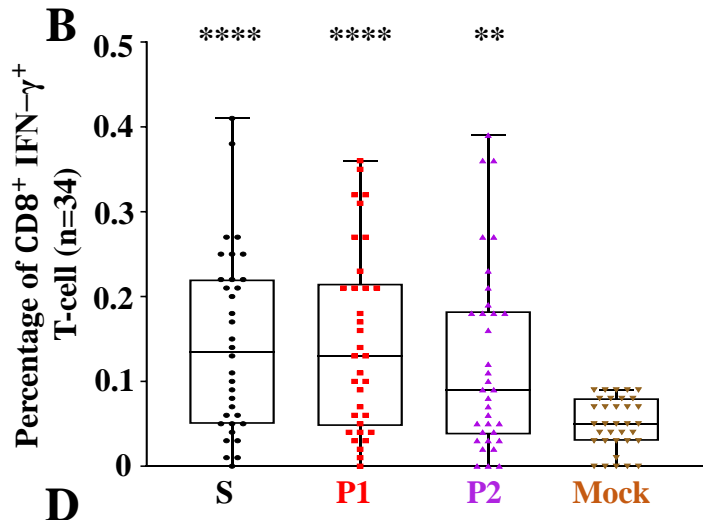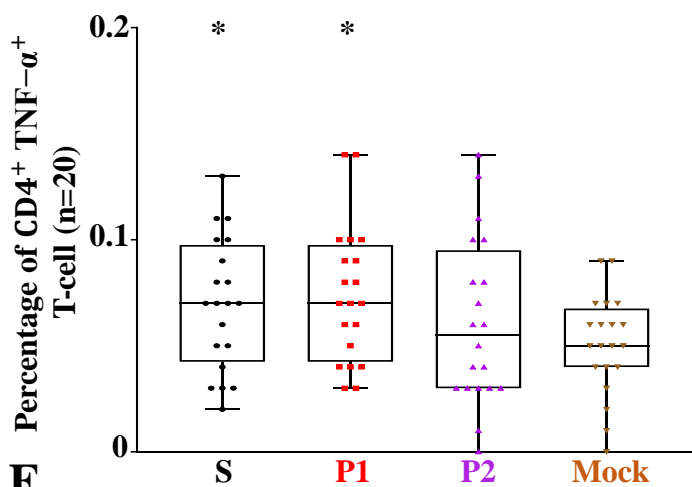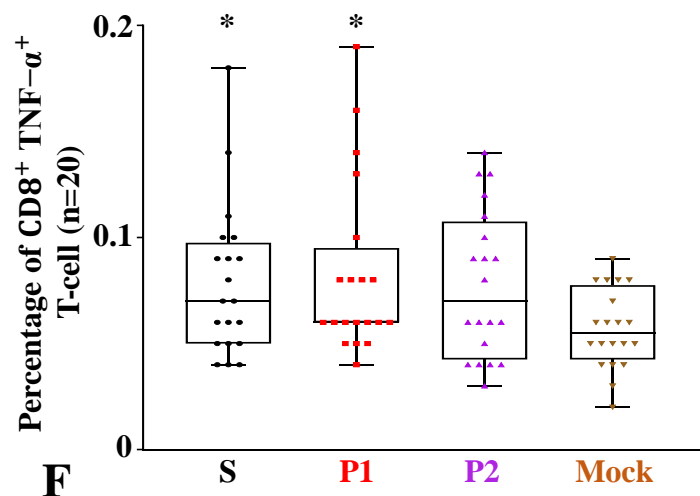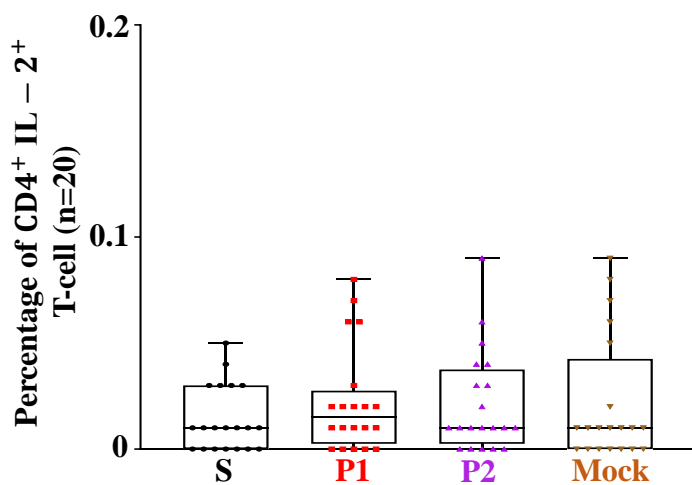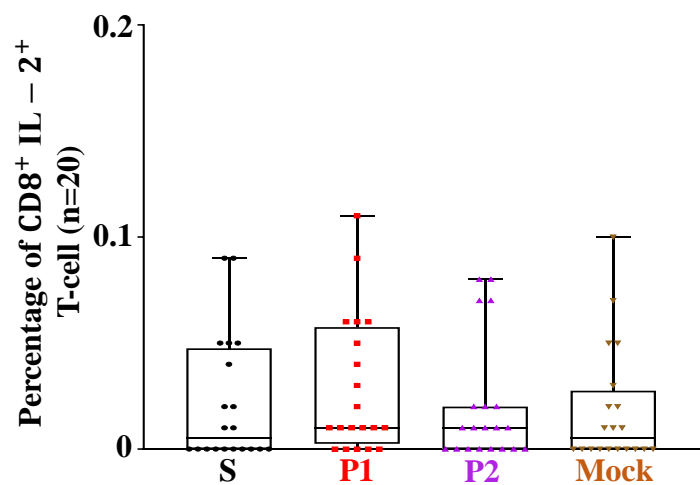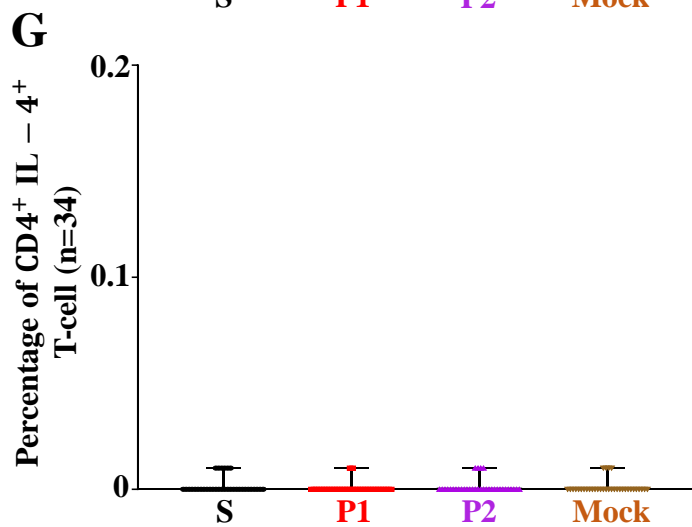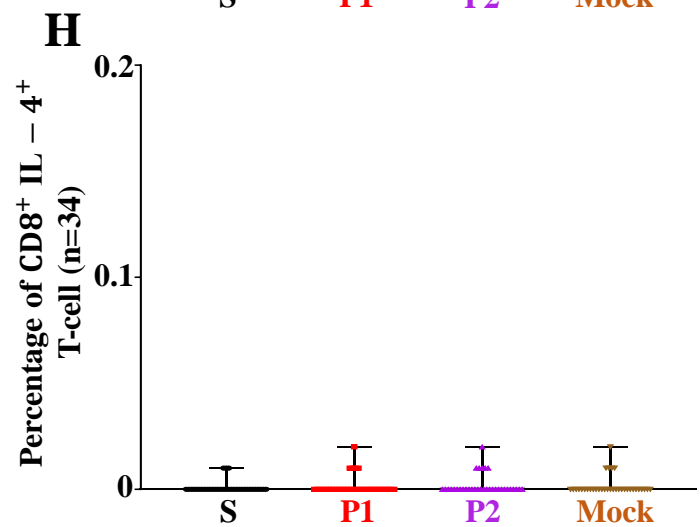

Supplement: Supplementary file 6 [file fvl-18-501-s6.pdf]
