# Supplementary material for: Design and generation of mRNAs encoding conserved regions of SARS-CoV-2 ORF1ab for T cell-mediated immune activation
Source: Future Virol. 2023 Jun 24;18(8):501–16. doi: 10.2217/fvl-2023-0066 (PMC10308627; doi:10.2217/fvl-2023-0066)
Supplement: Supplementary file 7 [file fvl-18-501-s7.docx]

| **Table S1: List of HLA I and II types retrieved from 4 different cohorts  in public databases** | | | | | |
| --- | --- | --- | --- | --- | --- |
| **HLA types** | **Class** | **Frequency   in Cohort 1** | **Frequency   in Cohort 2** | **Frequency   in Cohort 3** | **Frequency   in Cohort 4** |
| A*01:01 | I | 0 | 0.038 | 0 | 0.0149 |
| A*02:01 | I | 0 | 0.021 | 0 | 0.0297 |
| A*02:03 | I | 0 | 0.079 | 0 | 0.0793 |
| A*02:06 | I | 0 | 0.047 | 0 | 0.0297 |
| A*02:07 | I | 0 | 0.085 | 0 | 0.099 |
| A*03:02 | I | 0 | 0.003 | 0 | 0.0099 |
| A*11:01 | I | 0 | 0.229 | 0 | 0.2277 |
| A*11:02 | I | 0 | 0.035 | 0 | 0.0248 |
| A*11:04 | I | 0 | 0.003 | 0 | 0.0099 |
| A*24:02 | I | 0 | 0.138 | 0 | 0.1387 |
| A*24:03 | I | 0 | 0.015 | 0 | 0.0099 |
| A*24:07 | I | 0 | 0.044 | 0 | 0.0297 |
| A*24:10 | I | 0 | 0.024 | 0 | 0.005 |
| A*26:01 | I | 0 | 0.021 | 0 | 0.0198 |
| A*29:01 | I | 0 | 0.062 | 0 | 0.0842 |
| A*30:01 | I | 0 | 0.003 | 0 | 0.005 |
| A*31:01 | I | 0 | 0.021 | 0 | 0.0149 |
| A*33:03 | I | 0 | 0.115 | 0 | 0.1089 |
| A*34:01 | I | 0 | 0.006 | 0 | 0.0149 |
| A*68:01 | I | 0 | 0.009 | 0 | 0.005 |
| A*74:01 | I | 0 | 0.003 | 0 | 0 |
| B*07:05 | I | 0 | 0.074 | 0 | 0.0693 |
| B*08:01 | I | 0 | 0.003 | 0 | 0.005 |
| B*13:01 | I | 0 | 0.038 | 0 | 0.0297 |
| B*13:02 | I | 0 | 0.006 | 0 | 0.0099 |
| B*15:01 | I | 0 | 0.041 | 0 | 0.0099 |
| B*15:02 | I | 0 | 0.135 | 0 | 0.1188 |
| B*15:07 | I | 0 | 0.003 | 0 | 0 |
| B*15:17 | I | 0 | 0.003 | 0 | 0.005 |
| B*15:18 | I | 0 | 0.003 | 0 | 0 |
| B*15:21 | I | 0 | 0.003 | 0 | 0 |
| B*15:25 | I | 0 | 0.059 | 0 | 0.0545 |
| B*15:27 | I | 0 | 0.003 | 0 | 0.005 |
| B*18:01 | I | 0 | 0.006 | 0 | 0.0099 |
| B*18:02 | I | 0 | 0.018 | 0 | 0.005 |
| B*27:06 | I | 0 | 0.009 | 0 | 0.0198 |
| B*35:01 | I | 0 | 0.003 | 0 | 0.0198 |
| B*35:03 | I | 0 | 0.003 | 0 | 0.005 |
| B*35:05 | I | 0 | 0.041 | 0 | 0.0347 |
| B*38:02 | I | 0 | 0.056 | 0 | 0.0792 |
| B*39:01 | I | 0 | 0.012 | 0 | 0.0198 |
| B*39:05 | I | 0 | 0.003 | 0 | 0 |
| B*39:15 | I | 0 | 0.006 | 0 | 0 |
| B*40:01 | I | 0 | 0.062 | 0 | 0.0792 |
| B*40:02 | I | 0 | 0.012 | 0 | 0.005 |
| B*40:06 | I | 0 | 0.021 | 0 | 0.0198 |
| B*44:03 | I | 0 | 0.038 | 0 | 0.0099 |
| B*46:01 | I | 0 | 0.115 | 0 | 0.0941 |
| B*48:01 | I | 0 | 0.003 | 0 | 0.0149 |
| B*51:01 | I | 0 | 0.035 | 0 | 0.0198 |
| B*51:02 | I | 0 | 0.018 | 0 | 0.0149 |
| B*52:01 | I | 0 | 0.003 | 0 | 0.0198 |
| B*54:01 | I | 0 | 0.024 | 0 | 0.0149 |
| B*55:02 | I | 0 | 0.026 | 0 | 0.0198 |
| B*56:01 | I | 0 | 0.012 | 0 | 0.0149 |
| B*56:02 | I | 0 | 0.012 | 0 | 0 |
| B*57:01 | I | 0 | 0.029 | 0 | 0.005 |
| B*58:01 | I | 0 | 0.065 | 0 | 0.0842 |
| C*01:02 | I | 0 | 0.165 | 0 | 0.1337 |
| C*01:03 | I | 0 | 0.003 | 0 | 0 |
| C*03:02 | I | 0 | 0.068 | 0 | 0.0891 |
| C*03:03 | I | 0 | 0.05 | 0 | 0.0446 |
| C*03:04 | I | 0 | 0.062 | 0 | 0.0793 |
| C*04:01 | I | 0 | 0.053 | 0 | 0.0495 |
| C*04:03 | I | 0 | 0.059 | 0 | 0.0743 |
| C*04:06 | I | 0 | 0.006 | 0 | 0 |
| C*06:02 | I | 0 | 0.032 | 0 | 0.0198 |
| C*07:01 | I | 0 | 0.041 | 0 | 0.01 |
| C*07:02 | I | 0 | 0.147 | 0 | 0.2178 |
| C*07:04 | I | 0 | 0.024 | 0 | 0.0099 |
| C*08:01 | I | 0 | 0.156 | 0 | 0.1287 |
| C*12:02 | I | 0 | 0.021 | 0 | 0.0149 |
| C*12:03 | I | 0 | 0.009 | 0 | 0 |
| C*14:02 | I | 0 | 0.026 | 0 | 0.0198 |
| C*15:02 | I | 0 | 0.029 | 0 | 0.0149 |
| C*15:05 | I | 0 | 0.05 | 0 | 0.0693 |
| A*03:01 | I | 0 | 0 | 0 | 0.005 |
| A*24:20 | I | 0 | 0 | 0 | 0.0149 |
| A*32:01 | I | 0 | 0 | 0 | 0.005 |
| A*33:01 | I | 0 | 0 | 0 | 0.0099 |
| A*74:02 | I | 0 | 0 | 0 | 0.005 |
| B*07:02 | I | 0 | 0 | 0 | 0.0198 |
| B*15:11 | I | 0 | 0 | 0 | 0.005 |
| B*15:12 | I | 0 | 0 | 0 | 0.0149 |
| B*15:13 | I | 0 | 0 | 0 | 0.005 |
| B*15:35 | I | 0 | 0 | 0 | 0.0099 |
| B*37:01 | I | 0 | 0 | 0 | 0.005 |
| B*39:09 | I | 0 | 0 | 0 | 0.005 |
| B*51:06 | I | 0 | 0 | 0 | 0.005 |
| B*55:18 | I | 0 | 0 | 0 | 0.005 |
| B*56:04 | I | 0 | 0 | 0 | 0.0099 |
| C*03:17 | I | 0 | 0 | 0 | 0.005 |
| C*04:82 | I | 0 | 0 | 0 | 0.005 |
| C*07:06 | I | 0 | 0 | 0 | 0.005 |
| C*08:03 | I | 0 | 0 | 0 | 0.0099 |
| DQB1*02:01 | II | 0.065 | 0.091 | 0.11 | 0.0693 |
| DQB1*03:01 | II | 0.37 | 0.4 | 0.085 | 0.2871 |
| DQB1*03:02 | II | 0.045 | 0.024 | 0.024 | 0.0248 |
| DQB1*03:03 | II | 0.17 | 0.126 | 0.085 | 0.1337 |
| DQB1*04:01 | II | 0.05 | 0.035 | 0.024 | 0.0495 |
| DQB1*05:01 | II | 0.115 | 0.118 | 0.11 | 0.1238 |
| DQB1*05:02 | II | 0.08 | 0.065 | 0.481 | 0.1139 |
| DQB1*05:03 | II | 0.015 | 0.035 | 0.006 | 0.0348 |
| DQB1*06:01 | II | 0.035 | 0.065 | 0.018 | 0.0842 |
| DQB1*06:02 | II | 0.02 | 0.012 | 0.006 | 0.0099 |
| DQB1*06:03 | II | 0.01 | 0.006 | 0 | 0.005 |
| DQB1*06:05 | II | 0.005 | 0.015 | 0.006 | 0 |
| DQB1*06:09 | II | 0.02 | 0 | 0 | 0.0099 |
| DRB1*03:01 | II | 0.044 | 0.047 | 0.127 | 0.0743 |
| DRB1*04:03 | II | 0.025 | 0.021 | 0.018 | 0.0149 |
| DRB1*04:04 | II | 0.01 | 0.003 | 0 | 0 |
| DRB1*04:05 | II | 0.044 | 0.038 | 0.024 | 0.0644 |
| DRB1*04:08 | II | 0.005 | 0 | 0.006 | 0 |
| DRB1*07:01 | II | 0.049 | 0.076 | 0.006 | 0.0297 |
| DRB1*08:03 | II | 0.054 | 0.041 | 0 | 0.0545 |
| DRB1*09:01 | II | 0.142 | 0.097 | 0.078 | 0.1337 |
| DRB1*10:01 | II | 0.044 | 0.056 | 0.03 | 0.0792 |
| DRB1*11:01 | II | 0.005 | 0.015 | 0.012 | 0.0248 |
| DRB1*12:01 | II | 0.025 | 0.009 | 0 | 0 |
| DRB1*12:02 | II | 0.304 | 0.353 | 0.102 | 0.2228 |
| DRB1*13:01 | II | 0.02 | 0.006 | 0.006 | 0.005 |
| DRB1*13:02 | II | 0.02 | 0.018 | 0.006 | 0.0149 |
| DRB1*13:03 | II | 0.015 | 0.018 | 0 | 0 |
| DRB1*13:12 | II | 0.005 | 0 | 0.012 | 0.0297 |
| DRB1*14:01 | II | 0.029 | 0.032 | 0.151 | 0 |
| DRB1*14:04 | II | 0.01 | 0.018 | 0 | 0.005 |
| DRB1*14:05 | II | 0.005 | 0.012 | 0 | 0.0099 |
| DRB1*15:01 | II | 0.039 | 0.038 | 0.054 | 0.0248 |
| DRB1*15:02 | II | 0.078 | 0.062 | 0.127 | 0.104 |
| DRB1*15:05 | II | 0.005 | 0 | 0 | 0 |
| DRB1*16:02 | II | 0.01 | 0.018 | 0.199 | 0.0446 |
| DRB1*16:05 | II | 0.015 | 0 | 0 | 0 |
| DQB1*04:02 | II | 0 | 0.006 | 0 | 0.0099 |
| DQB1*06:04 | II | 0 | 0.003 | 0.012 | 0.005 |
| DRB1*04:01 | II | 0 | 0.003 | 0 | 0.005 |
| DRB1*04:06 | II | 0 | 0.006 | 0 | 0.0099 |
| DRB1*08:02 | II | 0 | 0.009 | 0 | 0 |
| DRB1*14:02 | II | 0 | 0.003 | 0 | 0 |
| DRB1*14:03 | II | 0 | 0.003 | 0 | 0 |
| DQB1*03:09 | II | 0 | 0 | 0.018 | 0 |
| DQB1*05:04 | II | 0 | 0 | 0.006 | 0 |
| DQB1*06:06 | II | 0 | 0 | 0.006 | 0 |
| DRB1*08:18 | II | 0 | 0 | 0.03 | 0 |
| DRB1*15:06 | II | 0 | 0 | 0.012 | 0 |
| DQB1*02:02 | II | 0 | 0 | 0 | 0.0198 |
| DQB1*03:05 | II | 0 | 0 | 0 | 0.005 |
| DQB1*05:10 | II | 0 | 0 | 0 | 0.005 |
| DQB1*05:18 | II | 0 | 0 | 0 | 0.0099 |
| DRB1*08:12 | II | 0 | 0 | 0 | 0.005 |
| DRB1*11:06 | II | 0 | 0 | 0 | 0.0149 |
| DRB1*11:12 | II | 0 | 0 | 0 | 0.005 |
| DRB1*14:10 | II | 0 | 0 | 0 | 0.005 |
| DRB1*14:18 | II | 0 | 0 | 0 | 0.005 |
| DRB1*14:54 | II | 0 | 0 | 0 | 0.0149 |
