# Supplementary material for: Design and generation of mRNAs encoding conserved regions of SARS-CoV-2 ORF1ab for T cell-mediated immune activation
Source: Future Virol. 2023 Jun 24;18(8):501–16. doi: 10.2217/fvl-2023-0066 (PMC10308627; doi:10.2217/fvl-2023-0066)
Supplement: Supplementary file 8 [file fvl-18-501-s8.docx]

| **Table S2: List of epitopes within the ORF1ab predicted to bind to HLA-I and HLA-II commonly found  among Vietnamese populations** | | | | | | | |
| --- | --- | --- | --- | --- | --- | --- | --- |
| **Position (aa)** | | **Peptide** | **IC50** | **Vaxijen** | **Class 1 immunogenicity** | **Strong binder** | **HLA class** |
| 84 | 92 | VMVELVAEL | 10.56 | 0.4101 | 0.23373 | Yes | Class I |
| 96 | 104 | QYGRSGETL | 36.1 | 0.8825 | 0.04106 | Yes | Class I |
| 103 | 111 | TLGVLVPHV | 31.99 | 0.5583 | 0.09014 | Yes | Class I |
| 214 | 222 | TLSEQLDFI | 34.09 | 1.0499 | 0.01093 | Yes | Class I |
| 296 | 304 | FMGRIRSVY | 29.96 | 0.5212 | 0.1259 | Yes | Class I |
| 376 | 384 | SEVGPEHSL | 19.92 | 0.7216 | 0.06159 | Yes | Class I |
| 468 | 476 | KLNEEIAII | 34.66 | 0.6394 | 0.43221 | Yes | Class I |
| 472 | 480 | EIAIILASF | 39.6 | 0.648 | 0.20214 | Yes | Class I |
| 722 | 730 | KSREETGLL | 41.33 | 0.6263 | 0.27371 | Yes | Class I |
| 798 | 806 | KYCALAPNM | 37.41 | 0.959 | 0.03476 | Yes | Class I |
| 808 | 816 | VTNNTFTLK | 22.63 | 0.7146 | 0.16567 | Yes | Class I |
| 823 | 831 | VTFGDDTVI | 42.41 | 0.5256 | 0.17146 | Yes | Class I |
| 1051 | 1059 | KVKPTVVVN | 48.53 | 1.156 | 0.05446 | Yes | Class I |
| 1367 | 1375 | ILGTVSWNL | 16.02 | 1.3875 | 0.11769 | Yes | Class I |
| 1387 | 1395 | KLMPVCVET | 27.59 | 1.42 | 0.01463 | Yes | Class I |
| 1437 | 1445 | TLNDLNETL | 34.61 | 0.7526 | 0.11051 | Yes | Class I |
| 1560 | 1568 | LSLREVRTI | 40.57 | 1.5576 | 0.2512 | Yes | Class I |
| 1634 | 1642 | YYHTTDPSF | 34.58 | 1.2164 | 0.00222 | Yes | Class I |
| 1709 | 1717 | NFCALILAY | 17.23 | 0.6336 | 0.14985 | Yes | Class I |
| 2002 | 2010 | TYKPNTWCI | 24.18 | 1.694 | 0.10452 | Yes | Class I |
| 2167 | 2175 | NYMPYFFTL | 4.41 | 1.0015 | 0.15992 | Yes | Class I |
| 2168 | 2176 | YMPYFFTLL | 10.78 | 0.6882 | 0.24316 | Yes | Class I |
| 2230 | 2238 | IIWFLLLSV | 42.84 | 0.7365 | 0.06244 | Yes | Class I |
| 2245 | 2253 | YSTAALGVL | 27.44 | 0.6702 | 0.13235 | Yes | Class I |
| 2363 | 2371 | WLMWLIINL | 9.18 | 1.0138 | 0.38891 | Yes | Class I |
| 2364 | 2372 | LMWLIINLV | 48.1 | 0.9675 | 0.30368 | Yes | Class I |
| 2511 | 2519 | KTYERHSLS | 15.2 | 0.5582 | 0.0343 | Yes | Class I |
| 2684 | 2692 | TPRDLGACI | 30.6 | 1.3016 | 0.06174 | Yes | Class I |
| 2705 | 2713 | KSHNIALIW | 32.84 | 1.2831 | 0.23882 | Yes | Class I |
| 2780 | 2788 | FLFVAAIFY | 11.35 | 0.4421 | 0.33519 | Yes | Class I |
| 2889 | 2897 | FSAVGNICY | 21.8 | 1.1852 | 0.16197 | Yes | Class I |
| 2901 | 2909 | KLIEYTDFA | 6.45 | 1.2764 | 0.26401 | Yes | Class I |
| 3016 | 3024 | GVFCGVDAV | 44.69 | 0.5684 | 0.09719 | Yes | Class I |
| 3086 | 3094 | LMSFTVLCL | 30.76 | 1.5912 | 0.0999 | Yes | Class I |
| 3108 | 3116 | IYLYLTFYL | 33.27 | 0.7247 | 0.11506 | Yes | Class I |
| 3109 | 3117 | YLYLTFYLT | 33.79 | 1.1437 | 0.12604 | Yes | Class I |
| 3122 | 3130 | FLAHIQWMV | 2.01 | 0.8064 | 0.15015 | Yes | Class I |
| 3152 | 3160 | HFYWFFSNY | 8.38 | 0.6725 | 0.30249 | Yes | Class I |
| 3286 | 3294 | GTTTLNGLW | 22.73 | 0.4718 | 0.05689 | Yes | Class I |
| 3392 | 3400 | AMRPNFTIK | 26.61 | 1.7141 | 0.22006 | Yes | Class I |
| 3553 | 3561 | EFTPFDVVR | 29.19 | 1.6049 | 0.19528 | Yes | Class I |
| 3596 | 3604 | QSTQWSLFF | 4.4 | 0.9087 | 0.01505 | Yes | Class I |
| 3598 | 3606 | TQWSLFFFL | 17.16 | 0.8119 | 0.17203 | Yes | Class I |
| 3599 | 3607 | QWSLFFFLY | 14 | 0.6731 | 0.25166 | Yes | Class I |
| 3606 | 3614 | LYENAFLPF | 42.26 | 0.4274 | 0.15845 | Yes | Class I |
| 3654 | 3662 | YMPASWVMR | 40.98 | 0.952 | 0.01542 | Yes | Class I |
| 3753 | 3761 | FLARGIVFM | 6.13 | 0.7022 | 0.3263 | Yes | Class I |
| 3760 | 3768 | FMCVEYCPI | 44.81 | 1.3582 | 0.06608 | Yes | Class I |
| 3807 | 3815 | TLGVYDYLV | 20.8 | 0.7171 | 0.06022 | Yes | Class I |
| 3815 | 3823 | VSTQEFRYM | 32.39 | 0.8979 | 0.14526 | Yes | Class I |
| 4094 | 4102 | ALWEIQQVV | 5.73 | 0.5893 | 0.11957 | Yes | Class I |
| 4168 | 4176 | ALAYYNTTK | 24.7 | 0.4618 | 0.05473 | Yes | Class I |
| 4308 | 4316 | ITVTPEANM | 35.74 | 1.1121 | 0.16515 | Yes | Class I |
| 4456 | 4464 | NLIDSYFVV | 4.85 | 0.4282 | 0.02386 | Yes | Class I |
| 4514 | 4522 | YTMADLVYA | 6.42 | 1.0142 | 0.02621 | Yes | Class I |
| 4515 | 4523 | TMADLVYAL | 4.08 | 0.8208 | 0.08282 | Yes | Class I |
| 4632 | 4640 | LLMPILTLT | 16.52 | 0.5803 | 0.07728 | Yes | Class I |
| 4699 | 4707 | ILHCANFNV | 7.37 | 0.5385 | 0.08328 | Yes | Class I |
| 4713 | 4721 | FPPTSFGPL | 42.01 | 1.6296 | 0.00668 | Yes | Class I |
| 4716 | 4724 | TSFGPLVRK | 41.89 | 1.7142 | 0.11594 | Yes | Class I |
| 4839 | 4847 | NAAISDYDY | 33.93 | 1.2918 | 0.01624 | Yes | Class I |
| 4842 | 4850 | ISDYDYYRY | 8.52 | 0.4552 | 0.04872 | Yes | Class I |
| 4859 | 4867 | RQLLFVVEV | 17.02 | 0.816 | 0.23144 | Yes | Class I |
| 4863 | 4871 | FVVEVVDKY | 18.8 | 0.6617 | 0.08593 | Yes | Class I |
| 4892 | 4900 | KSAGFPFNK | 22.93 | 1.1874 | 0.24538 | Yes | Class I |
| 4917 | 4925 | DALFAYTKR | 44.99 | 1.2074 | 0.05558 | Yes | Class I |
| 4969 | 4977 | KSIAATRGA | 29.29 | 0.8779 | 0.22069 | Yes | Class I |
| 4973 | 4981 | ATRGATVVI | 12.67 | 0.5688 | 0.1845 | Yes | Class I |
| 5003 | 5011 | NPHLMGWDY | 49.79 | 0.9515 | 0.06014 | Yes | Class I |
| 5046 | 5054 | RLANECAQV | 19.62 | 0.9246 | 0.01828 | Yes | Class I |
| 5253 | 5261 | SLAIDAYPL | 30.08 | 0.7576 | 0.19545 | Yes | Class I |
| 5274 | 5282 | HLYLQYIRK | 13.66 | 0.492 | 0.01392 | Yes | Class I |
| 5304 | 5312 | TSRYWEPEF | 16.58 | 0.5982 | 0.37217 | Yes | Class I |
| 5380 | 5388 | DVTDVTQLY | 26.69 | 0.9181 | 0.00742 | Yes | Class I |
| 5470 | 5478 | KLSYGIATV | 8.63 | 1.0767 | 0.15656 | Yes | Class I |
| 5528 | 5536 | DYGDAVVYR | 20.61 | 0.5459 | 0.14296 | Yes | Class I |
| 5534 | 5542 | VYRGTTTYK | 15.28 | 0.6644 | 0.15584 | Yes | Class I |
| 5542 | 5550 | KLNVGDYFV | 7.35 | 0.4501 | 0.1586 | Yes | Class I |
| 5563 | 5571 | TLVPQEHYV | 19.5 | 0.5402 | 0.00883 | Yes | Class I |
| 5612 | 5620 | KSHFAIGLA | 14.35 | 1.526 | 0.3138 | Yes | Class I |
| 5614 | 5622 | HFAIGLALY | 10.18 | 1.4046 | 0.19572 | Yes | Class I |
| 5661 | 5669 | RARVECFDK | 45 | 1.7869 | 0.21685 | Yes | Class I |
| 5679 | 5687 | YVFCTVNAL | 32.02 | 0.5377 | 0.07781 | Yes | Class I |
| 6032 | 6040 | LQLGFSTGV | 15.73 | 1.1419 | 0.04133 | Yes | Class I |
| 6042 | 6050 | LVAVPTGYV | 27.86 | 0.496 | 0.10642 | Yes | Class I |
| 6080 | 6088 | KGLPWNVVR | 24.96 | 0.6163 | 0.25381 | Yes | Class I |
| 6101 | 6109 | NLSDRVVFV | 10.8 | 0.9492 | 0.16112 | Yes | Class I |
| 6148 | 6156 | TYACWHHSI | 9.43 | 0.9009 | 0.13524 | Yes | Class I |
| 6453 | 6461 | SLENVAFNV | 26.04 | 1.0488 | 0.19804 | Yes | Class I |
| 6505 | 6513 | VAFELWAKR | 43.31 | 0.7406 | 0.24348 | Yes | Class I |
| 6695 | 6703 | SQLGGLHLL | 27.34 | 0.7799 | 0.07388 | Yes | Class I |
| 6886 | 6894 | WLPTGTLLV | 26.47 | 0.4535 | 0.08916 | Yes | Class I |
| 6970 | 6978 | TEHSWNADL | 42.23 | 0.5939 | 0.09962 | Yes | Class I |
| 7027 | 7035 | IFWRNTNPI | 47.1 | 1.1927 | 0.14228 | Yes | Class I |
| 7073 | 7081 | LSKGRLIIR | 44.45 | 0.6165 | 0.19414 | Yes | Class I |
| 7077 | 7085 | RLIIRENNR | 23.36 | 0.6013 | 0.31253 | Yes | Class I |
| 3 | 11 | SLVPGFNEK | 88.35 | 1.4706 | 0.19848 | No | Class I |
| 10 | 18 | EKTHVQLSL | 482.92 | 1.3539 | -0.12971 | No | Class I |
| 11 | 19 | KTHVQLSLP | 154.51 | 0.6061 | -0.2173 | No | Class I |
| 12 | 20 | THVQLSLPV | 347.98 | 0.6614 | -0.28553 | No | Class I |
| 13 | 21 | HVQLSLPVL | 494.81 | 0.622 | -0.20554 | No | Class I |
| 15 | 23 | QLSLPVLQV | 280.42 | 0.4648 | -0.11384 | No | Class I |
| 16 | 24 | LSLPVLQVR | 122.09 | 1.3892 | -0.05864 | No | Class I |
| 18 | 26 | LPVLQVRDV | 271 | 1.9536 | -0.01506 | No | Class I |
| 20 | 28 | VLQVRDVLV | 335.91 | 1.2075 | 0.10358 | No | Class I |
| 27 | 35 | LVRGFGDSV | 225.22 | -1.2177 | 0.11886 | No | Class I |
| 31 | 39 | FGDSVEEVL | 435.93 | -0.1079 | 0.0838 | No | Class I |
| 34 | 42 | SVEEVLSEA | 310.3 | -0.0405 | 0.08189 | No | Class I |
| 38 | 46 | VLSEARQHL | 337.62 | 0.4301 | 0.05501 | No | Class I |
| 39 | 47 | LSEARQHLK | 223.48 | 0.6432 | 0.03405 | No | Class I |
| 45 | 53 | HLKDGTCGL | 26.82 | 1.6536 | -0.00384 | No | Class I |
| 52 | 60 | GLVEVEKGV | 130.61 | 1.0813 | 0.0864 | No | Class I |
| 56 | 64 | VEKGVLPQL | 180.83 | 0.1106 | -0.08318 | No | Class I |
| 60 | 68 | VLPQLEQPY | 445.97 | -0.0327 | -0.14095 | No | Class I |
| 61 | 69 | LPQLEQPYV | 345.21 | -0.2419 | -0.07182 | No | Class I |
| 65 | 73 | EQPYVFIKR | 306.5 | 0.4801 | 0.1294 | No | Class I |
| 68 | 76 | YVFIKRSDA | 370 | 0.5528 | -0.11602 | No | Class I |
| 69 | 77 | VFIKRSDAR | 97.12 | 0.2295 | -0.23755 | No | Class I |
| 77 | 85 | RTAPHGHVM | 61.33 | 0.2594 | 0.11636 | No | Class I |
| 78 | 86 | TAPHGHVMV | 115.75 | -0.3432 | 0.02464 | No | Class I |
| 79 | 87 | APHGHVMVE | 484.64 | -0.0212 | -0.00912 | No | Class I |
| 87 | 95 | ELVAELEGI | 237.52 | 0.191 | 0.24413 | No | Class I |
| 89 | 97 | VAELEGIQY | 337.26 | 0.3183 | 0.19538 | No | Class I |
| 90 | 98 | AELEGIQYG | 405.49 | 0.7438 | 0.15551 | No | Class I |
| 91 | 99 | ELEGIQYGR | 180.75 | 1.1661 | 0.10384 | No | Class I |
| 99 | 107 | RSGETLGVL | 73.56 | 0.494 | 0.19183 | No | Class I |
| 102 | 110 | ETLGVLVPH | 496.21 | 0.4183 | 0.08862 | No | Class I |
| 106 | 114 | VLVPHVGEI | 207.58 | 0.2475 | 0.1597 | No | Class I |
| 108 | 116 | VPHVGEIPV | 168.64 | 0.3041 | 0.28513 | No | Class I |
| 110 | 118 | HVGEIPVAY | 67.47 | 0.6413 | 0.28861 | No | Class I |
| 111 | 119 | VGEIPVAYR | 297.18 | 0.8024 | 0.22534 | No | Class I |
| 114 | 122 | IPVAYRKVL | 11.16 | 0.7702 | -0.05999 | No | Class I |
| 116 | 124 | VAYRKVLLR | 201.97 | -0.456 | -0.1361 | No | Class I |
| 117 | 125 | AYRKVLLRK | 15.22 | -1.2982 | -0.14956 | No | Class I |
| 121 | 129 | VLLRKNGNK | 61.05 | -0.7264 | -0.14279 | No | Class I |
| 135 | 143 | SYGADLKSF | 84.23 | 0.2404 | -0.21713 | No | Class I |
| 141 | 149 | KSFDLGDEL | 52.08 | 1.7648 | 0.15864 | No | Class I |
| 165 | 173 | HSSGVTREL | 128.79 | -0.0488 | 0.15932 | No | Class I |
| 169 | 177 | VTRELMREL | 196.88 | -0.8692 | 0.04363 | No | Class I |
| 173 | 181 | LMRELNGGA | 112.81 | 0.1212 | 0.14906 | No | Class I |
| 174 | 182 | MRELNGGAY | 116.21 | 0.4424 | 0.0984 | No | Class I |
| 175 | 183 | RELNGGAYT | 297.15 | 0.4595 | 0.08565 | No | Class I |
| 176 | 184 | ELNGGAYTR | 91.31 | 0.7876 | 0.12139 | No | Class I |
| 182 | 190 | YTRYVDNNF | 113.42 | 0.4969 | 0.06492 | No | Class I |
| 196 | 204 | YPLECIKDL | 365.34 | 0.6396 | 0.00089 | No | Class I |
| 199 | 207 | ECIKDLLAR | 412.76 | -1.3279 | -0.14914 | No | Class I |
| 200 | 208 | CIKDLLARA | 53.86 | -0.8305 | -0.00566 | No | Class I |
| 207 | 215 | RAGKASCTL | 348.8 | 0.8727 | -0.34645 | No | Class I |
| 213 | 221 | CTLSEQLDF | 32.9 | 1.2746 | -0.17801 | No | Class I |
| 221 | 229 | FIDTKRGVY | 137.44 | 1.4512 | -0.0623 | No | Class I |
| 232 | 240 | REHEHEIAW | 311.92 | 0.9263 | 0.37218 | No | Class I |
| 234 | 242 | HEHEIAWYT | 337.8 | 0.4819 | 0.46246 | No | Class I |
| 236 | 244 | HEIAWYTER | 488.34 | 0.5937 | 0.38605 | No | Class I |
| 241 | 249 | YTERSEKSY | 77.96 | 0.6382 | -0.26093 | No | Class I |
| 243 | 251 | ERSEKSYEL | 46.89 | 0.8976 | -0.2633 | No | Class I |
| 247 | 255 | KSYELQTPF | 327.74 | 0.1923 | 0.00599 | No | Class I |
| 249 | 257 | YELQTPFEI | 483.61 | 0.5468 | 0.0645 | No | Class I |
| 250 | 258 | ELQTPFEIK | 250.47 | 0.9917 | 0.26312 | No | Class I |
| 251 | 259 | LQTPFEIKL | 163.4 | 0.8761 | 0.19601 | No | Class I |
| 253 | 261 | TPFEIKLAK | 426.85 | 0.9483 | 0.07885 | No | Class I |
| 255 | 263 | FEIKLAKKF | 48.33 | 1.0141 | -0.45577 | No | Class I |
| 258 | 266 | KLAKKFDTF | 237.23 | -0.1836 | -0.2627 | No | Class I |
| 265 | 273 | TFNGECPNF | 55.68 | 0.8622 | 0.06561 | No | Class I |
| 272 | 280 | NFVFPLNSI | 288.16 | 0.5874 | 0.00784 | No | Class I |
| 273 | 281 | FVFPLNSII | 257.9 | 0.0012 | -0.05191 | No | Class I |
| 275 | 283 | FPLNSIIKT | 27.17 | -0.0573 | -0.05961 | No | Class I |
| 279 | 287 | SIIKTIQPR | 102.02 | -0.0592 | -0.11496 | No | Class I |
| 280 | 288 | IIKTIQPRV | 276.46 | -0.2958 | 0.0105 | No | Class I |
| 282 | 290 | KTIQPRVEK | 39.76 | -0.4547 | 0.0579 | No | Class I |
| 283 | 291 | TIQPRVEKK | 366.14 | 1.1088 | -0.001 | No | Class I |
| 285 | 293 | QPRVEKKKL | 301.66 | -0.2826 | -0.35516 | No | Class I |
| 292 | 300 | KLDGFMGRI | 127.14 | 0.1234 | 0.04884 | No | Class I |
| 295 | 303 | GFMGRIRSV | 450.79 | 0.9706 | 0.0998 | No | Class I |
| 297 | 305 | MGRIRSVYP | 141.28 | 0.5534 | 0.07807 | No | Class I |
| 298 | 306 | GRIRSVYPV | 75.18 | 0.7972 | -0.03656 | No | Class I |
| 299 | 307 | RIRSVYPVA | 3.34 | 1.064 | -0.09819 | No | Class I |
| 304 | 312 | YPVASPNEC | 262.68 | -0.2378 | -0.06573 | No | Class I |
| 313 | 321 | NQMCLSTLM | 42.99 | 0.3429 | -0.2515 | No | Class I |
| 314 | 322 | QMCLSTLMK | 50 | -0.0324 | -0.26518 | No | Class I |
| 328 | 336 | ETSWQTGDF | 200.86 | 1.314 | 0.13449 | No | Class I |
| 331 | 339 | WQTGDFVKA | 108.8 | 0.5165 | 0.08734 | No | Class I |
| 335 | 343 | DFVKATCEF | 475.22 | 0.4408 | -0.11596 | No | Class I |
| 341 | 349 | CEFCGTENL | 494.8 | 0.3761 | 0.13401 | No | Class I |
| 351 | 359 | KEGATTCGY | 315.91 | 0.3206 | 0.09901 | No | Class I |
| 358 | 366 | GYLPQNAVV | 161.28 | 0.1549 | -0.07651 | No | Class I |
| 360 | 368 | LPQNAVVKI | 210.71 | -0.5381 | -0.05831 | No | Class I |
| 364 | 372 | AVVKIYCPA | 94.96 | 0.2369 | -0.12946 | No | Class I |
| 370 | 378 | CPACHNSEV | 125.89 | 0.41 | -0.09726 | No | Class I |
| 379 | 387 | GPEHSLAEY | 420.54 | -0.4007 | -0.01497 | No | Class I |
| 383 | 391 | SLAEYHNES | 104.82 | 0.7202 | 0.19334 | No | Class I |
| 385 | 393 | AEYHNESGL | 30.9 | 0.8911 | -0.00052 | No | Class I |
| 389 | 397 | NESGLKTIL | 293.32 | -0.6775 | -0.12288 | No | Class I |
| 390 | 398 | ESGLKTILR | 280.37 | -0.5503 | -0.06778 | No | Class I |
| 391 | 399 | SGLKTILRK | 195.11 | -0.9554 | -0.03664 | No | Class I |
| 394 | 402 | KTILRKGGR | 18.13 | -0.4757 | -0.07216 | No | Class I |
| 402 | 410 | RTIAFGGCV | 235.46 | 0.2424 | 0.22557 | No | Class I |
| 403 | 411 | TIAFGGCVF | 40.53 | -0.4015 | 0.17402 | No | Class I |
| 405 | 413 | AFGGCVFSY | 21.67 | -0.545 | 0.0336 | No | Class I |
| 406 | 414 | FGGCVFSYV | 321.72 | -0.6438 | -0.03463 | No | Class I |
| 411 | 419 | FSYVGCHNK | 206.1 | 1.0326 | 0.04611 | No | Class I |
| 418 | 426 | NKCAYWVPR | 436.23 | 0.5998 | 0.25514 | No | Class I |
| 420 | 428 | CAYWVPRAS | 384.15 | 0.5748 | 0.31799 | No | Class I |
| 421 | 429 | AYWVPRASA | 363.95 | 0.6133 | 0.08772 | No | Class I |
| 423 | 431 | WVPRASANI | 209.54 | 0.4549 | -0.03991 | No | Class I |
| 424 | 432 | VPRASANIG | 315.19 | 0.494 | 0.0042 | No | Class I |
| 443 | 451 | SEGLNDNLL | 129.74 | -0.1667 | 0.00248 | No | Class I |
| 445 | 453 | GLNDNLLEI | 15.5 | 0.3305 | 0.05262 | No | Class I |
| 451 | 459 | LEILQKEKV | 278.79 | 1.0205 | -0.32526 | No | Class I |
| 456 | 464 | KEKVNINIV | 172.72 | 1.1172 | 0.16282 | No | Class I |
| 460 | 468 | NINIVGDFK | 192.95 | 0.9633 | 0.29104 | No | Class I |
| 465 | 473 | GDFKLNEEI | 490.29 | 0.8016 | -0.05289 | No | Class I |
| 467 | 475 | FKLNEEIAI | 486.22 | 0.6674 | 0.31682 | No | Class I |
| 471 | 479 | EEIAIILAS | 201.13 | 0.6551 | 0.35095 | No | Class I |
| 474 | 482 | AIILASFSA | 426.69 | 0.4328 | -0.08345 | No | Class I |
| 476 | 484 | ILASFSAST | 110.65 | 0.1878 | -0.25914 | No | Class I |
| 478 | 486 | ASFSASTSA | 94.86 | 0.5585 | -0.31 | No | Class I |
| 479 | 487 | SFSASTSAF | 351.29 | 0.2742 | -0.25565 | No | Class I |
| 480 | 488 | FSASTSAFV | 56.04 | 0.0271 | -0.17028 | No | Class I |
| 483 | 491 | STSAFVETV | 179.9 | 0.0844 | 0.24571 | No | Class I |
| 484 | 492 | TSAFVETVK | 319.9 | 0.2433 | 0.32183 | No | Class I |
| 486 | 494 | AFVETVKGL | 82.84 | 0.4232 | 0.02861 | No | Class I |
| 489 | 497 | ETVKGLDYK | 144.62 | 1.2897 | -0.16448 | No | Class I |
| 492 | 500 | KGLDYKAFK | 421.66 | 0.5004 | -0.08646 | No | Class I |
| 494 | 502 | LDYKAFKQI | 395.1 | 0.5681 | -0.31958 | No | Class I |
| 501 | 509 | QIVESCGNF | 219.51 | -0.3186 | -0.07288 | No | Class I |
| 502 | 510 | IVESCGNFK | 210.76 | -0.2759 | -0.09163 | No | Class I |
| 503 | 511 | VESCGNFKV | 267.4 | -0.3508 | -0.10824 | No | Class I |
| 510 | 518 | KVTKGKAKK | 273.09 | -0.3952 | -0.46738 | No | Class I |
| 515 | 523 | KAKKGAWNI | 52.1 | 1.2931 | -0.03401 | No | Class I |
| 519 | 527 | GAWNIGEQK | 135.63 | 0.7237 | 0.24371 | No | Class I |
| 524 | 532 | GEQKSILSP | 437.24 | 0.259 | -0.39644 | No | Class I |
| 525 | 533 | EQKSILSPL | 451.85 | 0.5503 | -0.26341 | No | Class I |
| 527 | 535 | KSILSPLYA | 48.81 | 0.0288 | -0.15102 | No | Class I |
| 528 | 536 | SILSPLYAF | 442.72 | -0.1983 | -0.17157 | No | Class I |
| 529 | 537 | ILSPLYAFA | 13.11 | -0.2675 | 0.02228 | No | Class I |
| 532 | 540 | PLYAFASEA | 196.7 | -0.1739 | 0.10788 | No | Class I |
| 533 | 541 | LYAFASEAA | 320.68 | -0.0527 | 0.12023 | No | Class I |
| 534 | 542 | YAFASEAAR | 67.35 | 0.3454 | 0.0664 | No | Class I |
| 536 | 544 | FASEAARVV | 13.18 | 0.3127 | 0.18978 | No | Class I |
| 537 | 545 | ASEAARVVR | 199.52 | 0.2526 | 0.21765 | No | Class I |
| 539 | 547 | EAARVVRSI | 252.15 | -0.02 | 0.09086 | No | Class I |
| 540 | 548 | AARVVRSIF | 352.02 | -0.1843 | 0.0854 | No | Class I |
| 542 | 550 | RVVRSIFSR | 214.39 | -1.3477 | 0.0318 | No | Class I |
| 543 | 551 | VVRSIFSRT | 147.97 | -0.9335 | -0.01925 | No | Class I |
| 544 | 552 | VRSIFSRTL | 451.48 | -0.9551 | 0.10485 | No | Class I |
| 545 | 553 | RSIFSRTLE | 183.56 | -0.837 | 0.0749 | No | Class I |
| 546 | 554 | SIFSRTLET | 145.28 | -0.0162 | 0.00761 | No | Class I |
| 554 | 562 | TAQNSVRVL | 92.41 | 0.217 | -0.09855 | No | Class I |
| 558 | 566 | SVRVLQKAA | 69.71 | 0.516 | -0.22064 | No | Class I |
| 561 | 569 | VLQKAAITI | 442.86 | 0.4442 | -0.04467 | No | Class I |
| 562 | 570 | LQKAAITIL | 458.27 | 0.3872 | 0.24327 | No | Class I |
| 565 | 573 | AAITILDGI | 315.21 | 0.2519 | 0.23994 | No | Class I |
| 568 | 576 | TILDGISQY | 222.1 | -0.3966 | -0.0303 | No | Class I |
| 571 | 579 | DGISQYSLR | 78.85 | 0.4059 | -0.38565 | No | Class I |
| 573 | 581 | ISQYSLRLI | 80.33 | 0.6954 | -0.17566 | No | Class I |
| 576 | 584 | YSLRLIDAM | 183.55 | 1.0705 | 0.20454 | No | Class I |
| 577 | 585 | SLRLIDAMM | 290.96 | 0.8238 | 0.08654 | No | Class I |
| 579 | 587 | RLIDAMMFT | 16.55 | 0.1484 | -0.14148 | No | Class I |
| 583 | 591 | AMMFTSDLA | 96.07 | 0.0698 | -0.04489 | No | Class I |
| 584 | 592 | MMFTSDLAT | 126.64 | -0.2151 | -0.04966 | No | Class I |
| 587 | 595 | TSDLATNNL | 223.38 | 0.0627 | 0.06144 | No | Class I |
| 590 | 598 | LATNNLVVM | 148.34 | 0.1308 | 0.04831 | No | Class I |
| 591 | 599 | ATNNLVVMA | 331.82 | 0.2199 | -0.04831 | No | Class I |
| 592 | 600 | TNNLVVMAY | 129.23 | 0.3332 | -0.05954 | No | Class I |
| 597 | 605 | VMAYITGGV | 34.74 | 0.3546 | 0.22352 | No | Class I |
| 598 | 606 | MAYITGGVV | 193.79 | 0.6955 | 0.25514 | No | Class I |
| 600 | 608 | YITGGVVQL | 51.78 | 0.5879 | 0.08572 | No | Class I |
| 604 | 612 | GVVQLTSQW | 61.11 | 0.8423 | -0.28472 | No | Class I |
| 606 | 614 | VQLTSQWLT | 358.19 | 0.7181 | -0.05422 | No | Class I |
| 608 | 616 | LTSQWLTNI | 230.82 | 0.0202 | 0.06398 | No | Class I |
| 609 | 617 | TSQWLTNIF | 60.92 | -0.7528 | 0.28333 | No | Class I |
| 612 | 620 | WLTNIFGTV | 29.49 | -0.6679 | 0.29717 | No | Class I |
| 613 | 621 | LTNIFGTVY | 68.63 | -0.5738 | 0.3346 | No | Class I |
| 615 | 623 | NIFGTVYEK | 209.79 | 0.2946 | 0.20414 | No | Class I |
| 616 | 624 | IFGTVYEKL | 341.29 | 0.3123 | 0.04528 | No | Class I |
| 619 | 627 | TVYEKLKPV | 66.87 | 1.0578 | -0.30937 | No | Class I |
| 620 | 628 | VYEKLKPVL | 94.95 | 0.7193 | -0.38354 | No | Class I |
| 623 | 631 | KLKPVLDWL | 63.18 | 0.5582 | 0.09674 | No | Class I |
| 634 | 642 | KFKEGVEFL | 33.14 | 0.2423 | 0.25551 | No | Class I |
| 640 | 648 | EFLRDGWEI | 452.3 | 0.0445 | 0.34742 | No | Class I |
| 641 | 649 | FLRDGWEIV | 36.71 | 0.3189 | 0.44289 | No | Class I |
| 646 | 654 | WEIVKFIST | 285.52 | -0.2003 | 0.0006 | No | Class I |
| 648 | 656 | IVKFISTCA | 160.6 | -0.1925 | 0.02293 | No | Class I |
| 651 | 659 | FISTCACEI | 74.86 | -0.6982 | -0.01731 | No | Class I |
| 657 | 665 | CEIVGGQIV | 176.17 | 0.5324 | 0.12964 | No | Class I |
| 667 | 675 | CAKEIKESV | 440.3 | 0.5148 | -0.05481 | No | Class I |
| 669 | 677 | KEIKESVQT | 395.98 | 0.0262 | -0.26487 | No | Class I |
| 670 | 678 | EIKESVQTF | 69.85 | -0.2031 | -0.16657 | No | Class I |
| 674 | 682 | SVQTFFKLV | 188.62 | -0.1054 | 0.03718 | No | Class I |
| 676 | 684 | QTFFKLVNK | 42.68 | 0.2687 | -0.03358 | No | Class I |
| 677 | 685 | TFFKLVNKF | 114.91 | -0.073 | -0.2824 | No | Class I |
| 678 | 686 | FFKLVNKFL | 79.18 | -0.2845 | -0.16065 | No | Class I |
| 680 | 688 | KLVNKFLAL | 25.98 | 0.1508 | -0.07941 | No | Class I |
| 685 | 693 | FLALCADSI | 16.26 | -0.2029 | -0.09207 | No | Class I |
| 686 | 694 | LALCADSII | 484.36 | -0.5989 | -0.06073 | No | Class I |
| 687 | 695 | ALCADSIII | 498.33 | -0.2375 | 0.07782 | No | Class I |
| 691 | 699 | DSIIIGGAK | 327.8 | 0.5875 | 0.39008 | No | Class I |
| 693 | 701 | IIIGGAKLK | 148.66 | 1.5121 | -0.04135 | No | Class I |
| 697 | 705 | GAKLKALNL | 349.7 | 1.5095 | -0.26747 | No | Class I |
| 701 | 709 | KALNLGETF | 413.56 | 1.5838 | 0.11817 | No | Class I |
| 702 | 710 | ALNLGETFV | 58.55 | 0.8878 | 0.21515 | No | Class I |
| 708 | 716 | TFVTHSKGL | 37.11 | 0.2938 | -0.23397 | No | Class I |
| 709 | 717 | FVTHSKGLY | 223.23 | 0.2037 | -0.29683 | No | Class I |
| 710 | 718 | VTHSKGLYR | 325.42 | -0.1078 | -0.34559 | No | Class I |
| 714 | 722 | KGLYRKCVK | 193.68 | -0.883 | -0.1813 | No | Class I |
| 716 | 724 | LYRKCVKSR | 71.3 | -0.5319 | -0.4925 | No | Class I |
| 723 | 731 | SREETGLLM | 314.24 | 0.5475 | 0.18711 | No | Class I |
| 725 | 733 | EETGLLMPL | 60.35 | 0.4854 | -0.12922 | No | Class I |
| 726 | 734 | ETGLLMPLK | 283.95 | 0.8156 | -0.1921 | No | Class I |
| 729 | 737 | LLMPLKAPK | 24.31 | 0.179 | -0.25542 | No | Class I |
| 731 | 739 | MPLKAPKEI | 102.1 | 0.1775 | -0.31644 | No | Class I |
| 734 | 742 | KAPKEIIFL | 150.43 | 0.1561 | 0.1829 | No | Class I |
| 739 | 747 | IIFLEGETL | 231.16 | 0.1242 | 0.26342 | No | Class I |
| 741 | 749 | FLEGETLPT | 350.1 | 0.0371 | 0.1848 | No | Class I |
| 744 | 752 | GETLPTEVL | 9.79 | -0.3125 | 0.1358 | No | Class I |
| 749 | 757 | TEVLTEEVV | 261.76 | -0.3462 | 0.24291 | No | Class I |
| 751 | 759 | VLTEEVVLK | 485.86 | -0.0668 | 0.27807 | No | Class I |
| 766 | 774 | LEQPTSEAV | 178.71 | -0.3985 | -0.05933 | No | Class I |
| 771 | 779 | SEAVEAPLV | 212.66 | 0.1067 | 0.17273 | No | Class I |
| 776 | 784 | APLVGTPVC | 394.4 | 0.1188 | 0.12224 | No | Class I |
| 781 | 789 | TPVCINGLM | 274.12 | 0.0722 | 0.10478 | No | Class I |
| 791 | 799 | LEIKDTEKY | 410.79 | 1.8468 | -0.15716 | No | Class I |
| 794 | 802 | KDTEKYCAL | 372.06 | 0.3594 | -0.12277 | No | Class I |
| 799 | 807 | YCALAPNMM | 364.51 | 0.7415 | -0.07886 | No | Class I |
| 800 | 808 | CALAPNMMV | 224.64 | 0.4118 | -0.23192 | No | Class I |
| 801 | 809 | ALAPNMMVT | 37.77 | 0.6563 | -0.29414 | No | Class I |
| 805 | 813 | NMMVTNNTF | 437.47 | 0.3592 | 0.03347 | No | Class I |
| 806 | 814 | MMVTNNTFT | 371 | 0.3776 | 0.14123 | No | Class I |
| 807 | 815 | MVTNNTFTL | 157.14 | 0.3894 | 0.15781 | No | Class I |
| 813 | 821 | FTLKGGAPT | 115.63 | 0.8832 | -0.12916 | No | Class I |
| 814 | 822 | TLKGGAPTK | 85.65 | 0.8243 | 0.04725 | No | Class I |
| 825 | 833 | FGDDTVIEV | 374.51 | 0.0343 | 0.277 | No | Class I |
| 828 | 836 | DTVIEVQGY | 28.22 | 0.1358 | 0.20572 | No | Class I |
| 829 | 837 | TVIEVQGYK | 67.32 | 0.1168 | 0.10155 | No | Class I |
| 831 | 839 | IEVQGYKSV | 448.68 | 0.2293 | -0.3523 | No | Class I |
| 835 | 843 | GYKSVNITF | 83.59 | 2.3667 | -0.06736 | No | Class I |
| 837 | 845 | KSVNITFEL | 80.37 | 2.1377 | 0.33033 | No | Class I |
| 840 | 848 | NITFELDER | 68.17 | 1.7263 | 0.29468 | No | Class I |
| 841 | 849 | ITFELDERI | 402.45 | 1.0232 | 0.26357 | No | Class I |
| 848 | 856 | RIDKVLNEK | 168.26 | -0.2842 | -0.127 | No | Class I |
| 851 | 859 | KVLNEKCSA | 465.41 | 0.0289 | -0.25777 | No | Class I |
| 852 | 860 | VLNEKCSAY | 280.78 | 0.8473 | -0.27886 | No | Class I |
| 854 | 862 | NEKCSAYTV | 167.89 | 0.8005 | -0.22896 | No | Class I |
| 860 | 868 | YTVELGTEV | 144.55 | 0.7185 | 0.22651 | No | Class I |
| 866 | 874 | TEVNEFACV | 383.16 | 0.6182 | 0.21611 | No | Class I |
| 869 | 877 | NEFACVVAD | 287.45 | 0.9086 | 0.12143 | No | Class I |
| 871 | 879 | FACVVADAV | 127.84 | 0.5382 | 0.14265 | No | Class I |
| 873 | 881 | CVVADAVIK | 231.36 | 0.0862 | 0.2238 | No | Class I |
| 875 | 883 | VADAVIKTL | 202.63 | -0.0308 | 0.05273 | No | Class I |
| 878 | 886 | AVIKTLQPV | 20.84 | 0.505 | -0.25068 | No | Class I |
| 881 | 889 | KTLQPVSEL | 471.84 | 0.4736 | -0.17322 | No | Class I |
| 882 | 890 | TLQPVSELL | 132.81 | 0.295 | -0.08627 | No | Class I |
| 887 | 895 | SELLTPLGI | 412.07 | 0.3577 | 0.02304 | No | Class I |
| 889 | 897 | LLTPLGIDL | 258.49 | 0.9999 | 0.14782 | No | Class I |
| 898 | 906 | DEWSMATYY | 481.3 | 0.2181 | -0.19814 | No | Class I |
| 899 | 907 | EWSMATYYL | 490.07 | 0.5905 | -0.16104 | No | Class I |
| 900 | 908 | WSMATYYLF | 316.71 | 0.3328 | 0.00709 | No | Class I |
| 906 | 914 | YLFDESGEF | 189.33 | -0.0602 | 0.08919 | No | Class I |
| 912 | 920 | GEFKLASHM | 50.73 | 0.4407 | -0.27369 | No | Class I |
| 915 | 923 | KLASHMYCS | 272.57 | 0.1716 | -0.32219 | No | Class I |
| 916 | 924 | LASHMYCSF | 59.74 | 0.4943 | -0.33779 | No | Class I |
| 917 | 925 | ASHMYCSFY | 178.49 | 0.5435 | -0.29177 | No | Class I |
| 919 | 927 | HMYCSFYPP | 343.51 | 0.6885 | -0.11595 | No | Class I |
| 939 | 947 | EEFEPSTQY | 26.45 | 0.5736 | -0.0627 | No | Class I |
| 941 | 949 | FEPSTQYEY | 398.72 | 0.9195 | -0.18593 | No | Class I |
| 947 | 955 | YEYGTEDDY | 217.49 | 0.7611 | 0.19663 | No | Class I |
| 954 | 962 | DYQGKPLEF | 298.18 | 1.6136 | -0.1748 | No | Class I |
| 960 | 968 | LEFGATSAA | 295.83 | 1.8562 | 0.02998 | No | Class I |
| 961 | 969 | EFGATSAAL | 195.53 | 1.2984 | -0.01168 | No | Class I |
| 994 | 1002 | SEDNQTTTI | 99.37 | 0.4531 | -0.02013 | No | Class I |
| 997 | 1005 | NQTTTIQTI | 464.16 | 0.2996 | 0.13966 | No | Class I |
| 1000 | 1008 | TTIQTIVEV | 449.32 | 0.125 | 0.18306 | No | Class I |
| 1004 | 1012 | TIVEVQPQL | 430.06 | 1.35 | -0.03173 | No | Class I |
| 1006 | 1014 | VEVQPQLEM | 454.74 | 1.5772 | -0.17386 | No | Class I |
| 1011 | 1019 | QLEMELTPV | 224.64 | 2.1214 | -0.03086 | No | Class I |
| 1012 | 1020 | LEMELTPVV | 239.4 | 1.9688 | 0.08425 | No | Class I |
| 1014 | 1022 | MELTPVVQT | 383.32 | 1.6356 | 0.03068 | No | Class I |
| 1017 | 1025 | TPVVQTIEV | 168.01 | 0.5889 | 0.1495 | No | Class I |
| 1020 | 1028 | VQTIEVNSF | 105.4 | 0.0873 | 0.18076 | No | Class I |
| 1023 | 1031 | IEVNSFSGY | 347.64 | -0.0158 | -0.16383 | No | Class I |
| 1024 | 1032 | EVNSFSGYL | 128.94 | -0.3329 | -0.18386 | No | Class I |
| 1025 | 1033 | VNSFSGYLK | 310.7 | -0.4026 | -0.0747 | No | Class I |
| 1026 | 1034 | NSFSGYLKL | 47.53 | 0.0295 | -0.23431 | No | Class I |
| 1030 | 1038 | GYLKLTDNV | 85.28 | 0.8182 | -0.17992 | No | Class I |
| 1031 | 1039 | YLKLTDNVY | 253.19 | 0.9118 | -0.00382 | No | Class I |
| 1033 | 1041 | KLTDNVYIK | 129.77 | 0.7238 | 0.14212 | No | Class I |
| 1038 | 1046 | VYIKNADIV | 337.45 | 1.6068 | -0.04679 | No | Class I |
| 1053 | 1061 | KPTVVVNAA | 298.54 | 0.4319 | 0.1506 | No | Class I |
| 1055 | 1063 | TVVVNAANV | 399.2 | 0.644 | 0.11471 | No | Class I |
| 1056 | 1064 | VVVNAANVY | 235.46 | 0.4078 | 0.10048 | No | Class I |
| 1057 | 1065 | VVNAANVYL | 495.05 | 0.4769 | 0.10196 | No | Class I |
| 1058 | 1066 | VNAANVYLK | 325.74 | 0.8781 | 0.07503 | No | Class I |
| 1063 | 1071 | VYLKHGGGV | 231.42 | 0.5905 | -0.1088 | No | Class I |
| 1064 | 1072 | YLKHGGGVA | 85.6 | 0.4392 | 0.08017 | No | Class I |
| 1070 | 1078 | GVAGALNKA | 146.78 | 0.5787 | -0.057 | No | Class I |
| 1074 | 1082 | ALNKATNNA | 50.32 | 0.0403 | -0.1537 | No | Class I |
| 1077 | 1085 | KATNNAMQV | 95.07 | 0.6703 | -0.17926 | No | Class I |
| 1083 | 1091 | MQVESDDYI | 380.85 | 0.4191 | -0.00951 | No | Class I |
| 1089 | 1097 | DYIATNGPL | 110.63 | -0.0964 | 0.1364 | No | Class I |
| 1090 | 1098 | YIATNGPLK | 270.32 | -0.3944 | 0.06152 | No | Class I |
| 1091 | 1099 | IATNGPLKV | 390.1 | -0.3525 | -0.10671 | No | Class I |
| 1095 | 1103 | GPLKVGGSC | 250.82 | 1.0921 | -0.21656 | No | Class I |
| 1097 | 1105 | LKVGGSCVL | 174.17 | 1.0384 | -0.09661 | No | Class I |
| 1104 | 1112 | VLSGHNLAK | 19.69 | 0.1315 | 0.01931 | No | Class I |
| 1107 | 1115 | GHNLAKHCL | 276.08 | -0.1279 | -0.18236 | No | Class I |
| 1109 | 1117 | NLAKHCLHV | 61.81 | 0.2467 | -0.21401 | No | Class I |
| 1110 | 1118 | LAKHCLHVV | 488.97 | 0.4646 | -0.04897 | No | Class I |
| 1114 | 1122 | CLHVVGPNV | 445.16 | 0.123 | 0.111 | No | Class I |
| 1116 | 1124 | HVVGPNVNK | 132.29 | -0.0389 | 0.06167 | No | Class I |
| 1127 | 1135 | DIQLLKSAY | 313.18 | 0.5957 | -0.37932 | No | Class I |
| 1130 | 1138 | LLKSAYENF | 32.55 | -0.0917 | -0.12113 | No | Class I |
| 1133 | 1141 | SAYENFNQH | 120.25 | -0.0161 | 0.13031 | No | Class I |
| 1135 | 1143 | YENFNQHEV | 63.02 | 0.2099 | 0.08616 | No | Class I |
| 1137 | 1145 | NFNQHEVLL | 70.14 | 0.3044 | 0.03545 | No | Class I |
| 1140 | 1148 | QHEVLLAPL | 398.69 | 0.5005 | 0.07934 | No | Class I |
| 1141 | 1149 | HEVLLAPLL | 111.64 | 0.2468 | 0.01243 | No | Class I |
| 1143 | 1151 | VLLAPLLSA | 46.66 | 0.2836 | -0.09149 | No | Class I |
| 1144 | 1152 | LLAPLLSAG | 86.08 | 0.1405 | -0.13646 | No | Class I |
| 1145 | 1153 | LAPLLSAGI | 294.76 | 0.6365 | -0.12847 | No | Class I |
| 1146 | 1154 | APLLSAGIF | 109.66 | 0.1575 | -0.03267 | No | Class I |
| 1148 | 1156 | LLSAGIFGA | 15.71 | 0.3693 | 0.26255 | No | Class I |
| 1154 | 1162 | FGADPIHSL | 137.4 | 0.4569 | 0.08014 | No | Class I |
| 1155 | 1163 | GADPIHSLR | 267.91 | 0.7146 | 0.00999 | No | Class I |
| 1161 | 1169 | SLRVCVDTV | 142.9 | -1.0415 | 0.0861 | No | Class I |
| 1168 | 1176 | TVRTNVYLA | 35.2 | 0.3461 | 0.07882 | No | Class I |
| 1169 | 1177 | VRTNVYLAV | 93.49 | 0.2877 | 0.05631 | No | Class I |
| 1170 | 1178 | RTNVYLAVF | 287.83 | 0.065 | 0.08254 | No | Class I |
| 1172 | 1180 | NVYLAVFDK | 401.42 | 0.1673 | 0.17636 | No | Class I |
| 1174 | 1182 | YLAVFDKNL | 61.9 | 0.284 | 0.00334 | No | Class I |
| 1175 | 1183 | LAVFDKNLY | 365.58 | 0.1894 | -0.06214 | No | Class I |
| 1181 | 1189 | NLYDKLVSS | 92.69 | 0.1619 | -0.26114 | No | Class I |
| 1182 | 1190 | LYDKLVSSF | 76.96 | -0.091 | -0.41802 | No | Class I |
| 1183 | 1191 | YDKLVSSFL | 424.57 | -0.3573 | -0.26791 | No | Class I |
| 1185 | 1193 | KLVSSFLEM | 58.34 | -0.0423 | -0.15483 | No | Class I |
| 1186 | 1194 | LVSSFLEMK | 231.13 | 0.7565 | -0.13471 | No | Class I |
| 1189 | 1197 | SFLEMKSEK | 468.52 | 0.9698 | -0.35797 | No | Class I |
| 1191 | 1199 | LEMKSEKQV | 352.28 | 1.1517 | -0.59053 | No | Class I |
| 1195 | 1203 | SEKQVEQKI | 404.85 | 0.0037 | -0.27587 | No | Class I |
| 1206 | 1214 | IPKEEVKPF | 401.39 | 0.4749 | -0.02137 | No | Class I |
| 1214 | 1222 | FITESKPSV | 71.77 | 0.6293 | -0.35677 | No | Class I |
| 1217 | 1225 | ESKPSVEQR | 161.01 | 0.9755 | -0.18658 | No | Class I |
| 1231 | 1239 | KIKACVEEV | 405.51 | -0.6462 | 0.09873 | No | Class I |
| 1239 | 1247 | VTTTLEETK | 310.83 | 0.9372 | 0.24229 | No | Class I |
| 1240 | 1248 | TTTLEETKF | 394.52 | 0.7861 | 0.09995 | No | Class I |
| 1241 | 1249 | TTLEETKFL | 460.92 | 0.5613 | 0.11759 | No | Class I |
| 1247 | 1255 | KFLTENLLL | 38.39 | -0.6184 | 0.11103 | No | Class I |
| 1248 | 1256 | FLTENLLLY | 67.39 | -0.2678 | 0.08077 | No | Class I |
| 1249 | 1257 | LTENLLLYI | 326.92 | -0.1902 | -0.00677 | No | Class I |
| 1255 | 1263 | LYIDINGNL | 23.59 | -0.2906 | 0.21385 | No | Class I |
| 1262 | 1270 | NLHPDSATL | 479.38 | 0.6135 | -0.07909 | No | Class I |
| 1264 | 1272 | HPDSATLVS | 436.53 | 0.4237 | -0.06987 | No | Class I |
| 1270 | 1278 | LVSDIDITF | 345.13 | 1.783 | 0.2541 | No | Class I |
| 1271 | 1279 | VSDIDITFL | 157.52 | 2.2906 | 0.38916 | No | Class I |
| 1272 | 1280 | SDIDITFLK | 477.13 | 1.9096 | 0.32398 | No | Class I |
| 1277 | 1285 | TFLKKDAPY | 122.05 | 0.2485 | -0.38318 | No | Class I |
| 1278 | 1286 | FLKKDAPYI | 21.76 | 0.3364 | -0.24009 | No | Class I |
| 1283 | 1291 | APYIVGDVV | 276.01 | 0.2349 | 0.24766 | No | Class I |
| 1290 | 1298 | VVQEGVLTA | 393.2 | 0.227 | 0.14833 | No | Class I |
| 1291 | 1299 | VQEGVLTAV | 301.62 | 0.4206 | 0.15198 | No | Class I |
| 1292 | 1300 | QEGVLTAVV | 321.56 | 0.2219 | 0.13542 | No | Class I |
| 1295 | 1303 | VLTAVVIPT | 113.65 | 0.2386 | 0.23687 | No | Class I |
| 1296 | 1304 | LTAVVIPTK | 154.95 | 1.1167 | 0.23304 | No | Class I |
| 1297 | 1305 | TAVVIPTKK | 294.81 | 1.3254 | 0.08086 | No | Class I |
| 1308 | 1316 | GTTEMLAKA | 378.11 | 0.135 | -0.16107 | No | Class I |
| 1311 | 1319 | EMLAKALRK | 237.9 | 0.0675 | -0.11652 | No | Class I |
| 1312 | 1320 | MLAKALRKV | 32.09 | 0.2132 | -0.25896 | No | Class I |
| 1321 | 1329 | PTDNYITTY | 12.05 | -0.3649 | 0.17781 | No | Class I |
| 1326 | 1334 | ITTYPGQGL | 66.57 | 0.0047 | -0.04798 | No | Class I |
| 1329 | 1337 | YPGQGLNGY | 231.66 | -0.0391 | -0.06866 | No | Class I |
| 1331 | 1339 | GQGLNGYTV | 293.73 | 0.0374 | 0.045 | No | Class I |
| 1335 | 1343 | NGYTVEEAK | 420.18 | 0.3847 | 0.27967 | No | Class I |
| 1337 | 1345 | YTVEEAKTV | 234.19 | 0.5782 | 0.08916 | No | Class I |
| 1344 | 1352 | TVLKKCKSA | 473.38 | -0.0585 | -0.76001 | No | Class I |
| 1345 | 1353 | VLKKCKSAF | 372.15 | -0.4464 | -0.65926 | No | Class I |
| 1348 | 1356 | KCKSAFYIL | 326.93 | 0.6114 | -0.01353 | No | Class I |
| 1350 | 1358 | KSAFYILPS | 138.09 | 0.6413 | 0.23634 | No | Class I |
| 1351 | 1359 | SAFYILPSI | 446.61 | 0.5033 | 0.04742 | No | Class I |
| 1352 | 1360 | AFYILPSII | 263.14 | 0.4981 | 0.04962 | No | Class I |
| 1356 | 1364 | LPSIISNEK | 384.75 | 0.7159 | 0.10713 | No | Class I |
| 1360 | 1368 | ISNEKQEIL | 360.7 | 0.7304 | -0.05813 | No | Class I |
| 1365 | 1373 | QEILGTVSW | 227.98 | 0.6809 | 0.03976 | No | Class I |
| 1368 | 1376 | LGTVSWNLR | 101.72 | 2.5223 | 0.08961 | No | Class I |
| 1370 | 1378 | TVSWNLREM | 107.54 | 1.8822 | 0.25463 | No | Class I |
| 1371 | 1379 | VSWNLREML | 70.61 | 1.6102 | 0.08521 | No | Class I |
| 1374 | 1382 | NLREMLAHA | 14.58 | -0.0372 | -0.01197 | No | Class I |
| 1378 | 1386 | MLAHAEETR | 390.27 | 0.588 | 0.28478 | No | Class I |
| 1380 | 1388 | AHAEETRKL | 335.77 | 0.3697 | 0.16517 | No | Class I |
| 1381 | 1389 | HAEETRKLM | 276.38 | 0.1348 | 0.03129 | No | Class I |
| 1389 | 1397 | MPVCVETKA | 151.76 | 0.8443 | 0.00036 | No | Class I |
| 1393 | 1401 | VETKAIVST | 308.15 | 0.4315 | -0.10284 | No | Class I |
| 1394 | 1402 | ETKAIVSTI | 456.6 | 0.1083 | 0.02089 | No | Class I |
| 1396 | 1404 | KAIVSTIQR | 238.98 | -0.152 | 0.00482 | No | Class I |
| 1397 | 1405 | AIVSTIQRK | 118.49 | 1.1548 | -0.05751 | No | Class I |
| 1398 | 1406 | IVSTIQRKY | 148.55 | 1.0912 | -0.0764 | No | Class I |
| 1399 | 1407 | VSTIQRKYK | 224.07 | 1.3057 | -0.10172 | No | Class I |
| 1402 | 1410 | IQRKYKGIK | 71.16 | 0.5121 | -0.30044 | No | Class I |
| 1410 | 1418 | KIQEGVVDY | 113.03 | 0.4976 | 0.18281 | No | Class I |
| 1413 | 1421 | EGVVDYGAR | 361.17 | 0.5796 | 0.12452 | No | Class I |
| 1414 | 1422 | GVVDYGARF | 367.99 | 0.79 | 0.12728 | No | Class I |
| 1415 | 1423 | VVDYGARFY | 71.01 | 0.4908 | 0.18539 | No | Class I |
| 1416 | 1424 | VDYGARFYF | 499.37 | 1.0458 | 0.21636 | No | Class I |
| 1417 | 1425 | DYGARFYFY | 123.85 | 0.5667 | 0.27625 | No | Class I |
| 1419 | 1427 | GARFYFYTS | 164.12 | 0.0028 | 0.26076 | No | Class I |
| 1422 | 1430 | FYFYTSKTT | 71.94 | 0.524 | -0.24297 | No | Class I |
| 1423 | 1431 | YFYTSKTTV | 254.08 | 0.2042 | -0.2708 | No | Class I |
| 1424 | 1432 | FYTSKTTVA | 42.67 | 0.3361 | -0.27045 | No | Class I |
| 1426 | 1434 | TSKTTVASL | 236.26 | 0.6856 | -0.01792 | No | Class I |
| 1430 | 1438 | TVASLINTL | 116.92 | 0.1639 | -0.02207 | No | Class I |
| 1433 | 1441 | SLINTLNDL | 54.87 | 0.2271 | 0.07155 | No | Class I |
| 1440 | 1448 | DLNETLVTM | 454.04 | 0.7006 | 0.18353 | No | Class I |
| 1442 | 1450 | NETLVTMPL | 21.31 | 0.2505 | -0.0765 | No | Class I |
| 1444 | 1452 | TLVTMPLGY | 12.93 | 0.5281 | -0.11854 | No | Class I |
| 1445 | 1453 | LVTMPLGYV | 48.35 | 0.564 | -0.1589 | No | Class I |
| 1448 | 1456 | MPLGYVTHG | 360.84 | 1.2395 | 0.11742 | No | Class I |
| 1451 | 1459 | GYVTHGLNL | 59.16 | 0.8595 | 0.10272 | No | Class I |
| 1457 | 1465 | LNLEEAARY | 448.88 | 0.7721 | 0.29474 | No | Class I |
| 1461 | 1469 | EAARYMRSL | 493.79 | -0.1896 | -0.1571 | No | Class I |
| 1462 | 1470 | AARYMRSLK | 311.92 | -0.0269 | -0.2553 | No | Class I |
| 1463 | 1471 | ARYMRSLKV | 252.61 | 0.0163 | -0.41859 | No | Class I |
| 1465 | 1473 | YMRSLKVPA | 30.5 | -0.0639 | -0.33511 | No | Class I |
| 1467 | 1475 | RSLKVPATV | 337.15 | 0.3757 | -0.13514 | No | Class I |
| 1471 | 1479 | VPATVSVSS | 385.42 | 0.7956 | -0.12559 | No | Class I |
| 1478 | 1486 | SSPDAVTAY | 444.09 | 0.2934 | 0.1513 | No | Class I |
| 1481 | 1489 | DAVTAYNGY | 86.87 | 0.083 | 0.10142 | No | Class I |
| 1484 | 1492 | TAYNGYLTS | 396.42 | 0.151 | 0.03513 | No | Class I |
| 1493 | 1501 | SSKTPEEHF | 214.4 | 0.0873 | 0.15591 | No | Class I |
| 1499 | 1507 | EHFIETISL | 266.83 | 0.09 | 0.32162 | No | Class I |
| 1503 | 1511 | ETISLAGSY | 3.36 | 0.5916 | -0.1653 | No | Class I |
| 1504 | 1512 | TISLAGSYK | 49.53 | 0.824 | -0.13664 | No | Class I |
| 1510 | 1518 | SYKDWSYSG | 430.01 | 1.7748 | -0.08749 | No | Class I |
| 1515 | 1523 | SYSGQSTQL | 49.19 | 0.8521 | -0.32305 | No | Class I |
| 1519 | 1527 | QSTQLGIEF | 52.95 | 1.9341 | 0.08796 | No | Class I |
| 1521 | 1529 | TQLGIEFLK | 111.23 | 1.4708 | 0.34667 | No | Class I |
| 1527 | 1535 | FLKRGDKSV | 13.74 | 0.9989 | -0.2427 | No | Class I |
| 1529 | 1537 | KRGDKSVYY | 206.49 | 0.9685 | -0.29973 | No | Class I |
| 1535 | 1543 | VYYTSNPTT | 178.44 | 0.3379 | -0.11601 | No | Class I |
| 1536 | 1544 | YYTSNPTTF | 14.03 | 0.1415 | -0.11517 | No | Class I |
| 1537 | 1545 | YTSNPTTFH | 392.26 | 0.3043 | 0.06669 | No | Class I |
| 1538 | 1546 | TSNPTTFHL | 51.45 | 0.5611 | 0.17878 | No | Class I |
| 1543 | 1551 | TFHLDGEVI | 464.9 | -0.3676 | 0.16146 | No | Class I |
| 1548 | 1556 | GEVITFDNL | 16.13 | 0.2551 | 0.31026 | No | Class I |
| 1549 | 1557 | EVITFDNLK | 352.78 | 0.4738 | 0.2052 | No | Class I |
| 1551 | 1559 | ITFDNLKTL | 491.34 | 0.6567 | -0.11574 | No | Class I |
| 1552 | 1560 | TFDNLKTLL | 226.98 | -0.1457 | -0.18683 | No | Class I |
| 1555 | 1563 | NLKTLLSLR | 40.55 | 0.8788 | -0.19828 | No | Class I |
| 1557 | 1565 | KTLLSLREV | 322.04 | 0.9705 | -0.08412 | No | Class I |
| 1558 | 1566 | TLLSLREVR | 79.31 | 1.7234 | -0.02353 | No | Class I |
| 1561 | 1569 | SLREVRTIK | 62.76 | 0.7073 | 0.31699 | No | Class I |
| 1563 | 1571 | REVRTIKVF | 171.69 | -0.1086 | 0.07068 | No | Class I |
| 1566 | 1574 | RTIKVFTTV | 147.09 | -0.2404 | 0.03204 | No | Class I |
| 1569 | 1577 | KVFTTVDNI | 290.68 | -0.1001 | 0.16866 | No | Class I |
| 1571 | 1579 | FTTVDNINL | 293.86 | 1.2065 | 0.17819 | No | Class I |
| 1572 | 1580 | TTVDNINLH | 493.03 | 0.8953 | 0.14276 | No | Class I |
| 1576 | 1584 | NINLHTQVV | 270.65 | 0.4194 | -0.01886 | No | Class I |
| 1580 | 1588 | HTQVVDMSM | 35.06 | 1.174 | -0.17984 | No | Class I |
| 1582 | 1590 | QVVDMSMTY | 277.42 | 1.6094 | -0.41653 | No | Class I |
| 1586 | 1594 | MSMTYGQQF | 225.46 | 0.7602 | -0.15508 | No | Class I |
| 1590 | 1598 | YGQQFGPTY | 297.36 | 0.3233 | 0.00506 | No | Class I |
| 1591 | 1599 | GQQFGPTYL | 462.67 | 0.4322 | 0.13336 | No | Class I |
| 1606 | 1614 | KIKPHNSHE | 222.56 | 0.7456 | -0.17647 | No | Class I |
| 1611 | 1619 | NSHEGKTFY | 228.46 | -0.0341 | 0.04241 | No | Class I |
| 1612 | 1620 | SHEGKTFYV | 440.87 | 0.0319 | -0.01022 | No | Class I |
| 1613 | 1621 | HEGKTFYVL | 123.48 | 0.0616 | -0.037 | No | Class I |
| 1619 | 1627 | YVLPNDDTL | 89.4 | -0.1406 | 0.04122 | No | Class I |
| 1621 | 1629 | LPNDDTLRV | 450.43 | -0.6744 | 0.09924 | No | Class I |
| 1626 | 1634 | TLRVEAFEY | 54.59 | 0.4509 | 0.34997 | No | Class I |
| 1627 | 1635 | LRVEAFEYY | 402.78 | 0.3832 | 0.34479 | No | Class I |
| 1632 | 1640 | FEYYHTTDP | 387.58 | 1.0972 | 0.10884 | No | Class I |
| 1635 | 1643 | YHTTDPSFL | 205.42 | 1.2453 | -0.0084 | No | Class I |
| 1637 | 1645 | TTDPSFLGR | 104.96 | 1.7599 | -0.04442 | No | Class I |
| 1641 | 1649 | SFLGRYMSA | 280.57 | 0.037 | -0.16744 | No | Class I |
| 1642 | 1650 | FLGRYMSAL | 12.85 | -0.0882 | -0.22258 | No | Class I |
| 1645 | 1653 | RYMSALNHT | 151.91 | 0.4947 | -0.18237 | No | Class I |
| 1646 | 1654 | YMSALNHTK | 88.43 | 0.8228 | 0.01876 | No | Class I |
| 1647 | 1655 | MSALNHTKK | 352.88 | 0.9958 | -0.06755 | No | Class I |
| 1648 | 1656 | SALNHTKKW | 45.8 | 0.1935 | -0.25007 | No | Class I |
| 1649 | 1657 | ALNHTKKWK | 132.22 | 0.258 | -0.18733 | No | Class I |
| 1652 | 1660 | HTKKWKYPQ | 366.32 | -0.2801 | -0.2839 | No | Class I |
| 1655 | 1663 | KWKYPQVNG | 220.45 | 0.1965 | -0.1625 | No | Class I |
| 1656 | 1664 | WKYPQVNGL | 178.34 | 0.3456 | -0.07196 | No | Class I |
| 1658 | 1666 | YPQVNGLTS | 467.76 | 0.3408 | 0.04286 | No | Class I |
| 1660 | 1668 | QVNGLTSIK | 214.46 | 0.7752 | -0.00412 | No | Class I |
| 1668 | 1676 | KWADNNCYL | 43.71 | 0.3716 | -0.02503 | No | Class I |
| 1675 | 1683 | YLATALLTL | 6.4 | 0.3018 | 0.09274 | No | Class I |
| 1678 | 1686 | TALLTLQQI | 439.27 | 0.6505 | -0.15284 | No | Class I |
| 1680 | 1688 | LLTLQQIEL | 209.93 | 1.2748 | -0.04958 | No | Class I |
| 1681 | 1689 | LTLQQIELK | 466.44 | 1.8084 | -0.02966 | No | Class I |
| 1682 | 1690 | TLQQIELKF | 463.73 | 1.6841 | -0.06567 | No | Class I |
| 1686 | 1694 | IELKFNPPA | 51.28 | 1.9693 | -0.12853 | No | Class I |
| 1687 | 1695 | ELKFNPPAL | 176.67 | 1.7285 | 0.04456 | No | Class I |
| 1691 | 1699 | NPPALQDAY | 106.44 | 0.5266 | -0.04249 | No | Class I |
| 1694 | 1702 | ALQDAYYRA | 80.3 | 0.1677 | 0.04646 | No | Class I |
| 1695 | 1703 | LQDAYYRAR | 311.43 | 0.6243 | 0.10603 | No | Class I |
| 1699 | 1707 | YYRARAGEA | 403.29 | 0.5944 | 0.2305 | No | Class I |
| 1700 | 1708 | YRARAGEAA | 170 | 0.966 | 0.24214 | No | Class I |
| 1701 | 1709 | RARAGEAAN | 425.16 | 0.894 | 0.2393 | No | Class I |
| 1705 | 1713 | GEAANFCAL | 194.75 | 0.7491 | 0.13333 | No | Class I |
| 1707 | 1715 | AANFCALIL | 454.22 | 0.4556 | 0.16843 | No | Class I |
| 1712 | 1720 | ALILAYCNK | 92.17 | 0.4033 | 0.01738 | No | Class I |
| 1714 | 1722 | ILAYCNKTV | 69.62 | 0.5104 | -0.20893 | No | Class I |
| 1717 | 1725 | YCNKTVGEL | 421.23 | 0.4219 | -0.05534 | No | Class I |
| 1720 | 1728 | KTVGELGDV | 280.31 | 1.0744 | 0.17612 | No | Class I |
| 1721 | 1729 | TVGELGDVR | 117.86 | 1.3603 | 0.17569 | No | Class I |
| 1727 | 1735 | DVRETMSYL | 85.86 | 0.7418 | -0.15173 | No | Class I |
| 1728 | 1736 | VRETMSYLF | 419.71 | 0.143 | -0.26477 | No | Class I |
| 1730 | 1738 | ETMSYLFQH | 322.15 | 0.0267 | -0.20639 | No | Class I |
| 1731 | 1739 | TMSYLFQHA | 319.87 | 0.1461 | -0.03688 | No | Class I |
| 1732 | 1740 | MSYLFQHAN | 362.81 | 0.4374 | 0.04276 | No | Class I |
| 1733 | 1741 | SYLFQHANL | 71.21 | 0.584 | 0.06109 | No | Class I |
| 1736 | 1744 | FQHANLDSC | 157.23 | 0.1365 | -0.04481 | No | Class I |
| 1738 | 1746 | HANLDSCKR | 458.12 | -0.7835 | -0.31889 | No | Class I |
| 1757 | 1765 | QQQTTLKGV | 469.92 | 0.5149 | -0.13338 | No | Class I |
| 1760 | 1768 | TTLKGVEAV | 60.03 | 0.8423 | -0.04138 | No | Class I |
| 1761 | 1769 | TLKGVEAVM | 464.69 | 0.8998 | 0.15569 | No | Class I |
| 1763 | 1771 | KGVEAVMYM | 148.48 | 0.6336 | 0.04075 | No | Class I |
| 1766 | 1774 | EAVMYMGTL | 249.86 | 0.1967 | -0.28092 | No | Class I |
| 1768 | 1776 | VMYMGTLSY | 6.75 | 0.8139 | -0.21438 | No | Class I |
| 1769 | 1777 | MYMGTLSYE | 359.72 | 1.2662 | -0.13732 | No | Class I |
| 1772 | 1780 | GTLSYEQFK | 127.6 | 0.942 | -0.10878 | No | Class I |
| 1773 | 1781 | TLSYEQFKK | 178.43 | 0.0537 | -0.09616 | No | Class I |
| 1783 | 1791 | VQIPCTCGK | 386.1 | 1.0782 | -0.00962 | No | Class I |
| 1787 | 1795 | CTCGKQATK | 400.46 | 0.6543 | -0.24674 | No | Class I |
| 1796 | 1804 | YLVQQESPF | 19.55 | 0.5268 | -0.26781 | No | Class I |
| 1797 | 1805 | LVQQESPFV | 109.8 | 0.693 | -0.15335 | No | Class I |
| 1798 | 1806 | VQQESPFVM | 305.85 | 0.8422 | 0.01453 | No | Class I |
| 1802 | 1810 | SPFVMMSAP | 79.61 | 0.2846 | -0.37352 | No | Class I |
| 1804 | 1812 | FVMMSAPPA | 26.97 | 0.4871 | -0.37381 | No | Class I |
| 1806 | 1814 | MMSAPPAQY | 179.86 | 0.3458 | -0.07023 | No | Class I |
| 1808 | 1816 | SAPPAQYEL | 21.46 | 0.7462 | -0.03032 | No | Class I |
| 1813 | 1821 | QYELKHGTF | 111.83 | 1.5575 | -0.10693 | No | Class I |
| 1814 | 1822 | YELKHGTFT | 333.4 | 1.656 | -0.05604 | No | Class I |
| 1819 | 1827 | GTFTCASEY | 115.24 | 0.2357 | -0.01973 | No | Class I |
| 1823 | 1831 | CASEYTGNY | 445.23 | -0.1382 | 0.10481 | No | Class I |
| 1825 | 1833 | SEYTGNYQC | 107.38 | 0.6539 | -0.00603 | No | Class I |
| 1829 | 1837 | GNYQCGHYK | 153.93 | 0.5066 | -0.11322 | No | Class I |
| 1831 | 1839 | YQCGHYKHI | 472.02 | 0.2309 | -0.11848 | No | Class I |
| 1838 | 1846 | HITSKETLY | 224.47 | 0.918 | -0.24334 | No | Class I |
| 1844 | 1852 | TLYCIDGAL | 248.06 | 0.7378 | 0.14649 | No | Class I |
| 1845 | 1853 | LYCIDGALL | 345.69 | 0.4907 | 0.19646 | No | Class I |
| 1847 | 1855 | CIDGALLTK | 422.27 | 0.5028 | 0.08228 | No | Class I |
| 1851 | 1859 | ALLTKSSEY | 421.93 | 0.775 | -0.41139 | No | Class I |
| 1852 | 1860 | LLTKSSEYK | 167.23 | 0.2156 | -0.43889 | No | Class I |
| 1855 | 1863 | KSSEYKGPI | 233.42 | -0.2536 | -0.13743 | No | Class I |
| 1865 | 1873 | DVFYKENSY | 195.16 | 0.4052 | -0.18359 | No | Class I |
| 1867 | 1875 | FYKENSYTT | 91.07 | 0.2081 | -0.11172 | No | Class I |
| 1869 | 1877 | KENSYTTTI | 201.06 | 0.4376 | -0.08019 | No | Class I |
| 1870 | 1878 | ENSYTTTIK | 141.17 | 0.2433 | 0.12744 | No | Class I |
| 1872 | 1880 | SYTTTIKPV | 253.55 | 0.8092 | 0.02626 | No | Class I |
| 1874 | 1882 | TTTIKPVTY | 101.55 | 1.0113 | -0.0164 | No | Class I |
| 1875 | 1883 | TTIKPVTYK | 30.29 | 0.5561 | -0.11514 | No | Class I |
| 1880 | 1888 | VTYKLDGVV | 189.02 | 0.4162 | -0.1554 | No | Class I |
| 1887 | 1895 | VVCTEIDPK | 112.6 | 1.9854 | 0.25658 | No | Class I |
| 1891 | 1899 | EIDPKLDNY | 311.08 | 1.6159 | -0.20946 | No | Class I |
| 1898 | 1906 | NYYKKDNSY | 341.11 | 0.4268 | -0.50944 | No | Class I |
| 1899 | 1907 | YYKKDNSYF | 35.86 | 0.6729 | -0.41327 | No | Class I |
| 1904 | 1912 | NSYFTEQPI | 483.35 | 0.3395 | 0.14441 | No | Class I |
| 1906 | 1914 | YFTEQPIDL | 476.58 | 1.0229 | 0.11539 | No | Class I |
| 1907 | 1915 | FTEQPIDLV | 302.54 | 1.2023 | 0.04266 | No | Class I |
| 1915 | 1923 | VPNQPYPNA | 436.86 | -0.0252 | -0.14608 | No | Class I |
| 1917 | 1925 | NQPYPNASF | 455.42 | 0.5072 | -0.08785 | No | Class I |
| 1920 | 1928 | YPNASFDNF | 398 | 0.1805 | 0.00131 | No | Class I |
| 1922 | 1930 | NASFDNFKF | 79.96 | 0.8484 | 0.05241 | No | Class I |
| 1923 | 1931 | ASFDNFKFV | 457.2 | 0.96 | 0.05062 | No | Class I |
| 1929 | 1937 | KFVCDNIKF | 473.03 | 0.649 | -0.03902 | No | Class I |
| 1930 | 1938 | FVCDNIKFA | 109.47 | 0.9569 | 0.0102 | No | Class I |
| 1936 | 1944 | KFADDLNQL | 112.45 | -0.345 | -0.02696 | No | Class I |
| 1940 | 1948 | DLNQLTGYK | 488.03 | -0.1216 | -0.06648 | No | Class I |
| 1945 | 1953 | TGYKKPASR | 119.87 | -0.5637 | -0.50228 | No | Class I |
| 1947 | 1955 | YKKPASREL | 246.33 | -0.5571 | -0.09661 | No | Class I |
| 1949 | 1957 | KPASRELKV | 119.56 | 0.732 | -0.14448 | No | Class I |
| 1951 | 1959 | ASRELKVTF | 489.07 | 1.29 | -0.03873 | No | Class I |
| 1953 | 1961 | RELKVTFFP | 161.94 | 1.548 | 0.02334 | No | Class I |
| 1959 | 1967 | FFPDLNGDV | 287.14 | -0.6653 | 0.04339 | No | Class I |
| 1960 | 1968 | FPDLNGDVV | 371.33 | -0.0778 | 0.06448 | No | Class I |
| 1967 | 1975 | VVAIDYKHY | 484.68 | 2.1899 | 0.00164 | No | Class I |
| 1971 | 1979 | DYKHYTPSF | 370.23 | 1.3089 | -0.11053 | No | Class I |
| 1978 | 1986 | SFKKGAKLL | 74.67 | 0.5431 | -0.40565 | No | Class I |
| 1984 | 1992 | KLLHKPIVW | 29.17 | 0.3258 | -0.05505 | No | Class I |
| 1996 | 2004 | NATNKATYK | 62.44 | 0.3363 | -0.13648 | No | Class I |
| 2000 | 2008 | KATYKPNTW | 45.74 | 0.7551 | -0.19434 | No | Class I |
| 2006 | 2014 | NTWCIRCLW | 142.32 | 1.7684 | 0.14399 | No | Class I |
| 2009 | 2017 | CIRCLWSTK | 125.43 | 1.757 | 0.04332 | No | Class I |
| 2017 | 2025 | KPVETSNSF | 34.44 | 0.7666 | -0.1059 | No | Class I |
| 2021 | 2029 | TSNSFDVLK | 97.15 | 0.2756 | -0.00533 | No | Class I |
| 2039 | 2047 | LACEDLKPV | 285.67 | 1.57 | -0.09407 | No | Class I |
| 2051 | 2059 | VVENPTIQK | 261.69 | -0.6165 | 0.09637 | No | Class I |
| 2054 | 2062 | NPTIQKDVL | 421.82 | -0.7694 | -0.12644 | No | Class I |
| 2067 | 2075 | KTTEVVGDI | 297.15 | 0.4517 | 0.23397 | No | Class I |
| 2069 | 2077 | TEVVGDIIL | 17.95 | -0.3121 | 0.2989 | No | Class I |
| 2070 | 2078 | EVVGDIILK | 324.27 | 0.1445 | 0.30022 | No | Class I |
| 2076 | 2084 | ILKPANNSL | 57.09 | 0.0739 | -0.15127 | No | Class I |
| 2078 | 2086 | KPANNSLKI | 112.53 | 0.7019 | -0.2912 | No | Class I |
| 2087 | 2095 | TEEVGHTDL | 127.11 | 0.9435 | 0.18321 | No | Class I |
| 2088 | 2096 | EEVGHTDLM | 439.59 | 0.8173 | 0.12778 | No | Class I |
| 2091 | 2099 | GHTDLMAAY | 124.42 | 0.3899 | -0.0853 | No | Class I |
| 2092 | 2100 | HTDLMAAYV | 121.98 | 0.4101 | -0.10727 | No | Class I |
| 2095 | 2103 | LMAAYVDNS | 224.73 | 0.3771 | 0.10227 | No | Class I |
| 2097 | 2105 | AAYVDNSSL | 166.51 | 0.444 | -0.18043 | No | Class I |
| 2099 | 2107 | YVDNSSLTI | 414.11 | 0.6729 | -0.30282 | No | Class I |
| 2101 | 2109 | DNSSLTIKK | 302.89 | 1.0522 | -0.20811 | No | Class I |
| 2105 | 2113 | LTIKKPNEL | 87.95 | 0.7488 | -0.3412 | No | Class I |
| 2109 | 2117 | KPNELSRVL | 8.84 | 0.5252 | -0.00008 | No | Class I |
| 2111 | 2119 | NELSRVLGL | 377.07 | -0.0656 | -0.07037 | No | Class I |
| 2112 | 2120 | ELSRVLGLK | 445.28 | 0.0503 | 0.05026 | No | Class I |
| 2114 | 2122 | SRVLGLKTL | 247.63 | 0.1126 | -0.13452 | No | Class I |
| 2115 | 2123 | RVLGLKTLA | 136.03 | 0.1066 | -0.15702 | No | Class I |
| 2120 | 2128 | KTLATHGLA | 17.55 | -0.2092 | 0.12614 | No | Class I |
| 2121 | 2129 | TLATHGLAA | 38.08 | 0.1587 | 0.12866 | No | Class I |
| 2122 | 2130 | LATHGLAAV | 115.54 | 0.2607 | 0.12359 | No | Class I |
| 2127 | 2135 | LAAVNSVPW | 253.83 | 1.0786 | -0.07943 | No | Class I |
| 2133 | 2141 | VPWDTIANY | 194.85 | 0.1479 | 0.28654 | No | Class I |
| 2137 | 2145 | TIANYAKPF | 191.59 | 0.158 | -0.14906 | No | Class I |
| 2138 | 2146 | IANYAKPFL | 273.29 | -0.1405 | -0.11168 | No | Class I |
| 2140 | 2148 | NYAKPFLNK | 468.09 | -1.0386 | -0.11804 | No | Class I |
| 2141 | 2149 | YAKPFLNKV | 69.24 | -0.9308 | -0.10906 | No | Class I |
| 2145 | 2153 | FLNKVVSTT | 83.03 | 0.1652 | -0.25698 | No | Class I |
| 2148 | 2156 | KVVSTTTNI | 162.42 | 0.6207 | -0.04975 | No | Class I |
| 2149 | 2157 | VVSTTTNIV | 317.71 | 0.6946 | 0.132 | No | Class I |
| 2151 | 2159 | STTTNIVTR | 106.4 | 0.1489 | 0.22816 | No | Class I |
| 2155 | 2163 | NIVTRCLNR | 445.01 | -0.079 | 0.03897 | No | Class I |
| 2156 | 2164 | IVTRCLNRV | 310.23 | 0.0755 | 0.02652 | No | Class I |
| 2157 | 2165 | VTRCLNRVC | 465.33 | -0.3801 | 0.01346 | No | Class I |
| 2160 | 2168 | CLNRVCTNY | 336.71 | -0.4118 | 0.06841 | No | Class I |
| 2163 | 2171 | RVCTNYMPY | 184.91 | -0.3982 | -0.1429 | No | Class I |
| 2165 | 2173 | CTNYMPYFF | 277.27 | 0.3172 | -0.12198 | No | Class I |
| 2169 | 2177 | MPYFFTLLL | 416.18 | 0.4915 | 0.2513 | No | Class I |
| 2171 | 2179 | YFFTLLLQL | 155.86 | 0.3242 | -0.02122 | No | Class I |
| 2174 | 2182 | TLLLQLCTF | 198.75 | -0.7271 | -0.16082 | No | Class I |
| 2175 | 2183 | LLLQLCTFT | 203.78 | -0.6457 | -0.08055 | No | Class I |
| 2176 | 2184 | LLQLCTFTR | 196.24 | -0.6957 | 0.05676 | No | Class I |
| 2181 | 2189 | TFTRSTNSR | 265.27 | -0.3613 | -0.162 | No | Class I |
| 2182 | 2190 | FTRSTNSRI | 201.04 | -0.3988 | -0.22734 | No | Class I |
| 2184 | 2192 | RSTNSRIKA | 49.08 | -0.027 | -0.11997 | No | Class I |
| 2185 | 2193 | STNSRIKAS | 426.93 | 0.5841 | -0.15203 | No | Class I |
| 2189 | 2197 | RIKASMPTT | 10.46 | 0.2197 | -0.34371 | No | Class I |
| 2191 | 2199 | KASMPTTIA | 146.47 | 0.4192 | -0.09414 | No | Class I |
| 2192 | 2200 | ASMPTTIAK | 29.16 | 0.3643 | 0.14136 | No | Class I |
| 2194 | 2202 | MPTTIAKNT | 294.23 | 0.5046 | 0.03231 | No | Class I |
| 2196 | 2204 | TTIAKNTVK | 368.93 | 0.054 | -0.07664 | No | Class I |
| 2198 | 2206 | IAKNTVKSV | 33.2 | 0.2277 | -0.27851 | No | Class I |
| 2201 | 2209 | NTVKSVGKF | 50.83 | -0.0442 | -0.42324 | No | Class I |
| 2205 | 2213 | SVGKFCLEA | 481.56 | 0.5914 | -0.09361 | No | Class I |
| 2209 | 2217 | FCLEASFNY | 81.66 | 1.5042 | 0.07454 | No | Class I |
| 2210 | 2218 | CLEASFNYL | 278.93 | 1.2307 | 0.01335 | No | Class I |
| 2211 | 2219 | LEASFNYLK | 326.25 | 0.7711 | -0.05546 | No | Class I |
| 2215 | 2223 | FNYLKSPNF | 269.53 | 0.6743 | -0.39123 | No | Class I |
| 2217 | 2225 | YLKSPNFSK | 47.92 | 0.346 | -0.25122 | No | Class I |
| 2219 | 2227 | KSPNFSKLI | 359.54 | 0.4503 | -0.24032 | No | Class I |
| 2222 | 2230 | NFSKLINII | 321.72 | 0.1171 | -0.08392 | No | Class I |
| 2223 | 2231 | FSKLINIII | 247.98 | -0.09 | 0.23243 | No | Class I |
| 2225 | 2233 | KLINIIIWF | 161.41 | 0.1019 | 0.53331 | No | Class I |
| 2226 | 2234 | LINIIIWFL | 335.02 | -0.1505 | 0.64204 | No | Class I |
| 2234 | 2242 | LLLSVCLGS | 462.49 | 0.3205 | -0.17018 | No | Class I |
| 2235 | 2243 | LLSVCLGSL | 128.94 | 0.5685 | -0.14316 | No | Class I |
| 2236 | 2244 | LSVCLGSLI | 36.2 | 0.1423 | -0.16585 | No | Class I |
| 2237 | 2245 | SVCLGSLIY | 185.67 | 0.5739 | -0.08299 | No | Class I |
| 2239 | 2247 | CLGSLIYST | 274.01 | 0.4013 | -0.14077 | No | Class I |
| 2242 | 2250 | SLIYSTAAL | 42.67 | 0.452 | -0.0292 | No | Class I |
| 2244 | 2252 | IYSTAALGV | 114.42 | 0.6815 | 0.07073 | No | Class I |
| 2246 | 2254 | STAALGVLM | 463.02 | 0.4796 | 0.10153 | No | Class I |
| 2249 | 2257 | ALGVLMSNL | 471.52 | 0.6985 | -0.26696 | No | Class I |
| 2251 | 2259 | GVLMSNLGM | 415.08 | 0.9934 | -0.33705 | No | Class I |
| 2253 | 2261 | LMSNLGMPS | 185.37 | 0.3607 | -0.19379 | No | Class I |
| 2254 | 2262 | MSNLGMPSY | 145.76 | 0.9272 | -0.25158 | No | Class I |
| 2258 | 2266 | GMPSYCTGY | 151.5 | -0.0156 | -0.17186 | No | Class I |
| 2259 | 2267 | MPSYCTGYR | 285.07 | -0.2531 | -0.04694 | No | Class I |
| 2270 | 2278 | YLNSTNVTI | 12.5 | 1.1357 | -0.07934 | No | Class I |
| 2273 | 2281 | STNVTIATY | 234.54 | 0.7143 | 0.25822 | No | Class I |
| 2278 | 2286 | IATYCTGSI | 250.69 | -0.095 | -0.07514 | No | Class I |
| 2282 | 2290 | CTGSIPCSV | 478.71 | -0.0478 | -0.17847 | No | Class I |
| 2284 | 2292 | GSIPCSVCL | 290.81 | 0.41 | -0.17285 | No | Class I |
| 2299 | 2307 | DTYPSLETI | 471.59 | 0.7144 | -0.07672 | No | Class I |
| 2301 | 2309 | YPSLETIQI | 188.04 | 0.8151 | 0.11382 | No | Class I |
| 2305 | 2313 | ETIQITISS | 317.25 | 0.8045 | 0.10844 | No | Class I |
| 2306 | 2314 | TIQITISSF | 88.11 | 0.8518 | 0.02312 | No | Class I |
| 2307 | 2315 | IQITISSFK | 64.52 | 1.0272 | -0.01509 | No | Class I |
| 2308 | 2316 | QITISSFKW | 191.89 | 1.0201 | -0.19751 | No | Class I |
| 2312 | 2320 | SSFKWDLTA | 364.84 | 1.003 | 0.0709 | No | Class I |
| 2313 | 2321 | SFKWDLTAF | 430.09 | 1.0315 | 0.21967 | No | Class I |
| 2315 | 2323 | KWDLTAFGL | 161.36 | 1.3866 | 0.18927 | No | Class I |
| 2316 | 2324 | WDLTAFGLV | 295.21 | 1.5956 | 0.20588 | No | Class I |
| 2319 | 2327 | TAFGLVAEW | 354.11 | 1.0431 | 0.19168 | No | Class I |
| 2320 | 2328 | AFGLVAEWF | 51.66 | 0.5386 | 0.29079 | No | Class I |
| 2321 | 2329 | FGLVAEWFL | 278.86 | 0.5377 | 0.42563 | No | Class I |
| 2322 | 2330 | GLVAEWFLA | 52.09 | 0.0424 | 0.4511 | No | Class I |
| 2323 | 2331 | LVAEWFLAY | 300.92 | -0.1674 | 0.45285 | No | Class I |
| 2325 | 2333 | AEWFLAYIL | 150.1 | 0.0481 | 0.29037 | No | Class I |
| 2326 | 2334 | EWFLAYILF | 275.43 | 0.2753 | 0.1673 | No | Class I |
| 2328 | 2336 | FLAYILFTR | 237.63 | 0.5335 | 0.24962 | No | Class I |
| 2329 | 2337 | LAYILFTRF | 211.37 | 0.5081 | 0.29512 | No | Class I |
| 2330 | 2338 | AYILFTRFF | 35.14 | -0.3147 | 0.29466 | No | Class I |
| 2331 | 2339 | YILFTRFFY | 260.12 | -0.0847 | 0.36792 | No | Class I |
| 2332 | 2340 | ILFTRFFYV | 2.32 | 0.2566 | 0.3343 | No | Class I |
| 2333 | 2341 | LFTRFFYVL | 363.97 | -0.3384 | 0.30988 | No | Class I |
| 2334 | 2342 | FTRFFYVLG | 165.46 | -0.2079 | 0.27348 | No | Class I |
| 2335 | 2343 | TRFFYVLGL | 135.82 | 0.5606 | 0.2015 | No | Class I |
| 2336 | 2344 | RFFYVLGLA | 165.85 | 0.665 | 0.08616 | No | Class I |
| 2338 | 2346 | FYVLGLAAI | 233.82 | 1.0643 | 0.08068 | No | Class I |
| 2339 | 2347 | YVLGLAAIM | 92.76 | 0.6002 | 0.16731 | No | Class I |
| 2342 | 2350 | GLAAIMQLF | 461.31 | 0.0254 | -0.08787 | No | Class I |
| 2343 | 2351 | LAAIMQLFF | 434.39 | 0.0732 | -0.07438 | No | Class I |
| 2345 | 2353 | AIMQLFFSY | 207.12 | 0.0447 | -0.07202 | No | Class I |
| 2346 | 2354 | IMQLFFSYF | 243.6 | -0.2812 | 0.03366 | No | Class I |
| 2347 | 2355 | MQLFFSYFA | 18.7 | 0.0581 | 0.13775 | No | Class I |
| 2348 | 2356 | QLFFSYFAV | 7.15 | 0.2473 | 0.11288 | No | Class I |
| 2349 | 2357 | LFFSYFAVH | 392.25 | 0.4368 | 0.03527 | No | Class I |
| 2350 | 2358 | FFSYFAVHF | 348.15 | 0.5568 | 0.14715 | No | Class I |
| 2351 | 2359 | FSYFAVHFI | 76.01 | 0.8806 | 0.28926 | No | Class I |
| 2354 | 2362 | FAVHFISNS | 492.94 | 0.23 | 0.14183 | No | Class I |
| 2355 | 2363 | AVHFISNSW | 26.49 | 0.3022 | 0.00005 | No | Class I |
| 2356 | 2364 | VHFISNSWL | 376.94 | 0.2617 | -0.00547 | No | Class I |
| 2357 | 2365 | HFISNSWLM | 81.06 | 0.0245 | -0.10484 | No | Class I |
| 2358 | 2366 | FISNSWLMW | 313.48 | 0.3139 | -0.12476 | No | Class I |
| 2359 | 2367 | ISNSWLMWL | 361.11 | 0.2917 | 0.01791 | No | Class I |
| 2366 | 2374 | WLIINLVQM | 238.22 | 0.9119 | 0.12754 | No | Class I |
| 2367 | 2375 | LIINLVQMA | 106.64 | 0.6135 | -0.13561 | No | Class I |
| 2372 | 2380 | VQMAPISAM | 151.95 | 1.2268 | -0.01991 | No | Class I |
| 2373 | 2381 | QMAPISAMV | 16.04 | 0.8402 | -0.09417 | No | Class I |
| 2374 | 2382 | MAPISAMVR | 270.8 | 0.6547 | -0.11803 | No | Class I |
| 2375 | 2383 | APISAMVRM | 116.92 | 0.4496 | -0.18539 | No | Class I |
| 2377 | 2385 | ISAMVRMYI | 433.76 | -0.0324 | -0.22544 | No | Class I |
| 2378 | 2386 | SAMVRMYIF | 479.67 | -0.3203 | -0.05572 | No | Class I |
| 2379 | 2387 | AMVRMYIFF | 456.75 | 0.0931 | 0.07172 | No | Class I |
| 2380 | 2388 | MVRMYIFFA | 188.49 | 0.159 | 0.12898 | No | Class I |
| 2382 | 2390 | RMYIFFASF | 230.42 | 0.2317 | 0.29328 | No | Class I |
| 2383 | 2391 | MYIFFASFY | 165.83 | 0.0576 | 0.24061 | No | Class I |
| 2384 | 2392 | YIFFASFYY | 62.66 | 0.3186 | 0.13481 | No | Class I |
| 2385 | 2393 | IFFASFYYV | 95.15 | 0.3613 | 0.02119 | No | Class I |
| 2386 | 2394 | FFASFYYVW | 55.85 | -0.0413 | -0.02225 | No | Class I |
| 2387 | 2395 | FASFYYVWK | 490.08 | 0.1684 | 0.22128 | No | Class I |
| 2389 | 2397 | SFYYVWKSY | 15.42 | 0.482 | -0.03487 | No | Class I |
| 2390 | 2398 | FYYVWKSYV | 54.44 | 0.0662 | -0.08874 | No | Class I |
| 2391 | 2399 | YYVWKSYVH | 393.8 | -0.3785 | -0.10844 | No | Class I |
| 2392 | 2400 | YVWKSYVHV | 14.63 | -0.2955 | -0.25594 | No | Class I |
| 2393 | 2401 | VWKSYVHVV | 116.61 | -0.3587 | -0.14979 | No | Class I |
| 2395 | 2403 | KSYVHVVDG | 324.11 | 0.3247 | 0.1585 | No | Class I |
| 2405 | 2413 | NSSTCMMCY | 59.9 | 0.2538 | -0.41214 | No | Class I |
| 2406 | 2414 | SSTCMMCYK | 77.21 | 1.1941 | -0.42561 | No | Class I |
| 2407 | 2415 | STCMMCYKR | 466.55 | 1.0617 | -0.54507 | No | Class I |
| 2409 | 2417 | CMMCYKRNR | 11.15 | 2.2316 | -0.27795 | No | Class I |
| 2410 | 2418 | MMCYKRNRA | 474.02 | 1.692 | -0.15772 | No | Class I |
| 2412 | 2420 | CYKRNRATR | 53.38 | 0.1562 | 0.0802 | No | Class I |
| 2413 | 2421 | YKRNRATRV | 436.93 | -0.4027 | 0.16052 | No | Class I |
| 2415 | 2423 | RNRATRVEC | 195.96 | 0.1542 | 0.23603 | No | Class I |
| 2418 | 2426 | ATRVECTTI | 75.72 | 1.0578 | 0.16053 | No | Class I |
| 2419 | 2427 | TRVECTTIV | 432.79 | 1.1007 | 0.20871 | No | Class I |
| 2423 | 2431 | CTTIVNGVR | 251.47 | 0.4645 | 0.23335 | No | Class I |
| 2424 | 2432 | TTIVNGVRR | 417.37 | 0.1398 | 0.17542 | No | Class I |
| 2426 | 2434 | IVNGVRRSF | 307.28 | 0.2315 | 0.06794 | No | Class I |
| 2429 | 2437 | GVRRSFYVY | 370.69 | 0.1829 | 0.03898 | No | Class I |
| 2430 | 2438 | VRRSFYVYA | 298.78 | 0.4015 | -0.00647 | No | Class I |
| 2432 | 2440 | RSFYVYANG | 62.86 | -0.5728 | 0.10024 | No | Class I |
| 2436 | 2444 | VYANGGKGF | 375.94 | -0.1477 | -0.09111 | No | Class I |
| 2442 | 2450 | KGFCKLHNW | 164.41 | 0.4274 | -0.21317 | No | Class I |
| 2456 | 2464 | DTFCAGSTF | 75.52 | 0.0675 | -0.06319 | No | Class I |
| 2457 | 2465 | TFCAGSTFI | 352.12 | -0.0857 | 0.0003 | No | Class I |
| 2463 | 2471 | TFISDEVAR | 470.19 | -0.507 | 0.05028 | No | Class I |
| 2465 | 2473 | ISDEVARDL | 410.89 | -0.4247 | 0.24162 | No | Class I |
| 2467 | 2475 | DEVARDLSL | 284.7 | 0.1359 | 0.01803 | No | Class I |
| 2469 | 2477 | VARDLSLQF | 174.07 | 1.0239 | -0.20445 | No | Class I |
| 2473 | 2481 | LSLQFKRPI | 91.9 | 0.8992 | -0.17196 | No | Class I |
| 2488 | 2496 | SYIVDSVTV | 279.07 | 0.2816 | 0.00813 | No | Class I |
| 2489 | 2497 | YIVDSVTVK | 210.13 | 0.6881 | -0.02964 | No | Class I |
| 2499 | 2507 | GSIHLYFDK | 357.55 | 0.1725 | 0.17323 | No | Class I |
| 2500 | 2508 | SIHLYFDKA | 430.6 | 0.1874 | -0.00134 | No | Class I |
| 2507 | 2515 | KAGQKTYER | 70.17 | 0.6215 | -0.22364 | No | Class I |
| 2512 | 2520 | TYERHSLSH | 270.35 | 0.4098 | -0.14567 | No | Class I |
| 2513 | 2521 | YERHSLSHF | 169.02 | 0.3781 | -0.24291 | No | Class I |
| 2514 | 2522 | ERHSLSHFV | 290.29 | -0.0045 | -0.2268 | No | Class I |
| 2516 | 2524 | HSLSHFVNL | 361.53 | 0.2198 | 0.00269 | No | Class I |
| 2519 | 2527 | SHFVNLDNL | 253.84 | 0.8728 | 0.07774 | No | Class I |
| 2520 | 2528 | HFVNLDNLR | 112.44 | 0.9687 | 0.00503 | No | Class I |
| 2528 | 2536 | RANNTKGSL | 88.48 | 0.2941 | -0.24187 | No | Class I |
| 2534 | 2542 | GSLPINVIV | 307.74 | 0.1289 | 0.22135 | No | Class I |
| 2535 | 2543 | SLPINVIVF | 135.95 | -0.0762 | 0.29932 | No | Class I |
| 2546 | 2554 | KSKCEESSA | 98.07 | 0.6568 | -0.16878 | No | Class I |
| 2552 | 2560 | SSAKSASVY | 345.77 | 0.2713 | -0.44407 | No | Class I |
| 2553 | 2561 | SAKSASVYY | 279.1 | 0.5808 | -0.32142 | No | Class I |
| 2556 | 2564 | SASVYYSQL | 287.34 | 0.4664 | -0.22654 | No | Class I |
| 2557 | 2565 | ASVYYSQLM | 9.01 | 0.3442 | -0.25389 | No | Class I |
| 2561 | 2569 | YSQLMCQPI | 405.98 | 0.746 | -0.37475 | No | Class I |
| 2562 | 2570 | SQLMCQPIL | 104.72 | 0.428 | -0.27344 | No | Class I |
| 2563 | 2571 | QLMCQPILL | 13.21 | 0.733 | -0.12865 | No | Class I |
| 2564 | 2572 | LMCQPILLL | 136.64 | 0.4704 | -0.03542 | No | Class I |
| 2567 | 2575 | QPILLLDQA | 57.04 | 0.4044 | -0.03816 | No | Class I |
| 2569 | 2577 | ILLLDQALV | 17.57 | 0.3804 | -0.07566 | No | Class I |
| 2582 | 2590 | DSAEVAVKM | 485.25 | 0.7389 | 0.09932 | No | Class I |
| 2583 | 2591 | SAEVAVKMF | 494.52 | 0.1802 | -0.1336 | No | Class I |
| 2586 | 2594 | VAVKMFDAY | 355.47 | 0.274 | -0.22282 | No | Class I |
| 2587 | 2595 | AVKMFDAYV | 47.27 | -0.1997 | -0.08096 | No | Class I |
| 2589 | 2597 | KMFDAYVNT | 34.86 | -0.5665 | 0.126 | No | Class I |
| 2590 | 2598 | MFDAYVNTF | 127.29 | -0.9601 | 0.09905 | No | Class I |
| 2593 | 2601 | AYVNTFSST | 241.94 | -0.3809 | -0.08139 | No | Class I |
| 2594 | 2602 | YVNTFSSTF | 91.1 | -0.6245 | -0.12171 | No | Class I |
| 2596 | 2604 | NTFSSTFNV | 53.97 | -0.1846 | -0.15801 | No | Class I |
| 2598 | 2606 | FSSTFNVPM | 105.82 | -0.2191 | 0.12163 | No | Class I |
| 2600 | 2608 | STFNVPMEK | 11.64 | -0.3819 | -0.02845 | No | Class I |
| 2601 | 2609 | TFNVPMEKL | 447.48 | -0.5804 | -0.17816 | No | Class I |
| 2604 | 2612 | VPMEKLKTL | 16.5 | 0.3195 | -0.33601 | No | Class I |
| 2606 | 2614 | MEKLKTLVA | 263.32 | -0.2326 | -0.23986 | No | Class I |
| 2608 | 2616 | KLKTLVATA | 15.39 | 0.1977 | 0.05282 | No | Class I |
| 2610 | 2618 | KTLVATAEA | 378.91 | 0.1907 | 0.2041 | No | Class I |
| 2612 | 2620 | LVATAEAEL | 411.64 | 0.7661 | 0.27563 | No | Class I |
| 2614 | 2622 | ATAEAELAK | 272.1 | 0.0237 | 0.2593 | No | Class I |
| 2616 | 2624 | AEAELAKNV | 312.93 | -0.2254 | -0.0463 | No | Class I |
| 2618 | 2626 | AELAKNVSL | 222.77 | 0.4238 | -0.24214 | No | Class I |
| 2625 | 2633 | SLDNVLSTF | 370.05 | 0.1009 | -0.08649 | No | Class I |
| 2626 | 2634 | LDNVLSTFI | 415.39 | -0.2496 | -0.02593 | No | Class I |
| 2628 | 2636 | NVLSTFISA | 60.37 | -0.2514 | -0.00641 | No | Class I |
| 2629 | 2637 | VLSTFISAA | 61.11 | -0.0885 | 0.10788 | No | Class I |
| 2630 | 2638 | LSTFISAAR | 354.92 | -0.0338 | 0.16015 | No | Class I |
| 2633 | 2641 | FISAARQGF | 99.36 | 0.8197 | -0.00547 | No | Class I |
| 2634 | 2642 | ISAARQGFV | 493.55 | 0.7315 | 0.09043 | No | Class I |
| 2636 | 2644 | AARQGFVDS | 498.32 | 0.4589 | 0.09124 | No | Class I |
| 2669 | 2677 | SCNNYMLTY | 158 | 0.0653 | -0.16419 | No | Class I |
| 2671 | 2679 | NNYMLTYNK | 77.74 | 0.3554 | -0.15906 | No | Class I |
| 2672 | 2680 | NYMLTYNKV | 125.82 | 0.3583 | -0.1653 | No | Class I |
| 2675 | 2683 | LTYNKVENM | 15.93 | 0.6724 | -0.09813 | No | Class I |
| 2680 | 2688 | VENMTPRDL | 434.01 | 0.8575 | -0.0948 | No | Class I |
| 2695 | 2703 | SARHINAQV | 63.45 | 0.516 | 0.1382 | No | Class I |
| 2701 | 2709 | AQVAKSHNI | 228.46 | 0.6477 | -0.28944 | No | Class I |
| 2703 | 2711 | VAKSHNIAL | 342.72 | 1.0727 | -0.07588 | No | Class I |
| 2707 | 2715 | HNIALIWNV | 77.06 | 0.7397 | 0.38021 | No | Class I |
| 2708 | 2716 | NIALIWNVK | 329.27 | 1.6835 | 0.35831 | No | Class I |
| 2710 | 2718 | ALIWNVKDF | 452.53 | 1.2837 | 0.12961 | No | Class I |
| 2718 | 2726 | FMSLSEQLR | 240.6 | 0.8171 | -0.23595 | No | Class I |
| 2719 | 2727 | MSLSEQLRK | 120.89 | 0.4894 | -0.16073 | No | Class I |
| 2724 | 2732 | QLRKQIRSA | 452.6 | 0.5377 | -0.2407 | No | Class I |
| 2726 | 2734 | RKQIRSAAK | 368.8 | 0.6153 | 0.04687 | No | Class I |
| 2727 | 2735 | KQIRSAAKK | 128.21 | -0.1757 | -0.12197 | No | Class I |
| 2730 | 2738 | RSAAKKNNL | 185.23 | 0.1755 | -0.37017 | No | Class I |
| 2732 | 2740 | AAKKNNLPF | 88.17 | 1.8342 | -0.31523 | No | Class I |
| 2733 | 2741 | AKKNNLPFK | 101.85 | 1.8424 | -0.03421 | No | Class I |
| 2737 | 2745 | NLPFKLTCA | 229.11 | 2.031 | -0.10498 | No | Class I |
| 2738 | 2746 | LPFKLTCAT | 453.36 | 1.8714 | -0.1759 | No | Class I |
| 2745 | 2753 | ATTRQVVNV | 358.53 | 0.4528 | 0.0218 | No | Class I |
| 2746 | 2754 | TTRQVVNVV | 76.36 | 0.469 | -0.00204 | No | Class I |
| 2749 | 2757 | QVVNVVTTK | 265.25 | 0.8798 | 0.14139 | No | Class I |
| 2750 | 2758 | VVNVVTTKI | 483.92 | 0.4561 | 0.02294 | No | Class I |
| 2753 | 2761 | VVTTKIALK | 84.01 | 1.1143 | -0.00652 | No | Class I |
| 2758 | 2766 | IALKGGKIV | 344.3 | 1.3772 | -0.25994 | No | Class I |
| 2761 | 2769 | KGGKIVNNW | 135.67 | 0.5023 | -0.04678 | No | Class I |
| 2765 | 2773 | IVNNWLKQL | 391.07 | -1.0938 | -0.05303 | No | Class I |
| 2771 | 2779 | KQLIKVTLV | 227.16 | 0.3388 | -0.01454 | No | Class I |
| 2772 | 2780 | QLIKVTLVF | 139.1 | 0.7099 | -0.0823 | No | Class I |
| 2773 | 2781 | LIKVTLVFL | 370 | 0.9869 | 0.10214 | No | Class I |
| 2775 | 2783 | KVTLVFLFV | 138.21 | 0.9591 | 0.21088 | No | Class I |
| 2776 | 2784 | VTLVFLFVA | 297.22 | 0.893 | 0.26442 | No | Class I |
| 2777 | 2785 | TLVFLFVAA | 375.69 | 0.4641 | 0.2883 | No | Class I |
| 2778 | 2786 | LVFLFVAAI | 297.39 | 0.4938 | 0.23558 | No | Class I |
| 2779 | 2787 | VFLFVAAIF | 178.35 | -0.0364 | 0.30201 | No | Class I |
| 2781 | 2789 | LFVAAIFYL | 103.21 | 0.1471 | 0.31279 | No | Class I |
| 2782 | 2790 | FVAAIFYLI | 9.83 | 0.1406 | 0.28227 | No | Class I |
| 2785 | 2793 | AIFYLITPV | 12.49 | 0.1462 | 0.17504 | No | Class I |
| 2786 | 2794 | IFYLITPVH | 313.72 | 0.3338 | 0.16854 | No | Class I |
| 2787 | 2795 | FYLITPVHV | 328.21 | 0.6305 | 0.21142 | No | Class I |
| 2788 | 2796 | YLITPVHVM | 31.75 | 0.3085 | 0.16174 | No | Class I |
| 2790 | 2798 | ITPVHVMSK | 179.5 | 0.968 | -0.13656 | No | Class I |
| 2794 | 2802 | HVMSKHTDF | 192.1 | 1.1597 | -0.3573 | No | Class I |
| 2796 | 2804 | MSKHTDFSS | 295.54 | 1.2214 | 0.02337 | No | Class I |
| 2802 | 2810 | FSSEIIGYK | 171.4 | 0.3312 | 0.32837 | No | Class I |
| 2804 | 2812 | SEIIGYKAI | 59.69 | 0.3416 | 0.0475 | No | Class I |
| 2810 | 2818 | KAIDGGVTR | 83.13 | 0.5705 | 0.18794 | No | Class I |
| 2822 | 2830 | STDTCFANK | 269.02 | 0.7235 | 0.1332 | No | Class I |
| 2826 | 2834 | CFANKHADF | 307.54 | 1.8261 | -0.12738 | No | Class I |
| 2833 | 2841 | DFDTWFSQR | 25.29 | -0.0984 | 0.16486 | No | Class I |
| 2837 | 2845 | WFSQRGGSY | 47.31 | 0.7448 | -0.15602 | No | Class I |
| 2845 | 2853 | YTNDKACPL | 154.64 | 1.2036 | -0.20493 | No | Class I |
| 2849 | 2857 | KACPLIAAV | 321.55 | 1.2271 | 0.1417 | No | Class I |
| 2851 | 2859 | CPLIAAVIT | 234.31 | 0.8732 | 0.31785 | No | Class I |
| 2854 | 2862 | IAAVITREV | 330.22 | 0.7253 | 0.32256 | No | Class I |
| 2856 | 2864 | AVITREVGF | 322.21 | 0.9415 | 0.28155 | No | Class I |
| 2857 | 2865 | VITREVGFV | 288.13 | 1.0099 | 0.29804 | No | Class I |
| 2858 | 2866 | ITREVGFVV | 89.08 | 0.9122 | 0.31257 | No | Class I |
| 2860 | 2868 | REVGFVVPG | 301.29 | 1.2977 | 0.22872 | No | Class I |
| 2864 | 2872 | FVVPGLPGT | 289.86 | 0.8959 | 0.03524 | No | Class I |
| 2865 | 2873 | VVPGLPGTI | 457.35 | 0.7188 | 0.06054 | No | Class I |
| 2866 | 2874 | VPGLPGTIL | 52.36 | 0.1785 | 0.13146 | No | Class I |
| 2868 | 2876 | GLPGTILRT | 360.84 | 0.3116 | 0.21446 | No | Class I |
| 2873 | 2881 | ILRTTNGDF | 105.79 | 1.0037 | 0.12913 | No | Class I |
| 2876 | 2884 | TTNGDFLHF | 342.16 | 0.9569 | 0.17334 | No | Class I |
| 2881 | 2889 | FLHFLPRVF | 30.16 | 0.37 | 0.17486 | No | Class I |
| 2883 | 2891 | HFLPRVFSA | 274.86 | 0.2422 | 0.07664 | No | Class I |
| 2884 | 2892 | FLPRVFSAV | 5.52 | 0.1817 | 0.08212 | No | Class I |
| 2885 | 2893 | LPRVFSAVG | 162.48 | 0.0961 | 0.07375 | No | Class I |
| 2887 | 2895 | RVFSAVGNI | 99.77 | 0.0444 | -0.02669 | No | Class I |
| 2893 | 2901 | GNICYTPSK | 485.26 | 1.8425 | -0.08413 | No | Class I |
| 2897 | 2905 | YTPSKLIEY | 355.25 | 0.1688 | -0.21969 | No | Class I |
| 2907 | 2915 | DFATSACVL | 251.07 | 0.1343 | -0.09389 | No | Class I |
| 2908 | 2916 | FATSACVLA | 186.26 | -0.0131 | -0.13816 | No | Class I |
| 2909 | 2917 | ATSACVLAA | 318.53 | 0.5329 | -0.01447 | No | Class I |
| 2914 | 2922 | VLAAECTIF | 35.23 | 0.2785 | 0.20934 | No | Class I |
| 2915 | 2923 | LAAECTIFK | 295.71 | -0.1329 | 0.27821 | No | Class I |
| 2917 | 2925 | AECTIFKDA | 334.2 | -0.6772 | 0.09232 | No | Class I |
| 2920 | 2928 | TIFKDASGK | 92.45 | -1.5175 | -0.24039 | No | Class I |
| 2922 | 2930 | FKDASGKPV | 472.42 | -0.4429 | -0.27111 | No | Class I |
| 2924 | 2932 | DASGKPVPY | 106.25 | 0.698 | -0.21168 | No | Class I |
| 2930 | 2938 | VPYCYDTNV | 203.58 | 0.4732 | -0.00919 | No | Class I |
| 2937 | 2945 | NVLEGSVAY | 387.51 | 0.3194 | 0.03212 | No | Class I |
| 2941 | 2949 | GSVAYESLR | 427.09 | 0.8669 | -0.00268 | No | Class I |
| 2947 | 2955 | SLRPDTRYV | 445.1 | 0.8157 | 0.1053 | No | Class I |
| 2948 | 2956 | LRPDTRYVL | 407.97 | 0.3961 | 0.12624 | No | Class I |
| 2949 | 2957 | RPDTRYVLM | 33.52 | 0.352 | 0.12154 | No | Class I |
| 2953 | 2961 | RYVLMDGSI | 263.42 | 0.0586 | -0.21594 | No | Class I |
| 2954 | 2962 | YVLMDGSII | 424.14 | -0.0793 | -0.18866 | No | Class I |
| 2956 | 2964 | LMDGSIIQF | 464.02 | 0.0194 | 0.05012 | No | Class I |
| 2960 | 2968 | SIIQFPNTY | 343.37 | -0.0617 | 0.04742 | No | Class I |
| 2961 | 2969 | IIQFPNTYL | 364.52 | -0.1364 | 0.09391 | No | Class I |
| 2964 | 2972 | FPNTYLEGS | 96.56 | -0.1939 | 0.12722 | No | Class I |
| 2966 | 2974 | NTYLEGSVR | 492.97 | -0.1099 | 0.00154 | No | Class I |
| 2967 | 2975 | TYLEGSVRV | 483.46 | -0.0518 | 0.0395 | No | Class I |
| 2968 | 2976 | YLEGSVRVV | 224.05 | 0.0937 | 0.01216 | No | Class I |
| 2971 | 2979 | GSVRVVTTF | 106.73 | 0.0209 | 0.19998 | No | Class I |
| 2972 | 2980 | SVRVVTTFD | 276.93 | 0.6548 | 0.23624 | No | Class I |
| 2975 | 2983 | VVTTFDSEY | 81.42 | 0.4588 | 0.10542 | No | Class I |
| 2977 | 2985 | TTFDSEYCR | 131.92 | 0.0307 | -0.04115 | No | Class I |
| 2981 | 2989 | SEYCRHGTC | 418.93 | -0.0254 | 0.07668 | No | Class I |
| 2992 | 3000 | SEAGVCVST | 172.5 | 0.3441 | -0.02557 | No | Class I |
| 2995 | 3003 | GVCVSTSGR | 176.67 | 0.6131 | -0.22034 | No | Class I |
| 2996 | 3004 | VCVSTSGRW | 249.03 | 0.6413 | -0.21216 | No | Class I |
| 2998 | 3006 | VSTSGRWVL | 64.63 | 0.4843 | 0.13891 | No | Class I |
| 3003 | 3011 | RWVLNNDYY | 406.25 | -0.3626 | 0.00641 | No | Class I |
| 3004 | 3012 | WVLNNDYYR | 33.26 | -0.455 | -0.00081 | No | Class I |
| 3010 | 3018 | YYRSLPGVF | 111.47 | 0.1672 | -0.11819 | No | Class I |
| 3012 | 3020 | RSLPGVFCG | 336.16 | 0.052 | 0.1244 | No | Class I |
| 3013 | 3021 | SLPGVFCGV | 7.48 | 0.116 | 0.1552 | No | Class I |
| 3022 | 3030 | DAVNLLTNM | 234.29 | -0.1295 | 0.01463 | No | Class I |
| 3023 | 3031 | AVNLLTNMF | 250.46 | -0.5873 | -0.09558 | No | Class I |
| 3026 | 3034 | LLTNMFTPL | 16.66 | -0.5248 | -0.02843 | No | Class I |
| 3027 | 3035 | LTNMFTPLI | 448.16 | -0.3922 | -0.0441 | No | Class I |
| 3030 | 3038 | MFTPLIQPI | 398.84 | -0.0273 | 0.01168 | No | Class I |
| 3032 | 3040 | TPLIQPIGA | 75.91 | -0.0712 | 0.1392 | No | Class I |
| 3039 | 3047 | GALDISASI | 187.26 | 1.0215 | -0.07105 | No | Class I |
| 3040 | 3048 | ALDISASIV | 480.95 | 1.0373 | -0.04501 | No | Class I |
| 3047 | 3055 | IVAGGIVAI | 120.78 | 0.7674 | 0.26278 | No | Class I |
| 3048 | 3056 | VAGGIVAIV | 138.63 | 0.5099 | 0.32434 | No | Class I |
| 3053 | 3061 | VAIVVTCLA | 396.71 | 0.9759 | 0.1095 | No | Class I |
| 3054 | 3062 | AIVVTCLAY | 167.85 | 1.0125 | 0.05549 | No | Class I |
| 3055 | 3063 | IVVTCLAYY | 455.58 | 0.7728 | 0.02038 | No | Class I |
| 3056 | 3064 | VVTCLAYYF | 273.9 | 0.2441 | -0.0209 | No | Class I |
| 3057 | 3065 | VTCLAYYFM | 195.78 | 0.6534 | 0.07124 | No | Class I |
| 3058 | 3066 | TCLAYYFMR | 198.84 | 0.2985 | 0.02489 | No | Class I |
| 3059 | 3067 | CLAYYFMRF | 469.27 | 0.2746 | -0.00238 | No | Class I |
| 3060 | 3068 | LAYYFMRFR | 217.84 | 0.5648 | 0.05586 | No | Class I |
| 3061 | 3069 | AYYFMRFRR | 265.82 | 0.3577 | 0.12336 | No | Class I |
| 3062 | 3070 | YYFMRFRRA | 359.54 | 0.2477 | 0.09582 | No | Class I |
| 3063 | 3071 | YFMRFRRAF | 154.53 | -0.0952 | 0.22434 | No | Class I |
| 3064 | 3072 | FMRFRRAFG | 331.78 | -0.3698 | 0.33514 | No | Class I |
| 3066 | 3074 | RFRRAFGEY | 41.33 | -1.0034 | 0.30428 | No | Class I |
| 3069 | 3077 | RAFGEYSHV | 130.17 | -0.0735 | 0.0454 | No | Class I |
| 3070 | 3078 | AFGEYSHVV | 198.95 | 0.6737 | 0.00384 | No | Class I |
| 3072 | 3080 | GEYSHVVAF | 313.05 | 0.6428 | -0.03961 | No | Class I |
| 3075 | 3083 | SHVVAFNTL | 326.67 | 0.0773 | 0.22046 | No | Class I |
| 3076 | 3084 | HVVAFNTLL | 402.25 | -0.1586 | 0.18696 | No | Class I |
| 3077 | 3085 | VVAFNTLLF | 181.09 | -0.0121 | 0.1449 | No | Class I |
| 3078 | 3086 | VAFNTLLFL | 406.46 | 0.2353 | 0.11789 | No | Class I |
| 3079 | 3087 | AFNTLLFLM | 242.51 | 0.0288 | 0.10804 | No | Class I |
| 3081 | 3089 | NTLLFLMSF | 346.7 | 0.0075 | -0.15606 | No | Class I |
| 3082 | 3090 | TLLFLMSFT | 239.61 | 0.4399 | -0.13312 | No | Class I |
| 3083 | 3091 | LLFLMSFTV | 9.96 | 0.5211 | -0.17841 | No | Class I |
| 3084 | 3092 | LFLMSFTVL | 122.49 | 0.4537 | -0.17432 | No | Class I |
| 3085 | 3093 | FLMSFTVLC | 70.8 | 1.0032 | -0.04457 | No | Class I |
| 3087 | 3095 | MSFTVLCLT | 239.67 | 2.2251 | 0.05484 | No | Class I |
| 3089 | 3097 | FTVLCLTPV | 25.84 | 2.1714 | -0.03442 | No | Class I |
| 3090 | 3098 | TVLCLTPVY | 454.44 | 1.7229 | -0.01735 | No | Class I |
| 3093 | 3101 | CLTPVYSFL | 69.07 | 0.5139 | -0.03306 | No | Class I |
| 3095 | 3103 | TPVYSFLPG | 380.52 | -0.3471 | -0.05706 | No | Class I |
| 3096 | 3104 | PVYSFLPGV | 236.98 | -0.0332 | -0.05367 | No | Class I |
| 3097 | 3105 | VYSFLPGVY | 340.8 | 0.1176 | 0.09558 | No | Class I |
| 3099 | 3107 | SFLPGVYSV | 281.29 | 0.8097 | -0.04268 | No | Class I |
| 3100 | 3108 | FLPGVYSVI | 30.71 | 0.3538 | -0.04828 | No | Class I |
| 3101 | 3109 | LPGVYSVIY | 268.52 | 0.1811 | 0.00581 | No | Class I |
| 3103 | 3111 | GVYSVIYLY | 158.58 | 0.2697 | -0.01179 | No | Class I |
| 3104 | 3112 | VYSVIYLYL | 88.1 | 0.2783 | 0.10244 | No | Class I |
| 3105 | 3113 | YSVIYLYLT | 294.18 | 0.7896 | 0.12368 | No | Class I |
| 3106 | 3114 | SVIYLYLTF | 483.89 | 1.0482 | 0.03852 | No | Class I |
| 3107 | 3115 | VIYLYLTFY | 336.38 | 0.7994 | 0.07476 | No | Class I |
| 3112 | 3120 | LTFYLTNDV | 302.73 | 0.3755 | 0.06752 | No | Class I |
| 3114 | 3122 | FYLTNDVSF | 33.17 | 0.8216 | -0.01178 | No | Class I |
| 3115 | 3123 | YLTNDVSFL | 7.38 | 0.534 | -0.00467 | No | Class I |
| 3116 | 3124 | LTNDVSFLA | 97.83 | 0.4826 | -0.00299 | No | Class I |
| 3118 | 3126 | NDVSFLAHI | 413.27 | 0.7081 | 0.00241 | No | Class I |
| 3120 | 3128 | VSFLAHIQW | 185.57 | 1.4691 | 0.14003 | No | Class I |
| 3121 | 3129 | SFLAHIQWM | 123.99 | 1.0511 | 0.22421 | No | Class I |
| 3123 | 3131 | LAHIQWMVM | 462.21 | 1.3148 | 0.11605 | No | Class I |
| 3124 | 3132 | AHIQWMVMF | 392.62 | 1.1004 | -0.09072 | No | Class I |
| 3127 | 3135 | QWMVMFTPL | 174.12 | 0.5293 | -0.04998 | No | Class I |
| 3128 | 3136 | WMVMFTPLV | 10.24 | 0.4651 | -0.0286 | No | Class I |
| 3130 | 3138 | VMFTPLVPF | 359.3 | 0.6358 | 0.08418 | No | Class I |
| 3131 | 3139 | MFTPLVPFW | 143.45 | -0.0722 | 0.08854 | No | Class I |
| 3132 | 3140 | FTPLVPFWI | 384.78 | 0.4253 | 0.24322 | No | Class I |
| 3133 | 3141 | TPLVPFWIT | 190.33 | 0.5275 | 0.40204 | No | Class I |
| 3135 | 3143 | LVPFWITIA | 449.48 | 1.0741 | 0.5657 | No | Class I |
| 3136 | 3144 | VPFWITIAY | 95.56 | 1.0982 | 0.56221 | No | Class I |
| 3138 | 3146 | FWITIAYII | 250.54 | 0.7151 | 0.32333 | No | Class I |
| 3140 | 3148 | ITIAYIICI | 202.09 | 1.0331 | 0.28507 | No | Class I |
| 3143 | 3151 | AYIICISTK | 270.81 | 1.4349 | 0.13296 | No | Class I |
| 3145 | 3153 | IICISTKHF | 246.55 | 1.6562 | -0.17124 | No | Class I |
| 3147 | 3155 | CISTKHFYW | 418.62 | 1.9 | -0.09755 | No | Class I |
| 3148 | 3156 | ISTKHFYWF | 226.68 | 1.6767 | 0.0636 | No | Class I |
| 3149 | 3157 | STKHFYWFF | 331.7 | 1.341 | 0.32841 | No | Class I |
| 3153 | 3161 | FYWFFSNYL | 381.79 | 0.0226 | 0.14035 | No | Class I |
| 3154 | 3162 | YWFFSNYLK | 150.66 | -0.0204 | -0.02099 | No | Class I |
| 3155 | 3163 | WFFSNYLKR | 69.57 | -0.1788 | -0.27361 | No | Class I |
| 3156 | 3164 | FFSNYLKRR | 301.31 | -0.0181 | -0.22601 | No | Class I |
| 3157 | 3165 | FSNYLKRRV | 123.31 | -0.0985 | -0.1457 | No | Class I |
| 3158 | 3166 | SNYLKRRVV | 131.97 | -0.2054 | -0.10584 | No | Class I |
| 3159 | 3167 | NYLKRRVVF | 139.24 | 0.1867 | -0.06252 | No | Class I |
| 3162 | 3170 | KRRVVFNGV | 486.79 | 0.603 | 0.22308 | No | Class I |
| 3164 | 3172 | RVVFNGVSF | 322.47 | 0.7252 | 0.09498 | No | Class I |
| 3172 | 3180 | FSTFEEAAL | 50 | 0.2405 | 0.37803 | No | Class I |
| 3175 | 3183 | FEEAALCTF | 45.26 | 0.1803 | 0.07671 | No | Class I |
| 3176 | 3184 | EEAALCTFL | 95.2 | 0.0277 | 0.09168 | No | Class I |
| 3179 | 3187 | ALCTFLLNK | 18.62 | -0.3727 | 0.11198 | No | Class I |
| 3181 | 3189 | CTFLLNKEM | 97.75 | 0.1732 | -0.11355 | No | Class I |
| 3182 | 3190 | TFLLNKEMY | 101.58 | 0.0499 | -0.24216 | No | Class I |
| 3183 | 3191 | FLLNKEMYL | 2.67 | 0.44 | -0.27622 | No | Class I |
| 3184 | 3192 | LLNKEMYLK | 61 | 0.1795 | -0.2965 | No | Class I |
| 3187 | 3195 | KEMYLKLRS | 497.9 | 0.7862 | -0.25364 | No | Class I |
| 3189 | 3197 | MYLKLRSDV | 179.47 | 0.4144 | -0.30934 | No | Class I |
| 3190 | 3198 | YLKLRSDVL | 69.94 | 0.2052 | -0.14365 | No | Class I |
| 3192 | 3200 | KLRSDVLLP | 353.13 | -0.1494 | -0.10505 | No | Class I |
| 3193 | 3201 | LRSDVLLPL | 164.01 | 0.0306 | -0.01746 | No | Class I |
| 3198 | 3206 | LLPLTQYNR | 289.43 | 1.0422 | -0.0929 | No | Class I |
| 3199 | 3207 | LPLTQYNRY | 66.45 | 0.8861 | -0.05604 | No | Class I |
| 3201 | 3209 | LTQYNRYLA | 171.07 | 0.4737 | -0.0085 | No | Class I |
| 3202 | 3210 | TQYNRYLAL | 191.17 | 0.7829 | 0.05271 | No | Class I |
| 3203 | 3211 | QYNRYLALY | 159.76 | 0.2466 | 0.06248 | No | Class I |
| 3206 | 3214 | RYLALYNKY | 333.66 | 0.6952 | -0.10997 | No | Class I |
| 3207 | 3215 | YLALYNKYK | 102.83 | 0.9434 | -0.19231 | No | Class I |
| 3208 | 3216 | LALYNKYKY | 146.43 | 1.0479 | -0.34574 | No | Class I |
| 3209 | 3217 | ALYNKYKYF | 266.18 | 0.6668 | -0.40535 | No | Class I |
| 3211 | 3219 | YNKYKYFSG | 398.22 | -0.0601 | -0.28506 | No | Class I |
| 3213 | 3221 | KYKYFSGAM | 116.12 | 0.4963 | -0.06399 | No | Class I |
| 3218 | 3226 | SGAMDTTSY | 301.02 | 0.3928 | -0.16976 | No | Class I |
| 3219 | 3227 | GAMDTTSYR | 331.75 | 0.7031 | -0.10212 | No | Class I |
| 3225 | 3233 | SYREAACCH | 383.31 | 1.238 | 0.11548 | No | Class I |
| 3226 | 3234 | YREAACCHL | 138.15 | 1.2791 | 0.03262 | No | Class I |
| 3227 | 3235 | REAACCHLA | 76.52 | 1.4206 | -0.03036 | No | Class I |
| 3228 | 3236 | EAACCHLAK | 298.54 | 0.7978 | -0.0501 | No | Class I |
| 3233 | 3241 | HLAKALNDF | 93.72 | -0.1893 | -0.16914 | No | Class I |
| 3241 | 3249 | FSNSGSDVL | 279.35 | 0.0426 | -0.24846 | No | Class I |
| 3242 | 3250 | SNSGSDVLY | 294.84 | 0.1785 | -0.13146 | No | Class I |
| 3249 | 3257 | LYQPPQTSI | 180.67 | -0.0753 | -0.2325 | No | Class I |
| 3254 | 3262 | QTSITSAVL | 54.95 | 0.4963 | 0.01943 | No | Class I |
| 3258 | 3266 | TSAVLQSGF | 16.69 | 0.2645 | -0.18542 | No | Class I |
| 3259 | 3267 | SAVLQSGFR | 219.89 | 0.2732 | -0.16929 | No | Class I |
| 3260 | 3268 | AVLQSGFRK | 52.22 | -0.2915 | -0.12032 | No | Class I |
| 3261 | 3269 | VLQSGFRKM | 259.51 | -0.4118 | -0.14319 | No | Class I |
| 3263 | 3271 | QSGFRKMAF | 197.26 | 0.0906 | -0.14914 | No | Class I |
| 3265 | 3273 | GFRKMAFPS | 453.73 | 0.7466 | -0.24205 | No | Class I |
| 3267 | 3275 | RKMAFPSGK | 300.04 | 1.0658 | -0.03389 | No | Class I |
| 3268 | 3276 | KMAFPSGKV | 458.6 | 0.6043 | -0.13343 | No | Class I |
| 3275 | 3283 | KVEGCMVQV | 496.68 | -0.3985 | -0.18404 | No | Class I |
| 3279 | 3287 | CMVQVTCGT | 224.53 | 0.9765 | -0.05212 | No | Class I |
| 3287 | 3295 | TTTLNGLWL | 423.91 | 0.3046 | 0.1471 | No | Class I |
| 3292 | 3300 | GLWLDDVVY | 395.61 | -0.0961 | 0.16218 | No | Class I |
| 3298 | 3306 | VVYCPRHVI | 357.16 | 0.3984 | 0.03389 | No | Class I |
| 3301 | 3309 | CPRHVICTS | 234.56 | -0.08 | 0.19201 | No | Class I |
| 3304 | 3312 | HVICTSEDM | 448.03 | 0.7858 | -0.03152 | No | Class I |
| 3309 | 3317 | SEDMLNPNY | 271.39 | 0.7652 | -0.19953 | No | Class I |
| 3312 | 3320 | MLNPNYEDL | 216.54 | 1.521 | 0.07442 | No | Class I |
| 3321 | 3329 | LIRKSNHNF | 116.46 | 0.7071 | -0.34387 | No | Class I |
| 3322 | 3330 | IRKSNHNFL | 121.16 | 0.6636 | -0.14938 | No | Class I |
| 3330 | 3338 | LVQAGNVQL | 315.83 | 0.951 | -0.00416 | No | Class I |
| 3331 | 3339 | VQAGNVQLR | 476.29 | 1.9857 | -0.02488 | No | Class I |
| 3337 | 3345 | QLRVIGHSM | 429.02 | 0.0244 | 0.15048 | No | Class I |
| 3339 | 3347 | RVIGHSMQN | 364.47 | -0.0702 | -0.26281 | No | Class I |
| 3342 | 3350 | GHSMQNCVL | 132.72 | -0.095 | -0.37067 | No | Class I |
| 3343 | 3351 | HSMQNCVLK | 210.75 | -0.7971 | -0.20225 | No | Class I |
| 3344 | 3352 | SMQNCVLKL | 50.58 | -0.6459 | -0.19311 | No | Class I |
| 3345 | 3353 | MQNCVLKLK | 249.41 | 0.4992 | -0.21507 | No | Class I |
| 3349 | 3357 | VLKLKVDTA | 126.98 | -0.3876 | -0.2109 | No | Class I |
| 3355 | 3363 | DTANPKTPK | 490.67 | -0.3276 | -0.18133 | No | Class I |
| 3356 | 3364 | TANPKTPKY | 340.03 | -0.0019 | -0.32208 | No | Class I |
| 3360 | 3368 | KTPKYKFVR | 17.68 | -1.4302 | -0.30428 | No | Class I |
| 3361 | 3369 | TPKYKFVRI | 337.58 | -0.2699 | -0.10844 | No | Class I |
| 3363 | 3371 | KYKFVRIQP | 103.13 | 0.4782 | 0.18136 | No | Class I |
| 3367 | 3375 | VRIQPGQTF | 446.29 | 0.5434 | -0.12734 | No | Class I |
| 3368 | 3376 | RIQPGQTFS | 466.43 | 0.259 | -0.02364 | No | Class I |
| 3369 | 3377 | IQPGQTFSV | 138.48 | 0.4817 | -0.04362 | No | Class I |
| 3370 | 3378 | QPGQTFSVL | 24.45 | -0.1094 | -0.07306 | No | Class I |
| 3373 | 3381 | QTFSVLACY | 191.4 | 0.3763 | -0.09719 | No | Class I |
| 3380 | 3388 | CYNGSPSGV | 247.14 | 0.4766 | -0.25936 | No | Class I |
| 3381 | 3389 | YNGSPSGVY | 318.04 | 0.1654 | -0.26928 | No | Class I |
| 3384 | 3392 | SPSGVYQCA | 425.4 | -0.3679 | -0.11214 | No | Class I |
| 3386 | 3394 | SGVYQCAMR | 286.34 | -0.7446 | -0.22345 | No | Class I |
| 3389 | 3397 | YQCAMRPNF | 469.27 | 0.6464 | -0.11355 | No | Class I |
| 3391 | 3399 | CAMRPNFTI | 336.87 | 0.9571 | 0.09967 | No | Class I |
| 3396 | 3404 | NFTIKGSFL | 54.83 | 1.5061 | -0.1028 | No | Class I |
| 3398 | 3406 | TIKGSFLNG | 276.38 | 0.0738 | -0.09994 | No | Class I |
| 3403 | 3411 | FLNGSCGSV | 19.55 | 0.1804 | -0.24791 | No | Class I |
| 3409 | 3417 | GSVGFNIDY | 199.16 | 1.8062 | 0.28069 | No | Class I |
| 3416 | 3424 | DYDCVSFCY | 262.23 | 2.1139 | -0.09528 | No | Class I |
| 3420 | 3428 | VSFCYMHHM | 351.1 | 1.0477 | -0.13895 | No | Class I |
| 3422 | 3430 | FCYMHHMEL | 65.31 | 1.0828 | -0.20565 | No | Class I |
| 3424 | 3432 | YMHHMELPT | 137.68 | -0.061 | -0.04954 | No | Class I |
| 3426 | 3434 | HHMELPTGV | 244.42 | 0.1523 | 0.07507 | No | Class I |
| 3428 | 3436 | MELPTGVHA | 483.78 | 0.0107 | 0.10868 | No | Class I |
| 3430 | 3438 | LPTGVHAGT | 163.08 | 0.3466 | 0.17017 | No | Class I |
| 3437 | 3445 | GTDLEGNFY | 9.71 | -0.1424 | 0.18838 | No | Class I |
| 3440 | 3448 | LEGNFYGPF | 129.56 | -0.6267 | 0.13713 | No | Class I |
| 3443 | 3451 | NFYGPFVDR | 85.08 | 0.0903 | 0.1801 | No | Class I |
| 3446 | 3454 | GPFVDRQTA | 409.25 | 1.0654 | 0.07478 | No | Class I |
| 3448 | 3456 | FVDRQTAQA | 450.86 | 0.7887 | -0.05164 | No | Class I |
| 3461 | 3469 | TTITVNVLA | 483.95 | 0.3033 | 0.14473 | No | Class I |
| 3462 | 3470 | TITVNVLAW | 225.29 | 0.0932 | 0.1002 | No | Class I |
| 3463 | 3471 | ITVNVLAWL | 295.48 | 0.0729 | 0.19909 | No | Class I |
| 3464 | 3472 | TVNVLAWLY | 449.58 | 0.1215 | 0.24593 | No | Class I |
| 3466 | 3474 | NVLAWLYAA | 24.16 | 0.0799 | 0.26077 | No | Class I |
| 3467 | 3475 | VLAWLYAAV | 9.1 | 0.3598 | 0.27719 | No | Class I |
| 3468 | 3476 | LAWLYAAVI | 384.3 | 0.806 | 0.15111 | No | Class I |
| 3472 | 3480 | YAAVINGDR | 245.6 | -0.0491 | 0.21931 | No | Class I |
| 3473 | 3481 | AAVINGDRW | 178.17 | -0.6615 | 0.22188 | No | Class I |
| 3474 | 3482 | AVINGDRWF | 352.89 | -0.7519 | 0.26367 | No | Class I |
| 3475 | 3483 | VINGDRWFL | 408.55 | -1.0158 | 0.35766 | No | Class I |
| 3480 | 3488 | RWFLNRFTT | 313.24 | -2.2634 | 0.19074 | No | Class I |
| 3481 | 3489 | WFLNRFTTT | 473.37 | -0.5377 | 0.20593 | No | Class I |
| 3482 | 3490 | FLNRFTTTL | 6.61 | -0.6268 | 0.25596 | No | Class I |
| 3485 | 3493 | RFTTTLNDF | 424.08 | 0.2186 | 0.08652 | No | Class I |
| 3488 | 3496 | TTLNDFNLV | 493.36 | 1.4652 | 0.10975 | No | Class I |
| 3489 | 3497 | TLNDFNLVA | 141.64 | 1.4845 | 0.14289 | No | Class I |
| 3492 | 3500 | DFNLVAMKY | 146.7 | 1.8134 | -0.21043 | No | Class I |
| 3494 | 3502 | NLVAMKYNY | 56.21 | 1.2184 | -0.32813 | No | Class I |
| 3497 | 3505 | AMKYNYEPL | 67.88 | 1.5538 | -0.00548 | No | Class I |
| 3508 | 3516 | DHVDILGPL | 90.91 | 0.1151 | 0.177 | No | Class I |
| 3512 | 3520 | ILGPLSAQT | 292.69 | 0.6074 | -0.20135 | No | Class I |
| 3514 | 3522 | GPLSAQTGI | 249.15 | 1.0285 | -0.18845 | No | Class I |
| 3516 | 3524 | LSAQTGIAV | 155.38 | 0.9189 | 0.10102 | No | Class I |
| 3517 | 3525 | SAQTGIAVL | 380.4 | 0.7628 | 0.21688 | No | Class I |
| 3523 | 3531 | AVLDMCASL | 181.71 | 0.005 | -0.26667 | No | Class I |
| 3524 | 3532 | VLDMCASLK | 73.94 | 0.3342 | -0.33127 | No | Class I |
| 3534 | 3542 | LLQNGMNGR | 362.23 | 0.6615 | -0.16207 | No | Class I |
| 3541 | 3549 | GRTILGSAL | 409.74 | -0.3178 | 0.05086 | No | Class I |
| 3542 | 3550 | RTILGSALL | 200.05 | -0.2697 | -0.06415 | No | Class I |
| 3549 | 3557 | LLEDEFTPF | 223.72 | 0.564 | 0.2888 | No | Class I |
| 3552 | 3560 | DEFTPFDVV | 85.49 | 1.2644 | 0.2193 | No | Class I |
| 3555 | 3563 | TPFDVVRQC | 407.68 | 0.263 | 0.11538 | No | Class I |
| 3560 | 3568 | VRQCSGVTF | 332.98 | -0.1446 | -0.16353 | No | Class I |
| 3565 | 3573 | GVTFQSAVK | 377.93 | 0.5835 | -0.08099 | No | Class I |
| 3566 | 3574 | VTFQSAVKR | 296.16 | 0.0954 | -0.29399 | No | Class I |
| 3568 | 3576 | FQSAVKRTI | 285.89 | -0.37 | -0.11077 | No | Class I |
| 3569 | 3577 | QSAVKRTIK | 165.53 | -0.2405 | 0.00348 | No | Class I |
| 3574 | 3582 | RTIKGTHHW | 180.77 | 0.4453 | -0.05806 | No | Class I |
| 3579 | 3587 | THHWLLLTI | 308.94 | 0.8305 | 0.22547 | No | Class I |
| 3580 | 3588 | HHWLLLTIL | 422.81 | 0.8132 | 0.15002 | No | Class I |
| 3583 | 3591 | LLLTILTSL | 11.67 | 0.1737 | 0.09072 | No | Class I |
| 3584 | 3592 | LLTILTSLL | 82.75 | -0.3023 | 0.02616 | No | Class I |
| 3585 | 3593 | LTILTSLLV | 341.26 | 0.0023 | -0.10173 | No | Class I |
| 3586 | 3594 | TILTSLLVL | 290.7 | -0.0966 | -0.12132 | No | Class I |
| 3587 | 3595 | ILTSLLVLV | 6.75 | 0.0068 | -0.14675 | No | Class I |
| 3592 | 3600 | LVLVQSTQW | 15.39 | 1.0492 | -0.26551 | No | Class I |
| 3594 | 3602 | LVQSTQWSL | 314.51 | 1.095 | -0.18503 | No | Class I |
| 3595 | 3603 | VQSTQWSLF | 366.6 | 1.0681 | -0.06503 | No | Class I |
| 3597 | 3605 | STQWSLFFF | 248.92 | 0.7828 | 0.18095 | No | Class I |
| 3603 | 3611 | FFFLYENAF | 97.9 | 0.1318 | 0.13489 | No | Class I |
| 3604 | 3612 | FFLYENAFL | 213.09 | 0.0063 | 0.18551 | No | Class I |
| 3605 | 3613 | FLYENAFLP | 43.34 | 0.1667 | 0.2224 | No | Class I |
| 3607 | 3615 | YENAFLPFA | 380.46 | 0.9047 | 0.19987 | No | Class I |
| 3610 | 3618 | AFLPFAMGI | 170.39 | 1.4929 | 0.00767 | No | Class I |
| 3611 | 3619 | FLPFAMGII | 370.05 | 1.5447 | 0.09336 | No | Class I |
| 3612 | 3620 | LPFAMGIIA | 115.7 | 1.5082 | 0.12835 | No | Class I |
| 3613 | 3621 | PFAMGIIAM | 244.45 | 1.5004 | 0.12946 | No | Class I |
| 3614 | 3622 | FAMGIIAMS | 295.13 | 1.1569 | 0.1624 | No | Class I |
| 3615 | 3623 | AMGIIAMSA | 454.6 | 0.9558 | 0.06649 | No | Class I |
| 3616 | 3624 | MGIIAMSAF | 40.21 | 0.8089 | -0.06684 | No | Class I |
| 3617 | 3625 | GIIAMSAFA | 421.81 | 0.5505 | -0.14274 | No | Class I |
| 3618 | 3626 | IIAMSAFAM | 379.58 | 0.4804 | -0.16661 | No | Class I |
| 3619 | 3627 | IAMSAFAMM | 193.1 | 0.6384 | -0.14475 | No | Class I |
| 3620 | 3628 | AMSAFAMMF | 338.39 | 0.3032 | -0.1143 | No | Class I |
| 3621 | 3629 | MSAFAMMFV | 63.18 | 0.4244 | -0.0765 | No | Class I |
| 3622 | 3630 | SAFAMMFVK | 38.79 | 0.5411 | -0.13601 | No | Class I |
| 3624 | 3632 | FAMMFVKHK | 110.46 | 1.1005 | -0.24394 | No | Class I |
| 3626 | 3634 | MMFVKHKHA | 66.5 | 1.0072 | -0.26311 | No | Class I |
| 3627 | 3635 | MFVKHKHAF | 378.08 | 0.6621 | -0.32494 | No | Class I |
| 3628 | 3636 | FVKHKHAFL | 190.75 | 0.5763 | -0.11558 | No | Class I |
| 3632 | 3640 | KHAFLCLFL | 260.8 | 1.3666 | 0.12799 | No | Class I |
| 3633 | 3641 | HAFLCLFLL | 250.88 | 0.4608 | 0.05622 | No | Class I |
| 3635 | 3643 | FLCLFLLPS | 218.51 | 0.4233 | 0.05906 | No | Class I |
| 3637 | 3645 | CLFLLPSLA | 166.93 | 0.4482 | -0.1405 | No | Class I |
| 3639 | 3647 | FLLPSLATV | 2.41 | 0.5954 | -0.1306 | No | Class I |
| 3640 | 3648 | LLPSLATVA | 103.55 | 0.6257 | -0.08716 | No | Class I |
| 3641 | 3649 | LPSLATVAY | 402.57 | 0.6748 | 0.06748 | No | Class I |
| 3642 | 3650 | PSLATVAYF | 264.51 | 0.6614 | 0.14329 | No | Class I |
| 3644 | 3652 | LATVAYFNM | 119.88 | 0.8804 | 0.18378 | No | Class I |
| 3645 | 3653 | ATVAYFNMV | 179.47 | 0.9418 | 0.05131 | No | Class I |
| 3646 | 3654 | TVAYFNMVY | 360.18 | 0.8942 | -0.00719 | No | Class I |
| 3647 | 3655 | VAYFNMVYM | 34.38 | 0.9133 | -0.02232 | No | Class I |
| 3649 | 3657 | YFNMVYMPA | 154.42 | 0.7027 | -0.29676 | No | Class I |
| 3651 | 3659 | NMVYMPASW | 174.64 | 0.6912 | -0.2354 | No | Class I |
| 3652 | 3660 | MVYMPASWV | 213.9 | 0.3986 | -0.16207 | No | Class I |
| 3653 | 3661 | VYMPASWVM | 17.24 | 0.3448 | 0.02527 | No | Class I |
| 3655 | 3663 | MPASWVMRI | 99.55 | 0.6855 | -0.01717 | No | Class I |
| 3658 | 3666 | SWVMRIMTW | 92.7 | 0.0206 | -0.11314 | No | Class I |
| 3659 | 3667 | WVMRIMTWL | 159.47 | -0.1997 | 0.12156 | No | Class I |
| 3661 | 3669 | MRIMTWLDM | 255.13 | 0.3083 | 0.11641 | No | Class I |
| 3662 | 3670 | RIMTWLDMV | 6.02 | 0.2348 | 0.10344 | No | Class I |
| 3666 | 3674 | WLDMVDTSL | 23.07 | 1.1148 | -0.17232 | No | Class I |
| 3669 | 3677 | MVDTSLSGF | 397.47 | 0.1823 | -0.2451 | No | Class I |
| 3672 | 3680 | TSLSGFKLK | 244.57 | 1.0652 | -0.21535 | No | Class I |
| 3678 | 3686 | KLKDCVMYA | 18.2 | 0.076 | -0.21168 | No | Class I |
| 3682 | 3690 | CVMYASAVV | 234.39 | 0.5448 | -0.12121 | No | Class I |
| 3683 | 3691 | VMYASAVVL | 64.38 | 0.4778 | -0.02714 | No | Class I |
| 3684 | 3692 | MYASAVVLL | 23.32 | 0.4033 | -0.04845 | No | Class I |
| 3685 | 3693 | YASAVVLLI | 80.2 | 0.3843 | 0.04889 | No | Class I |
| 3686 | 3694 | ASAVVLLIL | 84.9 | 0.3238 | 0.1524 | No | Class I |
| 3687 | 3695 | SAVVLLILM | 323.51 | 0.3321 | 0.13954 | No | Class I |
| 3689 | 3697 | VVLLILMTA | 257.09 | 0.5358 | -0.02112 | No | Class I |
| 3690 | 3698 | VLLILMTAR | 154.58 | 1.0528 | 0.00984 | No | Class I |
| 3691 | 3699 | LLILMTART | 135.17 | 1.1183 | -0.03916 | No | Class I |
| 3692 | 3700 | LILMTARTV | 285.98 | 1.075 | -0.03931 | No | Class I |
| 3693 | 3701 | ILMTARTVY | 376.03 | 1.097 | 0.12576 | No | Class I |
| 3698 | 3706 | RTVYDDGAR | 366.16 | -0.8384 | 0.10362 | No | Class I |
| 3699 | 3707 | TVYDDGARR | 257.1 | -1.0705 | 0.13788 | No | Class I |
| 3700 | 3708 | VYDDGARRV | 361.14 | -1.2526 | 0.17327 | No | Class I |
| 3703 | 3711 | DGARRVWTL | 88.6 | -0.9234 | 0.36366 | No | Class I |
| 3704 | 3712 | GARRVWTLM | 72.69 | -0.1946 | 0.34387 | No | Class I |
| 3706 | 3714 | RRVWTLMNV | 136.5 | 0.3871 | 0.11167 | No | Class I |
| 3707 | 3715 | RVWTLMNVL | 131.79 | -0.1718 | -0.04648 | No | Class I |
| 3709 | 3717 | WTLMNVLTL | 337.99 | 0.6364 | -0.13442 | No | Class I |
| 3710 | 3718 | TLMNVLTLV | 3.31 | 0.2297 | -0.00747 | No | Class I |
| 3711 | 3719 | LMNVLTLVY | 280.18 | 0.278 | 0.07994 | No | Class I |
| 3712 | 3720 | MNVLTLVYK | 408.1 | 0.655 | 0.06228 | No | Class I |
| 3713 | 3721 | NVLTLVYKV | 73.8 | 0.4569 | -0.0656 | No | Class I |
| 3714 | 3722 | VLTLVYKVY | 474.77 | 0.6325 | -0.11972 | No | Class I |
| 3715 | 3723 | LTLVYKVYY | 433.85 | 1.0511 | -0.13598 | No | Class I |
| 3719 | 3727 | YKVYYGNAL | 241.94 | 0.1399 | 0.05538 | No | Class I |
| 3725 | 3733 | NALDQAISM | 35.07 | 0.4381 | -0.04159 | No | Class I |
| 3728 | 3736 | DQAISMWAL | 498.84 | 0.3033 | 0.03002 | No | Class I |
| 3729 | 3737 | QAISMWALI | 385.44 | 0.1989 | -0.05922 | No | Class I |
| 3732 | 3740 | SMWALIISV | 3.8 | 0.3925 | 0.24141 | No | Class I |
| 3736 | 3744 | LIISVTSNY | 95.52 | 0.935 | -0.18993 | No | Class I |
| 3739 | 3747 | SVTSNYSGV | 67.91 | 0.5854 | -0.28347 | No | Class I |
| 3740 | 3748 | VTSNYSGVV | 286.72 | 0.4834 | -0.16682 | No | Class I |
| 3743 | 3751 | NYSGVVTTV | 71.81 | 0.6448 | 0.1149 | No | Class I |
| 3744 | 3752 | YSGVVTTVM | 399.44 | 0.3704 | 0.18616 | No | Class I |
| 3745 | 3753 | SGVVTTVMF | 404.11 | 0.4296 | 0.06152 | No | Class I |
| 3746 | 3754 | GVVTTVMFL | 394.34 | 0.5221 | 0.04932 | No | Class I |
| 3747 | 3755 | VVTTVMFLA | 98.41 | 0.5223 | 0.01888 | No | Class I |
| 3748 | 3756 | VTTVMFLAR | 167.46 | 0.7986 | 0.00684 | No | Class I |
| 3750 | 3758 | TVMFLARGI | 204.11 | 0.8792 | 0.15031 | No | Class I |
| 3751 | 3759 | VMFLARGIV | 96.74 | 0.6861 | 0.22002 | No | Class I |
| 3752 | 3760 | MFLARGIVF | 170.48 | 0.6099 | 0.25451 | No | Class I |
| 3757 | 3765 | GIVFMCVEY | 189.36 | 1.1133 | 0.00279 | No | Class I |
| 3761 | 3769 | MCVEYCPIF | 468.81 | 0.8988 | 0.1282 | No | Class I |
| 3763 | 3771 | VEYCPIFFI | 183.19 | 0.9732 | 0.22623 | No | Class I |
| 3768 | 3776 | IFFITGNTL | 34.78 | 0.2315 | 0.25884 | No | Class I |
| 3774 | 3782 | NTLQCIMLV | 252.22 | 1.0406 | -0.20206 | No | Class I |
| 3775 | 3783 | TLQCIMLVY | 38.06 | 1.5051 | -0.11279 | No | Class I |
| 3780 | 3788 | MLVYCFLGY | 266.2 | 0.3026 | 0.07782 | No | Class I |
| 3781 | 3789 | LVYCFLGYF | 451.46 | -0.1115 | 0.07455 | No | Class I |
| 3785 | 3793 | FLGYFCTCY | 52.82 | -0.5859 | 0.07179 | No | Class I |
| 3786 | 3794 | LGYFCTCYF | 365.68 | -0.1892 | 0.05298 | No | Class I |
| 3788 | 3796 | YFCTCYFGL | 197.92 | -0.6624 | 0.08418 | No | Class I |
| 3792 | 3800 | CYFGLFCLL | 194.34 | 1.4515 | 0.11952 | No | Class I |
| 3794 | 3802 | FGLFCLLNR | 250.21 | 1.5548 | 0.03812 | No | Class I |
| 3795 | 3803 | GLFCLLNRY | 66.77 | 0.9526 | -0.01271 | No | Class I |
| 3796 | 3804 | LFCLLNRYF | 346.11 | 0.6222 | -0.00403 | No | Class I |
| 3797 | 3805 | FCLLNRYFR | 131.37 | 0.6714 | 0.09294 | No | Class I |
| 3798 | 3806 | CLLNRYFRL | 46.52 | 0.2999 | 0.16585 | No | Class I |
| 3799 | 3807 | LLNRYFRLT | 308.35 | 0.5335 | 0.19378 | No | Class I |
| 3800 | 3808 | LNRYFRLTL | 328.42 | 0.2874 | 0.18912 | No | Class I |
| 3802 | 3810 | RYFRLTLGV | 130.31 | 0.6783 | 0.12626 | No | Class I |
| 3803 | 3811 | YFRLTLGVY | 68.19 | 0.8939 | 0.08572 | No | Class I |
| 3805 | 3813 | RLTLGVYDY | 135.56 | 1.2905 | 0.08314 | No | Class I |
| 3806 | 3814 | LTLGVYDYL | 363.56 | 1.1816 | 0.08378 | No | Class I |
| 3809 | 3817 | GVYDYLVST | 75.07 | 0.2897 | -0.05474 | No | Class I |
| 3812 | 3820 | DYLVSTQEF | 164.51 | 0.4761 | -0.12588 | No | Class I |
| 3813 | 3821 | YLVSTQEFR | 197.77 | 0.775 | -0.07141 | No | Class I |
| 3814 | 3822 | LVSTQEFRY | 457.37 | 1.107 | 0.09585 | No | Class I |
| 3820 | 3828 | FRYMNSQGL | 38.61 | 0.1788 | -0.41789 | No | Class I |
| 3821 | 3829 | RYMNSQGLL | 95.24 | -0.1668 | -0.31153 | No | Class I |
| 3824 | 3832 | NSQGLLPPK | 408.71 | 1.022 | -0.04058 | No | Class I |
| 3837 | 3845 | AFKLNIKLL | 244.71 | 1.148 | -0.15066 | No | Class I |
| 3839 | 3847 | KLNIKLLGV | 35.39 | 1.4506 | -0.07818 | No | Class I |
| 3850 | 3858 | KPCIKVATV | 321.83 | 0.8368 | 0.00098 | No | Class I |
| 3854 | 3862 | KVATVQSKM | 67.72 | 0.7999 | -0.2827 | No | Class I |
| 3857 | 3865 | TVQSKMSDV | 291.17 | 1.3343 | -0.70603 | No | Class I |
| 3862 | 3870 | MSDVKCTSV | 392.75 | 1.6242 | -0.27591 | No | Class I |
| 3867 | 3875 | CTSVVLLSV | 185.97 | 0.6209 | -0.08842 | No | Class I |
| 3868 | 3876 | TSVVLLSVL | 62.73 | 0.4957 | -0.0818 | No | Class I |
| 3871 | 3879 | VLLSVLQQL | 17.88 | 0.181 | -0.30575 | No | Class I |
| 3872 | 3880 | LLSVLQQLR | 385.44 | 0.5588 | -0.23624 | No | Class I |
| 3873 | 3881 | LSVLQQLRV | 175.33 | 0.6107 | -0.19872 | No | Class I |
| 3878 | 3886 | QLRVESSSK | 178.5 | 0.7726 | -0.23617 | No | Class I |
| 3879 | 3887 | LRVESSSKL | 353.28 | 0.5996 | -0.4683 | No | Class I |
| 3880 | 3888 | RVESSSKLW | 53.33 | -0.1118 | -0.63928 | No | Class I |
| 3884 | 3892 | SSKLWAQCV | 335.49 | -0.0182 | 0.04211 | No | Class I |
| 3886 | 3894 | KLWAQCVQL | 8.97 | 0.3106 | -0.08512 | No | Class I |
| 3892 | 3900 | VQLHNDILL | 124.91 | 0.1531 | 0.14937 | No | Class I |
| 3893 | 3901 | QLHNDILLA | 370.9 | 0.2178 | 0.13503 | No | Class I |
| 3899 | 3907 | LLAKDTTEA | 234.16 | 0.5585 | -0.0549 | No | Class I |
| 3900 | 3908 | LAKDTTEAF | 67.86 | -0.0274 | 0.13402 | No | Class I |
| 3903 | 3911 | DTTEAFEKM | 258.98 | -0.0853 | 0.22015 | No | Class I |
| 3906 | 3914 | EAFEKMVSL | 314.61 | -0.129 | -0.29837 | No | Class I |
| 3907 | 3915 | AFEKMVSLL | 444.57 | -0.04 | -0.46274 | No | Class I |
| 3910 | 3918 | KMVSLLSVL | 33.15 | 0.6526 | -0.28981 | No | Class I |
| 3911 | 3919 | MVSLLSVLL | 127.24 | 0.3847 | -0.20303 | No | Class I |
| 3913 | 3921 | SLLSVLLSM | 17.23 | 0.5227 | -0.24633 | No | Class I |
| 3916 | 3924 | SVLLSMQGA | 396.26 | 0.6474 | -0.41912 | No | Class I |
| 3917 | 3925 | VLLSMQGAV | 86.01 | 0.4987 | -0.39865 | No | Class I |
| 3919 | 3927 | LSMQGAVDI | 72.09 | 0.7749 | -0.05593 | No | Class I |
| 3921 | 3929 | MQGAVDINK | 419.8 | 0.2308 | 0.21999 | No | Class I |
| 3940 | 3948 | TLQAIASEF | 79.52 | 0.2681 | 0.08708 | No | Class I |
| 3943 | 3951 | AIASEFSSL | 23.74 | 0.5439 | -0.18235 | No | Class I |
| 3946 | 3954 | SEFSSLPSY | 365.39 | 0.5595 | -0.40603 | No | Class I |
| 3948 | 3956 | FSSLPSYAA | 232.31 | 0.356 | -0.21165 | No | Class I |
| 3949 | 3957 | SSLPSYAAF | 112.06 | 0.3791 | -0.12346 | No | Class I |
| 3950 | 3958 | SLPSYAAFA | 42.23 | 0.4689 | -0.03542 | No | Class I |
| 3951 | 3959 | LPSYAAFAT | 256.15 | 0.336 | 0.13917 | No | Class I |
| 3953 | 3961 | SYAAFATAQ | 343.17 | 0.7174 | 0.25852 | No | Class I |
| 3955 | 3963 | AAFATAQEA | 244.39 | 0.5301 | 0.11274 | No | Class I |
| 3956 | 3964 | AFATAQEAY | 91.42 | 0.4527 | 0.08818 | No | Class I |
| 3967 | 3975 | AVANGDSEV | 122.07 | 0.1288 | -0.02105 | No | Class I |
| 3968 | 3976 | VANGDSEVV | 452.03 | 0.1037 | 0.00649 | No | Class I |
| 3974 | 3982 | EVVLKKLKK | 171.65 | 0.1968 | -0.54612 | No | Class I |
| 3976 | 3984 | VLKKLKKSL | 55.64 | -0.1057 | -0.77946 | No | Class I |
| 3979 | 3987 | KLKKSLNVA | 51.5 | 0.4823 | -0.43988 | No | Class I |
| 3983 | 3991 | SLNVAKSEF | 92.02 | 0.5671 | -0.20658 | No | Class I |
| 3985 | 3993 | NVAKSEFDR | 151.31 | -0.9865 | -0.15939 | No | Class I |
| 3989 | 3997 | SEFDRDAAM | 94.16 | 0.0334 | 0.18748 | No | Class I |
| 3995 | 4003 | AAMQRKLEK | 82.18 | 0.0256 | -0.27702 | No | Class I |
| 4001 | 4009 | LEKMADQAM | 266.61 | 0.0357 | -0.26262 | No | Class I |
| 4004 | 4012 | MADQAMTQM | 285.49 | 0.393 | -0.27148 | No | Class I |
| 4006 | 4014 | DQAMTQMYK | 124.76 | -0.0155 | -0.3856 | No | Class I |
| 4008 | 4016 | AMTQMYKQA | 406.01 | 0.2144 | -0.52812 | No | Class I |
| 4009 | 4017 | MTQMYKQAR | 15.32 | 0.2921 | -0.4958 | No | Class I |
| 4021 | 4029 | KRAKVTSAM | 144.79 | 0.7604 | -0.24432 | No | Class I |
| 4022 | 4030 | RAKVTSAMQ | 192.78 | 0.8064 | -0.21597 | No | Class I |
| 4024 | 4032 | KVTSAMQTM | 18.74 | 0.4846 | -0.35615 | No | Class I |
| 4025 | 4033 | VTSAMQTML | 22.59 | 0.1395 | -0.36421 | No | Class I |
| 4026 | 4034 | TSAMQTMLF | 233.16 | -0.0679 | -0.39494 | No | Class I |
| 4027 | 4035 | SAMQTMLFT | 395.85 | -0.1372 | -0.24202 | No | Class I |
| 4028 | 4036 | AMQTMLFTM | 29.18 | -0.0001 | -0.0585 | No | Class I |
| 4029 | 4037 | MQTMLFTML | 380.64 | -0.0398 | -0.13454 | No | Class I |
| 4030 | 4038 | QTMLFTMLR | 149.59 | 0.0312 | -0.0723 | No | Class I |
| 4031 | 4039 | TMLFTMLRK | 11.05 | 0.2549 | 0.00758 | No | Class I |
| 4032 | 4040 | MLFTMLRKL | 57.54 | 0.1454 | -0.1867 | No | Class I |
| 4044 | 4052 | ALNNIINNA | 116.12 | -0.5345 | 0.23703 | No | Class I |
| 4045 | 4053 | LNNIINNAR | 180.95 | -0.0504 | 0.27273 | No | Class I |
| 4051 | 4059 | NARDGCVPL | 252.02 | 1.7204 | 0.04973 | No | Class I |
| 4056 | 4064 | CVPLNIIPL | 384.19 | 1.3687 | 0.21006 | No | Class I |
| 4057 | 4065 | VPLNIIPLT | 299.34 | 1.4077 | 0.22893 | No | Class I |
| 4060 | 4068 | NIIPLTTAA | 176.18 | 1.1604 | 0.1134 | No | Class I |
| 4061 | 4069 | IIPLTTAAK | 404.48 | 1.1333 | 0.11546 | No | Class I |
| 4062 | 4070 | IPLTTAAKL | 193.43 | 0.938 | 0.01711 | No | Class I |
| 4064 | 4072 | LTTAAKLMV | 470.89 | 0.2092 | -0.22489 | No | Class I |
| 4065 | 4073 | TTAAKLMVV | 53.89 | 0.3457 | -0.29245 | No | Class I |
| 4066 | 4074 | TAAKLMVVI | 423.7 | 0.273 | -0.32144 | No | Class I |
| 4069 | 4077 | KLMVVIPDY | 246.38 | 0.1932 | 0.15362 | No | Class I |
| 4072 | 4080 | VVIPDYNTY | 100.52 | 0.3692 | 0.06738 | No | Class I |
| 4073 | 4081 | VIPDYNTYK | 374.69 | 0.0339 | 0.03963 | No | Class I |
| 4083 | 4091 | TCDGTTFTY | 109.65 | -0.0785 | 0.23712 | No | Class I |
| 4087 | 4095 | TTFTYASAL | 263.61 | 0.5713 | -0.00647 | No | Class I |
| 4088 | 4096 | TFTYASALW | 146.2 | 0.2311 | -0.08221 | No | Class I |
| 4090 | 4098 | TYASALWEI | 18.68 | 0.1933 | 0.11933 | No | Class I |
| 4093 | 4101 | SALWEIQQV | 106.1 | 0.7568 | 0.27663 | No | Class I |
| 4096 | 4104 | WEIQQVVDA | 109.15 | 0.7509 | -0.0995 | No | Class I |
| 4108 | 4116 | IVQLSEISM | 387.64 | 1.2265 | -0.09995 | No | Class I |
| 4114 | 4122 | ISMDNSPNL | 213.24 | 0.7721 | -0.20985 | No | Class I |
| 4116 | 4124 | MDNSPNLAW | 337.85 | 1.081 | -0.17196 | No | Class I |
| 4118 | 4126 | NSPNLAWPL | 262.75 | 0.5951 | 0.19638 | No | Class I |
| 4119 | 4127 | SPNLAWPLI | 170.63 | 0.774 | 0.21751 | No | Class I |
| 4121 | 4129 | NLAWPLIVT | 207.43 | 1.3774 | 0.35079 | No | Class I |
| 4122 | 4130 | LAWPLIVTA | 119.74 | 1.0167 | 0.23274 | No | Class I |
| 4123 | 4131 | AWPLIVTAL | 102.8 | 0.8684 | 0.20932 | No | Class I |
| 4124 | 4132 | WPLIVTALR | 308.87 | 1.2662 | 0.2336 | No | Class I |
| 4128 | 4136 | VTALRANSA | 496.88 | 0.5179 | -0.01335 | No | Class I |
| 4129 | 4137 | TALRANSAV | 199.09 | 0.2828 | -0.03627 | No | Class I |
| 4130 | 4138 | ALRANSAVK | 50.43 | 0.3333 | -0.04872 | No | Class I |
| 4131 | 4139 | LRANSAVKL | 238.01 | 0.3781 | -0.20924 | No | Class I |
| 4139 | 4147 | LQNNELSPV | 75.31 | 1.5437 | -0.06765 | No | Class I |
| 4144 | 4152 | LSPVALRQM | 308.71 | 1.3859 | 0.0416 | No | Class I |
| 4148 | 4156 | ALRQMSCAA | 74.1 | 0.3512 | -0.44913 | No | Class I |
| 4150 | 4158 | RQMSCAAGT | 133.31 | 0.8575 | -0.18632 | No | Class I |
| 4154 | 4162 | CAAGTTQTA | 331.7 | 0.793 | 0.04606 | No | Class I |
| 4163 | 4171 | CTDDNALAY | 2.6 | -0.0409 | 0.07355 | No | Class I |
| 4171 | 4179 | YYNTTKGGR | 219.97 | 0.8034 | -0.07984 | No | Class I |
| 4174 | 4182 | TTKGGRFVL | 254.65 | 0.6063 | 0.16874 | No | Class I |
| 4177 | 4185 | GGRFVLALL | 374.47 | -0.0714 | 0.1909 | No | Class I |
| 4180 | 4188 | FVLALLSDL | 70.4 | 0.2377 | -0.11213 | No | Class I |
| 4183 | 4191 | ALLSDLQDL | 48.1 | 0.5147 | -0.24371 | No | Class I |
| 4184 | 4192 | LLSDLQDLK | 461.99 | 0.7165 | -0.13898 | No | Class I |
| 4185 | 4193 | LSDLQDLKW | 231.1 | 1.8637 | -0.23124 | No | Class I |
| 4187 | 4195 | DLQDLKWAR | 11.51 | 1.9979 | -0.01928 | No | Class I |
| 4190 | 4198 | DLKWARFPK | 316.6 | 1.3034 | 0.33203 | No | Class I |
| 4198 | 4206 | KSDGTGTIY | 56.33 | 0.365 | 0.22152 | No | Class I |
| 4207 | 4215 | TELEPPCRF | 405.42 | 1.4251 | 0.06065 | No | Class I |
| 4216 | 4224 | VTDTPKGPK | 137.49 | 0.2801 | -0.14542 | No | Class I |
| 4222 | 4230 | GPKVKYLYF | 452.25 | 1.3841 | -0.25346 | No | Class I |
| 4224 | 4232 | KVKYLYFIK | 75.12 | 1.0623 | 0.08856 | No | Class I |
| 4226 | 4234 | KYLYFIKGL | 22.25 | 0.3663 | 0.06976 | No | Class I |
| 4229 | 4237 | YFIKGLNNL | 242.04 | 0.0972 | -0.16048 | No | Class I |
| 4233 | 4241 | GLNNLNRGM | 379.97 | 1.0073 | 0.03798 | No | Class I |
| 4235 | 4243 | NNLNRGMVL | 147.18 | 0.5195 | -0.05189 | No | Class I |
| 4238 | 4246 | NRGMVLGSL | 467.21 | 1.1229 | -0.204 | No | Class I |
| 4239 | 4247 | RGMVLGSLA | 209.32 | 0.9475 | -0.14046 | No | Class I |
| 4240 | 4248 | GMVLGSLAA | 85.02 | 0.5696 | -0.10699 | No | Class I |
| 4241 | 4249 | MVLGSLAAT | 476.64 | 0.9983 | -0.08516 | No | Class I |
| 4242 | 4250 | VLGSLAATV | 43.61 | 0.6548 | -0.07374 | No | Class I |
| 4244 | 4252 | GSLAATVRL | 65.02 | 0.9183 | 0.17549 | No | Class I |
| 4246 | 4254 | LAATVRLQA | 145.58 | 1.1024 | 0.06364 | No | Class I |
| 4249 | 4257 | TVRLQAGNA | 202.59 | 0.6603 | -0.04551 | No | Class I |
| 4252 | 4260 | LQAGNATEV | 354.02 | 1.0588 | 0.16859 | No | Class I |
| 4258 | 4266 | TEVPANSTV | 124.84 | -0.0178 | -0.08269 | No | Class I |
| 4259 | 4267 | EVPANSTVL | 180.8 | -0.0979 | -0.06938 | No | Class I |
| 4264 | 4272 | STVLSFCAF | 354.33 | 0.2897 | -0.0713 | No | Class I |
| 4265 | 4273 | TVLSFCAFA | 85.85 | 0.4516 | -0.0054 | No | Class I |
| 4266 | 4274 | VLSFCAFAV | 7.66 | 0.343 | 0.17009 | No | Class I |
| 4270 | 4278 | CAFAVDAAK | 317.01 | 0.1437 | 0.19433 | No | Class I |
| 4272 | 4280 | FAVDAAKAY | 195.13 | 0.1671 | -0.04849 | No | Class I |
| 4273 | 4281 | AVDAAKAYK | 114.67 | 0.3594 | -0.08747 | No | Class I |
| 4283 | 4291 | YLASGGQPI | 17.3 | 0.3202 | -0.19311 | No | Class I |
| 4289 | 4297 | QPITNCVKM | 179.53 | 0.3063 | -0.06595 | No | Class I |
| 4299 | 4307 | CTHTGTGQA | 462.57 | 1.1272 | 0.08002 | No | Class I |
| 4300 | 4308 | THTGTGQAI | 301.84 | 0.6026 | 0.0415 | No | Class I |
| 4306 | 4314 | QAITVTPEA | 387.44 | 0.9634 | 0.20814 | No | Class I |
| 4313 | 4321 | EANMDQESF | 88.75 | 0.1454 | -0.2784 | No | Class I |
| 4320 | 4328 | SFGGASCCL | 93.08 | 0.097 | -0.14953 | No | Class I |
| 4321 | 4329 | FGGASCCLY | 196.61 | -0.1526 | -0.21346 | No | Class I |
| 4323 | 4331 | GASCCLYCR | 103.8 | 1.3701 | -0.20551 | No | Class I |
| 4341 | 4349 | GFCDLKGKY | 210.06 | 2.3726 | -0.30638 | No | Class I |
| 4344 | 4352 | DLKGKYVQI | 138.55 | 1.3769 | -0.28222 | No | Class I |
| 4346 | 4354 | KGKYVQIPT | 103.61 | 0.2729 | -0.03672 | No | Class I |
| 4348 | 4356 | KYVQIPTTC | 347.9 | 0.6425 | 0.07144 | No | Class I |
| 4364 | 4372 | TLKNTVCTV | 217.18 | 0.1542 | -0.02267 | No | Class I |
| 4367 | 4375 | NTVCTVCGM | 154.44 | 0.8663 | 0.01011 | No | Class I |
| 4368 | 4376 | TVCTVCGMW | 342.1 | 0.6928 | -0.06299 | No | Class I |
| 4371 | 4379 | TVCGMWKGY | 289.79 | -0.2307 | -0.10809 | No | Class I |
| 4378 | 4386 | GYGCSCDQL | 440.29 | -0.018 | -0.30406 | No | Class I |
| 4388 | 4396 | EPMLQSADA | 283.4 | 0.4953 | -0.29071 | No | Class I |
| 4391 | 4399 | LQSADAQSF | 367.61 | 0.3631 | -0.15032 | No | Class I |
| 4392 | 4400 | QSADAQSFL | 104.78 | -0.0251 | -0.10714 | No | Class I |
| 4398 | 4406 | SFLNRVCGV | 162.58 | 0.0892 | 0.05345 | No | Class I |
| 4399 | 4407 | FLNRVCGVS | 153.82 | 0.1957 | 0.09215 | No | Class I |
| 4400 | 4408 | LNRVCGVSA | 368.7 | 0.5705 | -0.02408 | No | Class I |
| 4402 | 4410 | RVCGVSAAR | 231.11 | 0.6078 | -0.04305 | No | Class I |
| 4408 | 4416 | AARLTPCGT | 471.92 | 0.4278 | 0.0073 | No | Class I |
| 4416 | 4424 | TGTSTDVVY | 377.15 | 0.7823 | -0.03623 | No | Class I |
| 4417 | 4425 | GTSTDVVYR | 193.26 | 0.3881 | 0.0785 | No | Class I |
| 4419 | 4427 | STDVVYRAF | 100.55 | 0.1207 | 0.152 | No | Class I |
| 4422 | 4430 | VVYRAFDIY | 295.33 | 0.7331 | 0.29566 | No | Class I |
| 4425 | 4433 | RAFDIYNDK | 345.87 | 0.5993 | 0.19394 | No | Class I |
| 4429 | 4437 | IYNDKVAGF | 153.34 | 0.5316 | -0.0981 | No | Class I |
| 4433 | 4441 | KVAGFAKFL | 310.49 | -0.3185 | 0.08403 | No | Class I |
| 4434 | 4442 | VAGFAKFLK | 176.78 | -0.7456 | 0.05622 | No | Class I |
| 4439 | 4447 | KFLKTNCCR | 21.69 | -1.4883 | -0.26589 | No | Class I |
| 4440 | 4448 | FLKTNCCRF | 234.35 | -0.9871 | -0.10325 | No | Class I |
| 4457 | 4465 | LIDSYFVVK | 356.55 | 0.4939 | 0.00629 | No | Class I |
| 4461 | 4469 | YFVVKRHTF | 103.7 | 0.2169 | -0.05636 | No | Class I |
| 4463 | 4471 | VVKRHTFSN | 227.01 | 0.0384 | 0.05226 | No | Class I |
| 4471 | 4479 | NYQHEETIY | 314.16 | 0.5631 | 0.29722 | No | Class I |
| 4473 | 4481 | QHEETIYNL | 170.23 | 0.3403 | 0.28943 | No | Class I |
| 4474 | 4482 | HEETIYNLL | 16.81 | -0.1743 | 0.18574 | No | Class I |
| 4480 | 4488 | NLLKDCPAV | 23.28 | -0.5347 | -0.23625 | No | Class I |
| 4481 | 4489 | LLKDCPAVA | 15.72 | -0.1897 | -0.05348 | No | Class I |
| 4485 | 4493 | CPAVAKHDF | 349.46 | 1.1025 | -0.0704 | No | Class I |
| 4487 | 4495 | AVAKHDFFK | 30.39 | -0.8254 | 0.01528 | No | Class I |
| 4488 | 4496 | VAKHDFFKF | 70.06 | -0.7734 | 0.06715 | No | Class I |
| 4489 | 4497 | AKHDFFKFR | 491.37 | -0.0391 | 0.14342 | No | Class I |
| 4502 | 4510 | MVPHISRQR | 113.51 | 0.4279 | -0.02118 | No | Class I |
| 4503 | 4511 | VPHISRQRL | 15.53 | 0.1685 | -0.03548 | No | Class I |
| 4505 | 4513 | HISRQRLTK | 79.67 | -0.8467 | -0.05238 | No | Class I |
| 4506 | 4514 | ISRQRLTKY | 335.3 | -0.6868 | -0.15304 | No | Class I |
| 4508 | 4516 | RQRLTKYTM | 85.93 | -0.6352 | -0.14 | No | Class I |
| 4511 | 4519 | LTKYTMADL | 226.66 | 0.7838 | -0.15524 | No | Class I |
| 4513 | 4521 | KYTMADLVY | 344.09 | 1.0811 | -0.09036 | No | Class I |
| 4516 | 4524 | MADLVYALR | 114.64 | 1.1376 | 0.0593 | No | Class I |
| 4518 | 4526 | DLVYALRHF | 392.62 | 0.8873 | 0.09992 | No | Class I |
| 4533 | 4541 | TLKEILVTY | 187.6 | 0.1118 | 0.20743 | No | Class I |
| 4535 | 4543 | KEILVTYNC | 160.3 | 0.2136 | 0.10188 | No | Class I |
| 4557 | 4565 | FVENPDILR | 37.84 | -0.8253 | 0.14191 | No | Class I |
| 4560 | 4568 | NPDILRVYA | 115.09 | -0.4347 | 0.21172 | No | Class I |
| 4565 | 4573 | RVYANLGER | 69.81 | 0.6654 | 0.10853 | No | Class I |
| 4566 | 4574 | VYANLGERV | 341.67 | 0.8457 | 0.14203 | No | Class I |
| 4567 | 4575 | YANLGERVR | 385.56 | 1.5877 | 0.18179 | No | Class I |
| 4569 | 4577 | NLGERVRQA | 157.79 | 1.3291 | 0.17701 | No | Class I |
| 4571 | 4579 | GERVRQALL | 293.55 | 0.5377 | 0.02624 | No | Class I |
| 4573 | 4581 | RVRQALLKT | 13.89 | -0.5069 | -0.20746 | No | Class I |
| 4574 | 4582 | VRQALLKTV | 238.83 | -0.7141 | -0.17879 | No | Class I |
| 4576 | 4584 | QALLKTVQF | 114.19 | 0.4574 | -0.22106 | No | Class I |
| 4580 | 4588 | KTVQFCDAM | 493.43 | 1.0973 | 0.00167 | No | Class I |
| 4581 | 4589 | TVQFCDAMR | 113.47 | 0.0894 | -0.021 | No | Class I |
| 4587 | 4595 | AMRNAGIVG | 102.07 | 1.2886 | 0.21673 | No | Class I |
| 4588 | 4596 | MRNAGIVGV | 275.36 | 1.6262 | 0.25019 | No | Class I |
| 4607 | 4615 | NWYDFGDFI | 96.56 | 1.2208 | 0.25414 | No | Class I |
| 4615 | 4623 | IQTTPGSGV | 419.53 | 0.4543 | -0.04706 | No | Class I |
| 4617 | 4625 | TTPGSGVPV | 209.64 | -0.0706 | -0.07034 | No | Class I |
| 4618 | 4626 | TPGSGVPVV | 197.67 | -0.1432 | -0.06885 | No | Class I |
| 4621 | 4629 | SGVPVVDSY | 264.11 | 0.5855 | 0.00336 | No | Class I |
| 4622 | 4630 | GVPVVDSYY | 336.38 | 0.8383 | -0.04276 | No | Class I |
| 4625 | 4633 | VVDSYYSLL | 211.33 | 0.5381 | -0.31245 | No | Class I |
| 4628 | 4636 | SYYSLLMPI | 23.43 | 0.4337 | -0.34359 | No | Class I |
| 4629 | 4637 | YYSLLMPIL | 216.73 | 0.2957 | -0.17256 | No | Class I |
| 4631 | 4639 | SLLMPILTL | 47.58 | 0.2133 | -0.0525 | No | Class I |
| 4633 | 4641 | LMPILTLTR | 225.16 | 0.9617 | 0.16938 | No | Class I |
| 4634 | 4642 | MPILTLTRA | 286.46 | 0.8061 | 0.1224 | No | Class I |
| 4637 | 4645 | LTLTRALTA | 320.78 | 0.2746 | 0.13601 | No | Class I |
| 4639 | 4647 | LTRALTAES | 101.1 | -0.1807 | 0.17343 | No | Class I |
| 4645 | 4653 | AESHVDTDL | 34.01 | -0.2205 | 0.08565 | No | Class I |
| 4655 | 4663 | KPYIKWDLL | 116.74 | 0.4825 | 0.14347 | No | Class I |
| 4657 | 4665 | YIKWDLLKY | 33.2 | -0.321 | 0.02869 | No | Class I |
| 4659 | 4667 | KWDLLKYDF | 40.95 | -0.1545 | -0.20792 | No | Class I |
| 4664 | 4672 | KYDFTEERL | 209.17 | 0.6634 | 0.37179 | No | Class I |
| 4667 | 4675 | FTEERLKLF | 185.34 | 0.0551 | -0.01527 | No | Class I |
| 4671 | 4679 | RLKLFDRYF | 256.4 | 0.5225 | 0.09524 | No | Class I |
| 4673 | 4681 | KLFDRYFKY | 15.44 | -0.0533 | 0.08004 | No | Class I |
| 4674 | 4682 | LFDRYFKYW | 393.59 | -0.4457 | -0.01828 | No | Class I |
| 4677 | 4685 | RYFKYWDQT | 367.13 | 0.0653 | -0.02305 | No | Class I |
| 4678 | 4686 | YFKYWDQTY | 312.2 | 0.3243 | 0.08778 | No | Class I |
| 4686 | 4694 | YHPNCVNCL | 315.65 | 1.1402 | -0.06071 | No | Class I |
| 4697 | 4705 | RCILHCANF | 488.3 | 0.9708 | 0.04203 | No | Class I |
| 4700 | 4708 | LHCANFNVL | 118.73 | 1.0867 | 0.14443 | No | Class I |
| 4704 | 4712 | NFNVLFSTV | 158.39 | 0.7397 | 0.0219 | No | Class I |
| 4705 | 4713 | FNVLFSTVF | 317.28 | 0.116 | 0.01739 | No | Class I |
| 4707 | 4715 | VLFSTVFPP | 179.62 | 0.1228 | 0.04051 | No | Class I |
| 4710 | 4718 | STVFPPTSF | 230.25 | 0.4047 | 0.04606 | No | Class I |
| 4717 | 4725 | SFGPLVRKI | 126.26 | 1.2822 | -0.05442 | No | Class I |
| 4724 | 4732 | KIFVDGVPF | 31.83 | -0.7658 | 0.1614 | No | Class I |
| 4725 | 4733 | IFVDGVPFV | 221.34 | -0.2614 | 0.16662 | No | Class I |
| 4726 | 4734 | FVDGVPFVV | 7.54 | 0.1285 | 0.19398 | No | Class I |
| 4730 | 4738 | VPFVVSTGY | 497.36 | 1.1641 | 0.01657 | No | Class I |
| 4732 | 4740 | FVVSTGYHF | 325.42 | 0.8838 | -0.06759 | No | Class I |
| 4733 | 4741 | VVSTGYHFR | 155.24 | 1.4741 | 0.11058 | No | Class I |
| 4735 | 4743 | STGYHFREL | 455.16 | 1.3083 | 0.25116 | No | Class I |
| 4738 | 4746 | YHFRELGVV | 74.8 | 1.9563 | 0.22986 | No | Class I |
| 4739 | 4747 | HFRELGVVH | 464.55 | 1.7918 | 0.19761 | No | Class I |
| 4752 | 4760 | NLHSSRLSF | 399.61 | 0.8607 | -0.37437 | No | Class I |
| 4755 | 4763 | SSRLSFKEL | 44.9 | 1.4448 | -0.16876 | No | Class I |
| 4756 | 4764 | SRLSFKELL | 410.39 | 0.8666 | -0.18105 | No | Class I |
| 4757 | 4765 | RLSFKELLV | 99.68 | 0.8643 | -0.06749 | No | Class I |
| 4758 | 4766 | LSFKELLVY | 237.31 | 0.7234 | -0.07718 | No | Class I |
| 4759 | 4767 | SFKELLVYA | 233.51 | -0.0953 | 0.04219 | No | Class I |
| 4763 | 4771 | LLVYAADPA | 487.52 | 0.2037 | 0.09685 | No | Class I |
| 4764 | 4772 | LVYAADPAM | 278.22 | 0.5148 | 0.11065 | No | Class I |
| 4766 | 4774 | YAADPAMHA | 389.35 | 0.4901 | -0.06825 | No | Class I |
| 4771 | 4779 | AMHAASGNL | 53.24 | 0.4822 | -0.04294 | No | Class I |
| 4772 | 4780 | MHAASGNLL | 101.8 | 0.1758 | -0.08907 | No | Class I |
| 4773 | 4781 | HAASGNLLL | 65.22 | 0.1413 | -0.1427 | No | Class I |
| 4775 | 4783 | ASGNLLLDK | 416.69 | -0.4206 | -0.01315 | No | Class I |
| 4780 | 4788 | LLDKRTTCF | 209.03 | 1.7656 | -0.1216 | No | Class I |
| 4784 | 4792 | RTTCFSVAA | 229.54 | 1.751 | -0.02568 | No | Class I |
| 4785 | 4793 | TTCFSVAAL | 256.09 | 1.0411 | 0.03394 | No | Class I |
| 4789 | 4797 | SVAALTNNV | 249.87 | 0.3839 | 0.06857 | No | Class I |
| 4791 | 4799 | AALTNNVAF | 110.73 | 0.6381 | 0.08077 | No | Class I |
| 4795 | 4803 | NNVAFQTVK | 225.72 | 0.7987 | 0.11461 | No | Class I |
| 4799 | 4807 | FQTVKPGNF | 368.04 | 1.3501 | -0.14148 | No | Class I |
| 4801 | 4809 | TVKPGNFNK | 170.79 | 1.3778 | 0.04077 | No | Class I |
| 4806 | 4814 | NFNKDFYDF | 114.47 | 0.1232 | -0.07746 | No | Class I |
| 4808 | 4816 | NKDFYDFAV | 165.02 | 0.8605 | 0.26394 | No | Class I |
| 4810 | 4818 | DFYDFAVSK | 68.1 | 0.8301 | 0.11013 | No | Class I |
| 4814 | 4822 | FAVSKGFFK | 480.98 | -0.2311 | -0.16397 | No | Class I |
| 4819 | 4827 | GFFKEGSSV | 264.44 | -0.1274 | -0.28588 | No | Class I |
| 4821 | 4829 | FKEGSSVEL | 33.29 | 0.6423 | -0.15689 | No | Class I |
| 4824 | 4832 | GSSVELKHF | 172.91 | 1.2013 | -0.0882 | No | Class I |
| 4825 | 4833 | SSVELKHFF | 15.78 | 1.0328 | -0.00395 | No | Class I |
| 4827 | 4835 | VELKHFFFA | 477.19 | 1.6266 | 0.0883 | No | Class I |
| 4833 | 4841 | FFAQDGNAA | 230.07 | 0.264 | -0.03296 | No | Class I |
| 4834 | 4842 | FAQDGNAAI | 171.84 | 0.1992 | 0.06751 | No | Class I |
| 4840 | 4848 | AAISDYDYY | 175.23 | 1.1426 | -0.08859 | No | Class I |
| 4841 | 4849 | AISDYDYYR | 110.94 | 0.585 | -0.01938 | No | Class I |
| 4847 | 4855 | YYRYNLPTM | 94.9 | -0.037 | 0.00966 | No | Class I |
| 4848 | 4856 | YRYNLPTMC | 425.06 | 0.0511 | -0.09879 | No | Class I |
| 4854 | 4862 | TMCDIRQLL | 362.92 | -0.0353 | 0.0789 | No | Class I |
| 4860 | 4868 | QLLFVVEVV | 58.87 | 0.821 | 0.30188 | No | Class I |
| 4867 | 4875 | VVDKYFDCY | 19.96 | 0.1676 | -0.11598 | No | Class I |
| 4879 | 4887 | CINANQVIV | 408.24 | 0.6717 | 0.03453 | No | Class I |
| 4884 | 4892 | QVIVNNLDK | 277.56 | -0.5685 | 0.07595 | No | Class I |
| 4893 | 4901 | SAGFPFNKW | 90.28 | 0.0001 | 0.09674 | No | Class I |
| 4896 | 4904 | FPFNKWGKA | 480.37 | -0.4851 | -0.0674 | No | Class I |
| 4900 | 4908 | KWGKARLYY | 212.52 | -0.598 | -0.1307 | No | Class I |
| 4903 | 4911 | KARLYYDSM | 79.9 | -0.0551 | -0.07938 | No | Class I |
| 4905 | 4913 | RLYYDSMSY | 9.22 | -0.1146 | -0.38391 | No | Class I |
| 4911 | 4919 | MSYEDQDAL | 473.84 | 1.05 | 0.05369 | No | Class I |
| 4912 | 4920 | SYEDQDALF | 53.33 | 0.942 | -0.01056 | No | Class I |
| 4919 | 4927 | LFAYTKRNV | 448.21 | 1.0269 | -0.11632 | No | Class I |
| 4920 | 4928 | FAYTKRNVI | 25.95 | 1.0296 | -0.10476 | No | Class I |
| 4922 | 4930 | YTKRNVIPT | 268.15 | 0.9913 | 0.12048 | No | Class I |
| 4926 | 4934 | NVIPTITQM | 457.75 | 0.7329 | 0.1602 | No | Class I |
| 4928 | 4936 | IPTITQMNL | 218.19 | 0.7997 | -0.0767 | No | Class I |
| 4929 | 4937 | PTITQMNLK | 269.03 | 1.5033 | -0.20778 | No | Class I |
| 4930 | 4938 | TITQMNLKY | 31.52 | 1.6155 | -0.41641 | No | Class I |
| 4931 | 4939 | ITQMNLKYA | 314.58 | 1.6256 | -0.4152 | No | Class I |
| 4932 | 4940 | TQMNLKYAI | 232.41 | 1.2979 | -0.25757 | No | Class I |
| 4935 | 4943 | NLKYAISAK | 253.16 | 1.3508 | -0.0271 | No | Class I |
| 4937 | 4945 | KYAISAKNR | 50.38 | 1.5099 | -0.16343 | No | Class I |
| 4938 | 4946 | YAISAKNRA | 479.46 | 1.644 | -0.26339 | No | Class I |
| 4939 | 4947 | AISAKNRAR | 108.56 | 1.986 | -0.16388 | No | Class I |
| 4941 | 4949 | SAKNRARTV | 292.39 | 0.9661 | 0.07708 | No | Class I |
| 4943 | 4951 | KNRARTVAG | 93.56 | 0.5746 | 0.20081 | No | Class I |
| 4945 | 4953 | RARTVAGVS | 54.22 | 0.9583 | 0.18561 | No | Class I |
| 4953 | 4961 | SICSTMTNR | 400.23 | 1.1164 | -0.28249 | No | Class I |
| 4955 | 4963 | CSTMTNRQF | 17.48 | 1.1304 | -0.15639 | No | Class I |
| 4956 | 4964 | STMTNRQFH | 342.99 | 1.22 | -0.00488 | No | Class I |
| 4958 | 4966 | MTNRQFHQK | 55.54 | 0.5341 | 0.007 | No | Class I |
| 4960 | 4968 | NRQFHQKLL | 225.95 | -0.3713 | -0.18582 | No | Class I |
| 4961 | 4969 | RQFHQKLLK | 14.51 | -1.0437 | -0.26109 | No | Class I |
| 4963 | 4971 | FHQKLLKSI | 226.83 | -0.3929 | -0.5545 | No | Class I |
| 4966 | 4974 | KLLKSIAAT | 92.07 | -0.4372 | -0.20054 | No | Class I |
| 4967 | 4975 | LLKSIAATR | 28.53 | 0.2055 | -0.01434 | No | Class I |
| 4971 | 4979 | IAATRGATV | 112.03 | 0.8883 | 0.18976 | No | Class I |
| 4972 | 4980 | AATRGATVV | 428.06 | 0.7053 | 0.19139 | No | Class I |
| 4977 | 4985 | ATVVIGTSK | 181.89 | 0.7566 | 0.15254 | No | Class I |
| 4978 | 4986 | TVVIGTSKF | 299.89 | 0.5085 | -0.04876 | No | Class I |
| 4979 | 4987 | VVIGTSKFY | 482.3 | 0.7146 | -0.15423 | No | Class I |
| 4982 | 4990 | GTSKFYGGW | 359.18 | -0.4705 | -0.11178 | No | Class I |
| 4985 | 4993 | KFYGGWHNM | 187.61 | -0.5876 | 0.29793 | No | Class I |
| 4986 | 4994 | FYGGWHNML | 178.33 | -0.5032 | 0.18319 | No | Class I |
| 4987 | 4995 | YGGWHNMLK | 421.79 | -0.4574 | 0.10462 | No | Class I |
| 4990 | 4998 | WHNMLKTVY | 32.93 | -0.1503 | -0.33572 | No | Class I |
| 4993 | 5001 | MLKTVYSDV | 9.16 | -0.0749 | -0.12088 | No | Class I |
| 4998 | 5006 | YSDVENPHL | 190.09 | 0.502 | 0.14969 | No | Class I |
| 5001 | 5009 | VENPHLMGW | 20.78 | 0.4705 | -0.1206 | No | Class I |
| 5005 | 5013 | HLMGWDYPK | 31.38 | -0.2495 | 0.20408 | No | Class I |
| 5010 | 5018 | DYPKCDRAM | 260.04 | -0.7096 | -0.18568 | No | Class I |
| 5016 | 5024 | RAMPNMLRI | 468.57 | 0.0566 | -0.21888 | No | Class I |
| 5017 | 5025 | AMPNMLRIM | 408.8 | -0.1195 | -0.07011 | No | Class I |
| 5018 | 5026 | MPNMLRIMA | 75.91 | -0.2116 | -0.13116 | No | Class I |
| 5020 | 5028 | NMLRIMASL | 46.48 | -0.3326 | -0.05086 | No | Class I |
| 5021 | 5029 | MLRIMASLV | 15.88 | -0.1881 | -0.12955 | No | Class I |
| 5022 | 5030 | LRIMASLVL | 114.51 | 0.1355 | -0.23637 | No | Class I |
| 5023 | 5031 | RIMASLVLA | 41.28 | 0.1241 | -0.16081 | No | Class I |
| 5024 | 5032 | IMASLVLAR | 140.36 | 0.5587 | -0.11221 | No | Class I |
| 5025 | 5033 | MASLVLARK | 216.62 | 1.3011 | 0.02816 | No | Class I |
| 5029 | 5037 | VLARKHTTC | 408.48 | 1.4662 | -0.05933 | No | Class I |
| 5035 | 5043 | TTCCSLSHR | 231.71 | 1.8662 | -0.36401 | No | Class I |
| 5038 | 5046 | CSLSHRFYR | 115.45 | 0.629 | 0.00679 | No | Class I |
| 5039 | 5047 | SLSHRFYRL | 121.29 | 0.4779 | 0.16657 | No | Class I |
| 5040 | 5048 | LSHRFYRLA | 52.71 | 0.5957 | 0.2103 | No | Class I |
| 5047 | 5055 | LANECAQVL | 407.95 | -0.052 | 0.00934 | No | Class I |
| 5052 | 5060 | AQVLSEMVM | 41.08 | -0.0412 | -0.18869 | No | Class I |
| 5057 | 5065 | EMVMCGGSL | 413.76 | -0.1363 | -0.25196 | No | Class I |
| 5058 | 5066 | MVMCGGSLY | 312.41 | -0.259 | -0.19245 | No | Class I |
| 5059 | 5067 | VMCGGSLYV | 11.11 | -0.2332 | -0.11765 | No | Class I |
| 5064 | 5072 | SLYVKPGGT | 187.08 | 0.9216 | -0.1317 | No | Class I |
| 5073 | 5081 | SSGDATTAY | 288.04 | 0.3022 | 0.16358 | No | Class I |
| 5077 | 5085 | ATTAYANSV | 273.27 | 0.3175 | -0.01692 | No | Class I |
| 5078 | 5086 | TTAYANSVF | 317.92 | 0.2542 | -0.07451 | No | Class I |
| 5080 | 5088 | AYANSVFNI | 30.02 | 0.458 | -0.02103 | No | Class I |
| 5081 | 5089 | YANSVFNIC | 328.78 | -0.0674 | 0.05413 | No | Class I |
| 5084 | 5092 | SVFNICQAV | 61.15 | -0.416 | 0.03544 | No | Class I |
| 5091 | 5099 | AVTANVNAL | 476.79 | 0.3733 | 0.10193 | No | Class I |
| 5092 | 5100 | VTANVNALL | 368.36 | 0.1708 | 0.06684 | No | Class I |
| 5098 | 5106 | ALLSTDGNK | 221.91 | -0.1557 | -0.08657 | No | Class I |
| 5099 | 5107 | LLSTDGNKI | 196.44 | -0.449 | -0.0926 | No | Class I |
| 5107 | 5115 | IADKYVRNL | 269.29 | 0.1321 | -0.13464 | No | Class I |
| 5110 | 5118 | KYVRNLQHR | 32.55 | -0.32 | -0.03012 | No | Class I |
| 5111 | 5119 | YVRNLQHRL | 303.11 | -0.4066 | -0.05201 | No | Class I |
| 5112 | 5120 | VRNLQHRLY | 457.2 | -0.3047 | -0.05841 | No | Class I |
| 5116 | 5124 | QHRLYECLY | 390.5 | -0.3365 | 0.04431 | No | Class I |
| 5119 | 5127 | LYECLYRNR | 152.88 | 0.181 | 0.00387 | No | Class I |
| 5130 | 5138 | DTDFVNEFY | 6.35 | 0.2521 | 0.31201 | No | Class I |
| 5132 | 5140 | DFVNEFYAY | 125.51 | -0.0363 | 0.23433 | No | Class I |
| 5133 | 5141 | FVNEFYAYL | 4.97 | -0.4788 | 0.24003 | No | Class I |
| 5137 | 5145 | FYAYLRKHF | 277.75 | 0.0359 | -0.1162 | No | Class I |
| 5138 | 5146 | YAYLRKHFS | 349.15 | 0.0945 | -0.06926 | No | Class I |
| 5139 | 5147 | AYLRKHFSM | 238.93 | 0.5598 | -0.12893 | No | Class I |
| 5140 | 5148 | YLRKHFSMM | 20.15 | 0.4887 | -0.30072 | No | Class I |
| 5141 | 5149 | LRKHFSMMI | 108.43 | 0.3045 | -0.32998 | No | Class I |
| 5146 | 5154 | SMMILSDDA | 299.6 | -0.0837 | -0.05793 | No | Class I |
| 5147 | 5155 | MMILSDDAV | 35.28 | -0.1616 | -0.0666 | No | Class I |
| 5148 | 5156 | MILSDDAVV | 144.46 | -0.1842 | -0.07045 | No | Class I |
| 5150 | 5158 | LSDDAVVCF | 375.32 | 0.5894 | 0.10982 | No | Class I |
| 5154 | 5162 | AVVCFNSTY | 328.21 | 1.3806 | -0.04988 | No | Class I |
| 5160 | 5168 | STYASQGLV | 152.91 | 0.681 | -0.20985 | No | Class I |
| 5161 | 5169 | TYASQGLVA | 227.4 | 0.7675 | -0.21991 | No | Class I |
| 5162 | 5170 | YASQGLVAS | 284.82 | 0.65 | -0.09 | No | Class I |
| 5163 | 5171 | ASQGLVASI | 390.99 | 0.2387 | -0.03908 | No | Class I |
| 5164 | 5172 | SQGLVASIK | 241.41 | 0.6336 | 0.01501 | No | Class I |
| 5166 | 5174 | GLVASIKNF | 200.9 | 0.4718 | -0.16883 | No | Class I |
| 5167 | 5175 | LVASIKNFK | 51.8 | 0.8148 | -0.16423 | No | Class I |
| 5169 | 5177 | ASIKNFKSV | 298.76 | 0.6853 | -0.34856 | No | Class I |
| 5170 | 5178 | SIKNFKSVL | 256.83 | 0.2414 | -0.28101 | No | Class I |
| 5172 | 5180 | KNFKSVLYY | 49.56 | 0.1643 | -0.31276 | No | Class I |
| 5173 | 5181 | NFKSVLYYQ | 310.28 | 0.8391 | -0.21199 | No | Class I |
| 5176 | 5184 | SVLYYQNNV | 245.31 | 0.5524 | -0.1292 | No | Class I |
| 5177 | 5185 | VLYYQNNVF | 100 | 0.4247 | -0.10515 | No | Class I |
| 5178 | 5186 | LYYQNNVFM | 234.01 | 0.5713 | -0.02691 | No | Class I |
| 5179 | 5187 | YYQNNVFMS | 350.77 | 0.4722 | -0.01535 | No | Class I |
| 5182 | 5190 | NNVFMSEAK | 164.63 | 0.456 | -0.08817 | No | Class I |
| 5184 | 5192 | VFMSEAKCW | 289.31 | 0.5504 | -0.30264 | No | Class I |
| 5185 | 5193 | FMSEAKCWT | 223.84 | 0.3756 | -0.03393 | No | Class I |
| 5187 | 5195 | SEAKCWTET | 251.98 | 0.2635 | 0.04297 | No | Class I |
| 5202 | 5210 | HEFCSQHTM | 17.27 | 0.3759 | -0.23641 | No | Class I |
| 5204 | 5212 | FCSQHTMLV | 322.34 | 0.5596 | -0.2569 | No | Class I |
| 5205 | 5213 | CSQHTMLVK | 196.42 | 0.1398 | -0.11779 | No | Class I |
| 5210 | 5218 | MLVKQGDDY | 268.1 | 0.4433 | -0.25282 | No | Class I |
| 5213 | 5221 | KQGDDYVYL | 218.13 | 0.4041 | 0.08412 | No | Class I |
| 5220 | 5228 | YLPYPDPSR | 311.99 | -0.2672 | -0.10326 | No | Class I |
| 5221 | 5229 | LPYPDPSRI | 283.75 | -0.1931 | -0.11058 | No | Class I |
| 5228 | 5236 | RILGAGCFV | 55.38 | 0.3846 | 0.1234 | No | Class I |
| 5239 | 5247 | IVKTDGTLM | 488.66 | -0.1704 | 0.04884 | No | Class I |
| 5246 | 5254 | LMIERFVSL | 6.57 | -0.0605 | 0.24273 | No | Class I |
| 5247 | 5255 | MIERFVSLA | 370.44 | 0.0515 | 0.09134 | No | Class I |
| 5248 | 5256 | IERFVSLAI | 289.68 | 0.1775 | 0.03257 | No | Class I |
| 5251 | 5259 | FVSLAIDAY | 199.58 | 0.5865 | 0.1401 | No | Class I |
| 5255 | 5263 | AIDAYPLTK | 93.56 | 0.5972 | 0.04585 | No | Class I |
| 5261 | 5269 | LTKHPNQEY | 158.86 | 0.0465 | -0.0936 | No | Class I |
| 5264 | 5272 | HPNQEYADV | 367.26 | 0.6504 | 0.02134 | No | Class I |
| 5267 | 5275 | QEYADVFHL | 286.69 | 0.363 | 0.21633 | No | Class I |
| 5268 | 5276 | EYADVFHLY | 446.21 | 0.4626 | 0.20624 | No | Class I |
| 5269 | 5277 | YADVFHLYL | 115.31 | 0.0159 | 0.18167 | No | Class I |
| 5271 | 5279 | DVFHLYLQY | 86.53 | 0.6321 | -0.02077 | No | Class I |
| 5272 | 5280 | VFHLYLQYI | 62.94 | 0.6558 | -0.11462 | No | Class I |
| 5273 | 5281 | FHLYLQYIR | 477.37 | 0.6091 | -0.05252 | No | Class I |
| 5275 | 5283 | LYLQYIRKL | 141.6 | 0.282 | -0.0808 | No | Class I |
| 5279 | 5287 | YIRKLHDEL | 342.03 | -0.2677 | -0.10333 | No | Class I |
| 5283 | 5291 | LHDELTGHM | 435.54 | 0.2614 | 0.18119 | No | Class I |
| 5286 | 5294 | ELTGHMLDM | 485.68 | 0.4753 | -0.0835 | No | Class I |
| 5287 | 5295 | LTGHMLDMY | 59.47 | 0.2498 | -0.22177 | No | Class I |
| 5289 | 5297 | GHMLDMYSV | 88.16 | 0.4322 | -0.31164 | No | Class I |
| 5290 | 5298 | HMLDMYSVM | 306.93 | 0.5853 | -0.27126 | No | Class I |
| 5291 | 5299 | MLDMYSVML | 6.9 | 0.5626 | -0.39659 | No | Class I |
| 5298 | 5306 | MLTNDNTSR | 326.91 | -0.3109 | -0.0423 | No | Class I |
| 5299 | 5307 | LTNDNTSRY | 25.4 | -0.3497 | -0.05892 | No | Class I |
| 5305 | 5313 | SRYWEPEFY | 305.58 | 0.3034 | 0.46165 | No | Class I |
| 5308 | 5316 | WEPEFYEAM | 93.5 | 0.525 | 0.31503 | No | Class I |
| 5309 | 5317 | EPEFYEAMY | 299.41 | 0.4648 | 0.17137 | No | Class I |
| 5312 | 5320 | FYEAMYTPH | 88.51 | 0.0983 | -0.07633 | No | Class I |
| 5313 | 5321 | YEAMYTPHT | 197.96 | 0.3251 | -0.12152 | No | Class I |
| 5314 | 5322 | EAMYTPHTV | 113.04 | 0.4525 | 0.01662 | No | Class I |
| 5315 | 5323 | AMYTPHTVL | 108.14 | 0.1196 | 0.11439 | No | Class I |
| 5316 | 5324 | MYTPHTVLQ | 499.08 | 0.0086 | 0.09784 | No | Class I |
| 5317 | 5325 | YTPHTVLQA | 298.93 | 0.232 | 0.02857 | No | Class I |
| 5318 | 5326 | TPHTVLQAV | 63.24 | 0.1086 | 0.00442 | No | Class I |
| 5322 | 5330 | VLQAVGACV | 69.01 | 0.1978 | 0.07539 | No | Class I |
| 5323 | 5331 | LQAVGACVL | 383.83 | 0.4814 | 0.10269 | No | Class I |
| 5330 | 5338 | VLCNSQTSL | 255.78 | 0.43 | -0.35805 | No | Class I |
| 5337 | 5345 | SLRCGACIR | 53.58 | 1.6206 | 0.06464 | No | Class I |
| 5344 | 5352 | IRRPFLCCK | 349.19 | 1.1971 | 0.0322 | No | Class I |
| 5357 | 5365 | HVISTSHKL | 396.37 | 0.685 | -0.3399 | No | Class I |
| 5359 | 5367 | ISTSHKLVL | 22.14 | 0.4727 | -0.31061 | No | Class I |
| 5361 | 5369 | TSHKLVLSV | 485.66 | 0.1718 | -0.28446 | No | Class I |
| 5364 | 5372 | KLVLSVNPY | 21.68 | 0.542 | -0.13194 | No | Class I |
| 5365 | 5373 | LVLSVNPYV | 12.85 | 0.567 | -0.14748 | No | Class I |
| 5368 | 5376 | SVNPYVCNA | 70.35 | -0.0733 | -0.02728 | No | Class I |
| 5370 | 5378 | NPYVCNAPG | 420.76 | -0.0176 | 0.00829 | No | Class I |
| 5381 | 5389 | VTDVTQLYL | 98.64 | 0.5789 | -0.03402 | No | Class I |
| 5384 | 5392 | VTQLYLGGM | 146.94 | 0.4741 | -0.0144 | No | Class I |
| 5386 | 5394 | QLYLGGMSY | 88.57 | 0.6598 | -0.19232 | No | Class I |
| 5387 | 5395 | LYLGGMSYY | 139.19 | 0.7868 | -0.24358 | No | Class I |
| 5388 | 5396 | YLGGMSYYC | 178.83 | 0.7913 | -0.28691 | No | Class I |
| 5392 | 5400 | MSYYCKSHK | 20.7 | 0.5665 | -0.38114 | No | Class I |
| 5397 | 5405 | KSHKPPISF | 422.72 | 0.9349 | -0.21208 | No | Class I |
| 5399 | 5407 | HKPPISFPL | 80.55 | 0.2521 | 0.05143 | No | Class I |
| 5405 | 5413 | FPLCANGQV | 456.61 | -0.3461 | -0.06492 | No | Class I |
| 5408 | 5416 | CANGQVFGL | 451.6 | -0.3645 | 0.07666 | No | Class I |
| 5409 | 5417 | ANGQVFGLY | 181.68 | -0.3682 | 0.06696 | No | Class I |
| 5410 | 5418 | NGQVFGLYK | 348.16 | -0.5685 | 0.13832 | No | Class I |
| 5430 | 5438 | FNAIATCDW | 380.5 | 0.6276 | 0.18872 | No | Class I |
| 5438 | 5446 | WTNAGDYIL | 390.78 | 0.2966 | 0.16579 | No | Class I |
| 5445 | 5453 | ILANTCTER | 212.68 | -0.588 | 0.0845 | No | Class I |
| 5446 | 5454 | LANTCTERL | 398.41 | -0.5058 | 0.13574 | No | Class I |
| 5451 | 5459 | TERLKLFAA | 301.69 | -0.0308 | -0.09314 | No | Class I |
| 5453 | 5461 | RLKLFAAET | 203.59 | 0.1566 | 0.16119 | No | Class I |
| 5454 | 5462 | LKLFAAETL | 404.08 | -0.3077 | 0.29631 | No | Class I |
| 5455 | 5463 | KLFAAETLK | 8.24 | -0.2192 | 0.236 | No | Class I |
| 5457 | 5465 | FAAETLKAT | 429.84 | 0.7156 | -0.01833 | No | Class I |
| 5461 | 5469 | TLKATEETF | 84.1 | 0.762 | 0.2086 | No | Class I |
| 5463 | 5471 | KATEETFKL | 171.62 | 0.2766 | 0.22019 | No | Class I |
| 5465 | 5473 | TEETFKLSY | 36.5 | 0.6492 | -0.12346 | No | Class I |
| 5467 | 5475 | ETFKLSYGI | 295.47 | 0.2915 | -0.32885 | No | Class I |
| 5471 | 5479 | LSYGIATVR | 313.09 | 1.696 | 0.25621 | No | Class I |
| 5473 | 5481 | YGIATVREV | 388.36 | 1.4272 | 0.26141 | No | Class I |
| 5477 | 5485 | TVREVLSDR | 67.47 | 0.1002 | 0.02065 | No | Class I |
| 5479 | 5487 | REVLSDREL | 25.41 | 0.4281 | -0.0358 | No | Class I |
| 5481 | 5489 | VLSDRELHL | 93.65 | 1.5809 | 0.12281 | No | Class I |
| 5485 | 5493 | RELHLSWEV | 186.39 | 2.2601 | 0.10786 | No | Class I |
| 5489 | 5497 | LSWEVGKPR | 54.91 | 0.3276 | 0.05627 | No | Class I |
| 5497 | 5505 | RPPLNRNYV | 110.18 | -0.9881 | 0.02004 | No | Class I |
| 5501 | 5509 | NRNYVFTGY | 397.08 | 1.1158 | 0.19714 | No | Class I |
| 5502 | 5510 | RNYVFTGYR | 272.1 | 0.9465 | 0.21732 | No | Class I |
| 5503 | 5511 | NYVFTGYRV | 305.94 | 1.0902 | 0.22802 | No | Class I |
| 5504 | 5512 | YVFTGYRVT | 223.84 | 0.8954 | 0.17438 | No | Class I |
| 5505 | 5513 | VFTGYRVTK | 446.81 | 0.7603 | 0.14934 | No | Class I |
| 5508 | 5516 | GYRVTKNSK | 42.59 | 0.999 | -0.20898 | No | Class I |
| 5509 | 5517 | YRVTKNSKV | 64.7 | 0.485 | -0.42925 | No | Class I |
| 5511 | 5519 | VTKNSKVQI | 136.25 | 0.5717 | -0.47345 | No | Class I |
| 5516 | 5524 | KVQIGEYTF | 130.15 | 0.8369 | 0.24313 | No | Class I |
| 5518 | 5526 | QIGEYTFEK | 481.3 | 0.3447 | 0.30199 | No | Class I |
| 5533 | 5541 | VVYRGTTTY | 59.53 | 0.4005 | 0.17586 | No | Class I |
| 5535 | 5543 | YRGTTTYKL | 444.2 | 0.7167 | -0.00472 | No | Class I |
| 5537 | 5545 | GTTTYKLNV | 267.2 | 0.8706 | -0.16808 | No | Class I |
| 5540 | 5548 | TYKLNVGDY | 86.33 | 0.8651 | -0.00704 | No | Class I |
| 5548 | 5556 | YFVLTSHTV | 84.49 | 0.5996 | -0.06571 | No | Class I |
| 5549 | 5557 | FVLTSHTVM | 210.59 | 0.5299 | -0.03831 | No | Class I |
| 5551 | 5559 | LTSHTVMPL | 176.13 | 0.4828 | -0.09917 | No | Class I |
| 5552 | 5560 | TSHTVMPLS | 411.89 | 0.7043 | -0.09138 | No | Class I |
| 5553 | 5561 | SHTVMPLSA | 328.58 | 0.5893 | -0.23332 | No | Class I |
| 5555 | 5563 | TVMPLSAPT | 426.56 | 0.1454 | -0.20815 | No | Class I |
| 5556 | 5564 | VMPLSAPTL | 198.98 | 0.1375 | -0.12571 | No | Class I |
| 5557 | 5565 | MPLSAPTLV | 256.31 | 0.1072 | -0.11613 | No | Class I |
| 5564 | 5572 | LVPQEHYVR | 261.37 | 0.0154 | 0.02879 | No | Class I |
| 5565 | 5573 | VPQEHYVRI | 181.29 | -0.2532 | 0.15625 | No | Class I |
| 5568 | 5576 | EHYVRITGL | 231.82 | 0.5647 | 0.26858 | No | Class I |
| 5569 | 5577 | HYVRITGLY | 297.59 | 0.54 | 0.25374 | No | Class I |
| 5572 | 5580 | RITGLYPTL | 397.02 | 0.4786 | 0.04574 | No | Class I |
| 5575 | 5583 | GLYPTLNIS | 221.38 | 0.8311 | 0.0873 | No | Class I |
| 5582 | 5590 | ISDEFSSNV | 111.1 | -0.0752 | -0.07718 | No | Class I |
| 5584 | 5592 | DEFSSNVAN | 223.15 | -0.0918 | -0.23796 | No | Class I |
| 5585 | 5593 | EFSSNVANY | 245.04 | 0.2036 | -0.15837 | No | Class I |
| 5586 | 5594 | FSSNVANYQ | 351.22 | 0.2651 | 0.0092 | No | Class I |
| 5587 | 5595 | SSNVANYQK | 121.39 | 0.4591 | 0.00065 | No | Class I |
| 5590 | 5598 | VANYQKVGM | 247.81 | 1.3083 | -0.26698 | No | Class I |
| 5593 | 5601 | YQKVGMQKY | 27.94 | 0.2265 | -0.38452 | No | Class I |
| 5596 | 5604 | VGMQKYSTL | 57.59 | 0.896 | -0.50398 | No | Class I |
| 5598 | 5606 | MQKYSTLQG | 252.73 | 0.1084 | -0.27532 | No | Class I |
| 5613 | 5621 | SHFAIGLAL | 88.76 | 1.3872 | 0.25237 | No | Class I |
| 5615 | 5623 | FAIGLALYY | 485.8 | 1.189 | 0.09181 | No | Class I |
| 5618 | 5626 | GLALYYPSA | 56.13 | 0.2615 | -0.11156 | No | Class I |
| 5619 | 5627 | LALYYPSAR | 68.49 | 0.0287 | -0.13812 | No | Class I |
| 5620 | 5628 | ALYYPSARI | 394.6 | -0.1319 | -0.10819 | No | Class I |
| 5621 | 5629 | LYYPSARIV | 87.04 | -0.0593 | -0.01519 | No | Class I |
| 5622 | 5630 | YYPSARIVY | 76.66 | 0.4008 | 0.05319 | No | Class I |
| 5623 | 5631 | YPSARIVYT | 282.63 | 0.6329 | 0.19403 | No | Class I |
| 5627 | 5635 | RIVYTACSH | 464.98 | 0.0602 | -0.05785 | No | Class I |
| 5628 | 5636 | IVYTACSHA | 125.81 | -0.0283 | -0.09551 | No | Class I |
| 5629 | 5637 | VYTACSHAA | 156.36 | 0.0146 | -0.1061 | No | Class I |
| 5630 | 5638 | YTACSHAAV | 87.99 | 0.1331 | -0.11632 | No | Class I |
| 5633 | 5641 | CSHAAVDAL | 469.46 | 0.0818 | 0.16841 | No | Class I |
| 5636 | 5644 | AAVDALCEK | 65.45 | 0.6626 | 0.07638 | No | Class I |
| 5639 | 5647 | DALCEKALK | 202.44 | 0.7719 | -0.13681 | No | Class I |
| 5640 | 5648 | ALCEKALKY | 381.85 | 0.8104 | -0.22528 | No | Class I |
| 5645 | 5653 | ALKYLPIDK | 99.73 | 0.5198 | 0.03032 | No | Class I |
| 5649 | 5657 | LPIDKCSRI | 426.29 | -0.4671 | -0.30461 | No | Class I |
| 5653 | 5661 | KCSRIIPAR | 81.2 | -0.0971 | 0.26676 | No | Class I |
| 5654 | 5662 | CSRIIPARA | 70.44 | -0.4581 | 0.33314 | No | Class I |
| 5656 | 5664 | RIIPARARV | 79.11 | 0.0096 | 0.18212 | No | Class I |
| 5658 | 5666 | IPARARVEC | 98.38 | 1.9273 | 0.24494 | No | Class I |
| 5663 | 5671 | RVECFDKFK | 217.91 | 0.9777 | -0.00047 | No | Class I |
| 5671 | 5679 | KVNSTLEQY | 334.73 | 0.3625 | -0.12439 | No | Class I |
| 5673 | 5681 | NSTLEQYVF | 448.64 | -0.2794 | 0.0109 | No | Class I |
| 5676 | 5684 | LEQYVFCTV | 278.07 | 0.0992 | 0.08626 | No | Class I |
| 5682 | 5690 | CTVNALPET | 484.77 | 0.4119 | 0.08369 | No | Class I |
| 5686 | 5694 | ALPETTADI | 319.11 | 0.4539 | 0.21747 | No | Class I |
| 5689 | 5697 | ETTADIVVF | 179.18 | 0.1754 | 0.25781 | No | Class I |
| 5692 | 5700 | ADIVVFDEI | 165.7 | 0.1343 | 0.31236 | No | Class I |
| 5694 | 5702 | IVVFDEISM | 494.95 | 0.6349 | 0.26271 | No | Class I |
| 5695 | 5703 | VVFDEISMA | 62.91 | 0.7441 | 0.04088 | No | Class I |
| 5698 | 5706 | DEISMATNY | 9.46 | 0.4465 | -0.22846 | No | Class I |
| 5700 | 5708 | ISMATNYDL | 498.3 | 0.9613 | 0.02392 | No | Class I |
| 5702 | 5710 | MATNYDLSV | 141.02 | 0.8965 | -0.08265 | No | Class I |
| 5703 | 5711 | ATNYDLSVV | 123.84 | 1.4063 | -0.11016 | No | Class I |
| 5708 | 5716 | LSVVNARLR | 261.52 | 1.8592 | 0.12267 | No | Class I |
| 5709 | 5717 | SVVNARLRA | 414.01 | 1.3836 | 0.11459 | No | Class I |
| 5710 | 5718 | VVNARLRAK | 53.32 | 1.933 | 0.14377 | No | Class I |
| 5714 | 5722 | RLRAKHYVY | 237.22 | 1.4472 | -0.10238 | No | Class I |
| 5715 | 5723 | LRAKHYVYI | 36.07 | 1.651 | -0.1436 | No | Class I |
| 5716 | 5724 | RAKHYVYIG | 76.33 | 1.8539 | 0.07245 | No | Class I |
| 5719 | 5727 | HYVYIGDPA | 479.43 | 0.9151 | 0.18342 | No | Class I |
| 5721 | 5729 | VYIGDPAQL | 207.4 | 0.514 | 0.0538 | No | Class I |
| 5727 | 5735 | AQLPAPRTL | 378.14 | -0.356 | 0.07926 | No | Class I |
| 5728 | 5736 | QLPAPRTLL | 86.39 | -0.7166 | 0.09997 | No | Class I |
| 5729 | 5737 | LPAPRTLLT | 310.55 | -0.2838 | 0.07264 | No | Class I |
| 5731 | 5739 | APRTLLTKG | 433.42 | -0.5823 | -0.05862 | No | Class I |
| 5733 | 5741 | RTLLTKGTL | 172.93 | -0.369 | -0.12868 | No | Class I |
| 5741 | 5749 | LEPEYFNSV | 415.45 | 1.067 | 0.10163 | No | Class I |
| 5744 | 5752 | EYFNSVCRL | 145.62 | 0.2972 | -0.10601 | No | Class I |
| 5745 | 5753 | YFNSVCRLM | 114.84 | 0.2627 | -0.14192 | No | Class I |
| 5746 | 5754 | FNSVCRLMK | 358.52 | 0.0303 | -0.1279 | No | Class I |
| 5752 | 5760 | LMKTIGPDM | 144.29 | 0.0846 | 0.13416 | No | Class I |
| 5754 | 5762 | KTIGPDMFL | 74.56 | 0.0875 | 0.00758 | No | Class I |
| 5759 | 5767 | DMFLGTCRR | 421.31 | 0.7393 | 0.08112 | No | Class I |
| 5768 | 5776 | CPAEIVDTV | 194.7 | 0.4414 | 0.32331 | No | Class I |
| 5770 | 5778 | AEIVDTVSA | 117.76 | 0.7203 | 0.08106 | No | Class I |
| 5771 | 5779 | EIVDTVSAL | 27.73 | 0.656 | -0.00438 | No | Class I |
| 5772 | 5780 | IVDTVSALV | 69.15 | 0.254 | -0.04273 | No | Class I |
| 5776 | 5784 | VSALVYDNK | 410.04 | 0.852 | 0.0532 | No | Class I |
| 5777 | 5785 | SALVYDNKL | 288.04 | 0.6358 | -0.07624 | No | Class I |
| 5778 | 5786 | ALVYDNKLK | 450.43 | 0.5528 | -0.16329 | No | Class I |
| 5791 | 5799 | KSAQCFKMF | 383.36 | 0.2166 | -0.33076 | No | Class I |
| 5792 | 5800 | SAQCFKMFY | 184.74 | 0.3763 | -0.26065 | No | Class I |
| 5793 | 5801 | AQCFKMFYK | 35.14 | 0.0983 | -0.17836 | No | Class I |
| 5797 | 5805 | KMFYKGVIT | 320.96 | 0.062 | -0.03122 | No | Class I |
| 5798 | 5806 | MFYKGVITH | 357.2 | -0.1232 | -0.01134 | No | Class I |
| 5804 | 5812 | ITHDVSSAI | 275.09 | -0.0211 | -0.19947 | No | Class I |
| 5809 | 5817 | SSAINRPQI | 376.39 | -0.323 | 0.112 | No | Class I |
| 5811 | 5819 | AINRPQIGV | 260.22 | -0.0486 | 0.06226 | No | Class I |
| 5818 | 5826 | GVVREFLTR | 230.03 | -1.8051 | 0.2865 | No | Class I |
| 5821 | 5829 | REFLTRNPA | 236.09 | -1.233 | 0.10142 | No | Class I |
| 5823 | 5831 | FLTRNPAWR | 429.68 | -0.108 | 0.21038 | No | Class I |
| 5824 | 5832 | LTRNPAWRK | 123.88 | -0.5822 | 0.2535 | No | Class I |
| 5827 | 5835 | NPAWRKAVF | 21.31 | 0.2299 | 0.14013 | No | Class I |
| 5829 | 5837 | AWRKAVFIS | 230.78 | 0.6381 | 0.05332 | No | Class I |
| 5831 | 5839 | RKAVFISPY | 211.39 | 0.9219 | 0.14742 | No | Class I |
| 5837 | 5845 | SPYNSQNAV | 29.3 | 0.635 | -0.26045 | No | Class I |
| 5840 | 5848 | NSQNAVASK | 147.52 | 0.6232 | -0.03079 | No | Class I |
| 5841 | 5849 | SQNAVASKI | 206.93 | 0.2038 | -0.15132 | No | Class I |
| 5844 | 5852 | AVASKILGL | 57.52 | 0.4971 | -0.22805 | No | Class I |
| 5846 | 5854 | ASKILGLPT | 144.15 | 0.1317 | 0.06918 | No | Class I |
| 5849 | 5857 | ILGLPTQTV | 119.56 | 0.4344 | -0.0495 | No | Class I |
| 5859 | 5867 | SSQGSEYDY | 488.95 | 1.0337 | -0.06051 | No | Class I |
| 5860 | 5868 | SQGSEYDYV | 84.53 | 0.915 | -0.04489 | No | Class I |
| 5863 | 5871 | SEYDYVIFT | 105.72 | 0.8851 | 0.2371 | No | Class I |
| 5876 | 5884 | TAHSCNVNR | 91.16 | -0.3503 | -0.1835 | No | Class I |
| 5884 | 5892 | RFNVAITRA | 164.26 | 0.4438 | 0.26582 | No | Class I |
| 5885 | 5893 | FNVAITRAK | 90.57 | 1.0587 | 0.28545 | No | Class I |
| 5889 | 5897 | ITRAKVGIL | 91.33 | 0.9073 | -0.00861 | No | Class I |
| 5891 | 5899 | RAKVGILCI | 72.73 | 2.3668 | 0.08896 | No | Class I |
| 5898 | 5906 | CIMSDRDLY | 48.81 | 1.235 | -0.14091 | No | Class I |
| 5900 | 5908 | MSDRDLYDK | 471.18 | 0.1866 | 0.08028 | No | Class I |
| 5904 | 5912 | DLYDKLQFT | 350.55 | 1.1435 | -0.22868 | No | Class I |
| 5906 | 5914 | YDKLQFTSL | 208.63 | 1.2778 | -0.14766 | No | Class I |
| 5908 | 5916 | KLQFTSLEI | 95.1 | 2.2084 | 0.01141 | No | Class I |
| 5910 | 5918 | QFTSLEIPR | 126.26 | 0.4368 | 0.03542 | No | Class I |
| 5911 | 5919 | FTSLEIPRR | 309.51 | 0.7888 | 0.1788 | No | Class I |
| 5914 | 5922 | LEIPRRNVA | 422.07 | 0.0903 | 0.14982 | No | Class I |
| 5916 | 5924 | IPRRNVATL | 4.24 | -0.1191 | 0.15714 | No | Class I |
| 5924 | 5932 | LQAENVTGL | 122.38 | 0.8291 | 0.19857 | No | Class I |
| 5931 | 5939 | GLFKDCSKV | 65.7 | -0.9014 | -0.47377 | No | Class I |
| 5932 | 5940 | LFKDCSKVI | 203.11 | -0.9927 | -0.41379 | No | Class I |
| 5938 | 5946 | KVITGLHPT | 195.33 | 0.6717 | 0.12564 | No | Class I |
| 5942 | 5950 | GLHPTQAPT | 225.78 | 0.9779 | -0.04536 | No | Class I |
| 5944 | 5952 | HPTQAPTHL | 109 | 0.352 | -0.02464 | No | Class I |
| 5946 | 5954 | TQAPTHLSV | 88.09 | 0.5627 | -0.03623 | No | Class I |
| 5950 | 5958 | THLSVDTKF | 396.25 | 1.1249 | -0.20223 | No | Class I |
| 5951 | 5959 | HLSVDTKFK | 270.55 | 1.372 | -0.06762 | No | Class I |
| 5957 | 5965 | KFKTEGLCV | 296.86 | 1.7141 | 0.0576 | No | Class I |
| 5962 | 5970 | GLCVDIPGI | 105.96 | -0.8017 | 0.18136 | No | Class I |
| 5964 | 5972 | CVDIPGIPK | 384.16 | -0.7448 | 0.26806 | No | Class I |
| 5970 | 5978 | IPKDMTYRR | 347.51 | -0.3256 | -0.15502 | No | Class I |
| 5972 | 5980 | KDMTYRRLI | 193.91 | -0.4329 | 0.06438 | No | Class I |
| 5974 | 5982 | MTYRRLISM | 304.97 | 0.2626 | 0.1065 | No | Class I |
| 5975 | 5983 | TYRRLISMM | 89.67 | -0.1785 | -0.05886 | No | Class I |
| 5977 | 5985 | RRLISMMGF | 211.47 | -0.0628 | -0.32448 | No | Class I |
| 5978 | 5986 | RLISMMGFK | 6.16 | 0.6827 | -0.36257 | No | Class I |
| 5979 | 5987 | LISMMGFKM | 352.99 | 0.7827 | -0.3967 | No | Class I |
| 5981 | 5989 | SMMGFKMNY | 24.64 | 1.3098 | -0.26388 | No | Class I |
| 5983 | 5991 | MGFKMNYQV | 216.45 | 1.4042 | -0.42689 | No | Class I |
| 5986 | 5994 | KMNYQVNGY | 124.33 | 0.3801 | -0.06542 | No | Class I |
| 5989 | 5997 | YQVNGYPNM | 342.73 | 0.5166 | 0.02327 | No | Class I |
| 5993 | 6001 | GYPNMFITR | 120.76 | 0.0657 | 0.06409 | No | Class I |
| 5996 | 6004 | NMFITREEA | 424.57 | 0.3638 | 0.40144 | No | Class I |
| 5997 | 6005 | MFITREEAI | 63.31 | 0.7367 | 0.33427 | No | Class I |
| 5998 | 6006 | FITREEAIR | 142.84 | -0.4069 | 0.36721 | No | Class I |
| 6000 | 6008 | TREEAIRHV | 392.82 | -0.3717 | 0.35921 | No | Class I |
| 6003 | 6011 | EAIRHVRAW | 310.92 | -0.4405 | 0.23218 | No | Class I |
| 6004 | 6012 | AIRHVRAWI | 113.46 | -0.334 | 0.30071 | No | Class I |
| 6006 | 6014 | RHVRAWIGF | 268.6 | -0.1051 | 0.44421 | No | Class I |
| 6015 | 6023 | DVEGCHATR | 70 | 0.1004 | 0.10025 | No | Class I |
| 6021 | 6029 | ATREAVGTN | 274.6 | 0.1565 | 0.24579 | No | Class I |
| 6022 | 6030 | TREAVGTNL | 355.65 | 0.1332 | 0.17295 | No | Class I |
| 6024 | 6032 | EAVGTNLPL | 493.28 | 0.1546 | 0.06337 | No | Class I |
| 6030 | 6038 | LPLQLGFST | 476.7 | 1.8539 | -0.09692 | No | Class I |
| 6034 | 6042 | LGFSTGVNL | 330.79 | 0.8852 | -0.02771 | No | Class I |
| 6036 | 6044 | FSTGVNLVA | 156.33 | 0.4562 | 0.09557 | No | Class I |
| 6037 | 6045 | STGVNLVAV | 261.78 | 1.0015 | 0.0935 | No | Class I |
| 6041 | 6049 | NLVAVPTGY | 122.39 | 0.8113 | 0.13509 | No | Class I |
| 6060 | 6068 | RVSAKPPPG | 78.73 | 0.7584 | -0.25061 | No | Class I |
| 6069 | 6077 | DQFKHLIPL | 233.87 | 0.1529 | -0.0521 | No | Class I |
| 6070 | 6078 | QFKHLIPLM | 319.64 | 0.8288 | 0.06119 | No | Class I |
| 6072 | 6080 | KHLIPLMYK | 192.78 | 1.0967 | -0.04128 | No | Class I |
| 6078 | 6086 | MYKGLPWNV | 176.93 | 0.5946 | 0.12602 | No | Class I |
| 6081 | 6089 | GLPWNVVRI | 390.53 | 0.6233 | 0.31693 | No | Class I |
| 6086 | 6094 | VVRIKIVQM | 243.83 | 0.7244 | 0.03316 | No | Class I |
| 6087 | 6095 | VRIKIVQML | 45.95 | 0.6751 | -0.2057 | No | Class I |
| 6088 | 6096 | RIKIVQMLS | 16.53 | 0.362 | -0.1596 | No | Class I |
| 6092 | 6100 | VQMLSDTLK | 258.54 | -0.1123 | -0.1821 | No | Class I |
| 6094 | 6102 | MLSDTLKNL | 52.66 | 0.0928 | -0.1898 | No | Class I |
| 6097 | 6105 | DTLKNLSDR | 123.26 | -0.4996 | -0.364 | No | Class I |
| 6098 | 6106 | TLKNLSDRV | 49.08 | -0.0959 | -0.19408 | No | Class I |
| 6102 | 6110 | LSDRVVFVL | 482.48 | 0.759 | 0.26126 | No | Class I |
| 6105 | 6113 | RVVFVLWAH | 343.68 | 0.651 | 0.37076 | No | Class I |
| 6107 | 6115 | VFVLWAHGF | 132.94 | -0.6723 | 0.30187 | No | Class I |
| 6109 | 6117 | VLWAHGFEL | 4.54 | -0.422 | 0.33197 | No | Class I |
| 6113 | 6121 | HGFELTSMK | 401 | 0.8584 | -0.07773 | No | Class I |
| 6114 | 6122 | GFELTSMKY | 69.9 | 1.4309 | -0.37079 | No | Class I |
| 6115 | 6123 | FELTSMKYF | 67.65 | 0.9955 | -0.4751 | No | Class I |
| 6117 | 6125 | LTSMKYFVK | 158.88 | -0.0535 | -0.32096 | No | Class I |
| 6118 | 6126 | TSMKYFVKI | 191.15 | 0.0931 | -0.25856 | No | Class I |
| 6119 | 6127 | SMKYFVKIG | 486.48 | 0.9285 | -0.0251 | No | Class I |
| 6122 | 6130 | YFVKIGPER | 199.89 | 0.7119 | 0.00704 | No | Class I |
| 6126 | 6134 | IGPERTCCL | 124.04 | 1.2961 | 0.10709 | No | Class I |
| 6130 | 6138 | RTCCLCDRR | 45.43 | 3.1069 | -0.08434 | No | Class I |
| 6137 | 6145 | RRATCFSTA | 405.75 | 1.3673 | -0.00748 | No | Class I |
| 6141 | 6149 | CFSTASDTY | 102.37 | 0.3308 | -0.09087 | No | Class I |
| 6144 | 6152 | TASDTYACW | 77.91 | 0.9885 | 0.00446 | No | Class I |
| 6152 | 6160 | WHHSIGFDY | 135.66 | 1.0145 | 0.11729 | No | Class I |
| 6153 | 6161 | HHSIGFDYV | 199.28 | 1.3237 | 0.23998 | No | Class I |
| 6154 | 6162 | HSIGFDYVY | 403.13 | 1.0882 | 0.23318 | No | Class I |
| 6157 | 6165 | GFDYVYNPF | 88.47 | 0.5864 | 0.02826 | No | Class I |
| 6158 | 6166 | FDYVYNPFM | 483.07 | 0.5967 | 0.08969 | No | Class I |
| 6159 | 6167 | DYVYNPFMI | 189.1 | 0.751 | -0.01086 | No | Class I |
| 6161 | 6169 | VYNPFMIDV | 90.51 | 0.5367 | 0.06072 | No | Class I |
| 6164 | 6172 | PFMIDVQQW | 473.83 | 0.2171 | -0.02806 | No | Class I |
| 6165 | 6173 | FMIDVQQWG | 354.54 | -0.2522 | 0.02834 | No | Class I |
| 6170 | 6178 | QQWGFTGNL | 73.57 | 1.0003 | 0.28136 | No | Class I |
| 6177 | 6185 | NLQSNHDLY | 89.5 | 0.4574 | -0.16768 | No | Class I |
| 6186 | 6194 | CQVHGNAHV | 98.75 | -0.0914 | 0.12478 | No | Class I |
| 6192 | 6200 | AHVASCDAI | 347.01 | -0.104 | -0.1175 | No | Class I |
| 6193 | 6201 | HVASCDAIM | 438.83 | -0.1536 | -0.07461 | No | Class I |
| 6195 | 6203 | ASCDAIMTR | 99.63 | 0.3756 | 0.04268 | No | Class I |
| 6199 | 6207 | AIMTRCLAV | 47.18 | 0.397 | -0.00479 | No | Class I |
| 6200 | 6208 | IMTRCLAVH | 436.89 | 0.2307 | 0.05888 | No | Class I |
| 6201 | 6209 | MTRCLAVHE | 343.07 | 0.0622 | 0.04232 | No | Class I |
| 6204 | 6212 | CLAVHECFV | 59.67 | 0.2627 | 0.20289 | No | Class I |
| 6205 | 6213 | LAVHECFVK | 295.9 | -0.6341 | 0.21562 | No | Class I |
| 6206 | 6214 | AVHECFVKR | 132.82 | -0.6282 | 0.07779 | No | Class I |
| 6211 | 6219 | FVKRVDWTI | 197.25 | 1.9477 | 0.25278 | No | Class I |
| 6213 | 6221 | KRVDWTIEY | 315.42 | 2.4429 | 0.45878 | No | Class I |
| 6215 | 6223 | VDWTIEYPI | 322.73 | 0.982 | 0.32521 | No | Class I |
| 6220 | 6228 | EYPIIGDEL | 350.9 | 0.2055 | 0.36904 | No | Class I |
| 6226 | 6234 | DELKINAAC | 435.74 | 1.1773 | -0.04121 | No | Class I |
| 6227 | 6235 | ELKINAACR | 109.39 | 1.7112 | 0.09597 | No | Class I |
| 6229 | 6237 | KINAACRKV | 413.65 | 1.3888 | -0.0577 | No | Class I |
| 6236 | 6244 | KVQHMVVKA | 151.29 | 0.3747 | -0.22835 | No | Class I |
| 6238 | 6246 | QHMVVKAAL | 498.17 | -0.0885 | -0.12238 | No | Class I |
| 6239 | 6247 | HMVVKAALL | 180.37 | 0.3533 | -0.09169 | No | Class I |
| 6240 | 6248 | MVVKAALLA | 252.69 | 0.6124 | -0.14451 | No | Class I |
| 6243 | 6251 | KAALLADKF | 97.62 | 0.3822 | -0.07971 | No | Class I |
| 6245 | 6253 | ALLADKFPV | 4.7 | 0.0471 | -0.05331 | No | Class I |
| 6246 | 6254 | LLADKFPVL | 13.31 | 0.0845 | -0.05002 | No | Class I |
| 6253 | 6261 | VLHDIGNPK | 75.79 | -0.1952 | 0.18238 | No | Class I |
| 6256 | 6264 | DIGNPKAIK | 333.2 | 0.0784 | -0.09853 | No | Class I |
| 6261 | 6269 | KAIKCVPQA | 256.19 | 0.5994 | -0.26448 | No | Class I |
| 6268 | 6276 | QADVEWKFY | 241.47 | 2.0416 | 0.24115 | No | Class I |
| 6278 | 6286 | AQPCSDKAY | 142.35 | -0.3699 | -0.35721 | No | Class I |
| 6284 | 6292 | KAYKIEELF | 437.34 | 0.3352 | 0.08367 | No | Class I |
| 6285 | 6293 | AYKIEELFY | 161.69 | 0.5856 | 0.31471 | No | Class I |
| 6287 | 6295 | KIEELFYSY | 76.22 | 0.533 | 0.13287 | No | Class I |
| 6289 | 6297 | EELFYSYAT | 71.62 | 0.1387 | -0.02539 | No | Class I |
| 6293 | 6301 | YSYATHSDK | 129.37 | 0.4035 | -0.02024 | No | Class I |
| 6294 | 6302 | SYATHSDKF | 119.45 | 0.0317 | -0.17975 | No | Class I |
| 6301 | 6309 | KFTDGVCLF | 386.75 | -0.003 | 0.0548 | No | Class I |
| 6302 | 6310 | FTDGVCLFW | 378.51 | 0.2664 | 0.08979 | No | Class I |
| 6308 | 6316 | LFWNCNVDR | 62.89 | -1.1828 | 0.0546 | No | Class I |
| 6309 | 6317 | FWNCNVDRY | 349.09 | -1.4948 | 0.02517 | No | Class I |
| 6316 | 6324 | RYPANSIVC | 406.13 | 0.0763 | 0.01018 | No | Class I |
| 6317 | 6325 | YPANSIVCR | 363.71 | 0.17 | -0.02629 | No | Class I |
| 6321 | 6329 | SIVCRFDTR | 74.56 | 1.5888 | 0.16115 | No | Class I |
| 6328 | 6336 | TRVLSNLNL | 174 | 0.4533 | -0.17809 | No | Class I |
| 6336 | 6344 | LPGCDGGSL | 94.35 | -0.5856 | -0.05781 | No | Class I |
| 6343 | 6351 | SLYVNKHAF | 487.64 | -0.1773 | -0.1188 | No | Class I |
| 6344 | 6352 | LYVNKHAFH | 333.81 | -0.2771 | -0.07124 | No | Class I |
| 6348 | 6356 | KHAFHTPAF | 275.12 | -0.3517 | 0.21204 | No | Class I |
| 6353 | 6361 | TPAFDKSAF | 46.88 | -0.3817 | -0.16766 | No | Class I |
| 6356 | 6364 | FDKSAFVNL | 473.47 | 0.2124 | -0.05711 | No | Class I |
| 6359 | 6367 | SAFVNLKQL | 301.99 | 0.932 | -0.18688 | No | Class I |
| 6361 | 6369 | FVNLKQLPF | 182.05 | 1.8441 | -0.34814 | No | Class I |
| 6363 | 6371 | NLKQLPFFY | 19.47 | 1.4745 | -0.0406 | No | Class I |
| 6365 | 6373 | KQLPFFYYS | 431.47 | 1.0635 | 0.20416 | No | Class I |
| 6367 | 6375 | LPFFYYSDS | 139.46 | 0.3583 | 0.02206 | No | Class I |
| 6369 | 6377 | FFYYSDSPC | 98.27 | 0.3667 | -0.29124 | No | Class I |
| 6382 | 6390 | KQVVSDIDY | 134.67 | 0.674 | 0.04 | No | Class I |
| 6383 | 6391 | QVVSDIDYV | 235.32 | 1.0205 | 0.01037 | No | Class I |
| 6385 | 6393 | VSDIDYVPL | 270.77 | 1.9966 | 0.1876 | No | Class I |
| 6390 | 6398 | YVPLKSATC | 320.06 | 0.321 | -0.32479 | No | Class I |
| 6391 | 6399 | VPLKSATCI | 406.82 | 0.6097 | -0.34361 | No | Class I |
| 6399 | 6407 | ITRCNLGGA | 72.22 | 0.6338 | -0.00579 | No | Class I |
| 6410 | 6418 | RHHANEYRL | 428.95 | -0.2987 | 0.16494 | No | Class I |
| 6411 | 6419 | HHANEYRLY | 76.81 | -0.2673 | 0.13741 | No | Class I |
| 6412 | 6420 | HANEYRLYL | 419.34 | -0.3655 | 0.13225 | No | Class I |
| 6414 | 6422 | NEYRLYLDA | 342.71 | -0.4382 | 0.0402 | No | Class I |
| 6415 | 6423 | EYRLYLDAY | 303.94 | -0.3052 | 0.03318 | No | Class I |
| 6417 | 6425 | RLYLDAYNM | 113.12 | -0.3424 | 0.03917 | No | Class I |
| 6418 | 6426 | LYLDAYNMM | 216.67 | 0.0265 | -0.05472 | No | Class I |
| 6419 | 6427 | YLDAYNMMI | 221.43 | 0.1287 | -0.21392 | No | Class I |
| 6421 | 6429 | DAYNMMISA | 116.33 | 0.3353 | -0.32835 | No | Class I |
| 6423 | 6431 | YNMMISAGF | 499.3 | 0.8997 | -0.20701 | No | Class I |
| 6425 | 6433 | MMISAGFSL | 6.83 | 1.0248 | -0.05113 | No | Class I |
| 6426 | 6434 | MISAGFSLW | 274.29 | 0.5869 | -0.01723 | No | Class I |
| 6427 | 6435 | ISAGFSLWV | 235.46 | 0.3875 | 0.12513 | No | Class I |
| 6428 | 6436 | SAGFSLWVY | 150.84 | 0.0157 | 0.16832 | No | Class I |
| 6429 | 6437 | AGFSLWVYK | 119.75 | 0.1667 | 0.10192 | No | Class I |
| 6431 | 6439 | FSLWVYKQF | 27.06 | 0.2513 | 0.00633 | No | Class I |
| 6434 | 6442 | WVYKQFDTY | 171.85 | 0.1797 | -0.1794 | No | Class I |
| 6436 | 6444 | YKQFDTYNL | 68.4 | 0.3699 | 0.13144 | No | Class I |
| 6437 | 6445 | KQFDTYNLW | 304.33 | 0.0079 | 0.0827 | No | Class I |
| 6440 | 6448 | DTYNLWNTF | 76.44 | -0.0656 | 0.20722 | No | Class I |
| 6442 | 6450 | YNLWNTFTR | 70.62 | -0.4567 | 0.37101 | No | Class I |
| 6443 | 6451 | NLWNTFTRL | 31.07 | -0.5457 | 0.27639 | No | Class I |
| 6446 | 6454 | NTFTRLQSL | 50.24 | -0.1929 | -0.0774 | No | Class I |
| 6451 | 6459 | LQSLENVAF | 417.24 | 1.0621 | 0.08425 | No | Class I |
| 6454 | 6462 | LENVAFNVV | 418.5 | 0.9987 | 0.2064 | No | Class I |
| 6456 | 6464 | NVAFNVVNK | 174.09 | 1.1634 | 0.19412 | No | Class I |
| 6470 | 6478 | QQGEVPVSI | 436.79 | 0.6627 | 0.07969 | No | Class I |
| 6474 | 6482 | VPVSIINNT | 277.7 | 0.5553 | 0.09257 | No | Class I |
| 6476 | 6484 | VSIINNTVY | 100.1 | 0.3558 | 0.22161 | No | Class I |
| 6477 | 6485 | SIINNTVYT | 385.05 | -0.065 | 0.09961 | No | Class I |
| 6478 | 6486 | IINNTVYTK | 43.31 | 0.0429 | 0.08761 | No | Class I |
| 6482 | 6490 | TVYTKVDGV | 369.11 | 0.0843 | -0.09476 | No | Class I |
| 6486 | 6494 | KVDGVDVEL | 479.1 | 1.1329 | 0.19572 | No | Class I |
| 6495 | 6503 | FENKTTLPV | 482.38 | 0.9149 | -0.1606 | No | Class I |
| 6498 | 6506 | KTTLPVNVA | 142.19 | 0.5363 | 0.04816 | No | Class I |
| 6499 | 6507 | TTLPVNVAF | 108.4 | 0.9395 | 0.07705 | No | Class I |
| 6501 | 6509 | LPVNVAFEL | 155.18 | 1.2581 | 0.24122 | No | Class I |
| 6502 | 6510 | PVNVAFELW | 259.37 | 0.8336 | 0.26576 | No | Class I |
| 6504 | 6512 | NVAFELWAK | 229.45 | 0.6942 | 0.42736 | No | Class I |
| 6507 | 6515 | FELWAKRNI | 380.95 | 0.6009 | 0.09429 | No | Class I |
| 6508 | 6516 | ELWAKRNIK | 331.3 | 0.6673 | 0.02229 | No | Class I |
| 6510 | 6518 | WAKRNIKPV | 94.59 | 2.1335 | -0.08742 | No | Class I |
| 6513 | 6521 | RNIKPVPEV | 353.72 | 1.5495 | -0.0966 | No | Class I |
| 6514 | 6522 | NIKPVPEVK | 111.04 | 2.3203 | 0.05722 | No | Class I |
| 6516 | 6524 | KPVPEVKIL | 262.38 | 0.6634 | 0.03436 | No | Class I |
| 6523 | 6531 | ILNNLGVDI | 131.68 | 0.0034 | 0.06029 | No | Class I |
| 6532 | 6540 | AANTVIWDY | 207.6 | 0.0776 | 0.40234 | No | Class I |
| 6534 | 6542 | NTVIWDYKR | 98.07 | 0.8004 | 0.25478 | No | Class I |
| 6543 | 6551 | DAPAHISTI | 398.38 | 0.1673 | 0.07561 | No | Class I |
| 6544 | 6552 | APAHISTIG | 390.57 | 0.8594 | 0.12964 | No | Class I |
| 6548 | 6556 | ISTIGVCSM | 28.26 | 0.1967 | 0.07622 | No | Class I |
| 6554 | 6562 | CSMTDIAKK | 386.96 | 0.6207 | 0.03596 | No | Class I |
| 6559 | 6567 | IAKKPTETI | 410.34 | -0.0551 | -0.15408 | No | Class I |
| 6566 | 6574 | TICAPLTVF | 121.52 | -0.1451 | 0.05751 | No | Class I |
| 6567 | 6575 | ICAPLTVFF | 427.46 | -0.1507 | 0.13052 | No | Class I |
| 6578 | 6586 | RVDGQVDLF | 475.14 | -0.5439 | -0.0204 | No | Class I |
| 6581 | 6589 | GQVDLFRNA | 261.82 | -0.9701 | 0.17502 | No | Class I |
| 6582 | 6590 | QVDLFRNAR | 225.91 | -0.5669 | 0.17616 | No | Class I |
| 6585 | 6593 | LFRNARNGV | 272.75 | 0.3921 | 0.11145 | No | Class I |
| 6586 | 6594 | FRNARNGVL | 353.72 | 0.962 | 0.1343 | No | Class I |
| 6593 | 6601 | VLITEGSVK | 337.35 | 0.4667 | 0.09616 | No | Class I |
| 6599 | 6607 | SVKGLQPSV | 153.67 | 0.6803 | -0.26176 | No | Class I |
| 6602 | 6610 | GLQPSVGPK | 46.89 | 1.3482 | -0.14888 | No | Class I |
| 6611 | 6619 | QASLNGVTL | 124.31 | 0.5431 | 0.01826 | No | Class I |
| 6612 | 6620 | ASLNGVTLI | 494.78 | 0.4153 | 0.08803 | No | Class I |
| 6617 | 6625 | VTLIGEAVK | 444.13 | 0.5394 | 0.31471 | No | Class I |
| 6622 | 6630 | EAVKTQFNY | 265.17 | 0.7876 | -0.17982 | No | Class I |
| 6623 | 6631 | AVKTQFNYY | 358.73 | 0.9933 | -0.04116 | No | Class I |
| 6625 | 6633 | KTQFNYYKK | 93.13 | 0.4693 | -0.0587 | No | Class I |
| 6626 | 6634 | TQFNYYKKV | 183.24 | 0.4869 | -0.28359 | No | Class I |
| 6629 | 6637 | NYYKKVDGV | 466.15 | 0.0901 | -0.35082 | No | Class I |
| 6630 | 6638 | YYKKVDGVV | 235.4 | 0.1979 | -0.1732 | No | Class I |
| 6633 | 6641 | KVDGVVQQL | 457.79 | -0.2313 | -0.04508 | No | Class I |
| 6637 | 6645 | VVQQLPETY | 247.46 | -0.1094 | -0.06822 | No | Class I |
| 6638 | 6646 | VQQLPETYF | 248.46 | -0.1019 | 0.06529 | No | Class I |
| 6639 | 6647 | QQLPETYFT | 399.8 | 0.022 | 0.18456 | No | Class I |
| 6644 | 6652 | TYFTQSRNL | 421.97 | 0.3442 | -0.15157 | No | Class I |
| 6647 | 6655 | TQSRNLQEF | 67.22 | 0.3979 | -0.05762 | No | Class I |
| 6648 | 6656 | QSRNLQEFK | 264.31 | 0.4289 | 0.04335 | No | Class I |
| 6650 | 6658 | RNLQEFKPR | 123.32 | 0.5655 | -0.10094 | No | Class I |
| 6653 | 6661 | QEFKPRSQM | 256.03 | 1.1888 | -0.34838 | No | Class I |
| 6658 | 6666 | RSQMEIDFL | 299.76 | 1.3448 | 0.0956 | No | Class I |
| 6659 | 6667 | SQMEIDFLE | 265.23 | 1.1568 | 0.28655 | No | Class I |
| 6661 | 6669 | MEIDFLELA | 403.61 | 1.7498 | 0.2471 | No | Class I |
| 6666 | 6674 | LELAMDEFI | 301.41 | -0.1451 | 0.03855 | No | Class I |
| 6668 | 6676 | LAMDEFIER | 282.73 | -0.3797 | 0.34384 | No | Class I |
| 6669 | 6677 | AMDEFIERY | 79.14 | -0.7205 | 0.46197 | No | Class I |
| 6671 | 6679 | DEFIERYKL | 42.89 | -0.3244 | 0.18902 | No | Class I |
| 6676 | 6684 | RYKLEGYAF | 80.02 | 0.5587 | 0.06798 | No | Class I |
| 6679 | 6687 | LEGYAFEHI | 430.61 | 0.2302 | 0.25898 | No | Class I |
| 6681 | 6689 | GYAFEHIVY | 113.6 | 0.4935 | 0.39489 | No | Class I |
| 6682 | 6690 | YAFEHIVYG | 222.79 | 0.8955 | 0.32821 | No | Class I |
| 6684 | 6692 | FEHIVYGDF | 83.16 | 1.1633 | 0.2227 | No | Class I |
| 6689 | 6697 | YGDFSHSQL | 256.55 | 1.1036 | -0.21295 | No | Class I |
| 6692 | 6700 | FSHSQLGGL | 182.3 | 1.0806 | -0.23081 | No | Class I |
| 6694 | 6702 | HSQLGGLHL | 76.98 | 1.378 | 0.02568 | No | Class I |
| 6696 | 6704 | QLGGLHLLI | 195.37 | 0.5613 | 0.04891 | No | Class I |
| 6699 | 6707 | GLHLLIGLA | 70.7 | 1.0044 | 0.13594 | No | Class I |
| 6701 | 6709 | HLLIGLAKR | 134.5 | 1.6982 | 0.0599 | No | Class I |
| 6702 | 6710 | LLIGLAKRF | 182.7 | 0.9117 | -0.04843 | No | Class I |
| 6703 | 6711 | LIGLAKRFK | 359.92 | 1.1512 | -0.05298 | No | Class I |
| 6709 | 6717 | RFKESPFEL | 395.19 | 1.4061 | 0.01651 | No | Class I |
| 6713 | 6721 | SPFELEDFI | 466.85 | 0.9378 | 0.30932 | No | Class I |
| 6715 | 6723 | FELEDFIPM | 110.54 | 1.2669 | 0.33479 | No | Class I |
| 6739 | 6747 | SSKCVCSVI | 103.52 | 0.3985 | -0.2503 | No | Class I |
| 6742 | 6750 | CVCSVIDLL | 496.88 | 0.4323 | -0.00625 | No | Class I |
| 6749 | 6757 | LLLDDFVEI | 4.5 | -0.1957 | 0.24386 | No | Class I |
| 6750 | 6758 | LLDDFVEII | 38.08 | -0.5598 | 0.34464 | No | Class I |
| 6755 | 6763 | VEIIKSQDL | 266.32 | 0.8388 | -0.27341 | No | Class I |
| 6757 | 6765 | IIKSQDLSV | 230.3 | 1.2537 | -0.43441 | No | Class I |
| 6760 | 6768 | SQDLSVVSK | 449.87 | 1.1958 | -0.18802 | No | Class I |
| 6763 | 6771 | LSVVSKVVK | 467.63 | 0.6483 | -0.2502 | No | Class I |
| 6764 | 6772 | SVVSKVVKV | 359.77 | 0.3273 | -0.41537 | No | Class I |
| 6766 | 6774 | VSKVVKVTI | 454.24 | 0.3367 | -0.13374 | No | Class I |
| 6768 | 6776 | KVVKVTIDY | 264.25 | 0.3199 | -0.00158 | No | Class I |
| 6775 | 6783 | DYTEISFML | 330.44 | 1.7457 | 0.08342 | No | Class I |
| 6776 | 6784 | YTEISFMLW | 9.88 | 1.2159 | -0.03916 | No | Class I |
| 6778 | 6786 | EISFMLWCK | 474.79 | 1.3311 | 0.0381 | No | Class I |
| 6782 | 6790 | MLWCKDGHV | 27.64 | 0.251 | -0.12397 | No | Class I |
| 6793 | 6801 | FYPKLQSSQ | 395.38 | 0.5611 | -0.57672 | No | Class I |
| 6794 | 6802 | YPKLQSSQA | 169.25 | 0.1906 | -0.55699 | No | Class I |
| 6799 | 6807 | SSQAWQPGV | 440.29 | 0.2166 | 0.11887 | No | Class I |
| 6801 | 6809 | QAWQPGVAM | 220.94 | 0.2028 | 0.03414 | No | Class I |
| 6804 | 6812 | QPGVAMPNL | 260.68 | 0.405 | -0.0878 | No | Class I |
| 6806 | 6814 | GVAMPNLYK | 18.07 | -0.0047 | -0.19241 | No | Class I |
| 6807 | 6815 | VAMPNLYKM | 240.15 | -0.1728 | -0.21402 | No | Class I |
| 6809 | 6817 | MPNLYKMQR | 107.62 | -0.1851 | -0.43574 | No | Class I |
| 6811 | 6819 | NLYKMQRML | 239.4 | -0.5631 | -0.55716 | No | Class I |
| 6812 | 6820 | LYKMQRMLL | 72.69 | -0.3407 | -0.46546 | No | Class I |
| 6814 | 6822 | KMQRMLLEK | 10.86 | -1.0203 | -0.11782 | No | Class I |
| 6820 | 6828 | LEKCDLQNY | 270.3 | -0.0236 | -0.21463 | No | Class I |
| 6831 | 6839 | SATLPKGIM | 246.18 | -0.3773 | -0.106 | No | Class I |
| 6832 | 6840 | ATLPKGIMM | 27.17 | -0.2763 | -0.18314 | No | Class I |
| 6834 | 6842 | LPKGIMMNV | 282.48 | -0.2777 | -0.22358 | No | Class I |
| 6836 | 6844 | KGIMMNVAK | 330.18 | -0.1976 | -0.25289 | No | Class I |
| 6837 | 6845 | GIMMNVAKY | 137.94 | -0.2924 | -0.29412 | No | Class I |
| 6838 | 6846 | IMMNVAKYT | 338.61 | -0.1647 | -0.17064 | No | Class I |
| 6840 | 6848 | MNVAKYTQL | 204.3 | 0.2676 | -0.19563 | No | Class I |
| 6844 | 6852 | KYTQLCQYL | 47.72 | 0.9795 | -0.26543 | No | Class I |
| 6847 | 6855 | QLCQYLNTL | 261.96 | -0.1987 | -0.13088 | No | Class I |
| 6849 | 6857 | CQYLNTLTL | 228.94 | 0.299 | 0.0312 | No | Class I |
| 6851 | 6859 | YLNTLTLAV | 3.42 | 0.0716 | 0.0762 | No | Class I |
| 6853 | 6861 | NTLTLAVPY | 493.01 | -0.0367 | 0.08985 | No | Class I |
| 6855 | 6863 | LTLAVPYNM | 270.87 | 0.503 | 0.05863 | No | Class I |
| 6856 | 6864 | TLAVPYNMR | 155.35 | 0.9571 | -0.0681 | No | Class I |
| 6857 | 6865 | LAVPYNMRV | 113.49 | 0.8393 | -0.12541 | No | Class I |
| 6858 | 6866 | AVPYNMRVI | 329.57 | 0.9906 | -0.11112 | No | Class I |
| 6860 | 6868 | PYNMRVIHF | 473.24 | 1.6662 | 0.04168 | No | Class I |
| 6862 | 6870 | NMRVIHFGA | 461.79 | 1.922 | 0.33699 | No | Class I |
| 6875 | 6883 | GVAPGTAVL | 149.93 | 0.5767 | 0.12822 | No | Class I |
| 6876 | 6884 | VAPGTAVLR | 91.83 | 0.8933 | 0.13349 | No | Class I |
| 6884 | 6892 | RQWLPTGTL | 125.49 | 0.2484 | 0.13776 | No | Class I |
| 6885 | 6893 | QWLPTGTLL | 50.5 | 0.7327 | 0.08122 | No | Class I |
| 6901 | 6909 | FVSDADSTL | 210.03 | 0.0152 | -0.08934 | No | Class I |
| 6902 | 6910 | VSDADSTLI | 193.24 | 0.4543 | -0.06128 | No | Class I |
| 6908 | 6916 | TLIGDCATV | 11.38 | -0.2539 | 0.10385 | No | Class I |
| 6913 | 6921 | CATVHTANK | 337.53 | 0.6147 | 0.15142 | No | Class I |
| 6914 | 6922 | ATVHTANKW | 14.76 | 0.2276 | -0.01088 | No | Class I |
| 6918 | 6926 | TANKWDLII | 290.86 | 0.7673 | 0.08588 | No | Class I |
| 6925 | 6933 | IISDMYDPK | 116.35 | 0.2942 | -0.19362 | No | Class I |
| 6929 | 6937 | MYDPKTKNV | 322.08 | 1.1055 | -0.3632 | No | Class I |
| 6933 | 6941 | KTKNVTKEN | 140.61 | 0.7325 | -0.12327 | No | Class I |
| 6936 | 6944 | NVTKENDSK | 430.2 | -0.0446 | -0.19093 | No | Class I |
| 6939 | 6947 | KENDSKEGF | 408.65 | 0.3553 | -0.23958 | No | Class I |
| 6942 | 6950 | DSKEGFFTY | 231.54 | 1.03 | 0.29543 | No | Class I |
| 6944 | 6952 | KEGFFTYIC | 209.59 | 0.4284 | 0.35398 | No | Class I |
| 6946 | 6954 | GFFTYICGF | 247.66 | -0.8253 | 0.17304 | No | Class I |
| 6950 | 6958 | YICGFIQQK | 277.57 | 0.7704 | 0.09044 | No | Class I |
| 6953 | 6961 | GFIQQKLAL | 71.47 | 0.9943 | -0.37566 | No | Class I |
| 6958 | 6966 | KLALGGSVA | 80.1 | 0.7026 | -0.04906 | No | Class I |
| 6959 | 6967 | LALGGSVAI | 237.22 | 0.7429 | -0.03453 | No | Class I |
| 6960 | 6968 | ALGGSVAIK | 74.86 | 1.1524 | 0.03364 | No | Class I |
| 6966 | 6974 | AIKITEHSW | 406.08 | 1.2952 | 0.12661 | No | Class I |
| 6968 | 6976 | KITEHSWNA | 166.52 | 0.8345 | 0.17228 | No | Class I |
| 6971 | 6979 | EHSWNADLY | 401.69 | -0.0292 | 0.21196 | No | Class I |
| 6972 | 6980 | HSWNADLYK | 112.07 | -0.9997 | 0.11285 | No | Class I |
| 6973 | 6981 | SWNADLYKL | 414.52 | -0.8147 | -0.08069 | No | Class I |
| 6977 | 6985 | DLYKLMGHF | 260.22 | -0.7956 | -0.3468 | No | Class I |
| 6978 | 6986 | LYKLMGHFA | 241.38 | -0.5163 | -0.12456 | No | Class I |
| 6980 | 6988 | KLMGHFAWW | 317.87 | 0.3368 | 0.28124 | No | Class I |
| 6981 | 6989 | LMGHFAWWT | 163.22 | 0.5368 | 0.51074 | No | Class I |
| 6982 | 6990 | MGHFAWWTA | 457.04 | 0.8538 | 0.58453 | No | Class I |
| 6983 | 6991 | GHFAWWTAF | 273.41 | 0.6429 | 0.5572 | No | Class I |
| 6984 | 6992 | HFAWWTAFV | 468.66 | 0.6282 | 0.58925 | No | Class I |
| 6985 | 6993 | FAWWTAFVT | 158.6 | 0.2958 | 0.49234 | No | Class I |
| 6989 | 6997 | TAFVTNVNA | 257.34 | 0.1988 | 0.14231 | No | Class I |
| 6996 | 7004 | NASSSEAFL | 345 | -0.0868 | -0.1856 | No | Class I |
| 6997 | 7005 | ASSSEAFLI | 89.91 | 0.0392 | 0.00648 | No | Class I |
| 7001 | 7009 | EAFLIGCNY | 384.68 | 0.2576 | 0.13906 | No | Class I |
| 7002 | 7010 | AFLIGCNYL | 306.77 | 0.3417 | 0.10495 | No | Class I |
| 7003 | 7011 | FLIGCNYLG | 156.38 | 0.725 | 0.00911 | No | Class I |
| 7004 | 7012 | LIGCNYLGK | 436.59 | 0.7153 | -0.04259 | No | Class I |
| 7009 | 7017 | YLGKPREQI | 231.78 | 0.0178 | -0.15126 | No | Class I |
| 7014 | 7022 | REQIDGYVM | 23.14 | -0.3171 | 0.17082 | No | Class I |
| 7019 | 7027 | GYVMHANYI | 30.39 | 0.5628 | -0.10259 | No | Class I |
| 7020 | 7028 | YVMHANYIF | 120.68 | 0.6393 | 0.0822 | No | Class I |
| 7021 | 7029 | VMHANYIFW | 116.37 | 0.9171 | 0.22081 | No | Class I |
| 7022 | 7030 | MHANYIFWR | 74.27 | 0.5146 | 0.35609 | No | Class I |
| 7029 | 7037 | WRNTNPIQL | 465.1 | 1.3989 | 0.06486 | No | Class I |
| 7035 | 7043 | IQLSSYSLF | 441.2 | 0.7597 | -0.48075 | No | Class I |
| 7037 | 7045 | LSSYSLFDM | 36.01 | 0.7699 | -0.1172 | No | Class I |
| 7039 | 7047 | SYSLFDMSK | 455.21 | 0.6043 | -0.17484 | No | Class I |
| 7040 | 7048 | YSLFDMSKF | 350.07 | 0.175 | -0.29512 | No | Class I |
| 7042 | 7050 | LFDMSKFPL | 61.19 | -0.0311 | -0.44128 | No | Class I |
| 7045 | 7053 | MSKFPLKLR | 196.34 | 1.2433 | -0.16192 | No | Class I |
| 7048 | 7056 | FPLKLRGTA | 48.15 | 1.5912 | -0.1314 | No | Class I |
| 7049 | 7057 | PLKLRGTAV | 245.41 | 1.3234 | 0.05676 | No | Class I |
| 7051 | 7059 | KLRGTAVMS | 108.87 | 0.8928 | 0.05777 | No | Class I |
| 7052 | 7060 | LRGTAVMSL | 229.37 | 0.8822 | -0.11784 | No | Class I |
| 7053 | 7061 | RGTAVMSLK | 196.45 | 1.3773 | -0.21923 | No | Class I |
| 7061 | 7069 | KEGQINDMI | 403.49 | 0.5518 | -0.06593 | No | Class I |
| 7063 | 7071 | GQINDMILS | 358.05 | 0.4892 | -0.00117 | No | Class I |
| 7064 | 7072 | QINDMILSL | 347.66 | 0.797 | -0.13152 | No | Class I |
| 7067 | 7075 | DMILSLLSK | 484.01 | 0.6723 | -0.24552 | No | Class I |
| 7069 | 7077 | ILSLLSKGR | 346.53 | 1.0641 | -0.39359 | No | Class I |
| 7070 | 7078 | LSLLSKGRL | 97.65 | 1.223 | -0.32002 | No | Class I |
| 7071 | 7079 | SLLSKGRLI | 320.89 | 0.5798 | -0.31097 | No | Class I |
| 7072 | 7080 | LLSKGRLII | 262.1 | 0.4249 | -0.12058 | No | Class I |
| 7078 | 7086 | LIIRENNRV | 338.45 | 1.1346 | 0.21147 | No | Class I |
| 7079 | 7087 | IIRENNRVV | 427.86 | 0.6853 | 0.17296 | No | Class I |
| 7081 | 7089 | RENNRVVIS | 475.96 | 0.8121 | 0.19325 | No | Class I |
| 7085 | 7093 | RVVISSDVL | 302.39 | -0.3442 | -0.12667 | No | Class I |
| 7086 | 7094 | VVISSDVLV | 395.31 | -0.1481 | -0.23513 | No | Class I |
| 66 | 80 | QPYVFIKRSDARTAP | 16.29 | 0.4662 |  | Yes | Class II |
| 203 | 217 | DLLARAGKASCTLSE | 34.56 | 0.6379 |  | Yes | Class II |
| 246 | 260 | EKSYELQTPFEIKLA | 20.63 | 0.9236 |  | Yes | Class II |
| 253 | 267 | TPFEIKLAKKFDTFN | 45.01 | 0.7732 |  | Yes | Class II |
| 290 | 304 | KKKLDGFMGRIRSVY | 31.71 | 0.487 |  | Yes | Class II |
| 455 | 469 | QKEKVNINIVGDFKL | 32.31 | 0.8215 |  | Yes | Class II |
| 460 | 474 | NINIVGDFKLNEEIA | 38.25 | 0.7689 |  | Yes | Class II |
| 465 | 479 | GDFKLNEEIAIILAS | 22.35 | 0.8251 |  | Yes | Class II |
| 574 | 588 | SQYSLRLIDAMMFTS | 32.31 | 0.7276 |  | Yes | Class II |
| 594 | 608 | NLVVMAYITGGVVQL | 39.28 | 0.5545 |  | Yes | Class II |
| 598 | 612 | MAYITGGVVQLTSQW | 11.47 | 0.6904 |  | Yes | Class II |
| 802 | 816 | LAPNMMVTNNTFTLK | 23.04 | 0.6996 |  | Yes | Class II |
| 811 | 825 | NTFTLKGGAPTKVTF | 30.07 | 0.859 |  | Yes | Class II |
| 829 | 843 | TVIEVQGYKSVNITF | 15.59 | 1.2249 |  | Yes | Class II |
| 1021 | 1035 | QTIEVNSFSGYLKLT | 29.2 | 0.4111 |  | Yes | Class II |
| 1053 | 1067 | KPTVVVNAANVYLKH | 37.52 | 0.6614 |  | Yes | Class II |
| 1089 | 1103 | DYIATNGPLKVGGSC | 21.49 | 0.437 |  | Yes | Class II |
| 1124 | 1138 | KGEDIQLLKSAYENF | 36.47 | 0.445 |  | Yes | Class II |
| 1150 | 1164 | SAGIFGADPIHSLRV | 41.35 | 0.5839 |  | Yes | Class II |
| 1267 | 1281 | SATLVSDIDITFLKK | 26.74 | 1.2125 |  | Yes | Class II |
| 1350 | 1364 | KSAFYILPSIISNEK | 10.46 | 0.7169 |  | Yes | Class II |
| 1402 | 1416 | IQRKYKGIKIQEGVV | 30.58 | 0.6952 |  | Yes | Class II |
| 1550 | 1564 | VITFDNLKTLLSLRE | 18.01 | 0.5793 |  | Yes | Class II |
| 1632 | 1646 | FEYYHTTDPSFLGRY | 44.34 | 1.2694 |  | Yes | Class II |
| 1664 | 1678 | LTSIKWADNNCYLAT | 47.31 | 1.096 |  | Yes | Class II |
| 1685 | 1699 | QIELKFNPPALQDAY | 32.17 | 1.4234 |  | Yes | Class II |
| 1696 | 1710 | QDAYYRARAGEAANF | 45.99 | 0.6858 |  | Yes | Class II |
| 1707 | 1721 | AANFCALILAYCNKT | 37.03 | 0.4842 |  | Yes | Class II |
| 1712 | 1726 | ALILAYCNKTVGELG | 16.36 | 0.7269 |  | Yes | Class II |
| 1800 | 1814 | QESPFVMMSAPPAQY | 14.68 | 0.5853 |  | Yes | Class II |
| 2073 | 2087 | GDIILKPANNSLKIT | 48.42 | 0.8727 |  | Yes | Class II |
| 2212 | 2226 | EASFNYLKSPNFSKL | 26.03 | 0.5954 |  | Yes | Class II |
| 2239 | 2253 | CLGSLIYSTAALGVL | 15.31 | 0.5656 |  | Yes | Class II |
| 2311 | 2325 | ISSFKWDLTAFGLVA | 37.33 | 0.9895 |  | Yes | Class II |
| 2413 | 2427 | YKRNRATRVECTTIV | 48.55 | 0.4186 |  | Yes | Class II |
| 2465 | 2479 | ISDEVARDLSLQFKR | 31.39 | 0.7453 |  | Yes | Class II |
| 2518 | 2532 | LSHFVNLDNLRANNT | 12.51 | 0.6468 |  | Yes | Class II |
| 2698 | 2712 | HINAQVAKSHNIALI | 31.58 | 0.7673 |  | Yes | Class II |
| 2716 | 2730 | KDFMSLSEQLRKQIR | 46.91 | 0.4053 |  | Yes | Class II |
| 2803 | 2817 | SSEIIGYKAIDGGVT | 23.99 | 0.6869 |  | Yes | Class II |
| 2862 | 2876 | VGFVVPGLPGTILRT | 48.22 | 0.6637 |  | Yes | Class II |
| 2891 | 2905 | AVGNICYTPSKLIEY | 13.29 | 1.0892 |  | Yes | Class II |
| 2943 | 2957 | VAYESLRPDTRYVLM | 32.06 | 0.5807 |  | Yes | Class II |
| 3042 | 3056 | DISASIVAGGIVAIV | 14.38 | 0.7762 |  | Yes | Class II |
| 3047 | 3061 | IVAGGIVAIVVTCLA | 38.19 | 0.7363 |  | Yes | Class II |
| 3319 | 3333 | DLLIRKSNHNFLVQA | 15.04 | 0.6153 |  | Yes | Class II |
| 3611 | 3625 | FLPFAMGIIAMSAFA | 27.58 | 1.0725 |  | Yes | Class II |
| 3644 | 3658 | LATVAYFNMVYMPAS | 31.76 | 0.6554 |  | Yes | Class II |
| 3664 | 3678 | MTWLDMVDTSLSGFK | 49.27 | 0.8084 |  | Yes | Class II |
| 3733 | 3747 | MWALIISVTSNYSGV | 13.34 | 0.6264 |  | Yes | Class II |
| 3738 | 3752 | ISVTSNYSGVVTTVM | 41 | 0.7397 |  | Yes | Class II |
| 3739 | 3753 | SVTSNYSGVVTTVMF | 45.58 | 0.5599 |  | Yes | Class II |
| 3816 | 3830 | STQEFRYMNSQGLLP | 13.34 | 0.5127 |  | Yes | Class II |
| 3915 | 3929 | LSVLLSMQGAVDINK | 44.91 | 0.4912 |  | Yes | Class II |
| 3938 | 3952 | RATLQAIASEFSSLP | 47.19 | 0.515 |  | Yes | Class II |
| 3945 | 3959 | ASEFSSLPSYAAFAT | 7.78 | 0.4903 |  | Yes | Class II |
| 3950 | 3964 | SLPSYAAFATAQEAY | 23.54 | 0.421 |  | Yes | Class II |
| 4060 | 4074 | NIIPLTTAAKLMVVI | 41.79 | 0.7889 |  | Yes | Class II |
| 4062 | 4076 | IPLTTAAKLMVVIPD | 39.12 | 0.4302 |  | Yes | Class II |
| 4098 | 4112 | IQQVVDADSKIVQLS | 38.61 | 0.5952 |  | Yes | Class II |
| 4126 | 4140 | LIVTALRANSAVKLQ | 7.22 | 0.7473 |  | Yes | Class II |
| 4136 | 4150 | AVKLQNNELSPVALR | 26.01 | 1.5346 |  | Yes | Class II |
| 4225 | 4239 | VKYLYFIKGLNNLNR | 9.45 | 0.4595 |  | Yes | Class II |
| 4230 | 4244 | FIKGLNNLNRGMVLG | 28.03 | 0.6063 |  | Yes | Class II |
| 4239 | 4253 | RGMVLGSLAATVRLQ | 31.23 | 1.0211 |  | Yes | Class II |
| 4241 | 4255 | MVLGSLAATVRLQAG | 30.01 | 0.9441 |  | Yes | Class II |
| 4344 | 4358 | DLKGKYVQIPTTCAN | 28.22 | 0.8554 |  | Yes | Class II |
| 4511 | 4525 | LTKYTMADLVYALRH | 13 | 0.8184 |  | Yes | Class II |
| 4563 | 4577 | ILRVYANLGERVRQA | 11.13 | 0.5004 |  | Yes | Class II |
| 4627 | 4641 | DSYYSLLMPILTLTR | 8.9 | 0.5134 |  | Yes | Class II |
| 4660 | 4674 | WDLLKYDFTEERLKL | 32.7 | 0.4141 |  | Yes | Class II |
| 4705 | 4719 | FNVLFSTVFPPTSFG | 21.86 | 0.6119 |  | Yes | Class II |
| 4727 | 4741 | VDGVPFVVSTGYHFR | 12.06 | 0.9717 |  | Yes | Class II |
| 4738 | 4752 | YHFRELGVVHNQDVN | 13.52 | 1.49 |  | Yes | Class II |
| 4755 | 4769 | SSRLSFKELLVYAAD | 17.52 | 0.573 |  | Yes | Class II |
| 4770 | 4784 | PAMHAASGNLLLDKR | 49.42 | 0.5418 |  | Yes | Class II |
| 4783 | 4797 | KRTTCFSVAALTNNV | 37.97 | 1.0636 |  | Yes | Class II |
| 4788 | 4802 | FSVAALTNNVAFQTV | 19.49 | 0.7028 |  | Yes | Class II |
| 4829 | 4843 | LKHFFFAQDGNAAIS | 18.63 | 0.4787 |  | Yes | Class II |
| 4832 | 4846 | FFFAQDGNAAISDYD | 41.11 | 0.6016 |  | Yes | Class II |
| 4938 | 4952 | YAISAKNRARTVAGV | 34.54 | 1.2154 |  | Yes | Class II |
| 4971 | 4985 | IAATRGATVVIGTSK | 24.51 | 0.7805 |  | Yes | Class II |
| 5019 | 5033 | PNMLRIMASLVLARK | 23.8 | 0.4128 |  | Yes | Class II |
| 5160 | 5174 | STYASQGLVASIKNF | 38.34 | 0.5366 |  | Yes | Class II |
| 5268 | 5282 | EYADVFHLYLQYIRK | 48.72 | 0.439 |  | Yes | Class II |
| 5465 | 5479 | TEETFKLSYGIATVR | 18.34 | 0.8859 |  | Yes | Class II |
| 5470 | 5484 | KLSYGIATVREVLSD | 45.18 | 0.6721 |  | Yes | Class II |
| 5477 | 5491 | TVREVLSDRELHLSW | 47.77 | 1.3343 |  | Yes | Class II |
| 5500 | 5514 | LNRNYVFTGYRVTKN | 21.03 | 0.6492 |  | Yes | Class II |
| 5505 | 5519 | VFTGYRVTKNSKVQI | 23.71 | 0.6256 |  | Yes | Class II |
| 5538 | 5552 | TTTYKLNVGDYFVLT | 41.95 | 0.5567 |  | Yes | Class II |
| 5611 | 5625 | GKSHFAIGLALYYPS | 44.23 | 0.8414 |  | Yes | Class II |
| 5616 | 5630 | AIGLALYYPSARIVY | 14.09 | 0.6192 |  | Yes | Class II |
| 5678 | 5692 | QYVFCTVNALPETTA | 33.42 | 0.5566 |  | Yes | Class II |
| 5706 | 5720 | YDLSVVNARLRAKHY | 16.94 | 1.8581 |  | Yes | Class II |
| 5833 | 5847 | AVFISPYNSQNAVAS | 24.04 | 0.505 |  | Yes | Class II |
| 5838 | 5852 | PYNSQNAVASKILGL | 31.46 | 0.4765 |  | Yes | Class II |
| 5949 | 5963 | PTHLSVDTKFKTEGL | 38.94 | 1.07 |  | Yes | Class II |
| 5973 | 5987 | DMTYRRLISMMGFKM | 13.13 | 0.6502 |  | Yes | Class II |
| 6031 | 6045 | PLQLGFSTGVNLVAV | 27.07 | 1.2929 |  | Yes | Class II |
| 6120 | 6134 | MKYFVKIGPERTCCL | 13.36 | 0.6989 |  | Yes | Class II |
| 6234 | 6248 | CRKVQHMVVKAALLA | 34.43 | 0.4926 |  | Yes | Class II |
| 6358 | 6372 | KSAFVNLKQLPFFYY | 30.25 | 1.1654 |  | Yes | Class II |
| 6388 | 6402 | IDYVPLKSATCITRC | 24.47 | 0.8877 |  | Yes | Class II |
| 6445 | 6459 | WNTFTRLQSLENVAF | 6.3 | 0.4028 |  | Yes | Class II |
| 6519 | 6533 | PEVKILNNLGVDIAA | 8.25 | 0.4019 |  | Yes | Class II |
| 6760 | 6774 | SQDLSVVSKVVKVTI | 40.23 | 0.7673 |  | Yes | Class II |
| 6850 | 6864 | QYLNTLTLAVPYNMR | 25.83 | 0.4384 |  | Yes | Class II |
| 6956 | 6970 | QQKLALGGSVAIKIT | 20.01 | 1.2533 |  | Yes | Class II |
| 6957 | 6971 | QKLALGGSVAIKITE | 35.2 | 1.195 |  | Yes | Class II |
| 7075 | 7089 | KGRLIIRENNRVVIS | 7.52 | 0.7821 |  | Yes | Class II |
| 1 | 15 | MESLVPGFNEKTHVQ | 602.81 | 0.747 |  | No | Class II |
| 7 | 21 | GFNEKTHVQLSLPVL | 103.96 | 0.8143 |  | No | Class II |
| 10 | 24 | EKTHVQLSLPVLQVR | 446.69 | 1.155 |  | No | Class II |
| 12 | 26 | THVQLSLPVLQVRDV | 332 | 1.3747 |  | No | Class II |
| 17 | 31 | SLPVLQVRDVLVRGF | 616.76 | 0.2552 |  | No | Class II |
| 24 | 38 | RDVLVRGFGDSVEEV | 765 | -0.8488 |  | No | Class II |
| 25 | 39 | DVLVRGFGDSVEEVL | 163.67 | -0.7247 |  | No | Class II |
| 30 | 44 | GFGDSVEEVLSEARQ | 724.97 | 0.2174 |  | No | Class II |
| 33 | 47 | DSVEEVLSEARQHLK | 332.41 | 0.2668 |  | No | Class II |
| 43 | 57 | RQHLKDGTCGLVEVE | 559.91 | 1.2745 |  | No | Class II |
| 50 | 64 | TCGLVEVEKGVLPQL | 447.42 | 0.4822 |  | No | Class II |
| 54 | 68 | VEVEKGVLPQLEQPY | 614.82 | 0.1658 |  | No | Class II |
| 58 | 72 | KGVLPQLEQPYVFIK | 332.32 | 0.0865 |  | No | Class II |
| 68 | 82 | YVFIKRSDARTAPHG | 141.85 | 0.2914 |  | No | Class II |
| 76 | 90 | ARTAPHGHVMVELVA | 607.06 | 0.4329 |  | No | Class II |
| 81 | 95 | HGHVMVELVAELEGI | 331.02 | 0.1733 |  | No | Class II |
| 86 | 100 | VELVAELEGIQYGRS | 188.99 | 0.7533 |  | No | Class II |
| 92 | 106 | LEGIQYGRSGETLGV | 82.98 | 0.9268 |  | No | Class II |
| 100 | 114 | SGETLGVLVPHVGEI | 559.98 | 0.2348 |  | No | Class II |
| 106 | 120 | VLVPHVGEIPVAYRK | 72.34 | 0.6187 |  | No | Class II |
| 111 | 125 | VGEIPVAYRKVLLRK | 29.33 | -0.0353 |  | No | Class II |
| 116 | 130 | VAYRKVLLRKNGNKG | 72.44 | -0.1789 |  | No | Class II |
| 121 | 135 | VLLRKNGNKGAGGHS | 496.6 | 0.2631 |  | No | Class II |
| 127 | 141 | GNKGAGGHSYGADLK | 155.88 | 0.5152 |  | No | Class II |
| 132 | 146 | GGHSYGADLKSFDLG | 662.31 | 0.9321 |  | No | Class II |
| 153 | 167 | PYEDFQENWNTKHSS | 768.11 | 0.4231 |  | No | Class II |
| 161 | 175 | WNTKHSSGVTRELMR | 377.43 | 0.1181 |  | No | Class II |
| 170 | 184 | TRELMRELNGGAYTR | 117.76 | 0.1081 |  | No | Class II |
| 175 | 189 | RELNGGAYTRYVDNN | 151.82 | 0.5573 |  | No | Class II |
| 178 | 192 | NGGAYTRYVDNNFCG | 761.47 | 0.2733 |  | No | Class II |
| 194 | 208 | DGYPLECIKDLLARA | 59.1 | -0.252 |  | No | Class II |
| 199 | 213 | ECIKDLLARAGKASC | 400.03 | -0.0505 |  | No | Class II |
| 204 | 218 | LLARAGKASCTLSEQ | 897.36 | 0.8483 |  | No | Class II |
| 209 | 223 | GKASCTLSEQLDFID | 616.93 | 1.2716 |  | No | Class II |
| 215 | 229 | LSEQLDFIDTKRGVY | 181.68 | 1.5548 |  | No | Class II |
| 232 | 246 | REHEHEIAWYTERSE | 928.29 | 0.7199 |  | No | Class II |
| 235 | 249 | EHEIAWYTERSEKSY | 916.41 | 0.61 |  | No | Class II |
| 237 | 251 | EIAWYTERSEKSYEL | 97.21 | 0.9369 |  | No | Class II |
| 245 | 259 | SEKSYELQTPFEIKL | 799.86 | 0.9073 |  | No | Class II |
| 250 | 264 | ELQTPFEIKLAKKFD | 821.06 | 0.7673 |  | No | Class II |
| 258 | 272 | KLAKKFDTFNGECPN | 967.99 | 0.2536 |  | No | Class II |
| 263 | 277 | FDTFNGECPNFVFPL | 622.43 | 0.5109 |  | No | Class II |
| 269 | 283 | ECPNFVFPLNSIIKT | 134.54 | 0.2621 |  | No | Class II |
| 274 | 288 | VFPLNSIIKTIQPRV | 86.09 | -0.0018 |  | No | Class II |
| 275 | 289 | FPLNSIIKTIQPRVE | 830.31 | 0.0809 |  | No | Class II |
| 279 | 293 | SIIKTIQPRVEKKKL | 551.41 | -0.1507 |  | No | Class II |
| 292 | 306 | KLDGFMGRIRSVYPV | 231.9 | 0.5718 |  | No | Class II |
| 296 | 310 | FMGRIRSVYPVASPN | 90.17 | 0.5529 |  | No | Class II |
| 297 | 311 | MGRIRSVYPVASPNE | 170 | 0.47 |  | No | Class II |
| 308 | 322 | SPNECNQMCLSTLMK | 462.85 | 0.2962 |  | No | Class II |
| 311 | 325 | ECNQMCLSTLMKCDH | 570.33 | 0.3285 |  | No | Class II |
| 313 | 327 | NQMCLSTLMKCDHCG | 604.95 | 0.072 |  | No | Class II |
| 326 | 340 | CGETSWQTGDFVKAT | 900.17 | 0.8856 |  | No | Class II |
| 332 | 346 | QTGDFVKATCEFCGT | 727.53 | 0.512 |  | No | Class II |
| 348 | 362 | NLTKEGATTCGYLPQ | 756.7 | 0.3031 |  | No | Class II |
| 355 | 369 | TTCGYLPQNAVVKIY | 84.83 | 0.0368 |  | No | Class II |
| 358 | 372 | GYLPQNAVVKIYCPA | 306.93 | 0.0222 |  | No | Class II |
| 362 | 376 | QNAVVKIYCPACHNS | 92.25 | -0.0701 |  | No | Class II |
| 379 | 393 | GPEHSLAEYHNESGL | 632.58 | 0.2734 |  | No | Class II |
| 384 | 398 | LAEYHNESGLKTILR | 223 | 0.013 |  | No | Class II |
| 391 | 405 | SGLKTILRKGGRTIA | 48.11 | -0.2024 |  | No | Class II |
| 394 | 408 | KTILRKGGRTIAFGG | 445.76 | 0.2346 |  | No | Class II |
| 400 | 414 | GGRTIAFGGCVFSYV | 292.93 | 0.0718 |  | No | Class II |
| 401 | 415 | GRTIAFGGCVFSYVG | 98.79 | 0.2335 |  | No | Class II |
| 405 | 419 | AFGGCVFSYVGCHNK | 178.9 | 0.0124 |  | No | Class II |
| 410 | 424 | VFSYVGCHNKCAYWV | 456.39 | 0.6289 |  | No | Class II |
| 416 | 430 | CHNKCAYWVPRASAN | 674.26 | 0.5643 |  | No | Class II |
| 417 | 431 | HNKCAYWVPRASANI | 302.74 | 0.7308 |  | No | Class II |
| 421 | 435 | AYWVPRASANIGCNH | 359.75 | 0.8594 |  | No | Class II |
| 426 | 440 | RASANIGCNHTGVVG | 740.45 | 1.1915 |  | No | Class II |
| 429 | 443 | ANIGCNHTGVVGEGS | 487.64 | 1.1367 |  | No | Class II |
| 434 | 448 | NHTGVVGEGSEGLND | 898.81 | 0.6973 |  | No | Class II |
| 440 | 454 | GEGSEGLNDNLLEIL | 643.43 | 0.2022 |  | No | Class II |
| 442 | 456 | GSEGLNDNLLEILQK | 656.47 | 0.2807 |  | No | Class II |
| 449 | 463 | NLLEILQKEKVNINI | 357.07 | 0.7904 |  | No | Class II |
| 459 | 473 | VNINIVGDFKLNEEI | 402.75 | 0.7417 |  | No | Class II |
| 468 | 482 | KLNEEIAIILASFSA | 57.72 | 0.5048 |  | No | Class II |
| 472 | 486 | EIAIILASFSASTSA | 367.41 | 0.5487 |  | No | Class II |
| 477 | 491 | LASFSASTSAFVETV | 43.81 | 0.1931 |  | No | Class II |
| 482 | 496 | ASTSAFVETVKGLDY | 524.97 | 0.6912 |  | No | Class II |
| 484 | 498 | TSAFVETVKGLDYKA | 180.4 | 0.7925 |  | No | Class II |
| 491 | 505 | VKGLDYKAFKQIVES | 441.48 | 0.1548 |  | No | Class II |
| 499 | 513 | FKQIVESCGNFKVTK | 128.17 | -0.2099 |  | No | Class II |
| 504 | 518 | ESCGNFKVTKGKAKK | 193.61 | -0.0056 |  | No | Class II |
| 507 | 521 | GNFKVTKGKAKKGAW | 634.93 | 0.489 |  | No | Class II |
| 509 | 523 | FKVTKGKAKKGAWNI | 725.88 | 0.5349 |  | No | Class II |
| 520 | 534 | AWNIGEQKSILSPLY | 144.09 | 0.4974 |  | No | Class II |
| 525 | 539 | EQKSILSPLYAFASE | 430.79 | 0.1775 |  | No | Class II |
| 530 | 544 | LSPLYAFASEAARVV | 39.94 | 0.3102 |  | No | Class II |
| 535 | 549 | AFASEAARVVRSIFS | 14.09 | -0.0212 |  | No | Class II |
| 541 | 555 | ARVVRSIFSRTLETA | 13.39 | -0.4052 |  | No | Class II |
| 542 | 556 | RVVRSIFSRTLETAQ | 531.54 | -0.475 |  | No | Class II |
| 549 | 563 | SRTLETAQNSVRVLQ | 910.86 | 0.199 |  | No | Class II |
| 553 | 567 | ETAQNSVRVLQKAAI | 451.86 | 0.2593 |  | No | Class II |
| 556 | 570 | QNSVRVLQKAAITIL | 91.37 | 0.3765 |  | No | Class II |
| 560 | 574 | RVLQKAAITILDGIS | 241.59 | 0.0934 |  | No | Class II |
| 565 | 579 | AAITILDGISQYSLR | 187.75 | 0.5179 |  | No | Class II |
| 567 | 581 | ITILDGISQYSLRLI | 210.84 | 0.4384 |  | No | Class II |
| 572 | 586 | GISQYSLRLIDAMMF | 365.47 | 0.7195 |  | No | Class II |
| 578 | 592 | LRLIDAMMFTSDLAT | 545.63 | 0.0931 |  | No | Class II |
| 583 | 597 | AMMFTSDLATNNLVV | 49.61 | -0.0532 |  | No | Class II |
| 586 | 600 | FTSDLATNNLVVMAY | 411.66 | 0.0086 |  | No | Class II |
| 588 | 602 | SDLATNNLVVMAYIT | 43.97 | 0.3136 |  | No | Class II |
| 591 | 605 | ATNNLVVMAYITGGV | 193.25 | 0.3708 |  | No | Class II |
| 602 | 616 | TGGVVQLTSQWLTNI | 209.35 | 0.4277 |  | No | Class II |
| 606 | 620 | VQLTSQWLTNIFGTV | 921.48 | 0.0748 |  | No | Class II |
| 610 | 624 | SQWLTNIFGTVYEKL | 221.51 | -0.3542 |  | No | Class II |
| 611 | 625 | QWLTNIFGTVYEKLK | 830.85 | -0.2045 |  | No | Class II |
| 617 | 631 | FGTVYEKLKPVLDWL | 64.13 | 0.5998 |  | No | Class II |
| 621 | 635 | YEKLKPVLDWLEEKF | 310.12 | 0.3832 |  | No | Class II |
| 622 | 636 | EKLKPVLDWLEEKFK | 273.42 | 0.4723 |  | No | Class II |
| 637 | 651 | EGVEFLRDGWEIVKF | 634.28 | 0.3916 |  | No | Class II |
| 638 | 652 | GVEFLRDGWEIVKFI | 513.85 | 0.2769 |  | No | Class II |
| 642 | 656 | LRDGWEIVKFISTCA | 64.3 | 0.2993 |  | No | Class II |
| 646 | 660 | WEIVKFISTCACEIV | 685.83 | -0.1652 |  | No | Class II |
| 647 | 661 | EIVKFISTCACEIVG | 15.29 | -0.1946 |  | No | Class II |
| 651 | 665 | FISTCACEIVGGQIV | 778.25 | -0.0859 |  | No | Class II |
| 652 | 666 | ISTCACEIVGGQIVT | 567.09 | -0.2366 |  | No | Class II |
| 656 | 670 | ACEIVGGQIVTCAKE | 65.35 | 0.2362 |  | No | Class II |
| 661 | 675 | GGQIVTCAKEIKESV | 844.27 | 0.2034 |  | No | Class II |
| 668 | 682 | AKEIKESVQTFFKLV | 350.92 | -0.019 |  | No | Class II |
| 674 | 688 | SVQTFFKLVNKFLAL | 59.01 | 0.093 |  | No | Class II |
| 681 | 695 | LVNKFLALCADSIII | 185.37 | -0.0911 |  | No | Class II |
| 682 | 696 | VNKFLALCADSIIIG | 10.57 | -0.0167 |  | No | Class II |
| 686 | 700 | LALCADSIIIGGAKL | 272.91 | 0.1079 |  | No | Class II |
| 687 | 701 | ALCADSIIIGGAKLK | 827.27 | 0.576 |  | No | Class II |
| 691 | 705 | DSIIIGGAKLKALNL | 101.91 | 1.1502 |  | No | Class II |
| 692 | 706 | SIIIGGAKLKALNLG | 474.64 | 1.6995 |  | No | Class II |
| 698 | 712 | AKLKALNLGETFVTH | 247.6 | 1.0714 |  | No | Class II |
| 700 | 714 | LKALNLGETFVTHSK | 150.41 | 1.1964 |  | No | Class II |
| 707 | 721 | ETFVTHSKGLYRKCV | 27.73 | -0.3409 |  | No | Class II |
| 713 | 727 | SKGLYRKCVKSREET | 194.06 | -0.1202 |  | No | Class II |
| 722 | 736 | KSREETGLLMPLKAP | 121.16 | 0.4302 |  | No | Class II |
| 723 | 737 | SREETGLLMPLKAPK | 777.78 | 0.3326 |  | No | Class II |
| 727 | 741 | TGLLMPLKAPKEIIF | 143.71 | 0.2079 |  | No | Class II |
| 733 | 747 | LKAPKEIIFLEGETL | 545 | -0.0783 |  | No | Class II |
| 735 | 749 | APKEIIFLEGETLPT | 220.49 | 0.192 |  | No | Class II |
| 738 | 752 | EIIFLEGETLPTEVL | 188.29 | 0.0894 |  | No | Class II |
| 740 | 754 | IFLEGETLPTEVLTE | 478.84 | -0.246 |  | No | Class II |
| 743 | 757 | EGETLPTEVLTEEVV | 341.89 | -0.2176 |  | No | Class II |
| 747 | 761 | LPTEVLTEEVVLKTG | 975.9 | -0.0974 |  | No | Class II |
| 753 | 767 | TEEVVLKTGDLQPLE | 752.29 | 0.9877 |  | No | Class II |
| 755 | 769 | EVVLKTGDLQPLEQP | 960.3 | 0.8528 |  | No | Class II |
| 765 | 779 | PLEQPTSEAVEAPLV | 790.59 | -0.085 |  | No | Class II |
| 774 | 788 | VEAPLVGTPVCINGL | 224.97 | -0.0372 |  | No | Class II |
| 775 | 789 | EAPLVGTPVCINGLM | 631.73 | -0.0017 |  | No | Class II |
| 780 | 794 | GTPVCINGLMLLEIK | 61.27 | 0.8013 |  | No | Class II |
| 781 | 795 | TPVCINGLMLLEIKD | 406.05 | 1.0514 |  | No | Class II |
| 785 | 799 | INGLMLLEIKDTEKY | 562.68 | 1.0353 |  | No | Class II |
| 790 | 804 | LLEIKDTEKYCALAP | 979.1 | 1.3561 |  | No | Class II |
| 796 | 810 | TEKYCALAPNMMVTN | 716.88 | 0.6824 |  | No | Class II |
| 806 | 820 | MMVTNNTFTLKGGAP | 535.43 | 0.6488 |  | No | Class II |
| 822 | 836 | KVTFGDDTVIEVQGY | 695.03 | 0.4815 |  | No | Class II |
| 830 | 844 | VIEVQGYKSVNITFE | 883 | 1.2776 |  | No | Class II |
| 834 | 848 | QGYKSVNITFELDER | 94.76 | 1.5539 |  | No | Class II |
| 836 | 850 | YKSVNITFELDERID | 174.67 | 1.4773 |  | No | Class II |
| 839 | 853 | VNITFELDERIDKVL | 63.8 | 0.9044 |  | No | Class II |
| 841 | 855 | ITFELDERIDKVLNE | 955.16 | 0.3727 |  | No | Class II |
| 848 | 862 | RIDKVLNEKCSAYTV | 744.78 | 0.1169 |  | No | Class II |
| 858 | 872 | SAYTVELGTEVNEFA | 387.66 | 0.4624 |  | No | Class II |
| 865 | 879 | GTEVNEFACVVADAV | 153.94 | 0.4957 |  | No | Class II |
| 866 | 880 | TEVNEFACVVADAVI | 80.4 | 0.3459 |  | No | Class II |
| 870 | 884 | EFACVVADAVIKTLQ | 551.65 | 0.6432 |  | No | Class II |
| 871 | 885 | FACVVADAVIKTLQP | 89.41 | 0.4683 |  | No | Class II |
| 877 | 891 | DAVIKTLQPVSELLT | 862.36 | 0.0374 |  | No | Class II |
| 883 | 897 | LQPVSELLTPLGIDL | 144.85 | 0.6885 |  | No | Class II |
| 887 | 901 | SELLTPLGIDLDEWS | 336.7 | 0.1236 |  | No | Class II |
| 892 | 906 | PLGIDLDEWSMATYY | 341.18 | 0.4155 |  | No | Class II |
| 897 | 911 | LDEWSMATYYLFDES | 237.21 | -0.0714 |  | No | Class II |
| 902 | 916 | MATYYLFDESGEFKL | 403.03 | 0.1577 |  | No | Class II |
| 903 | 917 | ATYYLFDESGEFKLA | 194.88 | 0.231 |  | No | Class II |
| 911 | 925 | SGEFKLASHMYCSFY | 111.11 | 0.5067 |  | No | Class II |
| 912 | 926 | GEFKLASHMYCSFYP | 275.13 | 0.6989 |  | No | Class II |
| 916 | 930 | LASHMYCSFYPPDED | 556.77 | 0.2901 |  | No | Class II |
| 955 | 969 | YQGKPLEFGATSAAL | 96.08 | 1.7117 |  | No | Class II |
| 957 | 971 | GKPLEFGATSAALQP | 118.95 | 1.6005 |  | No | Class II |
| 960 | 974 | LEFGATSAALQPEEE | 127.69 | 1.4306 |  | No | Class II |
| 965 | 979 | TSAALQPEEEQEEDW | 786.52 | 1.0093 |  | No | Class II |
| 994 | 1008 | SEDNQTTTIQTIVEV | 156.96 | 0.2371 |  | No | Class II |
| 997 | 1011 | NQTTTIQTIVEVQPQ | 944.18 | 0.755 |  | No | Class II |
| 1000 | 1014 | TTIQTIVEVQPQLEM | 417.93 | 0.8106 |  | No | Class II |
| 1002 | 1016 | IQTIVEVQPQLEMEL | 523.54 | 0.9118 |  | No | Class II |
| 1005 | 1019 | IVEVQPQLEMELTPV | 777.69 | 1.716 |  | No | Class II |
| 1009 | 1023 | QPQLEMELTPVVQTI | 223.88 | 1.1467 |  | No | Class II |
| 1014 | 1028 | MELTPVVQTIEVNSF | 381.96 | 0.8902 |  | No | Class II |
| 1016 | 1030 | LTPVVQTIEVNSFSG | 919.33 | 0.4347 |  | No | Class II |
| 1019 | 1033 | VVQTIEVNSFSGYLK | 910.29 | 0.1093 |  | No | Class II |
| 1029 | 1043 | SGYLKLTDNVYIKNA | 79 | 0.7354 |  | No | Class II |
| 1036 | 1050 | DNVYIKNADIVEEAK | 537.93 | 0.952 |  | No | Class II |
| 1037 | 1051 | NVYIKNADIVEEAKK | 741.3 | 0.9128 |  | No | Class II |
| 1043 | 1057 | ADIVEEAKKVKPTVV | 635.07 | 0.8012 |  | No | Class II |
| 1048 | 1062 | EAKKVKPTVVVNAAN | 295.89 | 0.7655 |  | No | Class II |
| 1049 | 1063 | AKKVKPTVVVNAANV | 177.4 | 0.858 |  | No | Class II |
| 1054 | 1068 | PTVVVNAANVYLKHG | 248.01 | 0.745 |  | No | Class II |
| 1061 | 1075 | ANVYLKHGGGVAGAL | 277.08 | 0.3873 |  | No | Class II |
| 1063 | 1077 | VYLKHGGGVAGALNK | 21.25 | 0.3974 |  | No | Class II |
| 1068 | 1082 | GGGVAGALNKATNNA | 107.94 | 0.2774 |  | No | Class II |
| 1071 | 1085 | VAGALNKATNNAMQV | 73.8 | 0.3341 |  | No | Class II |
| 1077 | 1091 | KATNNAMQVESDDYI | 369.27 | 0.4911 |  | No | Class II |
| 1086 | 1100 | ESDDYIATNGPLKVG | 746.79 | 0.4494 |  | No | Class II |
| 1091 | 1105 | IATNGPLKVGGSCVL | 523.31 | 0.3055 |  | No | Class II |
| 1094 | 1108 | NGPLKVGGSCVLSGH | 180.38 | 0.5429 |  | No | Class II |
| 1096 | 1110 | PLKVGGSCVLSGHNL | 91.45 | 0.9802 |  | No | Class II |
| 1099 | 1113 | VGGSCVLSGHNLAKH | 907.93 | 0.334 |  | No | Class II |
| 1104 | 1118 | VLSGHNLAKHCLHVV | 228.72 | 0.3873 |  | No | Class II |
| 1110 | 1124 | LAKHCLHVVGPNVNK | 110.15 | -0.0262 |  | No | Class II |
| 1121 | 1135 | NVNKGEDIQLLKSAY | 603.66 | 0.5255 |  | No | Class II |
| 1132 | 1146 | KSAYENFNQHEVLLA | 238.71 | 0.2809 |  | No | Class II |
| 1137 | 1151 | NFNQHEVLLAPLLSA | 37.18 | 0.3907 |  | No | Class II |
| 1138 | 1152 | FNQHEVLLAPLLSAG | 245.02 | 0.3626 |  | No | Class II |
| 1142 | 1156 | EVLLAPLLSAGIFGA | 104.96 | 0.3021 |  | No | Class II |
| 1145 | 1159 | LAPLLSAGIFGADPI | 58.36 | 0.523 |  | No | Class II |
| 1155 | 1169 | GADPIHSLRVCVDTV | 815.78 | -0.1672 |  | No | Class II |
| 1156 | 1170 | ADPIHSLRVCVDTVR | 134.22 | -0.0502 |  | No | Class II |
| 1164 | 1178 | VCVDTVRTNVYLAVF | 543.74 | 0.2026 |  | No | Class II |
| 1169 | 1183 | VRTNVYLAVFDKNLY | 661.4 | 0.0577 |  | No | Class II |
| 1172 | 1186 | NVYLAVFDKNLYDKL | 221.37 | -0.1651 |  | No | Class II |
| 1180 | 1194 | KNLYDKLVSSFLEMK | 35.81 | 0.1732 |  | No | Class II |
| 1182 | 1196 | LYDKLVSSFLEMKSE | 937.47 | 0.4523 |  | No | Class II |
| 1185 | 1199 | KLVSSFLEMKSEKQV | 54.16 | 0.4492 |  | No | Class II |
| 1190 | 1204 | FLEMKSEKQVEQKIA | 201.51 | 0.5515 |  | No | Class II |
| 1200 | 1214 | EQKIAEIPKEEVKPF | 602.38 | 0.5428 |  | No | Class II |
| 1206 | 1220 | IPKEEVKPFITESKP | 324.29 | 0.3398 |  | No | Class II |
| 1211 | 1225 | VKPFITESKPSVEQR | 261.25 | 0.4789 |  | No | Class II |
| 1212 | 1226 | KPFITESKPSVEQRK | 730.47 | 0.5716 |  | No | Class II |
| 1228 | 1242 | DDKKIKACVEEVTTT | 984.07 | 0.22 |  | No | Class II |
| 1234 | 1248 | ACVEEVTTTLEETKF | 999.5 | 0.6167 |  | No | Class II |
| 1240 | 1254 | TTTLEETKFLTENLL | 786.94 | 0.0557 |  | No | Class II |
| 1242 | 1256 | TLEETKFLTENLLLY | 435.11 | -0.0496 |  | No | Class II |
| 1245 | 1259 | ETKFLTENLLLYIDI | 220.69 | 0.0685 |  | No | Class II |
| 1248 | 1262 | FLTENLLLYIDINGN | 173.15 | -0.1897 |  | No | Class II |
| 1252 | 1266 | NLLLYIDINGNLHPD | 98.97 | 0.1931 |  | No | Class II |
| 1253 | 1267 | LLLYIDINGNLHPDS | 857.86 | 0.1146 |  | No | Class II |
| 1260 | 1274 | NGNLHPDSATLVSDI | 334.08 | 0.2932 |  | No | Class II |
| 1261 | 1275 | GNLHPDSATLVSDID | 942.58 | 0.483 |  | No | Class II |
| 1266 | 1280 | DSATLVSDIDITFLK | 207.97 | 0.9044 |  | No | Class II |
| 1276 | 1290 | ITFLKKDAPYIVGDV | 84.83 | 0.6742 |  | No | Class II |
| 1282 | 1296 | DAPYIVGDVVQEGVL | 358.2 | 0.1525 |  | No | Class II |
| 1283 | 1297 | APYIVGDVVQEGVLT | 120.26 | 0.2457 |  | No | Class II |
| 1287 | 1301 | VGDVVQEGVLTAVVI | 144.05 | 0.4104 |  | No | Class II |
| 1292 | 1306 | QEGVLTAVVIPTKKA | 73.23 | 0.789 |  | No | Class II |
| 1297 | 1311 | TAVVIPTKKAGGTTE | 350.61 | 0.7214 |  | No | Class II |
| 1303 | 1317 | TKKAGGTTEMLAKAL | 401.58 | 0.0576 |  | No | Class II |
| 1304 | 1318 | KKAGGTTEMLAKALR | 747.96 | 0.1266 |  | No | Class II |
| 1309 | 1323 | TTEMLAKALRKVPTD | 115.72 | -0.1 |  | No | Class II |
| 1314 | 1328 | AKALRKVPTDNYITT | 705.06 | -0.2258 |  | No | Class II |
| 1324 | 1338 | NYITTYPGQGLNGYT | 30.17 | -0.1776 |  | No | Class II |
| 1325 | 1339 | YITTYPGQGLNGYTV | 579.03 | -0.0019 |  | No | Class II |
| 1329 | 1343 | YPGQGLNGYTVEEAK | 686.26 | 0.2686 |  | No | Class II |
| 1331 | 1345 | GQGLNGYTVEEAKTV | 213.32 | 0.3517 |  | No | Class II |
| 1334 | 1348 | LNGYTVEEAKTVLKK | 285.9 | -0.1439 |  | No | Class II |
| 1342 | 1356 | AKTVLKKCKSAFYIL | 128.42 | -0.0984 |  | No | Class II |
| 1347 | 1361 | KKCKSAFYILPSIIS | 263.96 | 0.4946 |  | No | Class II |
| 1352 | 1366 | AFYILPSIISNEKQE | 533.76 | 0.8262 |  | No | Class II |
| 1356 | 1370 | LPSIISNEKQEILGT | 341.3 | 0.7215 |  | No | Class II |
| 1362 | 1376 | NEKQEILGTVSWNLR | 312.86 | 1.5531 |  | No | Class II |
| 1367 | 1381 | ILGTVSWNLREMLAH | 656.73 | 1.0431 |  | No | Class II |
| 1372 | 1386 | SWNLREMLAHAEETR | 774.01 | 0.8303 |  | No | Class II |
| 1375 | 1389 | LREMLAHAEETRKLM | 227.41 | -0.0643 |  | No | Class II |
| 1383 | 1397 | EETRKLMPVCVETKA | 806.16 | 1.0702 |  | No | Class II |
| 1388 | 1402 | LMPVCVETKAIVSTI | 442.88 | 0.5606 |  | No | Class II |
| 1391 | 1405 | VCVETKAIVSTIQRK | 672.02 | 0.7548 |  | No | Class II |
| 1395 | 1409 | TKAIVSTIQRKYKGI | 119.45 | 0.6581 |  | No | Class II |
| 1403 | 1417 | QRKYKGIKIQEGVVD | 154.6 | 0.5975 |  | No | Class II |
| 1411 | 1425 | IQEGVVDYGARFYFY | 662.04 | 0.5255 |  | No | Class II |
| 1416 | 1430 | VDYGARFYFYTSKTT | 52.81 | 0.7523 |  | No | Class II |
| 1421 | 1435 | RFYFYTSKTTVASLI | 125.53 | 0.2811 |  | No | Class II |
| 1426 | 1440 | TSKTTVASLINTLND | 366.04 | 0.3378 |  | No | Class II |
| 1428 | 1442 | KTTVASLINTLNDLN | 75.23 | 0.5793 |  | No | Class II |
| 1432 | 1446 | ASLINTLNDLNETLV | 415.53 | 0.4308 |  | No | Class II |
| 1433 | 1447 | SLINTLNDLNETLVT | 257.56 | 0.3949 |  | No | Class II |
| 1439 | 1453 | NDLNETLVTMPLGYV | 388.65 | 0.5673 |  | No | Class II |
| 1441 | 1455 | LNETLVTMPLGYVTH | 172.24 | 0.5762 |  | No | Class II |
| 1448 | 1462 | MPLGYVTHGLNLEEA | 194.51 | 1.1412 |  | No | Class II |
| 1451 | 1465 | GYVTHGLNLEEAARY | 172.39 | 0.7127 |  | No | Class II |
| 1454 | 1468 | THGLNLEEAARYMRS | 115.02 | 0.3367 |  | No | Class II |
| 1457 | 1471 | LNLEEAARYMRSLKV | 596.99 | 0.4538 |  | No | Class II |
| 1462 | 1476 | AARYMRSLKVPATVS | 41.63 | 0.2773 |  | No | Class II |
| 1463 | 1477 | ARYMRSLKVPATVSV | 330.14 | 0.2741 |  | No | Class II |
| 1467 | 1481 | RSLKVPATVSVSSPD | 597.16 | 0.4333 |  | No | Class II |
| 1469 | 1483 | LKVPATVSVSSPDAV | 234.24 | 0.2859 |  | No | Class II |
| 1472 | 1486 | PATVSVSSPDAVTAY | 893.54 | 0.4967 |  | No | Class II |
| 1474 | 1488 | TVSVSSPDAVTAYNG | 319.61 | 0.2984 |  | No | Class II |
| 1480 | 1494 | PDAVTAYNGYLTSSS | 42.39 | 0.2962 |  | No | Class II |
| 1482 | 1496 | AVTAYNGYLTSSSKT | 323.97 | 0.4935 |  | No | Class II |
| 1485 | 1499 | AYNGYLTSSSKTPEE | 412.19 | 0.3762 |  | No | Class II |
| 1498 | 1512 | EEHFIETISLAGSYK | 39.91 | 0.3968 |  | No | Class II |
| 1499 | 1513 | EHFIETISLAGSYKD | 125.96 | 0.3726 |  | No | Class II |
| 1503 | 1517 | ETISLAGSYKDWSYS | 180.53 | 1.1073 |  | No | Class II |
| 1513 | 1527 | DWSYSGQSTQLGIEF | 138.92 | 1.8447 |  | No | Class II |
| 1514 | 1528 | WSYSGQSTQLGIEFL | 876.51 | 1.4229 |  | No | Class II |
| 1522 | 1536 | QLGIEFLKRGDKSVY | 102.79 | 1.3374 |  | No | Class II |
| 1527 | 1541 | FLKRGDKSVYYTSNP | 638.46 | 0.7184 |  | No | Class II |
| 1532 | 1546 | DKSVYYTSNPTTFHL | 849.34 | 0.5032 |  | No | Class II |
| 1540 | 1554 | NPTTFHLDGEVITFD | 436.36 | 0.1079 |  | No | Class II |
| 1545 | 1559 | HLDGEVITFDNLKTL | 51.32 | -0.016 |  | No | Class II |
| 1556 | 1570 | LKTLLSLREVRTIKV | 71.99 | 0.6373 |  | No | Class II |
| 1562 | 1576 | LREVRTIKVFTTVDN | 346.8 | 0.1634 |  | No | Class II |
| 1565 | 1579 | VRTIKVFTTVDNINL | 345.8 | 0.3619 |  | No | Class II |
| 1570 | 1584 | VFTTVDNINLHTQVV | 368.78 | 0.4624 |  | No | Class II |
| 1575 | 1589 | DNINLHTQVVDMSMT | 305.22 | 0.9347 |  | No | Class II |
| 1576 | 1590 | NINLHTQVVDMSMTY | 389.84 | 1.0335 |  | No | Class II |
| 1581 | 1595 | TQVVDMSMTYGQQFG | 562.23 | 1.0135 |  | No | Class II |
| 1586 | 1600 | MSMTYGQQFGPTYLD | 947.43 | 0.9985 |  | No | Class II |
| 1594 | 1608 | FGPTYLDGADVTKIK | 660.18 | 0.8186 |  | No | Class II |
| 1596 | 1610 | PTYLDGADVTKIKPH | 248.58 | 0.6202 |  | No | Class II |
| 1604 | 1618 | VTKIKPHNSHEGKTF | 294.88 | 0.4436 |  | No | Class II |
| 1608 | 1622 | KPHNSHEGKTFYVLP | 660.33 | 0.3115 |  | No | Class II |
| 1613 | 1627 | HEGKTFYVLPNDDTL | 815.72 | -0.055 |  | No | Class II |
| 1615 | 1629 | GKTFYVLPNDDTLRV | 37.91 | -0.2448 |  | No | Class II |
| 1621 | 1635 | LPNDDTLRVEAFEYY | 312.47 | -0.1761 |  | No | Class II |
| 1624 | 1638 | DDTLRVEAFEYYHTT | 824 | 0.3136 |  | No | Class II |
| 1629 | 1643 | VEAFEYYHTTDPSFL | 649.39 | 0.8309 |  | No | Class II |
| 1640 | 1654 | PSFLGRYMSALNHTK | 863.34 | 0.7798 |  | No | Class II |
| 1645 | 1659 | RYMSALNHTKKWKYP | 64.2 | 0.3024 |  | No | Class II |
| 1651 | 1665 | NHTKKWKYPQVNGLT | 594.66 | 0.0169 |  | No | Class II |
| 1656 | 1670 | WKYPQVNGLTSIKWA | 156.95 | 0.9219 |  | No | Class II |
| 1659 | 1673 | PQVNGLTSIKWADNN | 216.51 | 1.0364 |  | No | Class II |
| 1671 | 1685 | DNNCYLATALLTLQQ | 331 | 0.6103 |  | No | Class II |
| 1676 | 1690 | LATALLTLQQIELKF | 828.42 | 1.1902 |  | No | Class II |
| 1677 | 1691 | ATALLTLQQIELKFN | 170.88 | 1.2763 |  | No | Class II |
| 1691 | 1705 | NPPALQDAYYRARAG | 606.41 | 0.6794 |  | No | Class II |
| 1702 | 1716 | ARAGEAANFCALILA | 917.72 | 0.8328 |  | No | Class II |
| 1703 | 1717 | RAGEAANFCALILAY | 415.25 | 0.7984 |  | No | Class II |
| 1708 | 1722 | ANFCALILAYCNKTV | 478.35 | 0.5073 |  | No | Class II |
| 1725 | 1739 | LGDVRETMSYLFQHA | 54.4 | 0.492 |  | No | Class II |
| 1728 | 1742 | VRETMSYLFQHANLD | 772.8 | 0.5508 |  | No | Class II |
| 1731 | 1745 | TMSYLFQHANLDSCK | 30.57 | -0.132 |  | No | Class II |
| 1737 | 1751 | QHANLDSCKRVLNVV | 334.53 | -0.0785 |  | No | Class II |
| 1745 | 1759 | KRVLNVVCKTCGQQQ | 210.64 | 0.4721 |  | No | Class II |
| 1757 | 1771 | QQQTTLKGVEAVMYM | 66.16 | 0.6018 |  | No | Class II |
| 1759 | 1773 | QTTLKGVEAVMYMGT | 388.17 | 0.59 |  | No | Class II |
| 1765 | 1779 | VEAVMYMGTLSYEQF | 451.53 | 0.6745 |  | No | Class II |
| 1772 | 1786 | GTLSYEQFKKGVQIP | 178.12 | 0.5485 |  | No | Class II |
| 1788 | 1802 | TCGKQATKYLVQQES | 882.43 | 0.038 |  | No | Class II |
| 1792 | 1806 | QATKYLVQQESPFVM | 623.4 | 0.1913 |  | No | Class II |
| 1794 | 1808 | TKYLVQQESPFVMMS | 142.34 | 0.3119 |  | No | Class II |
| 1797 | 1811 | LVQQESPFVMMSAPP | 459.72 | 0.5772 |  | No | Class II |
| 1802 | 1816 | SPFVMMSAPPAQYEL | 91.58 | 0.5833 |  | No | Class II |
| 1805 | 1819 | VMMSAPPAQYELKHG | 409.68 | 0.9431 |  | No | Class II |
| 1813 | 1827 | QYELKHGTFTCASEY | 702.12 | 1.0158 |  | No | Class II |
| 1814 | 1828 | YELKHGTFTCASEYT | 411.82 | 1.0431 |  | No | Class II |
| 1818 | 1832 | HGTFTCASEYTGNYQ | 568.26 | 0.479 |  | No | Class II |
| 1832 | 1846 | QCGHYKHITSKETLY | 123.16 | 0.2405 |  | No | Class II |
| 1840 | 1854 | TSKETLYCIDGALLT | 746.37 | 0.7601 |  | No | Class II |
| 1843 | 1857 | ETLYCIDGALLTKSS | 68.57 | 0.6515 |  | No | Class II |
| 1845 | 1859 | LYCIDGALLTKSSEY | 126.63 | 0.759 |  | No | Class II |
| 1850 | 1864 | GALLTKSSEYKGPIT | 793.37 | 0.3465 |  | No | Class II |
| 1864 | 1878 | TDVFYKENSYTTTIK | 124.15 | 0.2717 |  | No | Class II |
| 1868 | 1882 | YKENSYTTTIKPVTY | 505.83 | 0.689 |  | No | Class II |
| 1871 | 1885 | NSYTTTIKPVTYKLD | 84.7 | 0.3274 |  | No | Class II |
| 1878 | 1892 | KPVTYKLDGVVCTEI | 60.73 | 0.3519 |  | No | Class II |
| 1887 | 1901 | VVCTEIDPKLDNYYK | 755.44 | 1.1009 |  | No | Class II |
| 1896 | 1910 | LDNYYKKDNSYFTEQ | 175.5 | 0.4533 |  | No | Class II |
| 1901 | 1915 | KKDNSYFTEQPIDLV | 330.36 | 1.003 |  | No | Class II |
| 1906 | 1920 | YFTEQPIDLVPNQPY | 978.67 | 0.742 |  | No | Class II |
| 1911 | 1925 | PIDLVPNQPYPNASF | 84.03 | 0.8308 |  | No | Class II |
| 1917 | 1931 | NQPYPNASFDNFKFV | 737.18 | 0.5766 |  | No | Class II |
| 1922 | 1936 | NASFDNFKFVCDNIK | 542.68 | 0.8981 |  | No | Class II |
| 1927 | 1941 | NFKFVCDNIKFADDL | 98.41 | 1.2057 |  | No | Class II |
| 1934 | 1948 | NIKFADDLNQLTGYK | 81.79 | 0.4849 |  | No | Class II |
| 1941 | 1955 | LNQLTGYKKPASREL | 331.31 | -0.2829 |  | No | Class II |
| 1946 | 1960 | GYKKPASRELKVTFF | 750.81 | 0.3218 |  | No | Class II |
| 1951 | 1965 | ASRELKVTFFPDLNG | 850.9 | 0.7467 |  | No | Class II |
| 1954 | 1968 | ELKVTFFPDLNGDVV | 111 | 0.5332 |  | No | Class II |
| 1956 | 1970 | KVTFFPDLNGDVVAI | 79.36 | 0.2998 |  | No | Class II |
| 1959 | 1973 | FFPDLNGDVVAIDYK | 406.85 | 0.6803 |  | No | Class II |
| 1965 | 1979 | GDVVAIDYKHYTPSF | 103.09 | 1.3734 |  | No | Class II |
| 1971 | 1985 | DYKHYTPSFKKGAKL | 66.67 | 0.9035 |  | No | Class II |
| 1976 | 1990 | TPSFKKGAKLLHKPI | 33.07 | 0.3161 |  | No | Class II |
| 1981 | 1995 | KGAKLLHKPIVWHVN | 286.46 | 0.5286 |  | No | Class II |
| 1987 | 2001 | HKPIVWHVNNATNKA | 74.13 | 0.3803 |  | No | Class II |
| 1990 | 2004 | IVWHVNNATNKATYK | 568.95 | 0.3093 |  | No | Class II |
| 1992 | 2006 | WHVNNATNKATYKPN | 702.16 | 0.4359 |  | No | Class II |
| 2000 | 2014 | KATYKPNTWCIRCLW | 913.39 | 1.7503 |  | No | Class II |
| 2008 | 2022 | WCIRCLWSTKPVETS | 227.5 | 1.7422 |  | No | Class II |
| 2010 | 2024 | IRCLWSTKPVETSNS | 778.32 | 1.1876 |  | No | Class II |
| 2016 | 2030 | TKPVETSNSFDVLKS | 153.19 | 0.4982 |  | No | Class II |
| 2019 | 2033 | VETSNSFDVLKSEDA | 456.69 | 0.6356 |  | No | Class II |
| 2022 | 2036 | SNSFDVLKSEDAQGM | 29.25 | 0.2186 |  | No | Class II |
| 2025 | 2039 | FDVLKSEDAQGMDNL | 997.03 | 0.2591 |  | No | Class II |
| 2042 | 2056 | EDLKPVSEEVVENPT | 963.6 | 0.3502 |  | No | Class II |
| 2048 | 2062 | SEEVVENPTIQKDVL | 862.16 | -0.5918 |  | No | Class II |
| 2054 | 2068 | NPTIQKDVLECNVKT | 699.32 | -0.078 |  | No | Class II |
| 2065 | 2079 | NVKTTEVVGDIILKP | 714.36 | 0.5938 |  | No | Class II |
| 2068 | 2082 | TTEVVGDIILKPANN | 118.17 | 0.1935 |  | No | Class II |
| 2086 | 2100 | ITEEVGHTDLMAAYV | 175.17 | 0.496 |  | No | Class II |
| 2090 | 2104 | VGHTDLMAAYVDNSS | 672.58 | 0.5585 |  | No | Class II |
| 2093 | 2107 | TDLMAAYVDNSSLTI | 129.17 | 0.5737 |  | No | Class II |
| 2095 | 2109 | LMAAYVDNSSLTIKK | 116.36 | 0.7068 |  | No | Class II |
| 2099 | 2113 | YVDNSSLTIKKPNEL | 767.92 | 0.6438 |  | No | Class II |
| 2103 | 2117 | SSLTIKKPNELSRVL | 254.67 | 0.5524 |  | No | Class II |
| 2108 | 2122 | KKPNELSRVLGLKTL | 103.5 | 0.4348 |  | No | Class II |
| 2113 | 2127 | LSRVLGLKTLATHGL | 519.43 | -0.0432 |  | No | Class II |
| 2118 | 2132 | GLKTLATHGLAAVNS | 315.58 | 0.3685 |  | No | Class II |
| 2120 | 2134 | KTLATHGLAAVNSVP | 28.05 | 0.3683 |  | No | Class II |
| 2124 | 2138 | THGLAAVNSVPWDTI | 155.2 | 0.501 |  | No | Class II |
| 2125 | 2139 | HGLAAVNSVPWDTIA | 362.78 | 0.5768 |  | No | Class II |
| 2131 | 2145 | NSVPWDTIANYAKPF | 782.92 | 0.1258 |  | No | Class II |
| 2132 | 2146 | SVPWDTIANYAKPFL | 270.31 | 0.009 |  | No | Class II |
| 2136 | 2150 | DTIANYAKPFLNKVV | 68.82 | -0.5102 |  | No | Class II |
| 2137 | 2151 | TIANYAKPFLNKVVS | 50.82 | -0.2857 |  | No | Class II |
| 2145 | 2159 | FLNKVVSTTTNIVTR | 854.38 | 0.1293 |  | No | Class II |
| 2150 | 2164 | VSTTTNIVTRCLNRV | 682.62 | 0.2494 |  | No | Class II |
| 2152 | 2166 | TTTNIVTRCLNRVCT | 137.09 | -0.0228 |  | No | Class II |
| 2158 | 2172 | TRCLNRVCTNYMPYF | 826.09 | 0.0059 |  | No | Class II |
| 2163 | 2177 | RVCTNYMPYFFTLLL | 669.66 | 0.0395 |  | No | Class II |
| 2168 | 2182 | YMPYFFTLLLQLCTF | 314.45 | 0.0181 |  | No | Class II |
| 2173 | 2187 | FTLLLQLCTFTRSTN | 376.83 | -0.091 |  | No | Class II |
| 2178 | 2192 | QLCTFTRSTNSRIKA | 13.04 | -0.2094 |  | No | Class II |
| 2182 | 2196 | FTRSTNSRIKASMPT | 286.13 | -0.0616 |  | No | Class II |
| 2186 | 2200 | TNSRIKASMPTTIAK | 22.81 | 0.3452 |  | No | Class II |
| 2187 | 2201 | NSRIKASMPTTIAKN | 54.01 | 0.345 |  | No | Class II |
| 2191 | 2205 | KASMPTTIAKNTVKS | 691.6 | 0.3375 |  | No | Class II |
| 2196 | 2210 | TTIAKNTVKSVGKFC | 34.51 | 0.2261 |  | No | Class II |
| 2201 | 2215 | NTVKSVGKFCLEASF | 270.79 | 0.6412 |  | No | Class II |
| 2206 | 2220 | VGKFCLEASFNYLKS | 115.43 | 0.6732 |  | No | Class II |
| 2219 | 2233 | KSPNFSKLINIIIWF | 86.2 | 0.3191 |  | No | Class II |
| 2224 | 2238 | SKLINIIIWFLLLSV | 733.24 | 0.3483 |  | No | Class II |
| 2229 | 2243 | IIIWFLLLSVCLGSL | 940.03 | 0.4122 |  | No | Class II |
| 2234 | 2248 | LLLSVCLGSLIYSTA | 551.59 | 0.276 |  | No | Class II |
| 2235 | 2249 | LLSVCLGSLIYSTAA | 119.98 | 0.4034 |  | No | Class II |
| 2242 | 2256 | SLIYSTAALGVLMSN | 120.95 | 0.4843 |  | No | Class II |
| 2247 | 2261 | TAALGVLMSNLGMPS | 462.99 | 0.6384 |  | No | Class II |
| 2252 | 2266 | VLMSNLGMPSYCTGY | 317.04 | 0.2943 |  | No | Class II |
| 2260 | 2274 | PSYCTGYREGYLNST | 663.24 | 0.2608 |  | No | Class II |
| 2262 | 2276 | YCTGYREGYLNSTNV | 743.44 | 0.4956 |  | No | Class II |
| 2267 | 2281 | REGYLNSTNVTIATY | 292.07 | 0.885 |  | No | Class II |
| 2272 | 2286 | NSTNVTIATYCTGSI | 551.8 | 0.5664 |  | No | Class II |
| 2275 | 2289 | NVTIATYCTGSIPCS | 173.12 | 0.4086 |  | No | Class II |
| 2278 | 2292 | IATYCTGSIPCSVCL | 126.7 | 0.1429 |  | No | Class II |
| 2280 | 2294 | TYCTGSIPCSVCLSG | 982.78 | 0.0059 |  | No | Class II |
| 2287 | 2301 | PCSVCLSGLDSLDTY | 846.47 | 0.3415 |  | No | Class II |
| 2292 | 2306 | LSGLDSLDTYPSLET | 110.99 | 0.7122 |  | No | Class II |
| 2301 | 2315 | YPSLETIQITISSFK | 344.96 | 0.765 |  | No | Class II |
| 2306 | 2320 | TIQITISSFKWDLTA | 759.4 | 1.0476 |  | No | Class II |
| 2314 | 2328 | FKWDLTAFGLVAEWF | 195.76 | 0.8059 |  | No | Class II |
| 2317 | 2331 | DLTAFGLVAEWFLAY | 917.23 | 0.5488 |  | No | Class II |
| 2322 | 2336 | GLVAEWFLAYILFTR | 385.27 | 0.3272 |  | No | Class II |
| 2323 | 2337 | LVAEWFLAYILFTRF | 417.03 | 0.1098 |  | No | Class II |
| 2328 | 2342 | FLAYILFTRFFYVLG | 793.43 | 0.2179 |  | No | Class II |
| 2334 | 2348 | FTRFFYVLGLAAIMQ | 413.93 | 0.2273 |  | No | Class II |
| 2339 | 2353 | YVLGLAAIMQLFFSY | 759.48 | 0.4027 |  | No | Class II |
| 2342 | 2356 | GLAAIMQLFFSYFAV | 847.39 | 0.3266 |  | No | Class II |
| 2346 | 2360 | IMQLFFSYFAVHFIS | 127.5 | 0.2072 |  | No | Class II |
| 2347 | 2361 | MQLFFSYFAVHFISN | 246.49 | 0.2033 |  | No | Class II |
| 2351 | 2365 | FSYFAVHFISNSWLM | 257.8 | 0.4114 |  | No | Class II |
| 2354 | 2368 | FAVHFISNSWLMWLI | 611.38 | 0.3286 |  | No | Class II |
| 2356 | 2370 | VHFISNSWLMWLIIN | 364.14 | 0.4643 |  | No | Class II |
| 2363 | 2377 | WLMWLIINLVQMAPI | 462.71 | 0.9777 |  | No | Class II |
| 2365 | 2379 | MWLIINLVQMAPISA | 75.4 | 0.9266 |  | No | Class II |
| 2368 | 2382 | IINLVQMAPISAMVR | 95.09 | 0.7682 |  | No | Class II |
| 2371 | 2385 | LVQMAPISAMVRMYI | 109.28 | 0.3885 |  | No | Class II |
| 2376 | 2390 | PISAMVRMYIFFASF | 267.53 | 0.2206 |  | No | Class II |
| 2377 | 2391 | ISAMVRMYIFFASFY | 787.89 | 0.1481 |  | No | Class II |
| 2382 | 2396 | RMYIFFASFYYVWKS | 612.81 | 0.1656 |  | No | Class II |
| 2387 | 2401 | FASFYYVWKSYVHVV | 586.5 | 0.1829 |  | No | Class II |
| 2392 | 2406 | YVWKSYVHVVDGCNS | 882.91 | -0.1529 |  | No | Class II |
| 2394 | 2408 | WKSYVHVVDGCNSST | 264.03 | -0.053 |  | No | Class II |
| 2401 | 2415 | VDGCNSSTCMMCYKR | 594.79 | 0.0359 |  | No | Class II |
| 2408 | 2422 | TCMMCYKRNRATRVE | 100.71 | 0.8512 |  | No | Class II |
| 2418 | 2432 | ATRVECTTIVNGVRR | 773.9 | 0.6025 |  | No | Class II |
| 2419 | 2433 | TRVECTTIVNGVRRS | 283.2 | 0.7409 |  | No | Class II |
| 2424 | 2438 | TTIVNGVRRSFYVYA | 18.41 | 0.2001 |  | No | Class II |
| 2430 | 2444 | VRRSFYVYANGGKGF | 84.67 | 0.1669 |  | No | Class II |
| 2432 | 2446 | RSFYVYANGGKGFCK | 720.47 | -0.1338 |  | No | Class II |
| 2435 | 2449 | YVYANGGKGFCKLHN | 384.76 | 0.1411 |  | No | Class II |
| 2440 | 2454 | GGKGFCKLHNWNCVN | 318.21 | 1.0043 |  | No | Class II |
| 2455 | 2469 | CDTFCAGSTFISDEV | 467.68 | -0.0374 |  | No | Class II |
| 2456 | 2470 | DTFCAGSTFISDEVA | 72.77 | -0.0408 |  | No | Class II |
| 2460 | 2474 | AGSTFISDEVARDLS | 166.5 | -0.2017 |  | No | Class II |
| 2461 | 2475 | GSTFISDEVARDLSL | 222.24 | 0.0168 |  | No | Class II |
| 2466 | 2480 | SDEVARDLSLQFKRP | 746.85 | 0.4803 |  | No | Class II |
| 2470 | 2484 | ARDLSLQFKRPINPT | 64.84 | 1.1967 |  | No | Class II |
| 2484 | 2498 | TDQSSYIVDSVTVKN | 922.95 | 0.586 |  | No | Class II |
| 2491 | 2505 | VDSVTVKNGSIHLYF | 417.67 | 0.9579 |  | No | Class II |
| 2496 | 2510 | VKNGSIHLYFDKAGQ | 523.17 | 0.6086 |  | No | Class II |
| 2499 | 2513 | GSIHLYFDKAGQKTY | 93.08 | 0.5093 |  | No | Class II |
| 2510 | 2524 | QKTYERHSLSHFVNL | 36.45 | 0.3384 |  | No | Class II |
| 2515 | 2529 | RHSLSHFVNLDNLRA | 822.65 | 0.3481 |  | No | Class II |
| 2524 | 2538 | LDNLRANNTKGSLPI | 210.14 | 0.5124 |  | No | Class II |
| 2529 | 2543 | ANNTKGSLPINVIVF | 101.02 | 0.425 |  | No | Class II |
| 2536 | 2550 | LPINVIVFDGKSKCE | 125.88 | 0.6261 |  | No | Class II |
| 2546 | 2560 | KSKCEESSAKSASVY | 328.9 | 0.4695 |  | No | Class II |
| 2548 | 2562 | KCEESSAKSASVYYS | 456.43 | 0.4309 |  | No | Class II |
| 2551 | 2565 | ESSAKSASVYYSQLM | 45.81 | 0.3903 |  | No | Class II |
| 2553 | 2567 | SAKSASVYYSQLMCQ | 234.23 | 0.6888 |  | No | Class II |
| 2558 | 2572 | SVYYSQLMCQPILLL | 495.93 | 0.591 |  | No | Class II |
| 2560 | 2574 | YYSQLMCQPILLLDQ | 729.56 | 0.4935 |  | No | Class II |
| 2565 | 2579 | MCQPILLLDQALVSD | 804.78 | 0.0537 |  | No | Class II |
| 2567 | 2581 | QPILLLDQALVSDVG | 44.01 | 0.3385 |  | No | Class II |
| 2574 | 2588 | QALVSDVGDSAEVAV | 665.06 | 0.2471 |  | No | Class II |
| 2580 | 2594 | VGDSAEVAVKMFDAY | 330.52 | 0.3392 |  | No | Class II |
| 2585 | 2599 | EVAVKMFDAYVNTFS | 507.22 | -0.2788 |  | No | Class II |
| 2590 | 2604 | MFDAYVNTFSSTFNV | 713.74 | -0.3922 |  | No | Class II |
| 2595 | 2609 | VNTFSSTFNVPMEKL | 601.2 | -0.5684 |  | No | Class II |
| 2602 | 2616 | FNVPMEKLKTLVATA | 53.08 | -0.0723 |  | No | Class II |
| 2607 | 2621 | EKLKTLVATAEAELA | 111.03 | 0.2953 |  | No | Class II |
| 2608 | 2622 | KLKTLVATAEAELAK | 53.62 | 0.1314 |  | No | Class II |
| 2612 | 2626 | LVATAEAELAKNVSL | 117.72 | 0.5172 |  | No | Class II |
| 2616 | 2630 | AEAELAKNVSLDNVL | 84.71 | 0.247 |  | No | Class II |
| 2621 | 2635 | AKNVSLDNVLSTFIS | 62 | 0.4234 |  | No | Class II |
| 2626 | 2640 | LDNVLSTFISAARQG | 136.35 | 0.3303 |  | No | Class II |
| 2628 | 2642 | NVLSTFISAARQGFV | 115.01 | 0.2764 |  | No | Class II |
| 2633 | 2647 | FISAARQGFVDSDVE | 621.93 | 0.6164 |  | No | Class II |
| 2647 | 2661 | ETKDVVECLKLSHQS | 358.64 | 1.4358 |  | No | Class II |
| 2652 | 2666 | VECLKLSHQSDIEVT | 174.21 | 1.6521 |  | No | Class II |
| 2662 | 2676 | DIEVTGDSCNNYMLT | 764.36 | 0.269 |  | No | Class II |
| 2667 | 2681 | GDSCNNYMLTYNKVE | 429.17 | 0.1879 |  | No | Class II |
| 2672 | 2686 | NYMLTYNKVENMTPR | 51.26 | 0.5131 |  | No | Class II |
| 2677 | 2691 | YNKVENMTPRDLGAC | 581.8 | 0.974 |  | No | Class II |
| 2684 | 2698 | TPRDLGACIDCSARH | 931.42 | 0.8906 |  | No | Class II |
| 2687 | 2701 | DLGACIDCSARHINA | 949.61 | 0.6376 |  | No | Class II |
| 2693 | 2707 | DCSARHINAQVAKSH | 312.88 | 0.1756 |  | No | Class II |
| 2702 | 2716 | QVAKSHNIALIWNVK | 309.82 | 1.1078 |  | No | Class II |
| 2706 | 2720 | SHNIALIWNVKDFMS | 222.98 | 0.8168 |  | No | Class II |
| 2707 | 2721 | HNIALIWNVKDFMSL | 316.06 | 1.0405 |  | No | Class II |
| 2711 | 2725 | LIWNVKDFMSLSEQL | 826.48 | 0.8064 |  | No | Class II |
| 2712 | 2726 | IWNVKDFMSLSEQLR | 550.64 | 0.7964 |  | No | Class II |
| 2724 | 2738 | QLRKQIRSAAKKNNL | 62.7 | 0.4485 |  | No | Class II |
| 2729 | 2743 | IRSAAKKNNLPFKLT | 318.18 | 1.2451 |  | No | Class II |
| 2734 | 2748 | KKNNLPFKLTCATTR | 526.12 | 1.606 |  | No | Class II |
| 2737 | 2751 | NLPFKLTCATTRQVV | 169.55 | 1.1632 |  | No | Class II |
| 2739 | 2753 | PFKLTCATTRQVVNV | 392.59 | 0.9882 |  | No | Class II |
| 2742 | 2756 | LTCATTRQVVNVVTT | 749.07 | 0.6049 |  | No | Class II |
| 2747 | 2761 | TRQVVNVVTTKIALK | 99.35 | 0.6678 |  | No | Class II |
| 2748 | 2762 | RQVVNVVTTKIALKG | 774.31 | 0.6794 |  | No | Class II |
| 2753 | 2767 | VVTTKIALKGGKIVN | 67.94 | 1.192 |  | No | Class II |
| 2757 | 2771 | KIALKGGKIVNNWLK | 73.82 | 0.4733 |  | No | Class II |
| 2762 | 2776 | GGKIVNNWLKQLIKV | 29.76 | -0.466 |  | No | Class II |
| 2767 | 2781 | NNWLKQLIKVTLVFL | 240.57 | 0.0016 |  | No | Class II |
| 2779 | 2793 | VFLFVAAIFYLITPV | 895.75 | 0.1478 |  | No | Class II |
| 2781 | 2795 | LFVAAIFYLITPVHV | 599.61 | 0.2925 |  | No | Class II |
| 2784 | 2798 | AAIFYLITPVHVMSK | 75.81 | 0.2703 |  | No | Class II |
| 2786 | 2800 | IFYLITPVHVMSKHT | 589.65 | 0.3448 |  | No | Class II |
| 2789 | 2803 | LITPVHVMSKHTDFS | 300.79 | 1.0217 |  | No | Class II |
| 2798 | 2812 | KHTDFSSEIIGYKAI | 895.66 | 0.9912 |  | No | Class II |
| 2808 | 2822 | GYKAIDGGVTRDIAS | 214.42 | 0.3105 |  | No | Class II |
| 2809 | 2823 | YKAIDGGVTRDIAST | 66.54 | 0.3257 |  | No | Class II |
| 2817 | 2831 | TRDIASTDTCFANKH | 673.01 | 0.7568 |  | No | Class II |
| 2831 | 2845 | HADFDTWFSQRGGSY | 862.28 | 0.3219 |  | No | Class II |
| 2836 | 2850 | TWFSQRGGSYTNDKA | 679 | 0.6999 |  | No | Class II |
| 2842 | 2856 | GGSYTNDKACPLIAA | 641.37 | 1.0317 |  | No | Class II |
| 2844 | 2858 | SYTNDKACPLIAAVI | 196.15 | 0.9863 |  | No | Class II |
| 2847 | 2861 | NDKACPLIAAVITRE | 494.36 | 1.1238 |  | No | Class II |
| 2852 | 2866 | PLIAAVITREVGFVV | 130.58 | 0.979 |  | No | Class II |
| 2857 | 2871 | VITREVGFVVPGLPG | 651.83 | 0.7171 |  | No | Class II |
| 2861 | 2875 | EVGFVVPGLPGTILR | 84.47 | 0.7955 |  | No | Class II |
| 2867 | 2881 | PGLPGTILRTTNGDF | 796.73 | 0.8902 |  | No | Class II |
| 2870 | 2884 | PGTILRTTNGDFLHF | 75.77 | 0.8217 |  | No | Class II |
| 2872 | 2886 | TILRTTNGDFLHFLP | 694.95 | 0.6897 |  | No | Class II |
| 2878 | 2892 | NGDFLHFLPRVFSAV | 427.88 | 0.4287 |  | No | Class II |
| 2883 | 2897 | HFLPRVFSAVGNICY | 445.36 | 0.6405 |  | No | Class II |
| 2886 | 2900 | PRVFSAVGNICYTPS | 248.58 | 0.7758 |  | No | Class II |
| 2898 | 2912 | TPSKLIEYTDFATSA | 426.68 | 0.6471 |  | No | Class II |
| 2903 | 2917 | IEYTDFATSACVLAA | 148.69 | 0.9882 |  | No | Class II |
| 2904 | 2918 | EYTDFATSACVLAAE | 67.55 | 0.7522 |  | No | Class II |
| 2909 | 2923 | ATSACVLAAECTIFK | 332.23 | 0.1571 |  | No | Class II |
| 2912 | 2926 | ACVLAAECTIFKDAS | 801.5 | 0.0581 |  | No | Class II |
| 2918 | 2932 | ECTIFKDASGKPVPY | 161.93 | -0.3101 |  | No | Class II |
| 2921 | 2935 | IFKDASGKPVPYCYD | 336.26 | 0.2356 |  | No | Class II |
| 2929 | 2943 | PVPYCYDTNVLEGSV | 137.63 | 0.4236 |  | No | Class II |
| 2935 | 2949 | DTNVLEGSVAYESLR | 27.55 | 0.3324 |  | No | Class II |
| 2949 | 2963 | RPDTRYVLMDGSIIQ | 772.97 | 0.1393 |  | No | Class II |
| 2950 | 2964 | PDTRYVLMDGSIIQF | 24.32 | 0.2706 |  | No | Class II |
| 2954 | 2968 | YVLMDGSIIQFPNTY | 137.51 | -0.1454 |  | No | Class II |
| 2959 | 2973 | GSIIQFPNTYLEGSV | 9.16 | -0.1924 |  | No | Class II |
| 2964 | 2978 | FPNTYLEGSVRVVTT | 157.04 | -0.0243 |  | No | Class II |
| 2965 | 2979 | PNTYLEGSVRVVTTF | 72.51 | -0.1563 |  | No | Class II |
| 2969 | 2983 | LEGSVRVVTTFDSEY | 57.96 | 0.2076 |  | No | Class II |
| 2970 | 2984 | EGSVRVVTTFDSEYC | 386.86 | 0.1427 |  | No | Class II |
| 2974 | 2988 | RVVTTFDSEYCRHGT | 999.07 | 0.2386 |  | No | Class II |
| 2981 | 2995 | SEYCRHGTCERSEAG | 422.04 | 0.207 |  | No | Class II |
| 2990 | 3004 | ERSEAGVCVSTSGRW | 81.69 | 0.6045 |  | No | Class II |
| 2993 | 3007 | EAGVCVSTSGRWVLN | 60.32 | 0.3325 |  | No | Class II |
| 3000 | 3014 | TSGRWVLNNDYYRSL | 87.61 | -0.0627 |  | No | Class II |
| 3007 | 3021 | NNDYYRSLPGVFCGV | 50.2 | 0.081 |  | No | Class II |
| 3008 | 3022 | NDYYRSLPGVFCGVD | 497.29 | 0.1873 |  | No | Class II |
| 3013 | 3027 | SLPGVFCGVDAVNLL | 855.56 | 0.5127 |  | No | Class II |
| 3014 | 3028 | LPGVFCGVDAVNLLT | 40.72 | 0.3298 |  | No | Class II |
| 3022 | 3036 | DAVNLLTNMFTPLIQ | 230.32 | -0.3435 |  | No | Class II |
| 3024 | 3038 | VNLLTNMFTPLIQPI | 429.92 | -0.3525 |  | No | Class II |
| 3027 | 3041 | LTNMFTPLIQPIGAL | 36.75 | -0.1099 |  | No | Class II |
| 3032 | 3046 | TPLIQPIGALDISAS | 314.2 | 0.6021 |  | No | Class II |
| 3037 | 3051 | PIGALDISASIVAGG | 51 | 1.028 |  | No | Class II |
| 3044 | 3058 | SASIVAGGIVAIVVT | 85.36 | 0.7537 |  | No | Class II |
| 3049 | 3063 | AGGIVAIVVTCLAYY | 617.6 | 0.6375 |  | No | Class II |
| 3054 | 3068 | AIVVTCLAYYFMRFR | 387.17 | 0.6202 |  | No | Class II |
| 3056 | 3070 | VVTCLAYYFMRFRRA | 515.6 | 0.3478 |  | No | Class II |
| 3059 | 3073 | CLAYYFMRFRRAFGE | 13.06 | -0.1485 |  | No | Class II |
| 3062 | 3076 | YYFMRFRRAFGEYSH | 780.06 | -0.0773 |  | No | Class II |
| 3064 | 3078 | FMRFRRAFGEYSHVV | 8.2 | -0.1045 |  | No | Class II |
| 3067 | 3081 | FRRAFGEYSHVVAFN | 626.88 | -0.1122 |  | No | Class II |
| 3071 | 3085 | FGEYSHVVAFNTLLF | 104.9 | 0.2858 |  | No | Class II |
| 3072 | 3086 | GEYSHVVAFNTLLFL | 535.07 | 0.3504 |  | No | Class II |
| 3076 | 3090 | HVVAFNTLLFLMSFT | 590.34 | 0.2622 |  | No | Class II |
| 3081 | 3095 | NTLLFLMSFTVLCLT | 917.9 | 0.9654 |  | No | Class II |
| 3086 | 3100 | LMSFTVLCLTPVYSF | 701.96 | 1.1432 |  | No | Class II |
| 3094 | 3108 | LTPVYSFLPGVYSVI | 552.21 | 0.3472 |  | No | Class II |
| 3095 | 3109 | TPVYSFLPGVYSVIY | 8.62 | 0.0273 |  | No | Class II |
| 3099 | 3113 | SFLPGVYSVIYLYLT | 959.36 | 0.6234 |  | No | Class II |
| 3100 | 3114 | FLPGVYSVIYLYLTF | 828.48 | 0.7509 |  | No | Class II |
| 3105 | 3119 | YSVIYLYLTFYLTND | 128.69 | 0.6447 |  | No | Class II |
| 3109 | 3123 | YLYLTFYLTNDVSFL | 145.56 | 0.8448 |  | No | Class II |
| 3110 | 3124 | LYLTFYLTNDVSFLA | 236.01 | 0.8067 |  | No | Class II |
| 3118 | 3132 | NDVSFLAHIQWMVMF | 373.36 | 0.9653 |  | No | Class II |
| 3124 | 3138 | AHIQWMVMFTPLVPF | 351.61 | 0.9031 |  | No | Class II |
| 3126 | 3140 | IQWMVMFTPLVPFWI | 275.67 | 0.5167 |  | No | Class II |
| 3131 | 3145 | MFTPLVPFWITIAYI | 252.5 | 0.6806 |  | No | Class II |
| 3136 | 3150 | VPFWITIAYIICIST | 182.77 | 0.8103 |  | No | Class II |
| 3142 | 3156 | IAYIICISTKHFYWF | 82.31 | 1.307 |  | No | Class II |
| 3150 | 3164 | TKHFYWFFSNYLKRR | 188.36 | 0.6182 |  | No | Class II |
| 3155 | 3169 | WFFSNYLKRRVVFNG | 179.45 | 0.0459 |  | No | Class II |
| 3161 | 3175 | LKRRVVFNGVSFSTF | 143.23 | 0.4503 |  | No | Class II |
| 3166 | 3180 | VFNGVSFSTFEEAAL | 236.62 | 0.3697 |  | No | Class II |
| 3171 | 3185 | SFSTFEEAALCTFLL | 463.09 | 0.0918 |  | No | Class II |
| 3175 | 3189 | FEEAALCTFLLNKEM | 925.01 | 0.1383 |  | No | Class II |
| 3177 | 3191 | EAALCTFLLNKEMYL | 478.66 | 0.0528 |  | No | Class II |
| 3180 | 3194 | LCTFLLNKEMYLKLR | 128.16 | 0.3182 |  | No | Class II |
| 3187 | 3201 | KEMYLKLRSDVLLPL | 277.04 | 0.3446 |  | No | Class II |
| 3192 | 3206 | KLRSDVLLPLTQYNR | 780.62 | 0.3798 |  | No | Class II |
| 3199 | 3213 | LPLTQYNRYLALYNK | 79.47 | 0.7527 |  | No | Class II |
| 3204 | 3218 | YNRYLALYNKYKYFS | 145.22 | 0.6622 |  | No | Class II |
| 3210 | 3224 | LYNKYKYFSGAMDTT | 31.76 | 0.3585 |  | No | Class II |
| 3213 | 3227 | KYKYFSGAMDTTSYR | 157.99 | 0.4624 |  | No | Class II |
| 3216 | 3230 | YFSGAMDTTSYREAA | 388.43 | 0.3045 |  | No | Class II |
| 3223 | 3237 | TTSYREAACCHLAKA | 251.75 | 0.9268 |  | No | Class II |
| 3224 | 3238 | TSYREAACCHLAKAL | 338.05 | 0.7496 |  | No | Class II |
| 3232 | 3246 | CHLAKALNDFSNSGS | 149.26 | 0.0026 |  | No | Class II |
| 3237 | 3251 | ALNDFSNSGSDVLYQ | 318.53 | 0.192 |  | No | Class II |
| 3240 | 3254 | DFSNSGSDVLYQPPQ | 76.43 | 0.3187 |  | No | Class II |
| 3246 | 3260 | SDVLYQPPQTSITSA | 144.13 | 0.2997 |  | No | Class II |
| 3249 | 3263 | LYQPPQTSITSAVLQ | 279.68 | 0.1469 |  | No | Class II |
| 3254 | 3268 | QTSITSAVLQSGFRK | 28.53 | 0.1571 |  | No | Class II |
| 3259 | 3273 | SAVLQSGFRKMAFPS | 146.19 | 0.2841 |  | No | Class II |
| 3264 | 3278 | SGFRKMAFPSGKVEG | 78.42 | 0.1995 |  | No | Class II |
| 3265 | 3279 | GFRKMAFPSGKVEGC | 714.97 | -0.0557 |  | No | Class II |
| 3270 | 3284 | AFPSGKVEGCMVQVT | 302.04 | 0.0847 |  | No | Class II |
| 3275 | 3289 | KVEGCMVQVTCGTTT | 682.55 | 0.4232 |  | No | Class II |
| 3277 | 3291 | EGCMVQVTCGTTTLN | 801.58 | 0.7175 |  | No | Class II |
| 3280 | 3294 | MVQVTCGTTTLNGLW | 372.22 | 0.6684 |  | No | Class II |
| 3285 | 3299 | CGTTTLNGLWLDDVV | 842.85 | 0.1895 |  | No | Class II |
| 3286 | 3300 | GTTTLNGLWLDDVVY | 146.31 | 0.3113 |  | No | Class II |
| 3290 | 3304 | LNGLWLDDVVYCPRH | 541.58 | 0.3792 |  | No | Class II |
| 3295 | 3309 | LDDVVYCPRHVICTS | 471.35 | 0.3431 |  | No | Class II |
| 3309 | 3323 | SEDMLNPNYEDLLIR | 995.81 | 0.7054 |  | No | Class II |
| 3311 | 3325 | DMLNPNYEDLLIRKS | 404.15 | 0.8952 |  | No | Class II |
| 3323 | 3337 | RKSNHNFLVQAGNVQ | 68.13 | 0.9041 |  | No | Class II |
| 3327 | 3341 | HNFLVQAGNVQLRVI | 69.56 | 0.7406 |  | No | Class II |
| 3328 | 3342 | NFLVQAGNVQLRVIG | 132.19 | 0.847 |  | No | Class II |
| 3335 | 3349 | NVQLRVIGHSMQNCV | 71.39 | 0.2289 |  | No | Class II |
| 3338 | 3352 | LRVIGHSMQNCVLKL | 792.61 | -0.3771 |  | No | Class II |
| 3340 | 3354 | VIGHSMQNCVLKLKV | 213.38 | 0.1944 |  | No | Class II |
| 3349 | 3363 | VLKLKVDTANPKTPK | 32.03 | -0.2549 |  | No | Class II |
| 3358 | 3372 | NPKTPKYKFVRIQPG | 29.11 | 0.0627 |  | No | Class II |
| 3360 | 3374 | KTPKYKFVRIQPGQT | 508.23 | 0.0142 |  | No | Class II |
| 3363 | 3377 | KYKFVRIQPGQTFSV | 6.78 | 0.1753 |  | No | Class II |
| 3365 | 3379 | KFVRIQPGQTFSVLA | 40.53 | 0.3213 |  | No | Class II |
| 3368 | 3382 | RIQPGQTFSVLACYN | 252.7 | 0.4873 |  | No | Class II |
| 3370 | 3384 | QPGQTFSVLACYNGS | 656.95 | 0.111 |  | No | Class II |
| 3375 | 3389 | FSVLACYNGSPSGVY | 125.73 | 0.3478 |  | No | Class II |
| 3376 | 3390 | SVLACYNGSPSGVYQ | 227.72 | 0.2787 |  | No | Class II |
| 3381 | 3395 | YNGSPSGVYQCAMRP | 681.17 | -0.1029 |  | No | Class II |
| 3386 | 3400 | SGVYQCAMRPNFTIK | 483.85 | 0.5868 |  | No | Class II |
| 3393 | 3407 | MRPNFTIKGSFLNGS | 251.87 | 1.0293 |  | No | Class II |
| 3396 | 3410 | NFTIKGSFLNGSCGS | 134.42 | 0.8048 |  | No | Class II |
| 3399 | 3413 | IKGSFLNGSCGSVGF | 387.06 | 0.9932 |  | No | Class II |
| 3401 | 3415 | GSFLNGSCGSVGFNI | 180.03 | 0.9703 |  | No | Class II |
| 3408 | 3422 | CGSVGFNIDYDCVSF | 727.24 | 1.6654 |  | No | Class II |
| 3410 | 3424 | SVGFNIDYDCVSFCY | 177.56 | 1.6358 |  | No | Class II |
| 3415 | 3429 | IDYDCVSFCYMHHME | 343.61 | 1.411 |  | No | Class II |
| 3417 | 3431 | YDCVSFCYMHHMELP | 904.82 | 0.982 |  | No | Class II |
| 3420 | 3434 | VSFCYMHHMELPTGV | 61.11 | 0.6623 |  | No | Class II |
| 3422 | 3436 | FCYMHHMELPTGVHA | 953.7 | 0.4924 |  | No | Class II |
| 3425 | 3439 | MHHMELPTGVHAGTD | 982.79 | 0.2822 |  | No | Class II |
| 3431 | 3445 | PTGVHAGTDLEGNFY | 657.11 | 0.221 |  | No | Class II |
| 3445 | 3459 | YGPFVDRQTAQAAGT | 672.23 | 0.7968 |  | No | Class II |
| 3448 | 3462 | FVDRQTAQAAGTDTT | 284.97 | 0.7289 |  | No | Class II |
| 3457 | 3471 | AGTDTTITVNVLAWL | 906.31 | 0.3401 |  | No | Class II |
| 3460 | 3474 | DTTITVNVLAWLYAA | 90.99 | 0.352 |  | No | Class II |
| 3463 | 3477 | ITVNVLAWLYAAVIN | 158.02 | 0.2509 |  | No | Class II |
| 3468 | 3482 | LAWLYAAVINGDRWF | 110.25 | -0.1121 |  | No | Class II |
| 3473 | 3487 | AAVINGDRWFLNRFT | 392.41 | -1.2257 |  | No | Class II |
| 3478 | 3492 | GDRWFLNRFTTTLND | 432.47 | -1.1108 |  | No | Class II |
| 3481 | 3495 | WFLNRFTTTLNDFNL | 663.78 | 0.1971 |  | No | Class II |
| 3483 | 3497 | LNRFTTTLNDFNLVA | 66.56 | 0.3709 |  | No | Class II |
| 3486 | 3500 | FTTTLNDFNLVAMKY | 366.83 | 1.1263 |  | No | Class II |
| 3489 | 3503 | TLNDFNLVAMKYNYE | 368.96 | 1.3972 |  | No | Class II |
| 3494 | 3508 | NLVAMKYNYEPLTQD | 295.61 | 1.0625 |  | No | Class II |
| 3499 | 3513 | KYNYEPLTQDHVDIL | 664.17 | 0.9406 |  | No | Class II |
| 3502 | 3516 | YEPLTQDHVDILGPL | 399.67 | 0.2311 |  | No | Class II |
| 3508 | 3522 | DHVDILGPLSAQTGI | 129.52 | 0.595 |  | No | Class II |
| 3511 | 3525 | DILGPLSAQTGIAVL | 182.06 | 0.6003 |  | No | Class II |
| 3513 | 3527 | LGPLSAQTGIAVLDM | 98.06 | 1.3554 |  | No | Class II |
| 3516 | 3530 | LSAQTGIAVLDMCAS | 206.3 | 0.559 |  | No | Class II |
| 3522 | 3536 | IAVLDMCASLKELLQ | 286.14 | 0.1239 |  | No | Class II |
| 3531 | 3545 | LKELLQNGMNGRTIL | 105.87 | -0.0333 |  | No | Class II |
| 3535 | 3549 | LQNGMNGRTILGSAL | 242.56 | 0.3459 |  | No | Class II |
| 3536 | 3550 | QNGMNGRTILGSALL | 468.91 | 0.2516 |  | No | Class II |
| 3540 | 3554 | NGRTILGSALLEDEF | 124.67 | -0.0241 |  | No | Class II |
| 3541 | 3555 | GRTILGSALLEDEFT | 682.9 | 0.0146 |  | No | Class II |
| 3545 | 3559 | LGSALLEDEFTPFDV | 358.27 | 0.946 |  | No | Class II |
| 3550 | 3564 | LEDEFTPFDVVRQCS | 205.85 | 0.6526 |  | No | Class II |
| 3557 | 3571 | FDVVRQCSGVTFQSA | 745.17 | 0.1128 |  | No | Class II |
| 3559 | 3573 | VVRQCSGVTFQSAVK | 330.14 | 0.0263 |  | No | Class II |
| 3563 | 3577 | CSGVTFQSAVKRTIK | 31.82 | 0.2082 |  | No | Class II |
| 3564 | 3578 | SGVTFQSAVKRTIKG | 512.88 | 0.2797 |  | No | Class II |
| 3568 | 3582 | FQSAVKRTIKGTHHW | 130.99 | 0.3755 |  | No | Class II |
| 3573 | 3587 | KRTIKGTHHWLLLTI | 89.87 | 0.7988 |  | No | Class II |
| 3574 | 3588 | RTIKGTHHWLLLTIL | 683.76 | 0.5541 |  | No | Class II |
| 3578 | 3592 | GTHHWLLLTILTSLL | 761.25 | 0.2398 |  | No | Class II |
| 3585 | 3599 | LTILTSLLVLVQSTQ | 380.91 | 0.4572 |  | No | Class II |
| 3590 | 3604 | SLLVLVQSTQWSLFF | 267.61 | 0.804 |  | No | Class II |
| 3595 | 3609 | VQSTQWSLFFFLYEN | 818.3 | 0.7509 |  | No | Class II |
| 3596 | 3610 | QSTQWSLFFFLYENA | 291.51 | 0.5759 |  | No | Class II |
| 3601 | 3615 | SLFFFLYENAFLPFA | 73.31 | 0.5134 |  | No | Class II |
| 3603 | 3617 | FFFLYENAFLPFAMG | 169.65 | 0.8851 |  | No | Class II |
| 3606 | 3620 | LYENAFLPFAMGIIA | 292.2 | 0.807 |  | No | Class II |
| 3616 | 3630 | MGIIAMSAFAMMFVK | 155.84 | 0.7353 |  | No | Class II |
| 3618 | 3632 | IIAMSAFAMMFVKHK | 249.2 | 0.6645 |  | No | Class II |
| 3622 | 3636 | SAFAMMFVKHKHAFL | 91.74 | 0.7305 |  | No | Class II |
| 3629 | 3643 | VKHKHAFLCLFLLPS | 875.07 | 1.0867 |  | No | Class II |
| 3631 | 3645 | HKHAFLCLFLLPSLA | 289.12 | 0.7464 |  | No | Class II |
| 3634 | 3648 | AFLCLFLLPSLATVA | 288.77 | 0.6254 |  | No | Class II |
| 3638 | 3652 | LFLLPSLATVAYFNM | 113.93 | 0.6553 |  | No | Class II |
| 3639 | 3653 | FLLPSLATVAYFNMV | 102.56 | 0.7173 |  | No | Class II |
| 3645 | 3659 | ATVAYFNMVYMPASW | 192.4 | 0.8025 |  | No | Class II |
| 3649 | 3663 | YFNMVYMPASWVMRI | 178.01 | 0.7244 |  | No | Class II |
| 3650 | 3664 | FNMVYMPASWVMRIM | 196.24 | 0.6579 |  | No | Class II |
| 3655 | 3669 | MPASWVMRIMTWLDM | 972.55 | 0.5001 |  | No | Class II |
| 3658 | 3672 | SWVMRIMTWLDMVDT | 443.39 | 0.4452 |  | No | Class II |
| 3660 | 3674 | VMRIMTWLDMVDTSL | 67.71 | 0.3834 |  | No | Class II |
| 3673 | 3687 | SLSGFKLKDCVMYAS | 691.38 | 0.4997 |  | No | Class II |
| 3678 | 3692 | KLKDCVMYASAVVLL | 404.13 | 0.2155 |  | No | Class II |
| 3683 | 3697 | VMYASAVVLLILMTA | 754.79 | 0.4247 |  | No | Class II |
| 3688 | 3702 | AVVLLILMTARTVYD | 65.58 | 0.6962 |  | No | Class II |
| 3691 | 3705 | LLILMTARTVYDDGA | 603.07 | 0.3364 |  | No | Class II |
| 3697 | 3711 | ARTVYDDGARRVWTL | 105.23 | -0.6118 |  | No | Class II |
| 3705 | 3719 | ARRVWTLMNVLTLVY | 731 | 0.2317 |  | No | Class II |
| 3706 | 3720 | RRVWTLMNVLTLVYK | 304.27 | 0.3142 |  | No | Class II |
| 3710 | 3724 | TLMNVLTLVYKVYYG | 300.03 | 0.4435 |  | No | Class II |
| 3711 | 3725 | LMNVLTLVYKVYYGN | 112.35 | 0.4219 |  | No | Class II |
| 3716 | 3730 | TLVYKVYYGNALDQA | 48.48 | 0.3278 |  | No | Class II |
| 3719 | 3733 | YKVYYGNALDQAISM | 304.37 | 0.3876 |  | No | Class II |
| 3722 | 3736 | YYGNALDQAISMWAL | 520.93 | 0.1976 |  | No | Class II |
| 3727 | 3741 | LDQAISMWALIISVT | 320.94 | 0.4647 |  | No | Class II |
| 3728 | 3742 | DQAISMWALIISVTS | 213.8 | 0.536 |  | No | Class II |
| 3732 | 3746 | SMWALIISVTSNYSG | 677.71 | 0.5056 |  | No | Class II |
| 3744 | 3758 | YSGVVTTVMFLARGI | 691.63 | 0.6505 |  | No | Class II |
| 3747 | 3761 | VVTTVMFLARGIVFM | 176.41 | 0.7068 |  | No | Class II |
| 3749 | 3763 | TTVMFLARGIVFMCV | 825.41 | 0.7332 |  | No | Class II |
| 3752 | 3766 | MFLARGIVFMCVEYC | 873.71 | 0.6653 |  | No | Class II |
| 3757 | 3771 | GIVFMCVEYCPIFFI | 693.17 | 0.9234 |  | No | Class II |
| 3762 | 3776 | CVEYCPIFFITGNTL | 270.36 | 0.6122 |  | No | Class II |
| 3767 | 3781 | PIFFITGNTLQCIML | 58.05 | 0.6769 |  | No | Class II |
| 3768 | 3782 | IFFITGNTLQCIMLV | 224.06 | 0.6756 |  | No | Class II |
| 3772 | 3786 | TGNTLQCIMLVYCFL | 692.78 | 0.6538 |  | No | Class II |
| 3780 | 3794 | MLVYCFLGYFCTCYF | 977.59 | -0.2792 |  | No | Class II |
| 3783 | 3797 | YCFLGYFCTCYFGLF | 751.9 | -0.2442 |  | No | Class II |
| 3785 | 3799 | FLGYFCTCYFGLFCL | 837.22 | 0.192 |  | No | Class II |
| 3789 | 3803 | FCTCYFGLFCLLNRY | 564.89 | 0.18 |  | No | Class II |
| 3790 | 3804 | CTCYFGLFCLLNRYF | 952.9 | 0.3139 |  | No | Class II |
| 3794 | 3808 | FGLFCLLNRYFRLTL | 201.7 | 1.1133 |  | No | Class II |
| 3799 | 3813 | LLNRYFRLTLGVYDY | 278.32 | 0.7157 |  | No | Class II |
| 3801 | 3815 | NRYFRLTLGVYDYLV | 60.21 | 0.5492 |  | No | Class II |
| 3804 | 3818 | FRLTLGVYDYLVSTQ | 197.32 | 0.864 |  | No | Class II |
| 3808 | 3822 | LGVYDYLVSTQEFRY | 52.73 | 0.8435 |  | No | Class II |
| 3809 | 3823 | GVYDYLVSTQEFRYM | 375.19 | 0.6234 |  | No | Class II |
| 3817 | 3831 | TQEFRYMNSQGLLPP | 731.68 | 0.5071 |  | No | Class II |
| 3821 | 3835 | RYMNSQGLLPPKNSI | 242.31 | 0.3972 |  | No | Class II |
| 3828 | 3842 | LLPPKNSIDAFKLNI | 516.35 | 0.1943 |  | No | Class II |
| 3833 | 3847 | NSIDAFKLNIKLLGV | 109.98 | 0.71 |  | No | Class II |
| 3838 | 3852 | FKLNIKLLGVGGKPC | 110.45 | 1.431 |  | No | Class II |
| 3842 | 3856 | IKLLGVGGKPCIKVA | 771.14 | 1.3022 |  | No | Class II |
| 3843 | 3857 | KLLGVGGKPCIKVAT | 782.17 | 1.0647 |  | No | Class II |
| 3847 | 3861 | VGGKPCIKVATVQSK | 977.52 | 1.1125 |  | No | Class II |
| 3849 | 3863 | GKPCIKVATVQSKMS | 159.78 | 1.1089 |  | No | Class II |
| 3852 | 3866 | CIKVATVQSKMSDVK | 649.36 | 1.1051 |  | No | Class II |
| 3854 | 3868 | KVATVQSKMSDVKCT | 328.65 | 1.1441 |  | No | Class II |
| 3861 | 3875 | KMSDVKCTSVVLLSV | 159.51 | 1.1708 |  | No | Class II |
| 3863 | 3877 | SDVKCTSVVLLSVLQ | 288.23 | 1.0228 |  | No | Class II |
| 3870 | 3884 | VVLLSVLQQLRVESS | 86.63 | 0.5017 |  | No | Class II |
| 3873 | 3887 | LSVLQQLRVESSSKL | 616.82 | 0.539 |  | No | Class II |
| 3876 | 3890 | LQQLRVESSSKLWAQ | 358.25 | 0.0522 |  | No | Class II |
| 3879 | 3893 | LRVESSSKLWAQCVQ | 306.69 | 0.2301 |  | No | Class II |
| 3882 | 3896 | ESSSKLWAQCVQLHN | 116.79 | 0.2419 |  | No | Class II |
| 3887 | 3901 | LWAQCVQLHNDILLA | 268.16 | 0.2169 |  | No | Class II |
| 3889 | 3903 | AQCVQLHNDILLAKD | 587.69 | 0.5525 |  | No | Class II |
| 3892 | 3906 | VQLHNDILLAKDTTE | 88.26 | 0.3502 |  | No | Class II |
| 3897 | 3911 | DILLAKDTTEAFEKM | 731.68 | 0.2299 |  | No | Class II |
| 3898 | 3912 | ILLAKDTTEAFEKMV | 52.66 | 0.0132 |  | No | Class II |
| 3903 | 3917 | DTTEAFEKMVSLLSV | 30.11 | 0.2305 |  | No | Class II |
| 3904 | 3918 | TTEAFEKMVSLLSVL | 720.53 | 0.1162 |  | No | Class II |
| 3908 | 3922 | FEKMVSLLSVLLSMQ | 457.01 | 0.4583 |  | No | Class II |
| 3910 | 3924 | KMVSLLSVLLSMQGA | 531.84 | 0.6672 |  | No | Class II |
| 3916 | 3930 | SVLLSMQGAVDINKL | 656.73 | 0.3695 |  | No | Class II |
| 3922 | 3936 | QGAVDINKLCEEMLD | 918.86 | 0.1213 |  | No | Class II |
| 3927 | 3941 | INKLCEEMLDNRATL | 839.48 | 0.0167 |  | No | Class II |
| 3932 | 3946 | EEMLDNRATLQAIAS | 200.12 | 0.384 |  | No | Class II |
| 3934 | 3948 | MLDNRATLQAIASEF | 57.26 | 0.5552 |  | No | Class II |
| 3939 | 3953 | ATLQAIASEFSSLPS | 582.87 | 0.3947 |  | No | Class II |
| 3946 | 3960 | SEFSSLPSYAAFATA | 139.38 | 0.5401 |  | No | Class II |
| 3951 | 3965 | LPSYAAFATAQEAYE | 37.64 | 0.3884 |  | No | Class II |
| 3955 | 3969 | AAFATAQEAYEQAVA | 445.54 | 0.3527 |  | No | Class II |
| 3956 | 3970 | AFATAQEAYEQAVAN | 449.84 | 0.2348 |  | No | Class II |
| 3961 | 3975 | QEAYEQAVANGDSEV | 505.85 | 0.0623 |  | No | Class II |
| 3964 | 3978 | YEQAVANGDSEVVLK | 234.47 | 0.2349 |  | No | Class II |
| 3969 | 3983 | ANGDSEVVLKKLKKS | 523.6 | 0.0703 |  | No | Class II |
| 3974 | 3988 | EVVLKKLKKSLNVAK | 18.07 | 0.2556 |  | No | Class II |
| 3979 | 3993 | KLKKSLNVAKSEFDR | 102.32 | -0.1709 |  | No | Class II |
| 3987 | 4001 | AKSEFDRDAAMQRKL | 64.2 | -0.1242 |  | No | Class II |
| 3993 | 4007 | RDAAMQRKLEKMADQ | 771.42 | 0.2077 |  | No | Class II |
| 3998 | 4012 | QRKLEKMADQAMTQM | 335.39 | 0.209 |  | No | Class II |
| 4004 | 4018 | MADQAMTQMYKQARS | 336.39 | 0.2678 |  | No | Class II |
| 4005 | 4019 | ADQAMTQMYKQARSE | 470.4 | 0.2207 |  | No | Class II |
| 4010 | 4024 | TQMYKQARSEDKRAK | 709.07 | 0.2643 |  | No | Class II |
| 4017 | 4031 | RSEDKRAKVTSAMQT | 527.32 | 0.4517 |  | No | Class II |
| 4022 | 4036 | RAKVTSAMQTMLFTM | 548.52 | 0.378 |  | No | Class II |
| 4027 | 4041 | SAMQTMLFTMLRKLD | 472.77 | 0.2366 |  | No | Class II |
| 4031 | 4045 | TMLFTMLRKLDNDAL | 9.63 | 0.0688 |  | No | Class II |
| 4036 | 4050 | MLRKLDNDALNNIIN | 388.69 | -0.3354 |  | No | Class II |
| 4043 | 4057 | DALNNIINNARDGCV | 459.13 | 0.1836 |  | No | Class II |
| 4048 | 4062 | IINNARDGCVPLNII | 594.18 | 0.8579 |  | No | Class II |
| 4050 | 4064 | NNARDGCVPLNIIPL | 697.43 | 1.2956 |  | No | Class II |
| 4055 | 4069 | GCVPLNIIPLTTAAK | 80.28 | 1.1262 |  | No | Class II |
| 4057 | 4071 | VPLNIIPLTTAAKLM | 649 | 0.884 |  | No | Class II |
| 4069 | 4083 | KLMVVIPDYNTYKNT | 89.3 | 0.0444 |  | No | Class II |
| 4074 | 4088 | IPDYNTYKNTCDGTT | 714.19 | 0.1606 |  | No | Class II |
| 4086 | 4100 | GTTFTYASALWEIQQ | 50.79 | 0.4963 |  | No | Class II |
| 4087 | 4101 | TTFTYASALWEIQQV | 195.43 | 0.6981 |  | No | Class II |
| 4092 | 4106 | ASALWEIQQVVDADS | 315.63 | 0.3081 |  | No | Class II |
| 4093 | 4107 | SALWEIQQVVDADSK | 122.01 | 0.4981 |  | No | Class II |
| 4105 | 4119 | DSKIVQLSEISMDNS | 281.61 | 0.8519 |  | No | Class II |
| 4106 | 4120 | SKIVQLSEISMDNSP | 868.41 | 0.7569 |  | No | Class II |
| 4110 | 4124 | QLSEISMDNSPNLAW | 91.88 | 1.03 |  | No | Class II |
| 4115 | 4129 | SMDNSPNLAWPLIVT | 358.61 | 0.8476 |  | No | Class II |
| 4117 | 4131 | DNSPNLAWPLIVTAL | 147.33 | 0.7662 |  | No | Class II |
| 4121 | 4135 | NLAWPLIVTALRANS | 610.5 | 0.9918 |  | No | Class II |
| 4125 | 4139 | PLIVTALRANSAVKL | 174.15 | 0.6555 |  | No | Class II |
| 4131 | 4145 | LRANSAVKLQNNELS | 72.42 | 0.7839 |  | No | Class II |
| 4139 | 4153 | LQNNELSPVALRQMS | 394.72 | 1.2572 |  | No | Class II |
| 4145 | 4159 | SPVALRQMSCAAGTT | 317.34 | 0.8871 |  | No | Class II |
| 4146 | 4160 | PVALRQMSCAAGTTQ | 406.32 | 0.8616 |  | No | Class II |
| 4151 | 4165 | QMSCAAGTTQTACTD | 71.49 | 0.8195 |  | No | Class II |
| 4160 | 4174 | QTACTDDNALAYYNT | 704.17 | 0.345 |  | No | Class II |
| 4169 | 4183 | LAYYNTTKGGRFVLA | 310.16 | 0.5921 |  | No | Class II |
| 4174 | 4188 | TTKGGRFVLALLSDL | 334.83 | 0.4321 |  | No | Class II |
| 4178 | 4192 | GRFVLALLSDLQDLK | 155.4 | 0.5578 |  | No | Class II |
| 4179 | 4193 | RFVLALLSDLQDLKW | 192.22 | 0.9095 |  | No | Class II |
| 4183 | 4197 | ALLSDLQDLKWARFP | 133.83 | 1.0755 |  | No | Class II |
| 4188 | 4202 | LQDLKWARFPKSDGT | 387.81 | 0.7545 |  | No | Class II |
| 4198 | 4212 | KSDGTGTIYTELEPP | 378.16 | 0.5309 |  | No | Class II |
| 4202 | 4216 | TGTIYTELEPPCRFV | 995.55 | 0.7912 |  | No | Class II |
| 4212 | 4226 | PCRFVTDTPKGPKVK | 809.88 | -0.1739 |  | No | Class II |
| 4220 | 4234 | PKGPKVKYLYFIKGL | 739.91 | 0.2838 |  | No | Class II |
| 4234 | 4248 | LNNLNRGMVLGSLAA | 67.66 | 0.8264 |  | No | Class II |
| 4244 | 4258 | GSLAATVRLQAGNAT | 119.83 | 0.9384 |  | No | Class II |
| 4249 | 4263 | TVRLQAGNATEVPAN | 50.05 | 0.4783 |  | No | Class II |
| 4254 | 4268 | AGNATEVPANSTVLS | 766.4 | 0.3184 |  | No | Class II |
| 4258 | 4272 | TEVPANSTVLSFCAF | 685.97 | 0.1735 |  | No | Class II |
| 4259 | 4273 | EVPANSTVLSFCAFA | 167.45 | 0.1931 |  | No | Class II |
| 4263 | 4277 | NSTVLSFCAFAVDAA | 23.67 | 0.3662 |  | No | Class II |
| 4264 | 4278 | STVLSFCAFAVDAAK | 179.23 | 0.2726 |  | No | Class II |
| 4269 | 4283 | FCAFAVDAAKAYKDY | 84.03 | 0.2604 |  | No | Class II |
| 4272 | 4286 | FAVDAAKAYKDYLAS | 152.83 | 0.0584 |  | No | Class II |
| 4277 | 4291 | AKAYKDYLASGGQPI | 481.33 | 0.0674 |  | No | Class II |
| 4278 | 4292 | KAYKDYLASGGQPIT | 16.8 | 0.0491 |  | No | Class II |
| 4282 | 4296 | DYLASGGQPITNCVK | 36.57 | 0.3112 |  | No | Class II |
| 4283 | 4297 | YLASGGQPITNCVKM | 286.7 | 0.3417 |  | No | Class II |
| 4291 | 4305 | ITNCVKMLCTHTGTG | 265.86 | 0.4285 |  | No | Class II |
| 4296 | 4310 | KMLCTHTGTGQAITV | 847.79 | 0.6311 |  | No | Class II |
| 4297 | 4311 | MLCTHTGTGQAITVT | 84.95 | 0.8046 |  | No | Class II |
| 4302 | 4316 | TGTGQAITVTPEANM | 251.91 | 0.7318 |  | No | Class II |
| 4307 | 4321 | AITVTPEANMDQESF | 854.03 | 0.5677 |  | No | Class II |
| 4317 | 4331 | DQESFGGASCCLYCR | 244.05 | 0.6538 |  | No | Class II |
| 4322 | 4336 | GGASCCLYCRCHIDH | 712.65 | 0.2683 |  | No | Class II |
| 4328 | 4342 | LYCRCHIDHPNPKGF | 932.62 | 0.2135 |  | No | Class II |
| 4338 | 4352 | NPKGFCDLKGKYVQI | 312.29 | 1.5023 |  | No | Class II |
| 4349 | 4363 | YVQIPTTCANDPVGF | 716 | 0.2689 |  | No | Class II |
| 4358 | 4372 | NDPVGFTLKNTVCTV | 325.89 | 1.0966 |  | No | Class II |
| 4363 | 4377 | FTLKNTVCTVCGMWK | 882.03 | 0.6757 |  | No | Class II |
| 4386 | 4400 | LREPMLQSADAQSFL | 336.59 | -0.0995 |  | No | Class II |
| 4388 | 4402 | EPMLQSADAQSFLNR | 146.12 | 0.0524 |  | No | Class II |
| 4391 | 4405 | LQSADAQSFLNRVCG | 756.8 | -0.2055 |  | No | Class II |
| 4393 | 4407 | SADAQSFLNRVCGVS | 812.85 | -0.0535 |  | No | Class II |
| 4398 | 4412 | SFLNRVCGVSAARLT | 182.39 | 0.6189 |  | No | Class II |
| 4403 | 4417 | VCGVSAARLTPCGTG | 168.41 | 0.6789 |  | No | Class II |
| 4410 | 4424 | RLTPCGTGTSTDVVY | 750.24 | 0.8066 |  | No | Class II |
| 4416 | 4430 | TGTSTDVVYRAFDIY | 134.88 | 0.7105 |  | No | Class II |
| 4421 | 4435 | DVVYRAFDIYNDKVA | 215.75 | 0.5186 |  | No | Class II |
| 4426 | 4440 | AFDIYNDKVAGFAKF | 992.49 | 0.471 |  | No | Class II |
| 4431 | 4445 | NDKVAGFAKFLKTNC | 903.4 | 0.1638 |  | No | Class II |
| 4437 | 4451 | FAKFLKTNCCRFQEK | 67.36 | -0.0672 |  | No | Class II |
| 4453 | 4467 | EDDNLIDSYFVVKRH | 111.5 | 0.4118 |  | No | Class II |
| 4458 | 4472 | IDSYFVVKRHTFSNY | 36.01 | 0.2947 |  | No | Class II |
| 4463 | 4477 | VVKRHTFSNYQHEET | 528.11 | 0.3823 |  | No | Class II |
| 4469 | 4483 | FSNYQHEETIYNLLK | 79.82 | 0.0664 |  | No | Class II |
| 4476 | 4490 | ETIYNLLKDCPAVAK | 53.36 | -0.1428 |  | No | Class II |
| 4488 | 4502 | VAKHDFFKFRIDGDM | 173.16 | -0.6673 |  | No | Class II |
| 4492 | 4506 | DFFKFRIDGDMVPHI | 415.5 | -0.2329 |  | No | Class II |
| 4493 | 4507 | FFKFRIDGDMVPHIS | 27.7 | -0.0276 |  | No | Class II |
| 4500 | 4514 | GDMVPHISRQRLTKY | 28.87 | -0.0627 |  | No | Class II |
| 4506 | 4520 | ISRQRLTKYTMADLV | 121.88 | 0.1738 |  | No | Class II |
| 4509 | 4523 | QRLTKYTMADLVYAL | 295.51 | 0.5025 |  | No | Class II |
| 4514 | 4528 | YTMADLVYALRHFDE | 713.22 | 0.7625 |  | No | Class II |
| 4516 | 4530 | MADLVYALRHFDEGN | 310.49 | 0.9657 |  | No | Class II |
| 4529 | 4543 | GNCDTLKEILVTYNC | 255.28 | -0.234 |  | No | Class II |
| 4534 | 4548 | LKEILVTYNCCDDDY | 218.54 | -0.1509 |  | No | Class II |
| 4549 | 4563 | FNKKDWYDFVENPDI | 395.41 | 0.9056 |  | No | Class II |
| 4551 | 4565 | KKDWYDFVENPDILR | 496.58 | 0.1286 |  | No | Class II |
| 4554 | 4568 | WYDFVENPDILRVYA | 264.13 | -0.2253 |  | No | Class II |
| 4561 | 4575 | PDILRVYANLGERVR | 975.58 | 0.4242 |  | No | Class II |
| 4566 | 4580 | VYANLGERVRQALLK | 96.77 | 0.3733 |  | No | Class II |
| 4568 | 4582 | ANLGERVRQALLKTV | 214.13 | 0.4854 |  | No | Class II |
| 4571 | 4585 | GERVRQALLKTVQFC | 669.56 | 0.3857 |  | No | Class II |
| 4573 | 4587 | RVRQALLKTVQFCDA | 207.26 | 0.3271 |  | No | Class II |
| 4580 | 4594 | KTVQFCDAMRNAGIV | 168.96 | 0.8207 |  | No | Class II |
| 4585 | 4599 | CDAMRNAGIVGVLTL | 290.2 | 0.5806 |  | No | Class II |
| 4586 | 4600 | DAMRNAGIVGVLTLD | 106.43 | 0.6743 |  | No | Class II |
| 4590 | 4604 | NAGIVGVLTLDNQDL | 95.63 | 1.2975 |  | No | Class II |
| 4591 | 4605 | AGIVGVLTLDNQDLN | 668.57 | 1.2778 |  | No | Class II |
| 4595 | 4609 | GVLTLDNQDLNGNWY | 853.41 | 0.8531 |  | No | Class II |
| 4603 | 4617 | DLNGNWYDFGDFIQT | 309.93 | 0.6768 |  | No | Class II |
| 4611 | 4625 | FGDFIQTTPGSGVPV | 99.98 | 0.2604 |  | No | Class II |
| 4616 | 4630 | QTTPGSGVPVVDSYY | 68.57 | 0.4113 |  | No | Class II |
| 4622 | 4636 | GVPVVDSYYSLLMPI | 97.28 | 0.6336 |  | No | Class II |
| 4625 | 4639 | VVDSYYSLLMPILTL | 407.97 | 0.4474 |  | No | Class II |
| 4631 | 4645 | SLLMPILTLTRALTA | 412.13 | 0.2378 |  | No | Class II |
| 4634 | 4648 | MPILTLTRALTAESH | 75.05 | 0.3258 |  | No | Class II |
| 4636 | 4650 | ILTLTRALTAESHVD | 99.65 | 0.1884 |  | No | Class II |
| 4639 | 4653 | LTRALTAESHVDTDL | 238.2 | -0.1664 |  | No | Class II |
| 4646 | 4660 | ESHVDTDLTKPYIKW | 184.35 | -0.0794 |  | No | Class II |
| 4654 | 4668 | TKPYIKWDLLKYDFT | 528.42 | 0.2061 |  | No | Class II |
| 4658 | 4672 | IKWDLLKYDFTEERL | 980.33 | 0.2361 |  | No | Class II |
| 4668 | 4682 | TEERLKLFDRYFKYW | 55.74 | 0.175 |  | No | Class II |
| 4673 | 4687 | KLFDRYFKYWDQTYH | 198.19 | 0.0064 |  | No | Class II |
| 4674 | 4688 | LFDRYFKYWDQTYHP | 463.95 | 0.1627 |  | No | Class II |
| 4678 | 4692 | YFKYWDQTYHPNCVN | 664.06 | 0.7978 |  | No | Class II |
| 4684 | 4698 | QTYHPNCVNCLDDRC | 422.14 | 0.8278 |  | No | Class II |
| 4696 | 4710 | DRCILHCANFNVLFS | 893.43 | 0.6166 |  | No | Class II |
| 4704 | 4718 | NFNVLFSTVFPPTSF | 398.17 | 0.5021 |  | No | Class II |
| 4713 | 4727 | FPPTSFGPLVRKIFV | 696.31 | 0.7364 |  | No | Class II |
| 4722 | 4736 | VRKIFVDGVPFVVST | 46.81 | -0.1419 |  | No | Class II |
| 4723 | 4737 | RKIFVDGVPFVVSTG | 72.8 | 0.0122 |  | No | Class II |
| 4732 | 4746 | FVVSTGYHFRELGVV | 90.54 | 1.4428 |  | No | Class II |
| 4743 | 4757 | LGVVHNQDVNLHSSR | 74.96 | 0.9604 |  | No | Class II |
| 4745 | 4759 | VVHNQDVNLHSSRLS | 233.88 | 0.6467 |  | No | Class II |
| 4749 | 4763 | QDVNLHSSRLSFKEL | 62.28 | 1.0307 |  | No | Class II |
| 4754 | 4768 | HSSRLSFKELLVYAA | 899.15 | 0.5448 |  | No | Class II |
| 4760 | 4774 | FKELLVYAADPAMHA | 86.73 | 0.0669 |  | No | Class II |
| 4765 | 4779 | VYAADPAMHAASGNL | 109.09 | 0.435 |  | No | Class II |
| 4769 | 4783 | DPAMHAASGNLLLDK | 170.08 | 0.111 |  | No | Class II |
| 4776 | 4790 | SGNLLLDKRTTCFSV | 68.62 | 1.0422 |  | No | Class II |
| 4784 | 4798 | RTTCFSVAALTNNVA | 158.04 | 1.0616 |  | No | Class II |
| 4789 | 4803 | SVAALTNNVAFQTVK | 604.21 | 0.6108 |  | No | Class II |
| 4795 | 4809 | NNVAFQTVKPGNFNK | 71.1 | 1.0318 |  | No | Class II |
| 4804 | 4818 | PGNFNKDFYDFAVSK | 167.54 | 0.4083 |  | No | Class II |
| 4808 | 4822 | NKDFYDFAVSKGFFK | 41.58 | 0.3145 |  | No | Class II |
| 4809 | 4823 | KDFYDFAVSKGFFKE | 681.5 | 0.1516 |  | No | Class II |
| 4813 | 4827 | DFAVSKGFFKEGSSV | 367.51 | 0.2035 |  | No | Class II |
| 4818 | 4832 | KGFFKEGSSVELKHF | 338.08 | 0.4146 |  | No | Class II |
| 4823 | 4837 | EGSSVELKHFFFAQD | 731.37 | 0.8813 |  | No | Class II |
| 4827 | 4841 | VELKHFFFAQDGNAA | 499.41 | 0.8875 |  | No | Class II |
| 4838 | 4852 | GNAAISDYDYYRYNL | 977.14 | 0.6247 |  | No | Class II |
| 4845 | 4859 | YDYYRYNLPTMCDIR | 170.04 | 0.3619 |  | No | Class II |
| 4850 | 4864 | YNLPTMCDIRQLLFV | 527.96 | 0.3694 |  | No | Class II |
| 4857 | 4871 | DIRQLLFVVEVVDKY | 223.91 | 0.7148 |  | No | Class II |
| 4858 | 4872 | IRQLLFVVEVVDKYF | 618.61 | 0.4575 |  | No | Class II |
| 4863 | 4877 | FVVEVVDKYFDCYDG | 993.69 | 0.5505 |  | No | Class II |
| 4868 | 4882 | VDKYFDCYDGGCINA | 908.37 | 0.3937 |  | No | Class II |
| 4872 | 4886 | FDCYDGGCINANQVI | 138.03 | 0.8898 |  | No | Class II |
| 4876 | 4890 | DGGCINANQVIVNNL | 104.66 | 0.259 |  | No | Class II |
| 4878 | 4892 | GCINANQVIVNNLDK | 904.29 | 0.104 |  | No | Class II |
| 4882 | 4896 | ANQVIVNNLDKSAGF | 83.94 | 0.2146 |  | No | Class II |
| 4887 | 4901 | VNNLDKSAGFPFNKW | 77.83 | 0.3548 |  | No | Class II |
| 4894 | 4908 | AGFPFNKWGKARLYY | 112.59 | -0.2455 |  | No | Class II |
| 4896 | 4910 | FPFNKWGKARLYYDS | 432.63 | -0.6118 |  | No | Class II |
| 4901 | 4915 | WGKARLYYDSMSYED | 267.47 | 0.2217 |  | No | Class II |
| 4902 | 4916 | GKARLYYDSMSYEDQ | 684.11 | 0.3624 |  | No | Class II |
| 4907 | 4921 | YYDSMSYEDQDALFA | 237.51 | 0.7563 |  | No | Class II |
| 4910 | 4924 | SMSYEDQDALFAYTK | 286.3 | 1.0799 |  | No | Class II |
| 4918 | 4932 | ALFAYTKRNVIPTIT | 107.29 | 0.9452 |  | No | Class II |
| 4923 | 4937 | TKRNVIPTITQMNLK | 976.91 | 1.1965 |  | No | Class II |
| 4924 | 4938 | KRNVIPTITQMNLKY | 66.23 | 1.2492 |  | No | Class II |
| 4928 | 4942 | IPTITQMNLKYAISA | 536.15 | 0.9082 |  | No | Class II |
| 4933 | 4947 | QMNLKYAISAKNRAR | 183.17 | 1.5044 |  | No | Class II |
| 4940 | 4954 | ISAKNRARTVAGVSI | 420.34 | 1.3447 |  | No | Class II |
| 4943 | 4957 | KNRARTVAGVSICST | 193.86 | 0.5688 |  | No | Class II |
| 4945 | 4959 | RARTVAGVSICSTMT | 73.74 | 0.6193 |  | No | Class II |
| 4950 | 4964 | AGVSICSTMTNRQFH | 75.15 | 0.9179 |  | No | Class II |
| 4957 | 4971 | TMTNRQFHQKLLKSI | 651.99 | 0.0096 |  | No | Class II |
| 4961 | 4975 | RQFHQKLLKSIAATR | 792.61 | -0.1856 |  | No | Class II |
| 4962 | 4976 | QFHQKLLKSIAATRG | 129.03 | 0.1975 |  | No | Class II |
| 4966 | 4980 | KLLKSIAATRGATVV | 137.32 | 0.1825 |  | No | Class II |
| 4967 | 4981 | LLKSIAATRGATVVI | 7.31 | 0.3182 |  | No | Class II |
| 4972 | 4986 | AATRGATVVIGTSKF | 972.29 | 0.4846 |  | No | Class II |
| 4976 | 4990 | GATVVIGTSKFYGGW | 585.71 | 0.0972 |  | No | Class II |
| 4977 | 4991 | ATVVIGTSKFYGGWH | 195.9 | 0.1629 |  | No | Class II |
| 4985 | 4999 | KFYGGWHNMLKTVYS | 141.29 | -0.5162 |  | No | Class II |
| 4991 | 5005 | HNMLKTVYSDVENPH | 402.11 | -0.0627 |  | No | Class II |
| 4993 | 5007 | MLKTVYSDVENPHLM | 306.22 | 0.2057 |  | No | Class II |
| 5009 | 5023 | WDYPKCDRAMPNMLR | 217.96 | -0.569 |  | No | Class II |
| 5014 | 5028 | CDRAMPNMLRIMASL | 151.75 | -0.1763 |  | No | Class II |
| 5015 | 5029 | DRAMPNMLRIMASLV | 164.83 | -0.0333 |  | No | Class II |
| 5020 | 5034 | NMLRIMASLVLARKH | 127.05 | 0.4897 |  | No | Class II |
| 5024 | 5038 | IMASLVLARKHTTCC | 72.26 | 1.3386 |  | No | Class II |
| 5029 | 5043 | VLARKHTTCCSLSHR | 982.1 | 1.7683 |  | No | Class II |
| 5034 | 5048 | HTTCCSLSHRFYRLA | 406.7 | 0.9613 |  | No | Class II |
| 5041 | 5055 | SHRFYRLANECAQVL | 15.3 | 0.1719 |  | No | Class II |
| 5045 | 5059 | YRLANECAQVLSEMV | 335.48 | 0.1886 |  | No | Class II |
| 5046 | 5060 | RLANECAQVLSEMVM | 140.35 | 0.2301 |  | No | Class II |
| 5054 | 5068 | VLSEMVMCGGSLYVK | 259.23 | 0.111 |  | No | Class II |
| 5056 | 5070 | SEMVMCGGSLYVKPG | 239.93 | 0.5354 |  | No | Class II |
| 5064 | 5078 | SLYVKPGGTSSGDAT | 157.97 | 0.8247 |  | No | Class II |
| 5069 | 5083 | PGGTSSGDATTAYAN | 85.59 | 0.335 |  | No | Class II |
| 5074 | 5088 | SGDATTAYANSVFNI | 104.52 | 0.331 |  | No | Class II |
| 5078 | 5092 | TTAYANSVFNICQAV | 197.42 | -0.0101 |  | No | Class II |
| 5080 | 5094 | AYANSVFNICQAVTA | 258.54 | 0.0231 |  | No | Class II |
| 5083 | 5097 | NSVFNICQAVTANVN | 76.03 | 0.008 |  | No | Class II |
| 5085 | 5099 | VFNICQAVTANVNAL | 172.55 | -0.0507 |  | No | Class II |
| 5088 | 5102 | ICQAVTANVNALLST | 12.99 | 0.1861 |  | No | Class II |
| 5090 | 5104 | QAVTANVNALLSTDG | 314.94 | 0.0387 |  | No | Class II |
| 5094 | 5108 | ANVNALLSTDGNKIA | 20.64 | -0.0985 |  | No | Class II |
| 5099 | 5113 | LLSTDGNKIADKYVR | 545 | -0.1514 |  | No | Class II |
| 5105 | 5119 | NKIADKYVRNLQHRL | 382.89 | -0.161 |  | No | Class II |
| 5110 | 5124 | KYVRNLQHRLYECLY | 77.7 | -0.3626 |  | No | Class II |
| 5115 | 5129 | LQHRLYECLYRNRDV | 323.51 | 0.5161 |  | No | Class II |
| 5120 | 5134 | YECLYRNRDVDTDFV | 295 | 1.0436 |  | No | Class II |
| 5125 | 5139 | RNRDVDTDFVNEFYA | 705.6 | 0.6566 |  | No | Class II |
| 5126 | 5140 | NRDVDTDFVNEFYAY | 121.01 | 0.1938 |  | No | Class II |
| 5130 | 5144 | DTDFVNEFYAYLRKH | 45.58 | 0.1363 |  | No | Class II |
| 5131 | 5145 | TDFVNEFYAYLRKHF | 716.45 | 0.0036 |  | No | Class II |
| 5135 | 5149 | NEFYAYLRKHFSMMI | 11.19 | 0.2279 |  | No | Class II |
| 5137 | 5151 | FYAYLRKHFSMMILS | 702.84 | 0.4202 |  | No | Class II |
| 5142 | 5156 | RKHFSMMILSDDAVV | 253.54 | 0.3798 |  | No | Class II |
| 5147 | 5161 | MMILSDDAVVCFNST | 964.54 | 0.5592 |  | No | Class II |
| 5155 | 5169 | VVCFNSTYASQGLVA | 290.4 | 1.1113 |  | No | Class II |
| 5165 | 5179 | QGLVASIKNFKSVLY | 15.31 | 0.369 |  | No | Class II |
| 5169 | 5183 | ASIKNFKSVLYYQNN | 197.8 | 0.509 |  | No | Class II |
| 5174 | 5188 | FKSVLYYQNNVFMSE | 185.3 | 0.3246 |  | No | Class II |
| 5179 | 5193 | YYQNNVFMSEAKCWT | 819.58 | 0.4219 |  | No | Class II |
| 5200 | 5214 | GPHEFCSQHTMLVKQ | 58.87 | 0.3519 |  | No | Class II |
| 5203 | 5217 | EFCSQHTMLVKQGDD | 497.18 | 0.5127 |  | No | Class II |
| 5207 | 5221 | QHTMLVKQGDDYVYL | 859.52 | 0.4187 |  | No | Class II |
| 5216 | 5230 | DDYVYLPYPDPSRIL | 460.01 | -0.205 |  | No | Class II |
| 5221 | 5235 | LPYPDPSRILGAGCF | 486.85 | 0.1249 |  | No | Class II |
| 5226 | 5240 | PSRILGAGCFVDDIV | 370.2 | -0.0597 |  | No | Class II |
| 5231 | 5245 | GAGCFVDDIVKTDGT | 810 | -0.1079 |  | No | Class II |
| 5237 | 5251 | DDIVKTDGTLMIERF | 331.02 | -0.4258 |  | No | Class II |
| 5238 | 5252 | DIVKTDGTLMIERFV | 35.33 | -0.3823 |  | No | Class II |
| 5247 | 5261 | MIERFVSLAIDAYPL | 94.57 | 0.2978 |  | No | Class II |
| 5252 | 5266 | VSLAIDAYPLTKHPN | 512.05 | 0.6283 |  | No | Class II |
| 5258 | 5272 | AYPLTKHPNQEYADV | 378.53 | 0.3985 |  | No | Class II |
| 5262 | 5276 | TKHPNQEYADVFHLY | 600.52 | 0.3377 |  | No | Class II |
| 5263 | 5277 | KHPNQEYADVFHLYL | 599.19 | 0.2885 |  | No | Class II |
| 5267 | 5281 | QEYADVFHLYLQYIR | 365.65 | 0.3913 |  | No | Class II |
| 5272 | 5286 | VFHLYLQYIRKLHDE | 957.95 | 0.2814 |  | No | Class II |
| 5273 | 5287 | FHLYLQYIRKLHDEL | 75.18 | 0.3059 |  | No | Class II |
| 5278 | 5292 | QYIRKLHDELTGHML | 221.67 | -0.0869 |  | No | Class II |
| 5284 | 5298 | HDELTGHMLDMYSVM | 907.2 | 0.3708 |  | No | Class II |
| 5286 | 5300 | ELTGHMLDMYSVMLT | 582.78 | 0.537 |  | No | Class II |
| 5289 | 5303 | GHMLDMYSVMLTNDN | 101 | 0.5082 |  | No | Class II |
| 5291 | 5305 | MLDMYSVMLTNDNTS | 782.2 | 0.5661 |  | No | Class II |
| 5294 | 5308 | MYSVMLTNDNTSRYW | 42.62 | 0.0531 |  | No | Class II |
| 5304 | 5318 | TSRYWEPEFYEAMYT | 712.35 | 0.3176 |  | No | Class II |
| 5306 | 5320 | RYWEPEFYEAMYTPH | 941.89 | 0.2406 |  | No | Class II |
| 5311 | 5325 | EFYEAMYTPHTVLQA | 15.67 | 0.1496 |  | No | Class II |
| 5314 | 5328 | EAMYTPHTVLQAVGA | 430.77 | 0.277 |  | No | Class II |
| 5317 | 5331 | YTPHTVLQAVGACVL | 58.2 | 0.2151 |  | No | Class II |
| 5322 | 5336 | VLQAVGACVLCNSQT | 162.21 | 0.1991 |  | No | Class II |
| 5325 | 5339 | AVGACVLCNSQTSLR | 512.31 | 0.45 |  | No | Class II |
| 5328 | 5342 | ACVLCNSQTSLRCGA | 550.59 | 0.8641 |  | No | Class II |
| 5330 | 5344 | VLCNSQTSLRCGACI | 912.33 | 0.724 |  | No | Class II |
| 5335 | 5349 | QTSLRCGACIRRPFL | 240.13 | 0.6037 |  | No | Class II |
| 5345 | 5359 | RRPFLCCKCCYDHVI | 943.45 | 0.4864 |  | No | Class II |
| 5350 | 5364 | CCKCCYDHVISTSHK | 896.15 | 0.3524 |  | No | Class II |
| 5355 | 5369 | YDHVISTSHKLVLSV | 68.47 | 0.4857 |  | No | Class II |
| 5357 | 5371 | HVISTSHKLVLSVNP | 334.05 | 0.6602 |  | No | Class II |
| 5362 | 5376 | SHKLVLSVNPYVCNA | 993.04 | 0.0593 |  | No | Class II |
| 5384 | 5398 | VTQLYLGGMSYYCKS | 168.38 | 0.6805 |  | No | Class II |
| 5385 | 5399 | TQLYLGGMSYYCKSH | 125.11 | 0.6045 |  | No | Class II |
| 5391 | 5405 | GMSYYCKSHKPPISF | 561.67 | 0.6235 |  | No | Class II |
| 5399 | 5413 | HKPPISFPLCANGQV | 609.37 | -0.2276 |  | No | Class II |
| 5404 | 5418 | SFPLCANGQVFGLYK | 558.79 | -0.412 |  | No | Class II |
| 5405 | 5419 | FPLCANGQVFGLYKN | 302.41 | -0.4073 |  | No | Class II |
| 5410 | 5424 | NGQVFGLYKNTCVGS | 103.03 | 0.1556 |  | No | Class II |
| 5415 | 5429 | GLYKNTCVGSDNVTD | 645.46 | 0.5272 |  | No | Class II |
| 5427 | 5441 | VTDFNAIATCDWTNA | 869.6 | 0.6958 |  | No | Class II |
| 5435 | 5449 | TCDWTNAGDYILANT | 838.34 | 0.4883 |  | No | Class II |
| 5441 | 5455 | AGDYILANTCTERLK | 35.51 | -0.1205 |  | No | Class II |
| 5443 | 5457 | DYILANTCTERLKLF | 875.32 | -0.2168 |  | No | Class II |
| 5446 | 5460 | LANTCTERLKLFAAE | 399.69 | -0.033 |  | No | Class II |
| 5448 | 5462 | NTCTERLKLFAAETL | 372.55 | -0.2091 |  | No | Class II |
| 5451 | 5465 | TERLKLFAAETLKAT | 163.7 | 0.2798 |  | No | Class II |
| 5453 | 5467 | RLKLFAAETLKATEE | 85.15 | 0.4049 |  | No | Class II |
| 5456 | 5470 | LFAAETLKATEETFK | 156.32 | 0.3372 |  | No | Class II |
| 5464 | 5478 | ATEETFKLSYGIATV | 505.28 | 0.6446 |  | No | Class II |
| 5469 | 5483 | FKLSYGIATVREVLS | 65.96 | 0.7783 |  | No | Class II |
| 5475 | 5489 | IATVREVLSDRELHL | 405.96 | 0.816 |  | No | Class II |
| 5484 | 5498 | DRELHLSWEVGKPRP | 522.42 | 1.1149 |  | No | Class II |
| 5488 | 5502 | HLSWEVGKPRPPLNR | 369.26 | -0.0942 |  | No | Class II |
| 5495 | 5509 | KPRPPLNRNYVFTGY | 243.43 | 0.1999 |  | No | Class II |
| 5509 | 5523 | YRVTKNSKVQIGEYT | 275.61 | 0.5667 |  | No | Class II |
| 5510 | 5524 | RVTKNSKVQIGEYTF | 288.24 | 0.675 |  | No | Class II |
| 5519 | 5533 | IGEYTFEKGDYGDAV | 781.05 | 0.8459 |  | No | Class II |
| 5525 | 5539 | EKGDYGDAVVYRGTT | 675 | 0.7783 |  | No | Class II |
| 5526 | 5540 | KGDYGDAVVYRGTTT | 188.03 | 0.6543 |  | No | Class II |
| 5531 | 5545 | DAVVYRGTTTYKLNV | 7.8 | 0.3576 |  | No | Class II |
| 5532 | 5546 | AVVYRGTTTYKLNVG | 429.01 | 0.7324 |  | No | Class II |
| 5540 | 5554 | TYKLNVGDYFVLTSH | 678.77 | 0.4726 |  | No | Class II |
| 5545 | 5559 | VGDYFVLTSHTVMPL | 23.18 | 0.3939 |  | No | Class II |
| 5549 | 5563 | FVLTSHTVMPLSAPT | 81.98 | 0.3449 |  | No | Class II |
| 5551 | 5565 | LTSHTVMPLSAPTLV | 310.76 | 0.237 |  | No | Class II |
| 5556 | 5570 | VMPLSAPTLVPQEHY | 709.73 | 0.4048 |  | No | Class II |
| 5560 | 5574 | SAPTLVPQEHYVRIT | 812.25 | 0.5056 |  | No | Class II |
| 5568 | 5582 | EHYVRITGLYPTLNI | 392.57 | 0.5456 |  | No | Class II |
| 5573 | 5587 | ITGLYPTLNISDEFS | 109.36 | 0.7408 |  | No | Class II |
| 5582 | 5596 | ISDEFSSNVANYQKV | 228.91 | 0.2427 |  | No | Class II |
| 5590 | 5604 | VANYQKVGMQKYSTL | 557.75 | 0.5956 |  | No | Class II |
| 5595 | 5609 | KVGMQKYSTLQGPPG | 116.5 | 0.2559 |  | No | Class II |
| 5600 | 5614 | KYSTLQGPPGTGKSH | 272.48 | 0.3994 |  | No | Class II |
| 5605 | 5619 | QGPPGTGKSHFAIGL | 69.4 | 1.0633 |  | No | Class II |
| 5610 | 5624 | TGKSHFAIGLALYYP | 239.58 | 0.9777 |  | No | Class II |
| 5615 | 5629 | FAIGLALYYPSARIV | 319.41 | 0.6023 |  | No | Class II |
| 5620 | 5634 | ALYYPSARIVYTACS | 236.7 | 0.1399 |  | No | Class II |
| 5621 | 5635 | LYYPSARIVYTACSH | 415.32 | 0.0895 |  | No | Class II |
| 5626 | 5640 | ARIVYTACSHAAVDA | 72.53 | 0.2735 |  | No | Class II |
| 5632 | 5646 | ACSHAAVDALCEKAL | 555.74 | 0.2231 |  | No | Class II |
| 5637 | 5651 | AVDALCEKALKYLPI | 700.09 | 0.8316 |  | No | Class II |
| 5643 | 5657 | EKALKYLPIDKCSRI | 627.98 | 0.0554 |  | No | Class II |
| 5648 | 5662 | YLPIDKCSRIIPARA | 290.13 | -0.3652 |  | No | Class II |
| 5650 | 5664 | PIDKCSRIIPARARV | 390 | -0.1729 |  | No | Class II |
| 5653 | 5667 | KCSRIIPARARVECF | 251.83 | 0.859 |  | No | Class II |
| 5655 | 5669 | SRIIPARARVECFDK | 327.3 | 0.8398 |  | No | Class II |
| 5661 | 5675 | RARVECFDKFKVNST | 203.21 | 0.9658 |  | No | Class II |
| 5667 | 5681 | FDKFKVNSTLEQYVF | 318.41 | -0.2549 |  | No | Class II |
| 5673 | 5687 | NSTLEQYVFCTVNAL | 250.41 | 0.2234 |  | No | Class II |
| 5676 | 5690 | LEQYVFCTVNALPET | 369.21 | 0.2994 |  | No | Class II |
| 5681 | 5695 | FCTVNALPETTADIV | 702.9 | 0.5664 |  | No | Class II |
| 5686 | 5700 | ALPETTADIVVFDEI | 259.07 | 0.1909 |  | No | Class II |
| 5691 | 5705 | TADIVVFDEISMATN | 524.68 | 0.3905 |  | No | Class II |
| 5692 | 5706 | ADIVVFDEISMATNY | 91.39 | 0.4771 |  | No | Class II |
| 5697 | 5711 | FDEISMATNYDLSVV | 338.49 | 1.0082 |  | No | Class II |
| 5702 | 5716 | MATNYDLSVVNARLR | 352.23 | 1.4026 |  | No | Class II |
| 5707 | 5721 | DLSVVNARLRAKHYV | 998.64 | 1.6866 |  | No | Class II |
| 5711 | 5725 | VNARLRAKHYVYIGD | 370.21 | 1.6287 |  | No | Class II |
| 5717 | 5731 | AKHYVYIGDPAQLPA | 378.28 | 0.4136 |  | No | Class II |
| 5727 | 5741 | AQLPAPRTLLTKGTL | 978.66 | -0.2714 |  | No | Class II |
| 5732 | 5746 | PRTLLTKGTLEPEYF | 853.97 | 0.4747 |  | No | Class II |
| 5738 | 5752 | KGTLEPEYFNSVCRL | 911.98 | 0.8582 |  | No | Class II |
| 5742 | 5756 | EPEYFNSVCRLMKTI | 472.53 | 0.2381 |  | No | Class II |
| 5743 | 5757 | PEYFNSVCRLMKTIG | 21.39 | 0.159 |  | No | Class II |
| 5748 | 5762 | SVCRLMKTIGPDMFL | 689.36 | -0.2941 |  | No | Class II |
| 5753 | 5767 | MKTIGPDMFLGTCRR | 615.77 | 0.3977 |  | No | Class II |
| 5757 | 5771 | GPDMFLGTCRRCPAE | 425.63 | -0.0621 |  | No | Class II |
| 5758 | 5772 | PDMFLGTCRRCPAEI | 142.29 | 0.1333 |  | No | Class II |
| 5767 | 5781 | RCPAEIVDTVSALVY | 59.29 | 0.2457 |  | No | Class II |
| 5771 | 5785 | EIVDTVSALVYDNKL | 265.16 | 0.5631 |  | No | Class II |
| 5775 | 5789 | TVSALVYDNKLKAHK | 19.91 | 0.0934 |  | No | Class II |
| 5787 | 5801 | AHKDKSAQCFKMFYK | 413.64 | 0.2517 |  | No | Class II |
| 5792 | 5806 | SAQCFKMFYKGVITH | 31.07 | 0.3749 |  | No | Class II |
| 5797 | 5811 | KMFYKGVITHDVSSA | 329.14 | -0.0028 |  | No | Class II |
| 5800 | 5814 | YKGVITHDVSSAINR | 61.65 | 0.0079 |  | No | Class II |
| 5805 | 5819 | THDVSSAINRPQIGV | 593.49 | 0.1828 |  | No | Class II |
| 5812 | 5826 | INRPQIGVVREFLTR | 250.96 | -0.8777 |  | No | Class II |
| 5814 | 5828 | RPQIGVVREFLTRNP | 96.3 | -0.4904 |  | No | Class II |
| 5821 | 5835 | REFLTRNPAWRKAVF | 23.65 | -0.6716 |  | No | Class II |
| 5828 | 5842 | PAWRKAVFISPYNSQ | 118.83 | 0.5153 |  | No | Class II |
| 5836 | 5850 | ISPYNSQNAVASKIL | 223.99 | 0.4752 |  | No | Class II |
| 5841 | 5855 | SQNAVASKILGLPTQ | 210.75 | 0.3251 |  | No | Class II |
| 5846 | 5860 | ASKILGLPTQTVDSS | 917.97 | 0.2969 |  | No | Class II |
| 5860 | 5874 | SQGSEYDYVIFTQTT | 479.48 | 0.8529 |  | No | Class II |
| 5864 | 5878 | EYDYVIFTQTTETAH | 886.1 | 0.946 |  | No | Class II |
| 5865 | 5879 | YDYVIFTQTTETAHS | 548.13 | 0.7983 |  | No | Class II |
| 5871 | 5885 | TQTTETAHSCNVNRF | 603.15 | -0.0163 |  | No | Class II |
| 5877 | 5891 | AHSCNVNRFNVAITR | 142.59 | -0.1512 |  | No | Class II |
| 5882 | 5896 | VNRFNVAITRAKVGI | 76.1 | 0.5721 |  | No | Class II |
| 5883 | 5897 | NRFNVAITRAKVGIL | 225.94 | 0.7899 |  | No | Class II |
| 5887 | 5901 | VAITRAKVGILCIMS | 151.11 | 1.5174 |  | No | Class II |
| 5890 | 5904 | TRAKVGILCIMSDRD | 929.81 | 1.6491 |  | No | Class II |
| 5892 | 5906 | AKVGILCIMSDRDLY | 329.44 | 1.5744 |  | No | Class II |
| 5895 | 5909 | GILCIMSDRDLYDKL | 408.07 | 0.6503 |  | No | Class II |
| 5897 | 5911 | LCIMSDRDLYDKLQF | 232.31 | 0.7118 |  | No | Class II |
| 5903 | 5917 | RDLYDKLQFTSLEIP | 404.3 | 0.6446 |  | No | Class II |
| 5905 | 5919 | LYDKLQFTSLEIPRR | 723.27 | 0.9199 |  | No | Class II |
| 5908 | 5922 | KLQFTSLEIPRRNVA | 113.04 | 1.0177 |  | No | Class II |
| 5913 | 5927 | SLEIPRRNVATLQAE | 663.95 | 0.3095 |  | No | Class II |
| 5915 | 5929 | EIPRRNVATLQAENV | 178.59 | 0.2347 |  | No | Class II |
| 5918 | 5932 | RRNVATLQAENVTGL | 442.73 | 0.538 |  | No | Class II |
| 5920 | 5934 | NVATLQAENVTGLFK | 439.23 | 0.3315 |  | No | Class II |
| 5929 | 5943 | VTGLFKDCSKVITGL | 418.75 | -0.3521 |  | No | Class II |
| 5937 | 5951 | SKVITGLHPTQAPTH | 550.65 | 0.4236 |  | No | Class II |
| 5942 | 5956 | GLHPTQAPTHLSVDT | 695.73 | 0.6706 |  | No | Class II |
| 5954 | 5968 | VDTKFKTEGLCVDIP | 973.87 | 1.0218 |  | No | Class II |
| 5960 | 5974 | TEGLCVDIPGIPKDM | 940.66 | -0.4995 |  | No | Class II |
| 5967 | 5981 | IPGIPKDMTYRRLIS | 196.23 | -0.452 |  | No | Class II |
| 5972 | 5986 | KDMTYRRLISMMGFK | 941.32 | 0.3405 |  | No | Class II |
| 5977 | 5991 | RRLISMMGFKMNYQV | 384.62 | 0.5569 |  | No | Class II |
| 5978 | 5992 | RLISMMGFKMNYQVN | 77.68 | 0.7686 |  | No | Class II |
| 5987 | 6001 | MNYQVNGYPNMFITR | 25.51 | 0.3169 |  | No | Class II |
| 5988 | 6002 | NYQVNGYPNMFITRE | 443.92 | 0.3896 |  | No | Class II |
| 5993 | 6007 | GYPNMFITREEAIRH | 723.31 | -0.0459 |  | No | Class II |
| 5996 | 6010 | NMFITREEAIRHVRA | 166.81 | 0.1263 |  | No | Class II |
| 6000 | 6014 | TREEAIRHVRAWIGF | 251.7 | -0.0277 |  | No | Class II |
| 6003 | 6017 | EAIRHVRAWIGFDVE | 11.5 | 0.3307 |  | No | Class II |
| 6005 | 6019 | IRHVRAWIGFDVEGC | 430.49 | -0.1032 |  | No | Class II |
| 6009 | 6023 | RAWIGFDVEGCHATR | 844.66 | 0.1477 |  | No | Class II |
| 6023 | 6037 | REAVGTNLPLQLGFS | 108.52 | 0.962 |  | No | Class II |
| 6024 | 6038 | EAVGTNLPLQLGFST | 559.39 | 1.0387 |  | No | Class II |
| 6033 | 6047 | QLGFSTGVNLVAVPT | 139.44 | 0.7433 |  | No | Class II |
| 6038 | 6052 | TGVNLVAVPTGYVDT | 539.1 | 0.568 |  | No | Class II |
| 6046 | 6060 | PTGYVDTPNNTDFSR | 445.34 | 0.1231 |  | No | Class II |
| 6053 | 6067 | PNNTDFSRVSAKPPP | 652.94 | 1.2209 |  | No | Class II |
| 6069 | 6083 | DQFKHLIPLMYKGLP | 47.33 | 0.3983 |  | No | Class II |
| 6075 | 6089 | IPLMYKGLPWNVVRI | 162.5 | 0.8293 |  | No | Class II |
| 6081 | 6095 | GLPWNVVRIKIVQML | 150.93 | 0.5871 |  | No | Class II |
| 6086 | 6100 | VVRIKIVQMLSDTLK | 273.99 | 0.1768 |  | No | Class II |
| 6091 | 6105 | IVQMLSDTLKNLSDR | 301.75 | -0.1269 |  | No | Class II |
| 6096 | 6110 | SDTLKNLSDRVVFVL | 93.87 | 0.0313 |  | No | Class II |
| 6101 | 6115 | NLSDRVVFVLWAHGF | 906.85 | 0.2154 |  | No | Class II |
| 6105 | 6119 | RVVFVLWAHGFELTS | 122.09 | 0.352 |  | No | Class II |
| 6106 | 6120 | VVFVLWAHGFELTSM | 334.82 | 0.3776 |  | No | Class II |
| 6112 | 6126 | AHGFELTSMKYFVKI | 214.93 | 0.3189 |  | No | Class II |
| 6136 | 6150 | DRRATCFSTASDTYA | 570.18 | 0.8802 |  | No | Class II |
| 6139 | 6153 | ATCFSTASDTYACWH | 239.26 | 0.8016 |  | No | Class II |
| 6142 | 6156 | FSTASDTYACWHHSI | 775.51 | 0.5144 |  | No | Class II |
| 6149 | 6163 | YACWHHSIGFDYVYN | 70.55 | 0.9877 |  | No | Class II |
| 6155 | 6169 | SIGFDYVYNPFMIDV | 72.57 | 1.0823 |  | No | Class II |
| 6156 | 6170 | IGFDYVYNPFMIDVQ | 270.11 | 1.2501 |  | No | Class II |
| 6161 | 6175 | VYNPFMIDVQQWGFT | 901.82 | 0.5069 |  | No | Class II |
| 6162 | 6176 | YNPFMIDVQQWGFTG | 554.5 | 0.9722 |  | No | Class II |
| 6168 | 6182 | DVQQWGFTGNLQSNH | 280.47 | 0.4947 |  | No | Class II |
| 6175 | 6189 | TGNLQSNHDLYCQVH | 671.66 | 0.7223 |  | No | Class II |
| 6182 | 6196 | HDLYCQVHGNAHVAS | 295.44 | 0.3456 |  | No | Class II |
| 6184 | 6198 | LYCQVHGNAHVASCD | 311.27 | -0.0042 |  | No | Class II |
| 6189 | 6203 | HGNAHVASCDAIMTR | 435.83 | -0.0175 |  | No | Class II |
| 6194 | 6208 | VASCDAIMTRCLAVH | 659.17 | 0.1644 |  | No | Class II |
| 6201 | 6215 | MTRCLAVHECFVKRV | 812.12 | -0.3329 |  | No | Class II |
| 6206 | 6220 | AVHECFVKRVDWTIE | 793.93 | 0.8561 |  | No | Class II |
| 6210 | 6224 | CFVKRVDWTIEYPII | 629.13 | 1.089 |  | No | Class II |
| 6211 | 6225 | FVKRVDWTIEYPIIG | 425.53 | 1.1634 |  | No | Class II |
| 6215 | 6229 | VDWTIEYPIIGDELK | 965.96 | 0.8426 |  | No | Class II |
| 6217 | 6231 | WTIEYPIIGDELKIN | 791.18 | 0.2807 |  | No | Class II |
| 6222 | 6236 | PIIGDELKINAACRK | 168.14 | 0.7389 |  | No | Class II |
| 6227 | 6241 | ELKINAACRKVQHMV | 70.78 | 1.1171 |  | No | Class II |
| 6235 | 6249 | RKVQHMVVKAALLAD | 213.21 | 0.3167 |  | No | Class II |
| 6240 | 6254 | MVVKAALLADKFPVL | 415.28 | 0.4032 |  | No | Class II |
| 6241 | 6255 | VVKAALLADKFPVLH | 179.47 | 0.3295 |  | No | Class II |
| 6250 | 6264 | KFPVLHDIGNPKAIK | 96.4 | -0.1166 |  | No | Class II |
| 6260 | 6274 | PKAIKCVPQADVEWK | 712.49 | 1.0603 |  | No | Class II |
| 6269 | 6283 | ADVEWKFYDAQPCSD | 910.8 | 1.3889 |  | No | Class II |
| 6270 | 6284 | DVEWKFYDAQPCSDK | 593.11 | 1.1388 |  | No | Class II |
| 6282 | 6296 | SDKAYKIEELFYSYA | 496.24 | 0.44 |  | No | Class II |
| 6283 | 6297 | DKAYKIEELFYSYAT | 207.51 | 0.3865 |  | No | Class II |
| 6288 | 6302 | IEELFYSYATHSDKF | 489.02 | -0.0337 |  | No | Class II |
| 6297 | 6311 | THSDKFTDGVCLFWN | 716.48 | 0.0506 |  | No | Class II |
| 6303 | 6317 | TDGVCLFWNCNVDRY | 635.48 | -0.4923 |  | No | Class II |
| 6311 | 6325 | NCNVDRYPANSIVCR | 71.31 | -0.2714 |  | No | Class II |
| 6315 | 6329 | DRYPANSIVCRFDTR | 471.07 | 0.5344 |  | No | Class II |
| 6316 | 6330 | RYPANSIVCRFDTRV | 445.37 | 0.3223 |  | No | Class II |
| 6321 | 6335 | SIVCRFDTRVLSNLN | 70.98 | 0.7834 |  | No | Class II |
| 6326 | 6340 | FDTRVLSNLNLPGCD | 102.5 | -0.3284 |  | No | Class II |
| 6336 | 6350 | LPGCDGGSLYVNKHA | 723.46 | -0.421 |  | No | Class II |
| 6337 | 6351 | PGCDGGSLYVNKHAF | 492.85 | -0.2665 |  | No | Class II |
| 6342 | 6356 | GSLYVNKHAFHTPAF | 630.78 | -0.2587 |  | No | Class II |
| 6348 | 6362 | KHAFHTPAFDKSAFV | 782.01 | -0.3179 |  | No | Class II |
| 6353 | 6367 | TPAFDKSAFVNLKQL | 240.84 | 0.3572 |  | No | Class II |
| 6364 | 6378 | LKQLPFFYYSDSPCE | 416.17 | 0.5514 |  | No | Class II |
| 6366 | 6380 | QLPFFYYSDSPCESH | 74.53 | 0.2953 |  | No | Class II |
| 6379 | 6393 | SHGKQVVSDIDYVPL | 736.61 | 0.9016 |  | No | Class II |
| 6381 | 6395 | GKQVVSDIDYVPLKS | 247.02 | 0.9913 |  | No | Class II |
| 6387 | 6401 | DIDYVPLKSATCITR | 761.29 | 1.1807 |  | No | Class II |
| 6399 | 6413 | ITRCNLGGAVCRHHA | 181.31 | 0.5434 |  | No | Class II |
| 6406 | 6420 | GAVCRHHANEYRLYL | 149.77 | 0.0396 |  | No | Class II |
| 6409 | 6423 | CRHHANEYRLYLDAY | 867.91 | -0.3798 |  | No | Class II |
| 6414 | 6428 | NEYRLYLDAYNMMIS | 194.77 | -0.04 |  | No | Class II |
| 6415 | 6429 | EYRLYLDAYNMMISA | 125.97 | 0.1293 |  | No | Class II |
| 6419 | 6433 | YLDAYNMMISAGFSL | 180.49 | 0.664 |  | No | Class II |
| 6421 | 6435 | DAYNMMISAGFSLWV | 122.33 | 0.3079 |  | No | Class II |
| 6425 | 6439 | MMISAGFSLWVYKQF | 149.91 | 0.424 |  | No | Class II |
| 6428 | 6442 | SAGFSLWVYKQFDTY | 137.95 | 0.1973 |  | No | Class II |
| 6430 | 6444 | GFSLWVYKQFDTYNL | 867.23 | 0.3389 |  | No | Class II |
| 6433 | 6447 | LWVYKQFDTYNLWNT | 117.09 | 0.1422 |  | No | Class II |
| 6439 | 6453 | FDTYNLWNTFTRLQS | 54.41 | -0.0162 |  | No | Class II |
| 6443 | 6457 | NLWNTFTRLQSLENV | 181.6 | 0.0223 |  | No | Class II |
| 6450 | 6464 | RLQSLENVAFNVVNK | 92.43 | 0.9529 |  | No | Class II |
| 6453 | 6467 | SLENVAFNVVNKGHF | 561.92 | 1.237 |  | No | Class II |
| 6455 | 6469 | ENVAFNVVNKGHFDG | 115.5 | 0.8011 |  | No | Class II |
| 6464 | 6478 | KGHFDGQQGEVPVSI | 698.7 | 0.6284 |  | No | Class II |
| 6466 | 6480 | HFDGQQGEVPVSIIN | 497.22 | 0.4703 |  | No | Class II |
| 6473 | 6487 | EVPVSIINNTVYTKV | 26.92 | 0.1398 |  | No | Class II |
| 6481 | 6495 | NTVYTKVDGVDVELF | 60.29 | 0.2425 |  | No | Class II |
| 6482 | 6496 | TVYTKVDGVDVELFE | 87.7 | 0.2535 |  | No | Class II |
| 6487 | 6501 | VDGVDVELFENKTTL | 688.03 | 0.3404 |  | No | Class II |
| 6492 | 6506 | VELFENKTTLPVNVA | 391.83 | 0.3429 |  | No | Class II |
| 6495 | 6509 | FENKTTLPVNVAFEL | 408.08 | 1.125 |  | No | Class II |
| 6498 | 6512 | KTTLPVNVAFELWAK | 142.69 | 0.6072 |  | No | Class II |
| 6501 | 6515 | LPVNVAFELWAKRNI | 708.99 | 0.7645 |  | No | Class II |
| 6504 | 6518 | NVAFELWAKRNIKPV | 240.74 | 1.4 |  | No | Class II |
| 6511 | 6525 | AKRNIKPVPEVKILN | 212.93 | 1.3742 |  | No | Class II |
| 6523 | 6537 | ILNNLGVDIAANTVI | 44.42 | -0.0115 |  | No | Class II |
| 6528 | 6542 | GVDIAANTVIWDYKR | 482.73 | 0.6285 |  | No | Class II |
| 6534 | 6548 | NTVIWDYKRDAPAHI | 148.33 | 0.5217 |  | No | Class II |
| 6539 | 6553 | DYKRDAPAHISTIGV | 546.69 | 0.8259 |  | No | Class II |
| 6540 | 6554 | YKRDAPAHISTIGVC | 546.03 | 0.4753 |  | No | Class II |
| 6545 | 6559 | PAHISTIGVCSMTDI | 244.11 | 0.6635 |  | No | Class II |
| 6559 | 6573 | IAKKPTETICAPLTV | 801.8 | 0.2445 |  | No | Class II |
| 6561 | 6575 | KKPTETICAPLTVFF | 644.99 | -0.1343 |  | No | Class II |
| 6564 | 6578 | TETICAPLTVFFDGR | 783.56 | 0.1695 |  | No | Class II |
| 6566 | 6580 | TICAPLTVFFDGRVD | 276.02 | 0.0497 |  | No | Class II |
| 6569 | 6583 | APLTVFFDGRVDGQV | 117.43 | -0.1568 |  | No | Class II |
| 6571 | 6585 | LTVFFDGRVDGQVDL | 921.7 | 0.0322 |  | No | Class II |
| 6577 | 6591 | GRVDGQVDLFRNARN | 151.85 | -0.3907 |  | No | Class II |
| 6582 | 6596 | QVDLFRNARNGVLIT | 41.24 | 0.2663 |  | No | Class II |
| 6586 | 6600 | FRNARNGVLITEGSV | 428.34 | 0.8234 |  | No | Class II |
| 6590 | 6604 | RNGVLITEGSVKGLQ | 86.53 | 0.5861 |  | No | Class II |
| 6592 | 6606 | GVLITEGSVKGLQPS | 246.34 | 0.6465 |  | No | Class II |
| 6595 | 6609 | ITEGSVKGLQPSVGP | 489.86 | 0.6876 |  | No | Class II |
| 6597 | 6611 | EGSVKGLQPSVGPKQ | 856.16 | 0.7804 |  | No | Class II |
| 6600 | 6614 | VKGLQPSVGPKQASL | 487.79 | 1.1194 |  | No | Class II |
| 6606 | 6620 | SVGPKQASLNGVTLI | 588.46 | 1.0205 |  | No | Class II |
| 6609 | 6623 | PKQASLNGVTLIGEA | 169.59 | 0.4691 |  | No | Class II |
| 6611 | 6625 | QASLNGVTLIGEAVK | 78.17 | 0.3529 |  | No | Class II |
| 6615 | 6629 | NGVTLIGEAVKTQFN | 571.27 | 0.4408 |  | No | Class II |
| 6616 | 6630 | GVTLIGEAVKTQFNY | 277.7 | 0.7763 |  | No | Class II |
| 6620 | 6634 | IGEAVKTQFNYYKKV | 155.91 | 0.5682 |  | No | Class II |
| 6627 | 6641 | QFNYYKKVDGVVQQL | 127.33 | 0.134 |  | No | Class II |
| 6632 | 6646 | KKVDGVVQQLPETYF | 849.99 | -0.1067 |  | No | Class II |
| 6634 | 6648 | VDGVVQQLPETYFTQ | 473.75 | -0.205 |  | No | Class II |
| 6642 | 6656 | PETYFTQSRNLQEFK | 101.24 | 0.1265 |  | No | Class II |
| 6649 | 6663 | SRNLQEFKPRSQMEI | 818.7 | 0.7894 |  | No | Class II |
| 6655 | 6669 | FKPRSQMEIDFLELA | 274.43 | 1.5183 |  | No | Class II |
| 6658 | 6672 | RSQMEIDFLELAMDE | 241.73 | 0.956 |  | No | Class II |
| 6662 | 6676 | EIDFLELAMDEFIER | 87.22 | 0.5475 |  | No | Class II |
| 6665 | 6679 | FLELAMDEFIERYKL | 112.23 | -0.0633 |  | No | Class II |
| 6670 | 6684 | MDEFIERYKLEGYAF | 721.07 | -0.0955 |  | No | Class II |
| 6675 | 6689 | ERYKLEGYAFEHIVY | 391.1 | 0.3774 |  | No | Class II |
| 6679 | 6693 | LEGYAFEHIVYGDFS | 174.67 | 0.7431 |  | No | Class II |
| 6684 | 6698 | FEHIVYGDFSHSQLG | 611.17 | 1.283 |  | No | Class II |
| 6689 | 6703 | YGDFSHSQLGGLHLL | 848.85 | 1.0934 |  | No | Class II |
| 6694 | 6708 | HSQLGGLHLLIGLAK | 233.93 | 0.8929 |  | No | Class II |
| 6698 | 6712 | GGLHLLIGLAKRFKE | 65.71 | 0.7112 |  | No | Class II |
| 6699 | 6713 | GLHLLIGLAKRFKES | 901.92 | 0.7829 |  | No | Class II |
| 6703 | 6717 | LIGLAKRFKESPFEL | 189.21 | 1.1525 |  | No | Class II |
| 6706 | 6720 | LAKRFKESPFELEDF | 720.39 | 1.0638 |  | No | Class II |
| 6711 | 6725 | KESPFELEDFIPMDS | 513.93 | 0.9596 |  | No | Class II |
| 6712 | 6726 | ESPFELEDFIPMDST | 321.74 | 0.8756 |  | No | Class II |
| 6717 | 6731 | LEDFIPMDSTVKNYF | 111.3 | 0.2559 |  | No | Class II |
| 6722 | 6736 | PMDSTVKNYFITDAQ | 517.16 | 0.319 |  | No | Class II |
| 6723 | 6737 | MDSTVKNYFITDAQT | 813.04 | 0.1779 |  | No | Class II |
| 6727 | 6741 | VKNYFITDAQTGSSK | 70.03 | 0.3701 |  | No | Class II |
| 6729 | 6743 | NYFITDAQTGSSKCV | 217.92 | 0.3626 |  | No | Class II |
| 6734 | 6748 | DAQTGSSKCVCSVID | 498.7 | 0.5231 |  | No | Class II |
| 6738 | 6752 | GSSKCVCSVIDLLLD | 917.7 | 0.1747 |  | No | Class II |
| 6740 | 6754 | SKCVCSVIDLLLDDF | 429.74 | 0.1049 |  | No | Class II |
| 6745 | 6759 | SVIDLLLDDFVEIIK | 241.08 | -0.213 |  | No | Class II |
| 6746 | 6760 | VIDLLLDDFVEIIKS | 301.56 | -0.2651 |  | No | Class II |
| 6751 | 6765 | LDDFVEIIKSQDLSV | 115.49 | 0.5165 |  | No | Class II |
| 6752 | 6766 | DDFVEIIKSQDLSVV | 612.16 | 0.7838 |  | No | Class II |
| 6758 | 6772 | IKSQDLSVVSKVVKV | 761.51 | 1.1986 |  | No | Class II |
| 6768 | 6782 | KVVKVTIDYTEISFM | 105.05 | 1.2331 |  | No | Class II |
| 6769 | 6783 | VVKVTIDYTEISFML | 570.43 | 1.2925 |  | No | Class II |
| 6776 | 6790 | YTEISFMLWCKDGHV | 790.53 | 0.9594 |  | No | Class II |
| 6781 | 6795 | FMLWCKDGHVETFYP | 960.33 | 0.0882 |  | No | Class II |
| 6789 | 6803 | HVETFYPKLQSSQAW | 456.46 | -0.0043 |  | No | Class II |
| 6794 | 6808 | YPKLQSSQAWQPGVA | 405.18 | 0.2599 |  | No | Class II |
| 6800 | 6814 | SQAWQPGVAMPNLYK | 37.18 | -0.1018 |  | No | Class II |
| 6808 | 6822 | AMPNLYKMQRMLLEK | 125.44 | -0.509 |  | No | Class II |
| 6813 | 6827 | YKMQRMLLEKCDLQN | 963.39 | -0.3819 |  | No | Class II |
| 6822 | 6836 | KCDLQNYGDSATLPK | 790.85 | -0.0707 |  | No | Class II |
| 6829 | 6843 | GDSATLPKGIMMNVA | 604.64 | -0.2591 |  | No | Class II |
| 6832 | 6846 | ATLPKGIMMNVAKYT | 175.81 | -0.2841 |  | No | Class II |
| 6834 | 6848 | LPKGIMMNVAKYTQL | 15.4 | -0.0205 |  | No | Class II |
| 6840 | 6854 | MNVAKYTQLCQYLNT | 109.07 | 0.3568 |  | No | Class II |
| 6845 | 6859 | YTQLCQYLNTLTLAV | 40.13 | 0.1863 |  | No | Class II |
| 6851 | 6865 | YLNTLTLAVPYNMRV | 141.51 | 0.3613 |  | No | Class II |
| 6855 | 6869 | LTLAVPYNMRVIHFG | 464.15 | 1.4443 |  | No | Class II |
| 6859 | 6873 | VPYNMRVIHFGAGSD | 903.42 | 1.3749 |  | No | Class II |
| 6860 | 6874 | PYNMRVIHFGAGSDK | 487.9 | 1.4221 |  | No | Class II |
| 6864 | 6878 | RVIHFGAGSDKGVAP | 327.41 | 1.1174 |  | No | Class II |
| 6873 | 6887 | DKGVAPGTAVLRQWL | 140.01 | 0.201 |  | No | Class II |
| 6882 | 6896 | VLRQWLPTGTLLVDS | 14.84 | -0.3493 |  | No | Class II |
| 6883 | 6897 | LRQWLPTGTLLVDSD | 517.89 | -0.3317 |  | No | Class II |
| 6888 | 6902 | PTGTLLVDSDLNDFV | 431.15 | -0.1044 |  | No | Class II |
| 6889 | 6903 | TGTLLVDSDLNDFVS | 220.13 | -0.0309 |  | No | Class II |
| 6896 | 6910 | SDLNDFVSDADSTLI | 246.41 | -0.0388 |  | No | Class II |
| 6897 | 6911 | DLNDFVSDADSTLIG | 496.85 | 0.1951 |  | No | Class II |
| 6907 | 6921 | STLIGDCATVHTANK | 583.67 | 0.072 |  | No | Class II |
| 6913 | 6927 | CATVHTANKWDLIIS | 607.52 | 0.733 |  | No | Class II |
| 6918 | 6932 | TANKWDLIISDMYDP | 533.01 | 0.5272 |  | No | Class II |
| 6921 | 6935 | KWDLIISDMYDPKTK | 329.86 | 0.7384 |  | No | Class II |
| 6945 | 6959 | EGFFTYICGFIQQKL | 149.17 | -0.0199 |  | No | Class II |
| 6947 | 6961 | FFTYICGFIQQKLAL | 363.61 | 0.192 |  | No | Class II |
| 6951 | 6965 | ICGFIQQKLALGGSV | 53.69 | 0.644 |  | No | Class II |
| 6962 | 6976 | GGSVAIKITEHSWNA | 524.11 | 1.1578 |  | No | Class II |
| 6968 | 6982 | KITEHSWNADLYKLM | 853.13 | -0.1827 |  | No | Class II |
| 6970 | 6984 | TEHSWNADLYKLMGH | 881.03 | -0.3781 |  | No | Class II |
| 6975 | 6989 | NADLYKLMGHFAWWT | 76.18 | -0.1408 |  | No | Class II |
| 6976 | 6990 | ADLYKLMGHFAWWTA | 938.46 | -0.0591 |  | No | Class II |
| 6981 | 6995 | LMGHFAWWTAFVTNV | 312.74 | 0.3144 |  | No | Class II |
| 6982 | 6996 | MGHFAWWTAFVTNVN | 95.4 | 0.6284 |  | No | Class II |
| 6986 | 7000 | AWWTAFVTNVNASSS | 353.14 | 0.2138 |  | No | Class II |
| 6988 | 7002 | WTAFVTNVNASSSEA | 64.4 | 0.3444 |  | No | Class II |
| 6991 | 7005 | FVTNVNASSSEAFLI | 119.06 | 0.1783 |  | No | Class II |
| 6993 | 7007 | TNVNASSSEAFLIGC | 191.54 | 0.3429 |  | No | Class II |
| 6996 | 7010 | NASSSEAFLIGCNYL | 592.06 | 0.2123 |  | No | Class II |
| 7000 | 7014 | SEAFLIGCNYLGKPR | 54.01 | 0.3284 |  | No | Class II |
| 7001 | 7015 | EAFLIGCNYLGKPRE | 992.93 | 0.4176 |  | No | Class II |
| 7011 | 7025 | GKPREQIDGYVMHAN | 991.94 | 0.0028 |  | No | Class II |
| 7014 | 7028 | REQIDGYVMHANYIF | 430.02 | 0.1154 |  | No | Class II |
| 7016 | 7030 | QIDGYVMHANYIFWR | 69.62 | 0.0023 |  | No | Class II |
| 7024 | 7038 | ANYIFWRNTNPIQLS | 177.37 | 1.0311 |  | No | Class II |
| 7029 | 7043 | WRNTNPIQLSSYSLF | 188.13 | 0.9997 |  | No | Class II |
| 7034 | 7048 | PIQLSSYSLFDMSKF | 305.35 | 0.6304 |  | No | Class II |
| 7039 | 7053 | SYSLFDMSKFPLKLR | 144.98 | 0.9396 |  | No | Class II |
| 7047 | 7061 | KFPLKLRGTAVMSLK | 364.69 | 1.2277 |  | No | Class II |
| 7048 | 7062 | FPLKLRGTAVMSLKE | 87.95 | 1.4136 |  | No | Class II |
| 7052 | 7066 | LRGTAVMSLKEGQIN | 649.42 | 1.2164 |  | No | Class II |
| 7061 | 7075 | KEGQINDMILSLLSK | 388.14 | 0.704 |  | No | Class II |
| 7063 | 7077 | GQINDMILSLLSKGR | 212.05 | 0.7779 |  | No | Class II |
| 7067 | 7081 | DMILSLLSKGRLIIR | 50.79 | 0.6687 |  | No | Class II |
| 7068 | 7082 | MILSLLSKGRLIIRE | 947.88 | 0.5843 |  | No | Class II |
| 7080 | 7094 | IRENNRVVISSDVLV | 274.4 | 0.3209 |  | No | Class II |
| 7082 | 7096 | ENNRVVISSDVLVNN | 42.94 | -0.0527 |  | No | Class II |
