# Supplementary material for: Design and generation of mRNAs encoding conserved regions of SARS-CoV-2 ORF1ab for T cell-mediated immune activation
Source: Future Virol. 2023 Jun 24;18(8):501–16. doi: 10.2217/fvl-2023-0066 (PMC10308627; doi:10.2217/fvl-2023-0066)
Supplement: Supplementary file 9 [file fvl-18-501-s9.docx]

| **Table S3: Participants information** | | | | | |  |  | |  | |  |  |  |  |  | |
| --- | --- | --- | --- | --- | --- | --- | --- | --- | --- | --- | --- | --- | --- | --- | --- | --- |
|  |  |  |  | Vaccine day | | | Type of vaccine | | | Covid infection | | Blood draw day | Blood draw - last vaccine (day) | Blood draw - last infect (day) | HLA | |
| Donor | Group | Age | Gender | 1st | 2nd | 3rd | 1st | 2nd | 3rd | 1st | 2nd |  |  |  | Allele 1 | Allele 2 |
| 1 | < 1 month | 25 | M | 07/28/21 | 09/29/21 | 01/28/22 | A | A | P | 07/18/22 | 04/27/23 | 05/08/23 | 465 | 11 |  |  |
| 2 | < 1 month | 30 | M | 07/29/21 | 09/26/21 | 12/28/21 | A | P | P | 08/10/22 | 04/18/23 | 05/08/23 | 496 | 20 |  |  |
| 3 | < 1 month | 24 | M | 06/12/21 | 08/22/21 | 01/20/22 | A | A | P | 08/20/22 | 04/10/23 | 05/08/23 | 473 | 28 |  |  |
| 4 | < 1 month | 25 | F | 02/01/21 | 03/01/21 | 07/02/21 | A | P | P | 09/01/22 | 04/15/23 | 05/08/23 | 675 | 23 |  |  |
| 5 | < 1 month | 25 | F | 09/19/21 | 11/01/21 | 01/23/22 | A | A | P | 03/01/22 | 08/12/22 | 08/15/22 | 204 | 3 | 11:01 | 11:01 |
| 6 | < 1 month | 32 | M | 09/08/21 | 11/04/22 | 02/07/22 | A | A | P | 08/02/22 |  | 08/15/22 | 189 | 13 | 33:03 | 33:03 |
| 7 | < 1 month | 23 | F | 08/10/21 | 09/10/21 | 01/28/22 | C | C | P | 04/23/23 |  | 05/08/23 | 465 | 15 |  |  |
| 8 | < 1 month | 23 | F | 07/10/21 | 09/11/21 | 01/15/22 | A | A | A | 03/10/23 | 04/15/23 | 05/08/23 | 478 | 23 |  |  |
| 9 | < 1 month | 28 | F | 06/29/21 | 09/17/21 | 01/05/22 | A | A | A | 10/01/22 | 08/01/22 | 08/15/22 | 222 | 14 | 24:02 | 02:07 |
| 10 | < 1 month | 33 | F | 04/19/21 | 05/20/21 | 01/25/22 | M | M | M | 04/28/23 |  | 05/08/23 | 468 | 10 |  |  |
| 11 | < 1 month | 24 | F | 06/26/21 | 08/27/21 | 12/29/21 | A | A | P | 03/08/22 | 04/22/23 | 05/08/23 | 495 | 16 |  |  |
| 12 | < 1 month | 31 | F | 02/14/21 | 05/16/21 | 12/20/21 | A | P | P | 04/23/23 |  | 05/08/23 | 504 | 15 |  |  |
| 13 | < 1 month | 32 | M | 07/29/21 | 09/23/21 | 01/04/22 | A | A | P | 07/14/22 |  | 08/15/22 | 223 | 32 | 24:02 | 24:03 |
| 14 | < 1 month | 29 | F | 07/29/21 | 09/23/21 | 01/04/22 | A | P | P | 03/10/22 | 04/27/23 | 05/08/23 | 489 | 11 |  |  |
| 15 | > 1 month | 23 | F | 07/18/21 | 08/19/21 | 02/20/22 | A | A | P | 03/15/22 |  | 05/08/23 | 442 | 419 |  |  |
| 16 | > 1 month | 28 | M | 08/12/21 | 11/10/21 | 02/21/22 | A | P | P | 08/10/22 |  | 05/08/23 | 441 | 271 |  |  |
| 17 | > 1 month | 39 | M | 07/29/21 | 09/25/21 | 01/04/22 | A | A | P | 07/20/22 |  | 05/08/23 | 489 | 292 |  |  |
| 18 | > 1 month | 23 | F | 10/21/21 | 11/25/21 | 01/29/22 | C | C | P | 03/06/22 |  | 05/08/23 | 464 | 428 |  |  |
| 19 | > 1 month | 29 | M | 08/03/21 | 09/28/21 | 01/18/22 | A | A | A | 08/12/22 |  | 05/08/23 | 475 | 269 |  |  |
| 20 | > 1 month | 24 | M | 08/26/21 | 10/06/21 | 01/13/22 | A | A | P | 10/28/21 | 02/01/22 | 09/20/22 | 250 | 231 | 11:01 | 11:04 |
| 21 | > 1 month | 24 | M | 07/19/21 | 10/04/21 | 01/16/22 | A | A | M | 03/12/22 |  | 05/08/23 | 477 | 422 |  |  |
| 22 | > 1 month | 23 | M | 08/15/21 | 10/26/21 | 01/22/22 | A | A | P | 03/10/22 |  | 09/15/22 | 236 | 189 | 02:01 | 02:06 |
| 23 | > 1 month | 25 | M | 07/29/21 | 10/05/21 | 01/22/22 | A | A | P | 03/28/22 |  | 10/11/22 | 262 | 197 | 02:07 | 24:02 |
| 24 | > 1 month | 22 | F | 07/10/21 | 08/12/21 | 02/20/22 | C | C | M | 12/15/22 |  | 05/08/23 | 442 | 144 |  |  |
| 25 | > 1 month | 24 | F | 07/14/21 | 08/15/21 | 02/20/22 | P | P | P | 08/20/22 |  | 05/08/23 | 442 | 261 |  |  |
| 26 | > 1 month | 40 | F | 07/29/21 | 09/25/21 |  | A | P |  | 11/27/21 | 04/01/22 | 09/15/22 | 355 | 167 | 02:01 | 02:06 |
| 27 | Unknown | 30 | M | 03/31/21 | 04/28/21 | 12/30/21 | M | M | M |  |  | 05/08/23 | 494 | N/A |  |  |
| 28 | Unknown | 26 | F | 06/29/21 | 08/31/21 | 02/03/22 | A | A | P |  |  | 08/15/22 | 193 | N/A | 24:02 | 24:07 |
| 29 | Unknown | 40 | M | 07/07/21 | 08/04/21 |  | M | M |  |  |  | 05/08/23 | 642 | N/A |  |  |
| 30 | Unknown | 25 | M | 07/11/21 | 08/01/21 | 12/30/21 | C | C | A |  |  | 09/15/22 | 259 | N/A | 03:01 | 33:03 |
| 31 | Unknown | 25 | F | 06/25/21 | 09/25/21 | 01/05/22 | M | P | P |  |  | 09/15/22 | 253 | N/A | 11:01 | 24:02 |
| 32 | Unknown | 24 | F | 08/02/21 | 09/18/21 | 01/15/22 | A | A | P |  |  | 09/20/22 | 248 | N/A | 11:01 | 02:01 |
| 33 | Unknown | 26 | F | 06/29/21 | 09/15/21 | 12/31/21 | A | A | P |  |  | 09/20/22 | 263 | N/A | 11:01 | 11:01 |
| 34 | Unknown | 30 | F | 02/10/21 | 05/10/21 | 12/12/21 | A | P | P |  |  | 10/11/22 | 303 | N/A | 11:01 | 11:01 |

M: male

F: female

A: Astrazeneca

C: CoronaVac (Vero cell)

M: Moderna

P: Pfizer

|  |
| --- |
